# Supplementary figures and images for: 14-3-3 protein augments the protein stability of phosphorylated spastin and promotes the recovery of spinal cord injury through its agonist intervention (part 1 of 2)
Source: eLife. 2024 Jan 17;12:RP90184. doi: 10.7554/eLife.90184 (PMC10945579; doi:10.7554/eLife.90184)

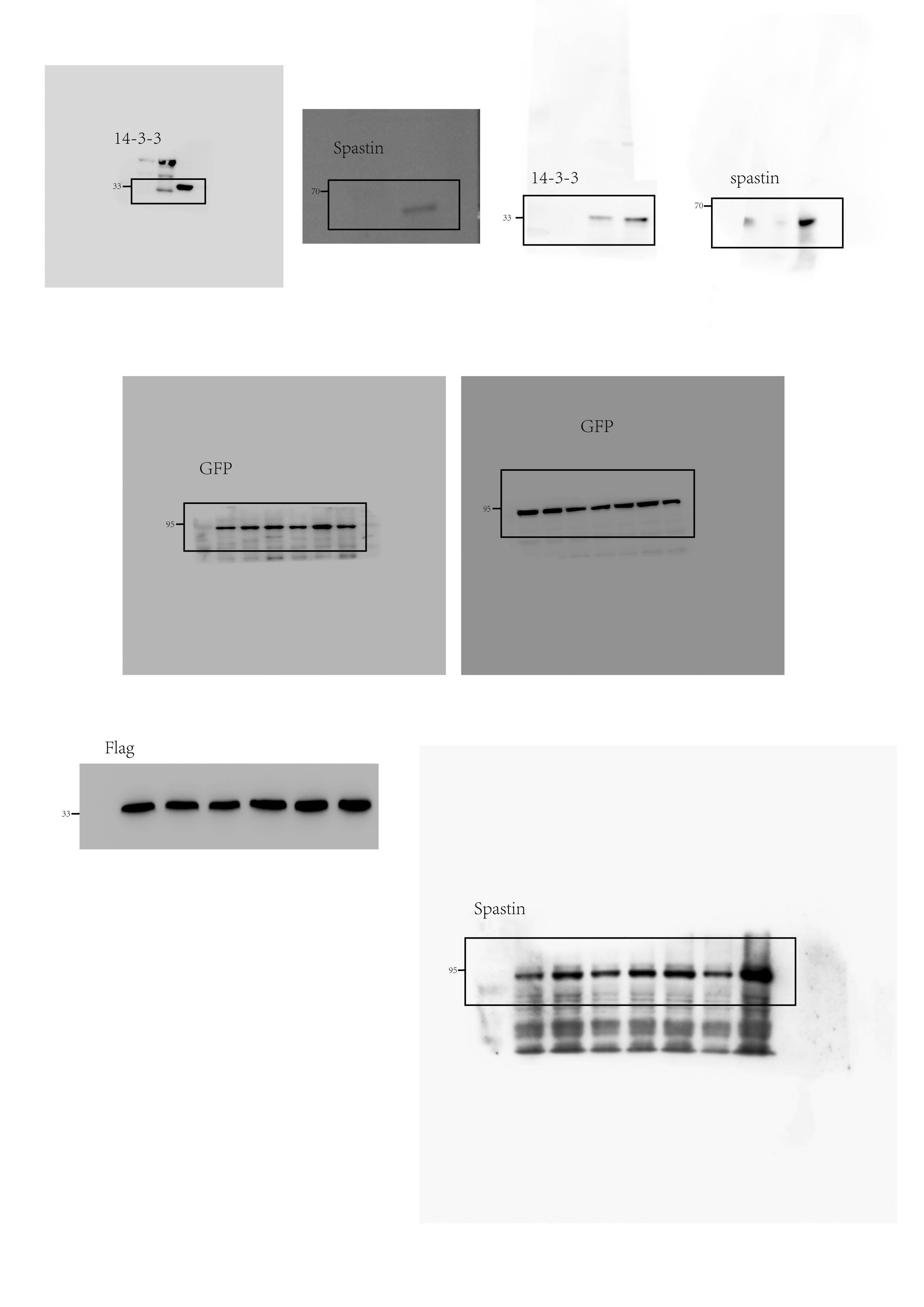

Supplement: Figure 1—source data 2. [file elife-90184-fig1-data2.zip › Raw and annotated immunoblots/Annotated blots.tif]

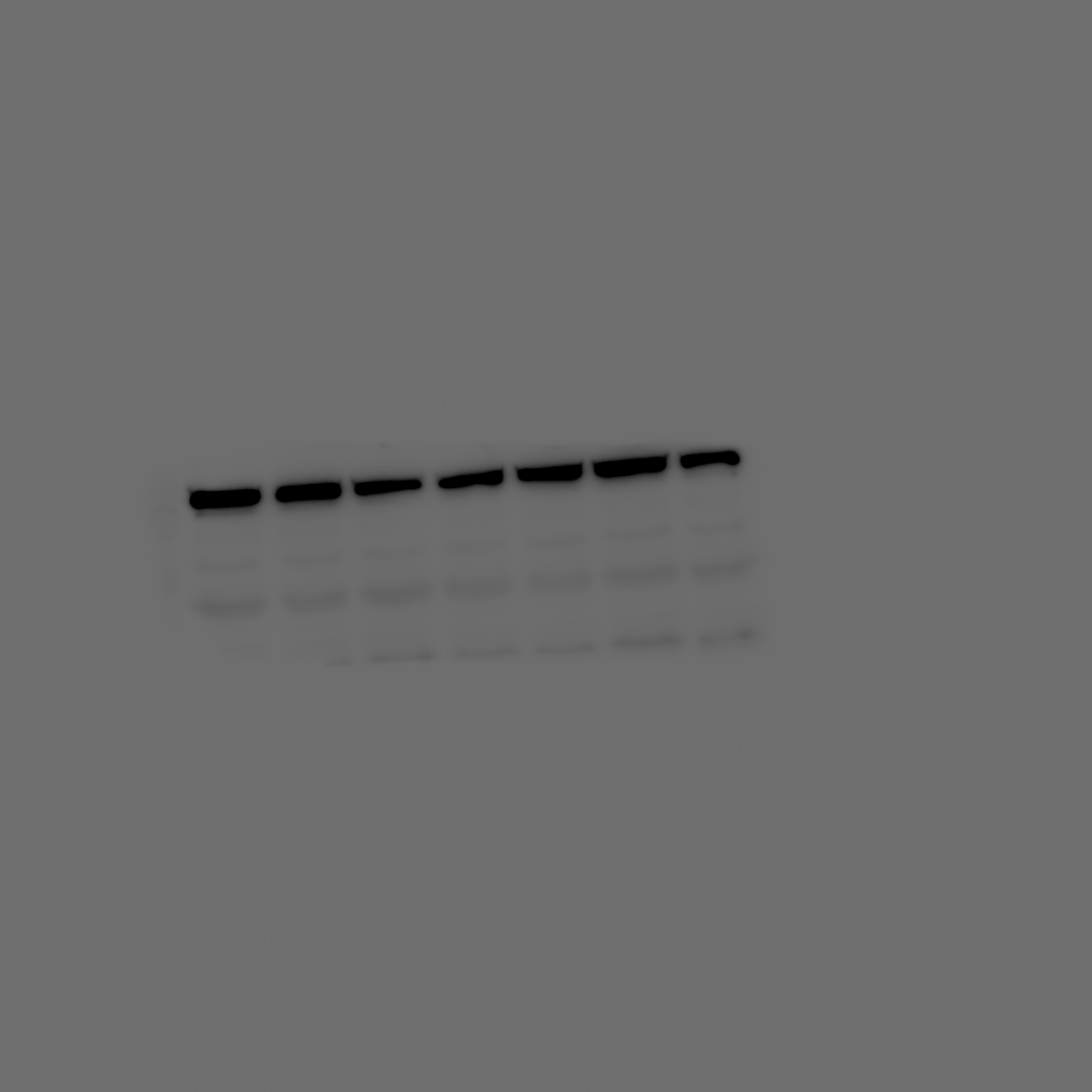

Supplement: Figure 1—source data 2. [file elife-90184-fig1-data2.zip › Raw and annotated immunoblots/Raw blots/Input.tif]

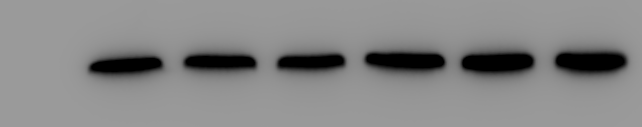

Supplement: Figure 1—source data 2. [file elife-90184-fig1-data2.zip › Raw and annotated immunoblots/Raw blots/IP Flag.tif]

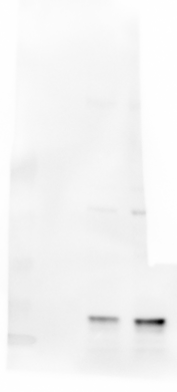

Supplement: Figure 1—source data 2. [file elife-90184-fig1-data2.zip › Raw and annotated immunoblots/Raw blots/IP spastin IB 14.tif]

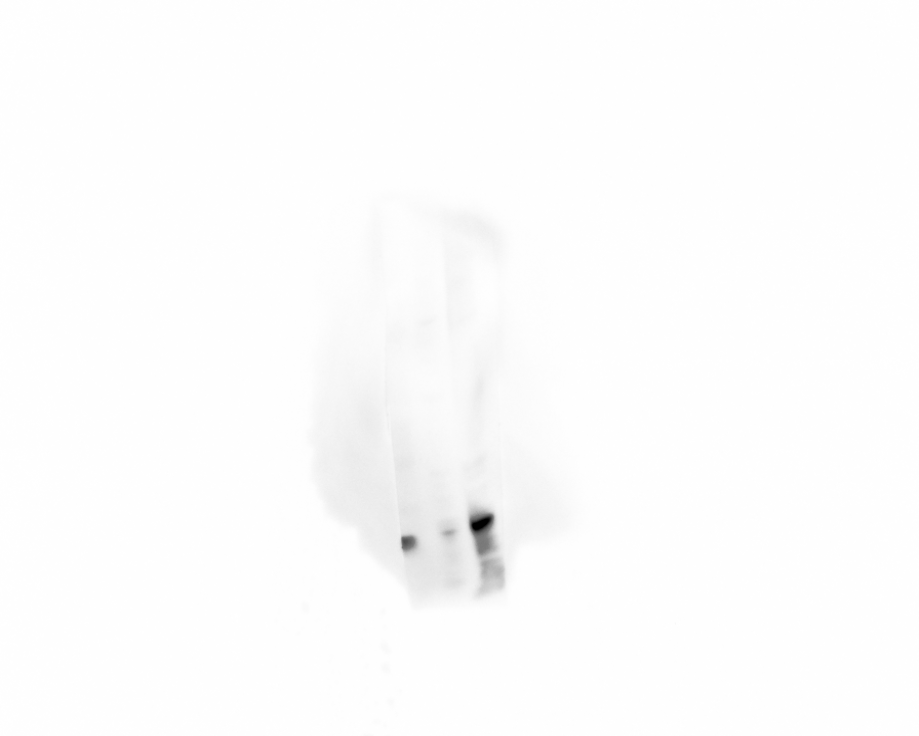

Supplement: Figure 1—source data 2. [file elife-90184-fig1-data2.zip › Raw and annotated immunoblots/Raw blots/IP spastin neuron.tif]

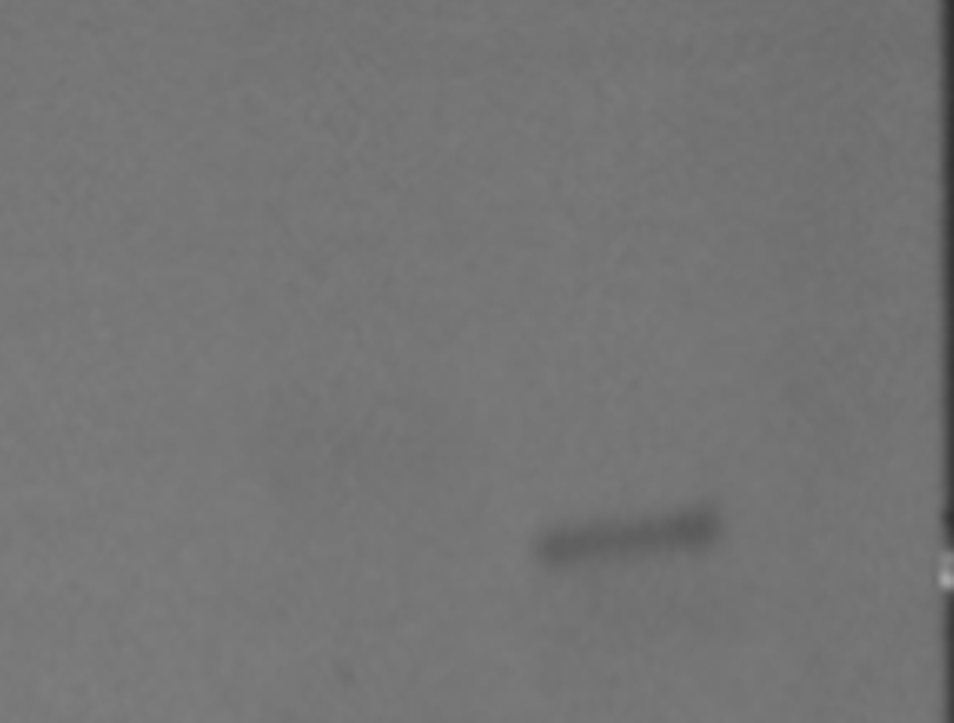

Supplement: Figure 1—source data 2. [file elife-90184-fig1-data2.zip › Raw and annotated immunoblots/Raw blots/IP spastin.tif]

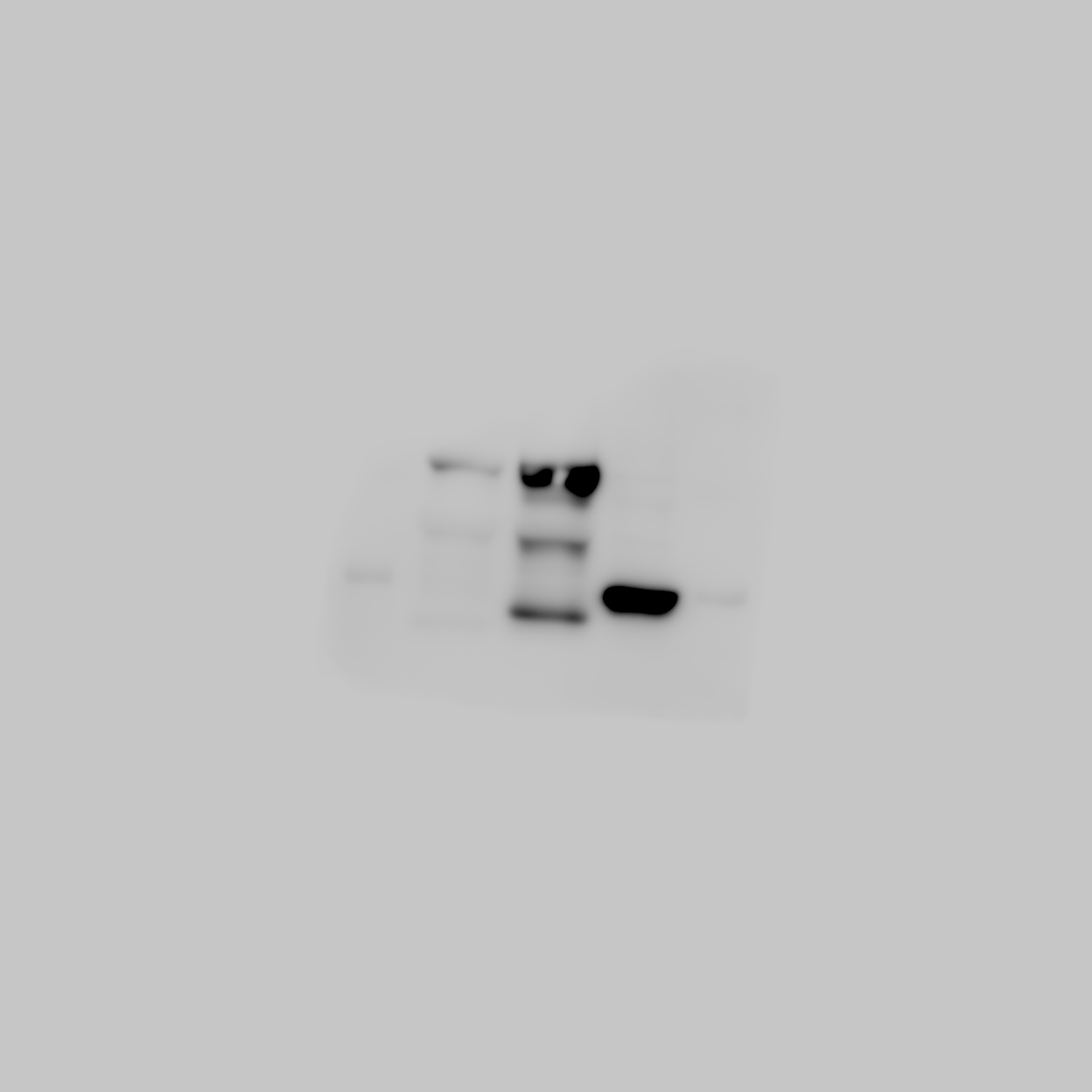

Supplement: Figure 1—source data 2. [file elife-90184-fig1-data2.zip › Raw and annotated immunoblots/Raw blots/IP WB 14-3-3.tif]

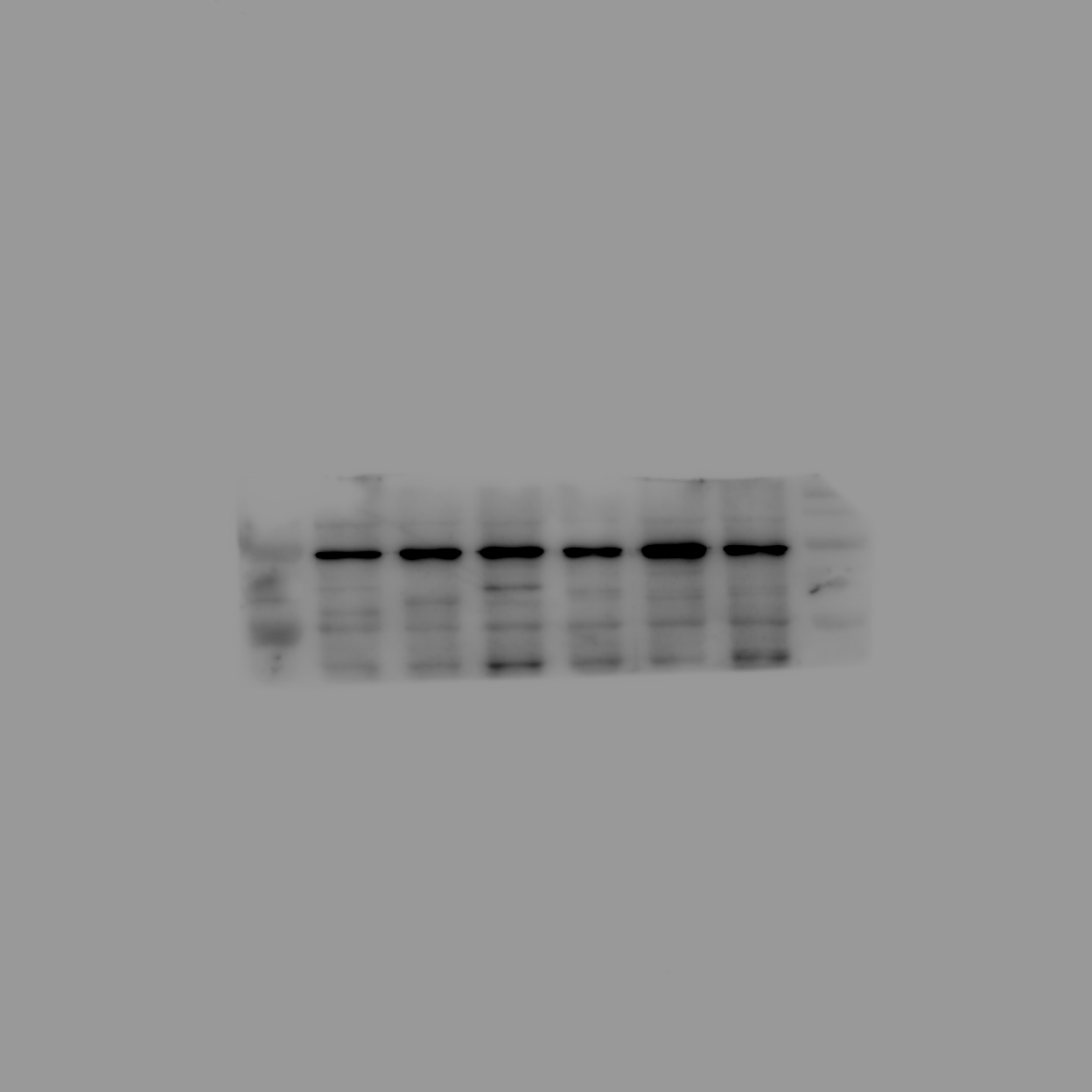

Supplement: Figure 1—source data 2. [file elife-90184-fig1-data2.zip › Raw and annotated immunoblots/Raw blots/IP.tif]

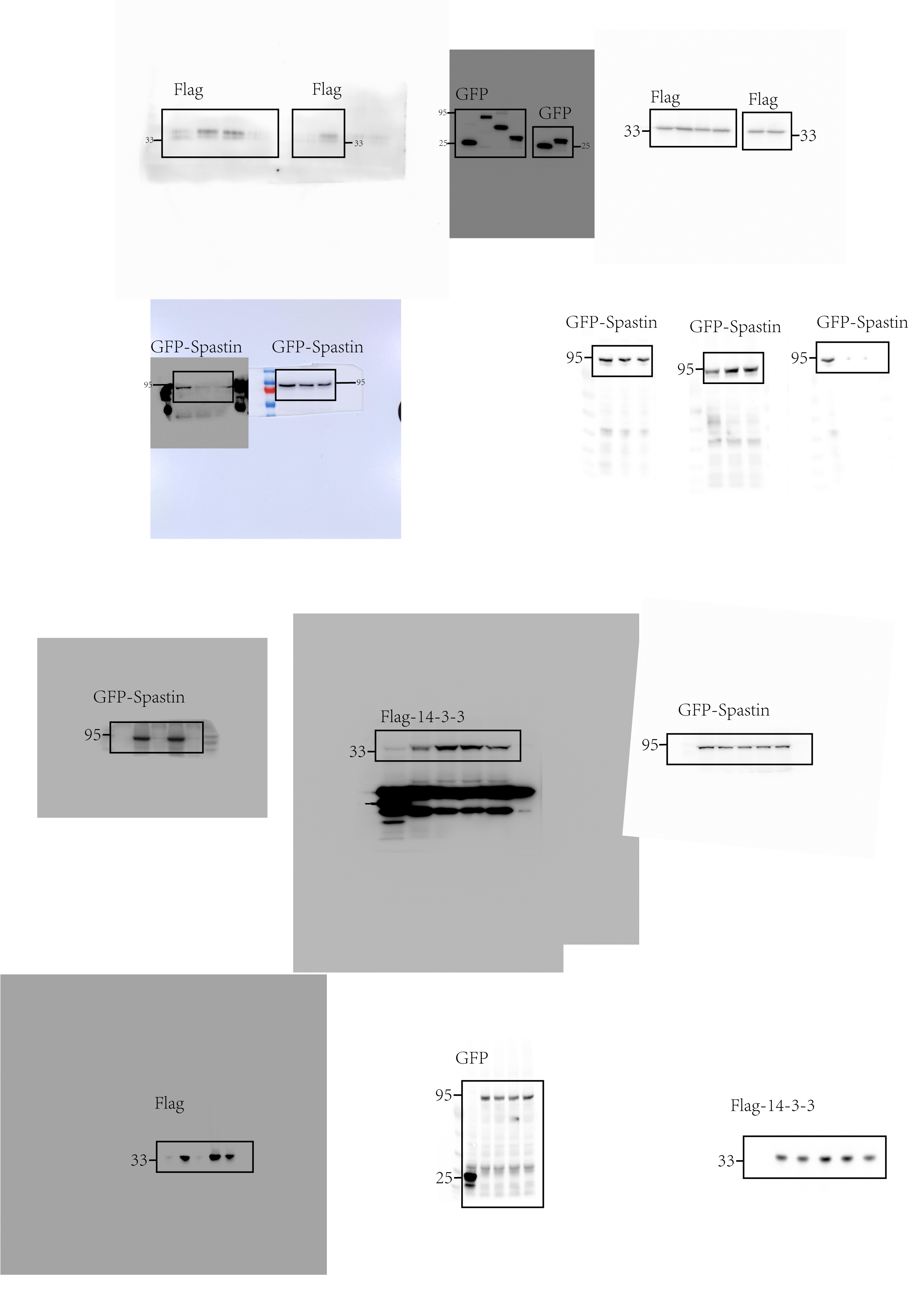

Supplement: Figure 2—source data 1. [file elife-90184-fig2-data1.zip › Figure 2-Source data 1. Raw and annotated blots for Figure 2/Annotated blots.tif]

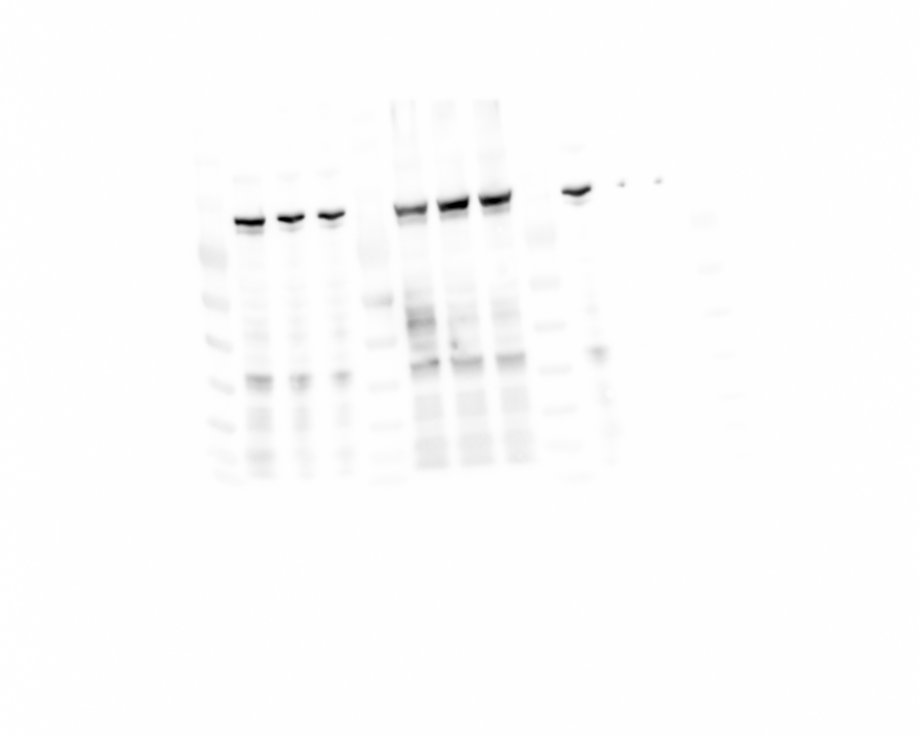

Supplement: Figure 2—source data 1. [file elife-90184-fig2-data1.zip › Figure 2-Source data 1. Raw and annotated blots for Figure 2/Raw blots/GFP-33.tif]

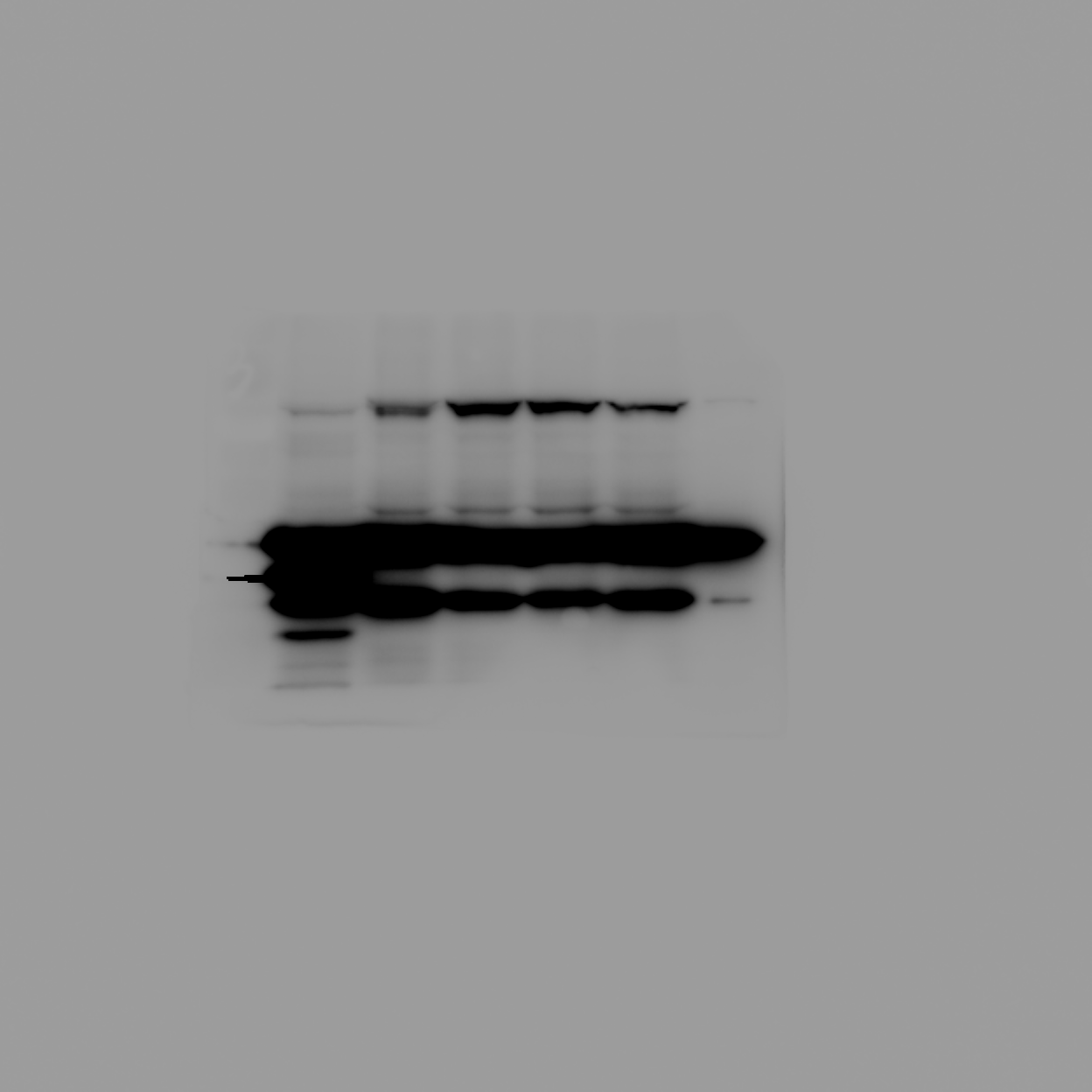

Supplement: Figure 2—source data 1. [file elife-90184-fig2-data1.zip › Figure 2-Source data 1. Raw and annotated blots for Figure 2/Raw blots/IB Flag 1433.tif]

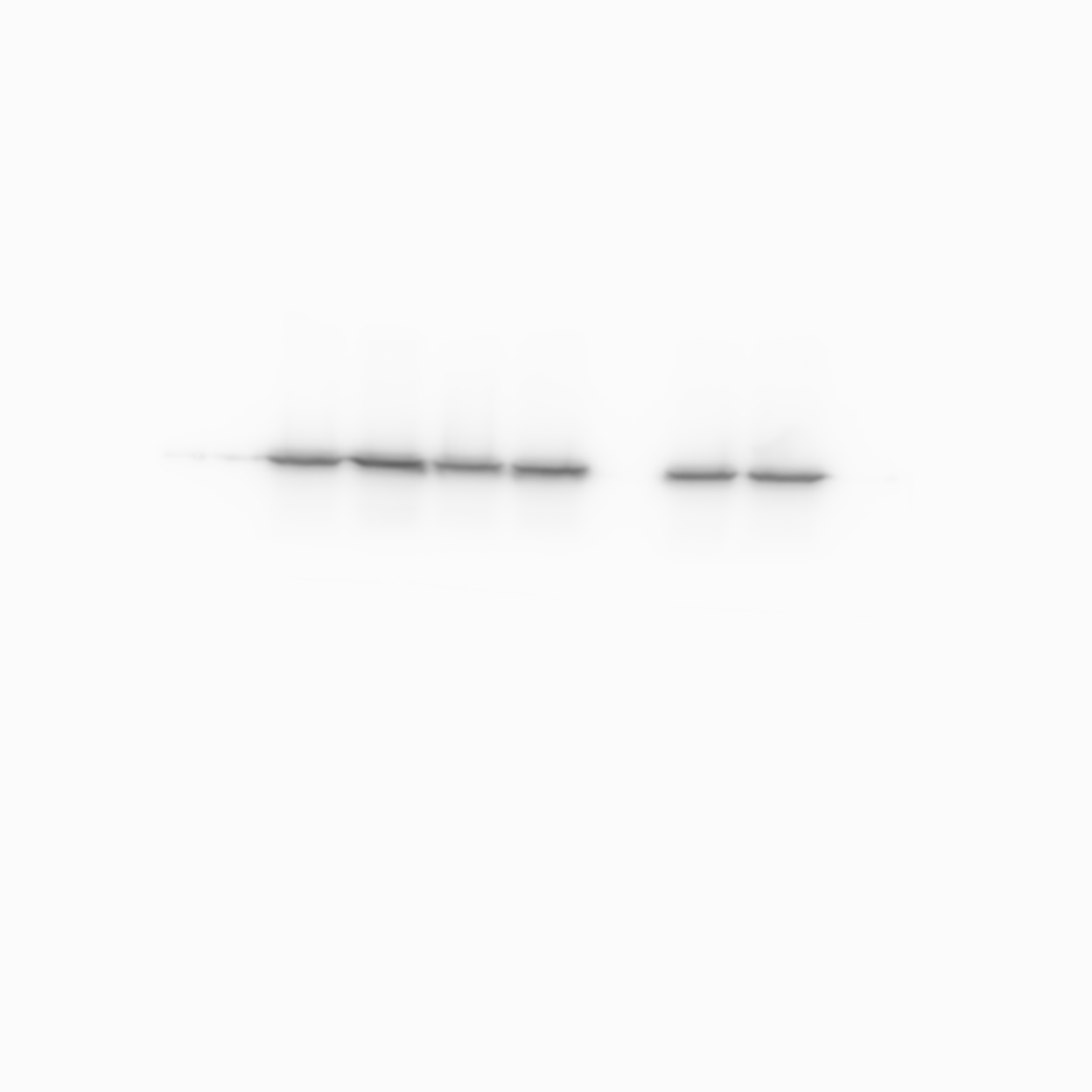

Supplement: Figure 2—source data 1. [file elife-90184-fig2-data1.zip › Figure 2-Source data 1. Raw and annotated blots for Figure 2/Raw blots/input 14-3-3.tif]

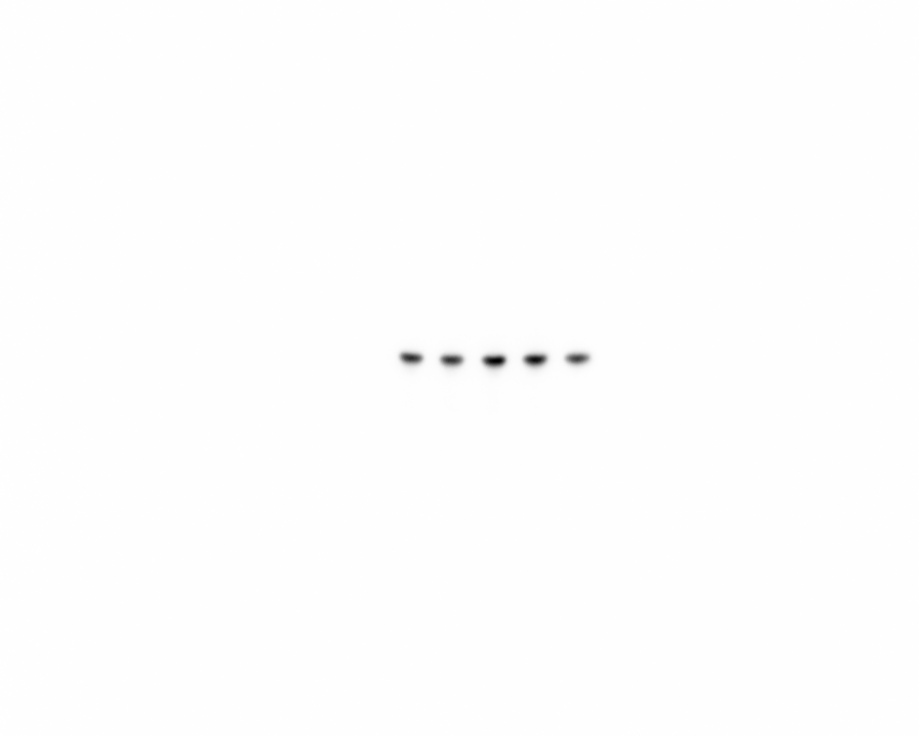

Supplement: Figure 2—source data 1. [file elife-90184-fig2-data1.zip › Figure 2-Source data 1. Raw and annotated blots for Figure 2/Raw blots/Input Flag.tif]

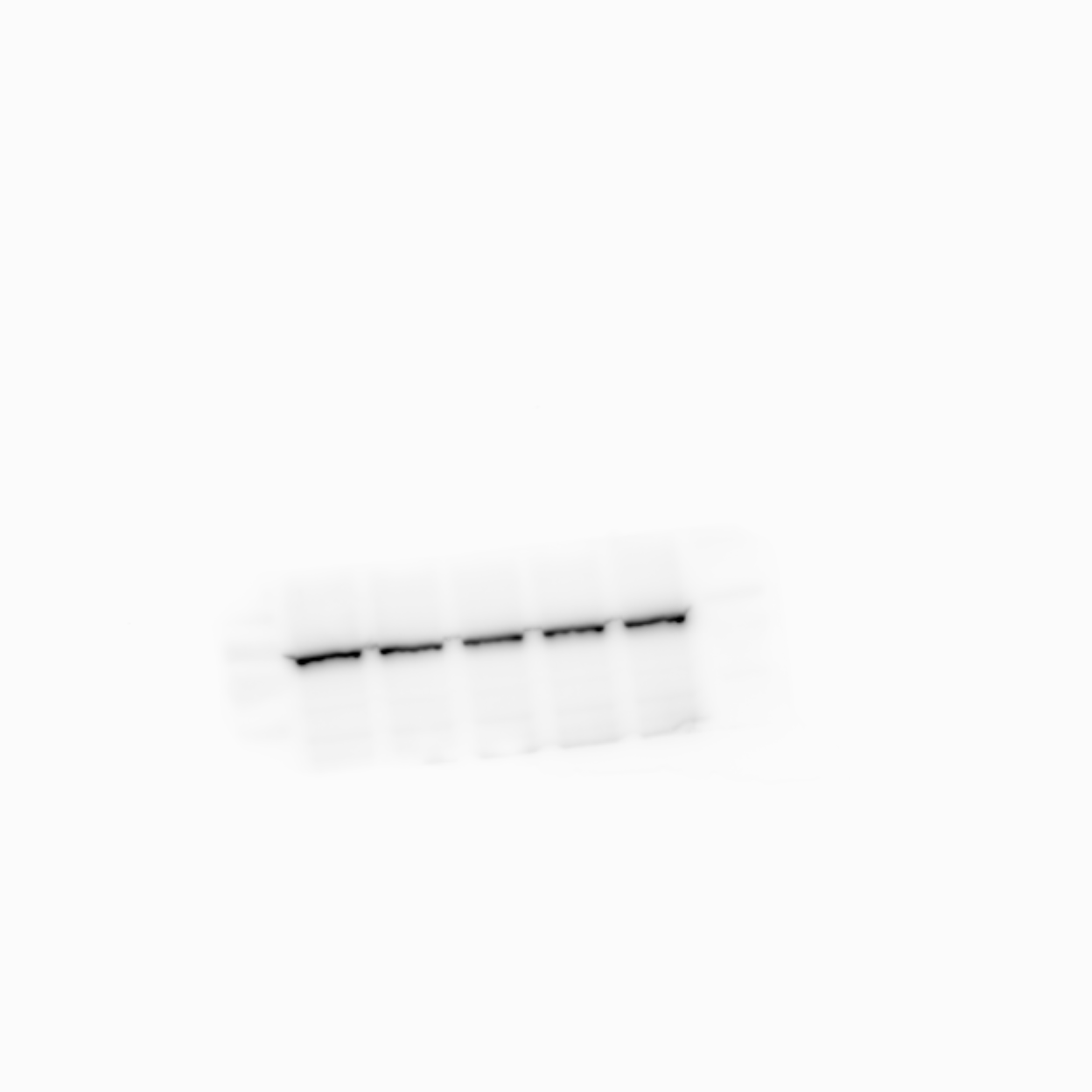

Supplement: Figure 2—source data 1. [file elife-90184-fig2-data1.zip › Figure 2-Source data 1. Raw and annotated blots for Figure 2/Raw blots/Input GFP spastin.tif]

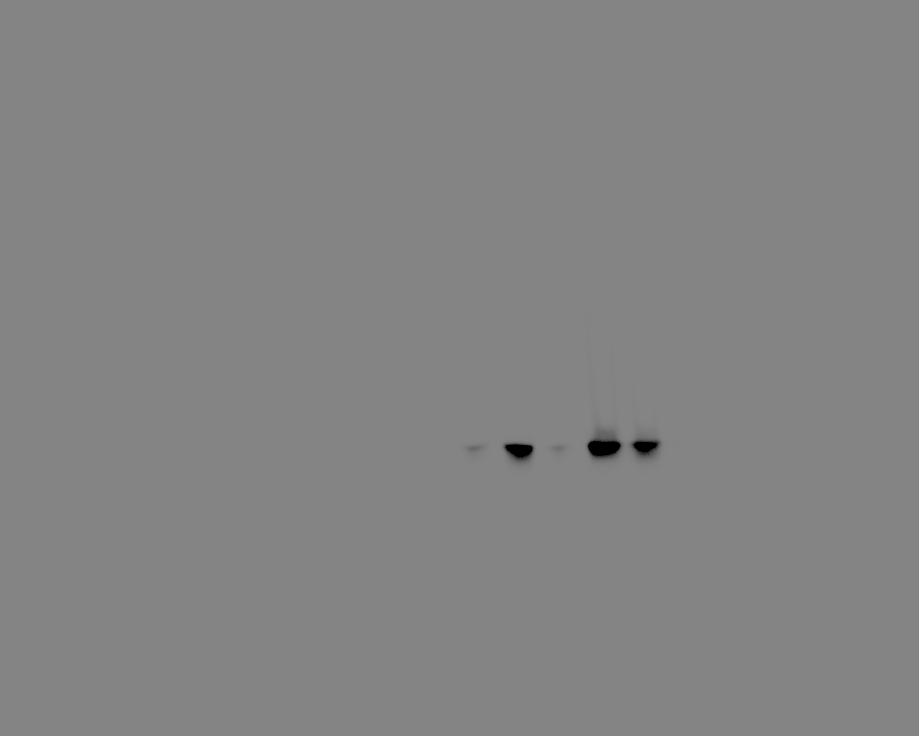

Supplement: Figure 2—source data 1. [file elife-90184-fig2-data1.zip › Figure 2-Source data 1. Raw and annotated blots for Figure 2/Raw blots/IP flag.tif]

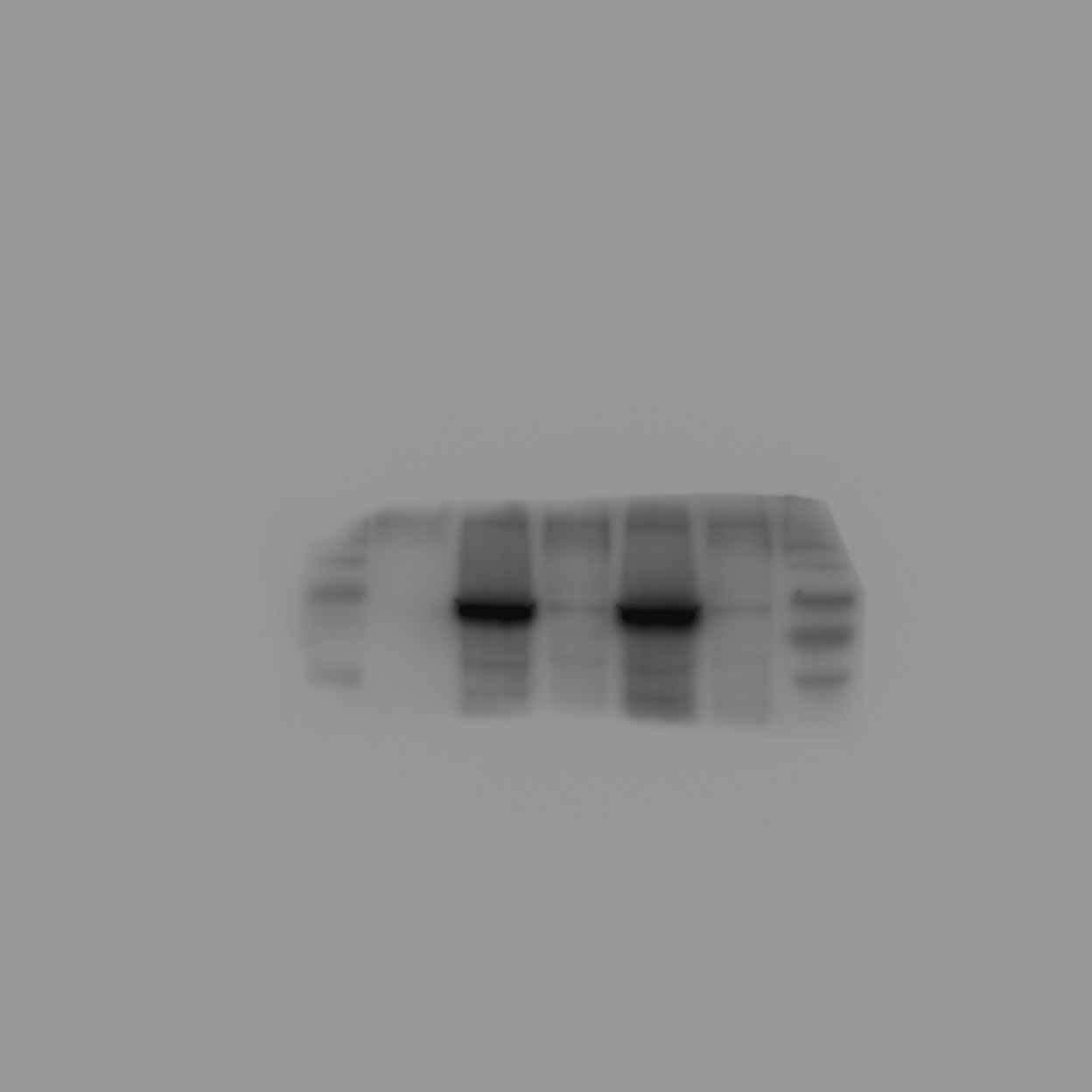

Supplement: Figure 2—source data 1. [file elife-90184-fig2-data1.zip › Figure 2-Source data 1. Raw and annotated blots for Figure 2/Raw blots/IP GFP 1.tif]

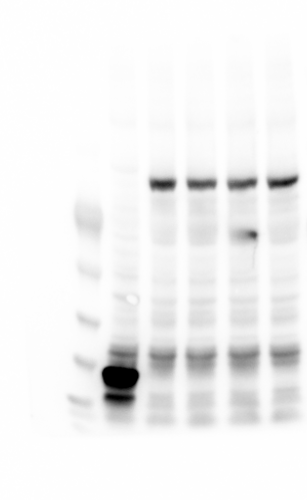

Supplement: Figure 2—source data 1. [file elife-90184-fig2-data1.zip › Figure 2-Source data 1. Raw and annotated blots for Figure 2/Raw blots/IP GFP.tif]

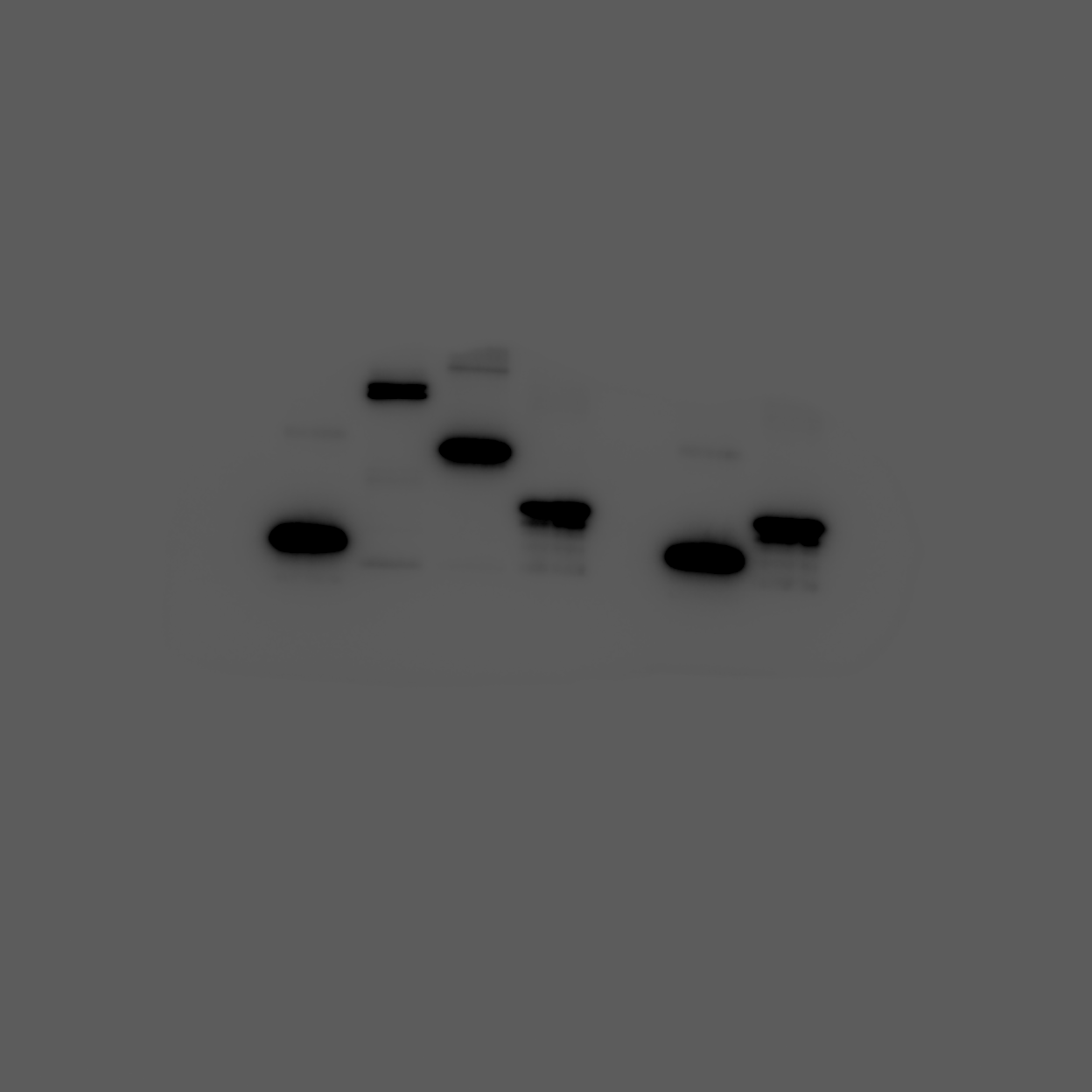

Supplement: Figure 2—source data 1. [file elife-90184-fig2-data1.zip › Figure 2-Source data 1. Raw and annotated blots for Figure 2/Raw blots/IP WB GFP.tif]

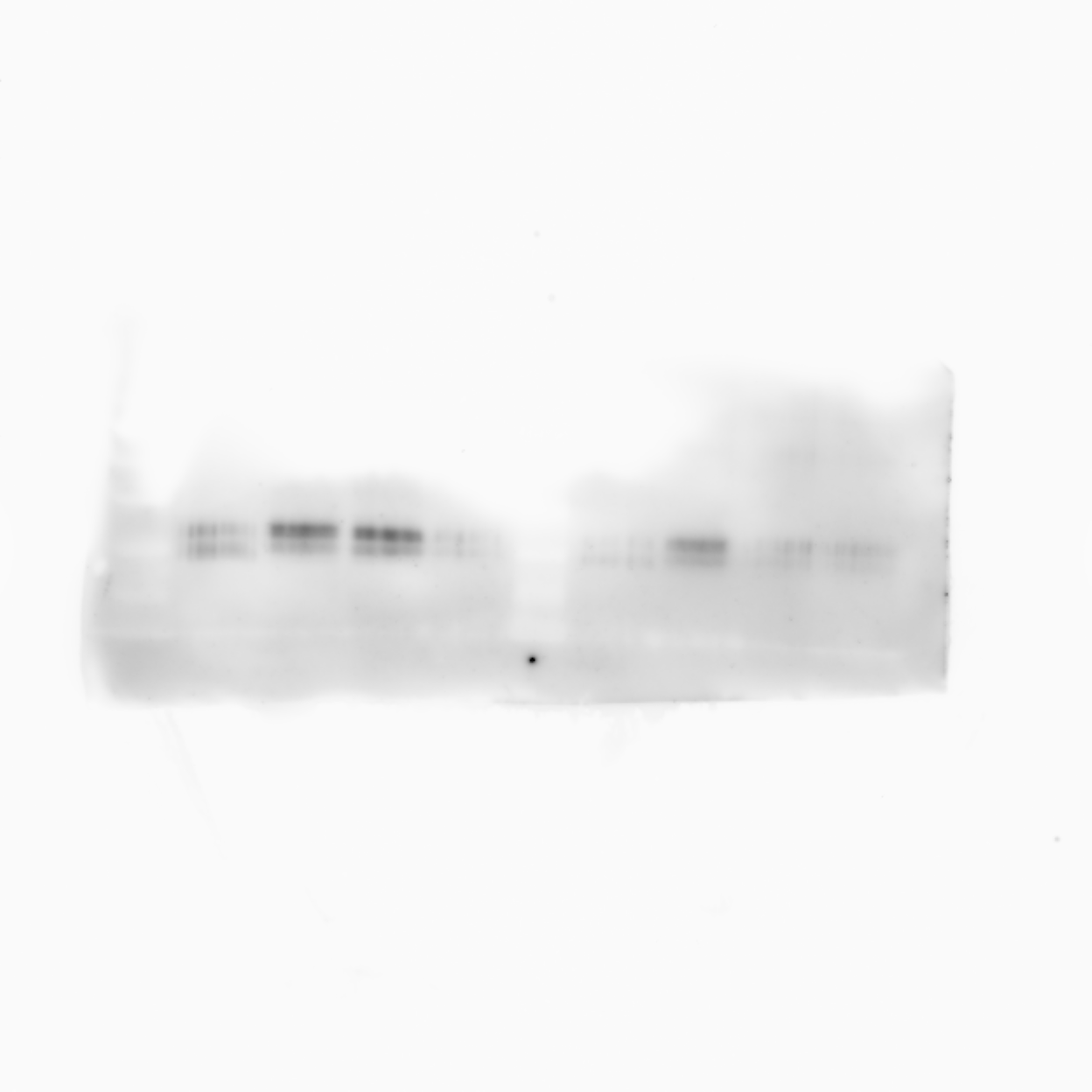

Supplement: Figure 2—source data 1. [file elife-90184-fig2-data1.zip › Figure 2-Source data 1. Raw and annotated blots for Figure 2/Raw blots/IP WBGFP.tif]

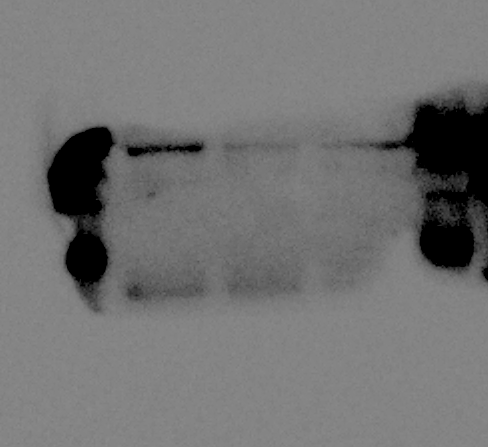

Supplement: Figure 2—source data 1. [file elife-90184-fig2-data1.zip › Figure 2-Source data 1. Raw and annotated blots for Figure 2/Raw blots/pull 20200120_002243_Ch.tif]

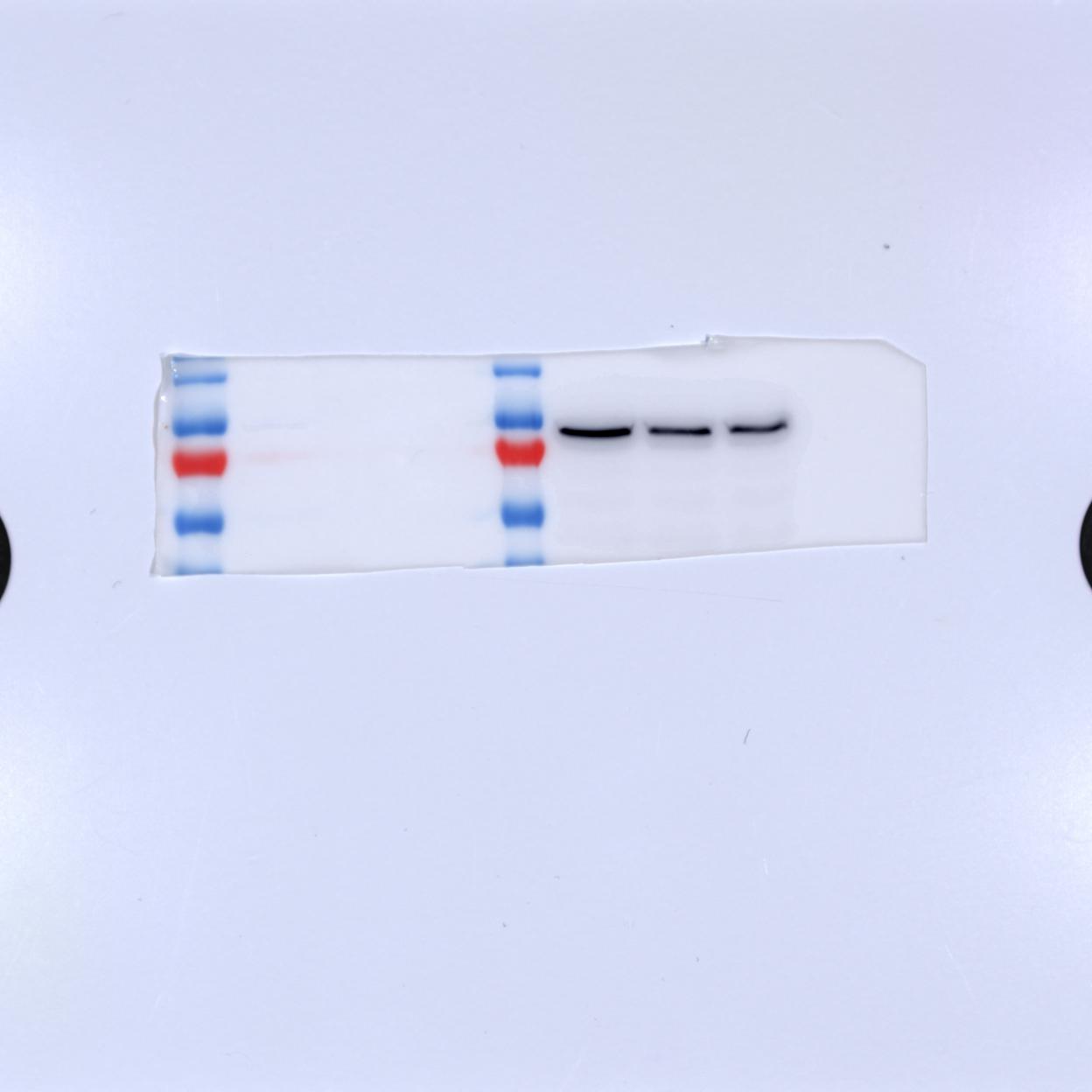

Supplement: Figure 2—source data 1. [file elife-90184-fig2-data1.zip › Figure 2-Source data 1. Raw and annotated blots for Figure 2/Raw blots/pull 20200120_002243_Ch+Marker.jpg]

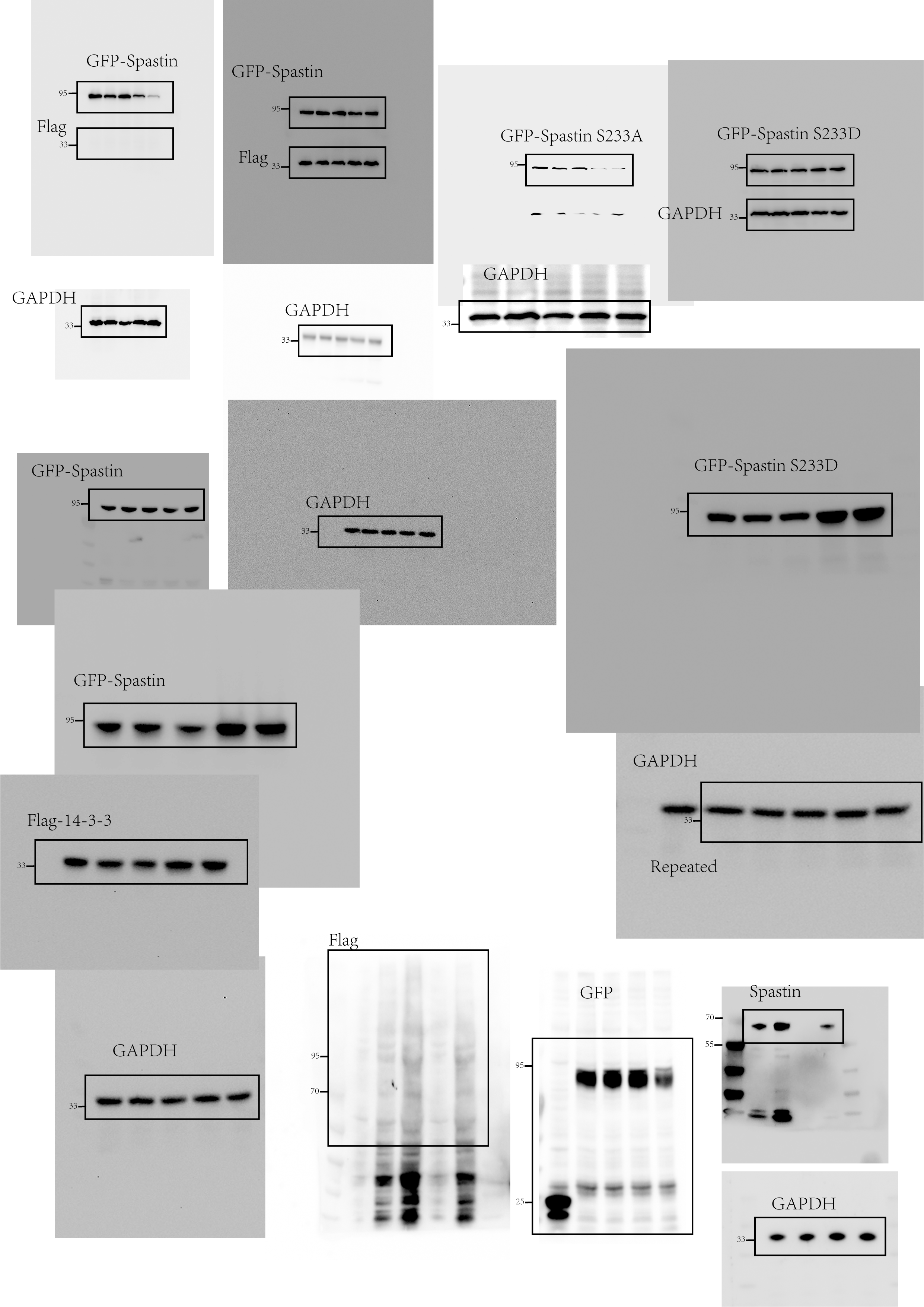

Supplement: Figure 3—source data 1. [file elife-90184-fig3-data1.zip › Figure 3-Source data 1. Raw and annotated blots/Annotated blots.tif]

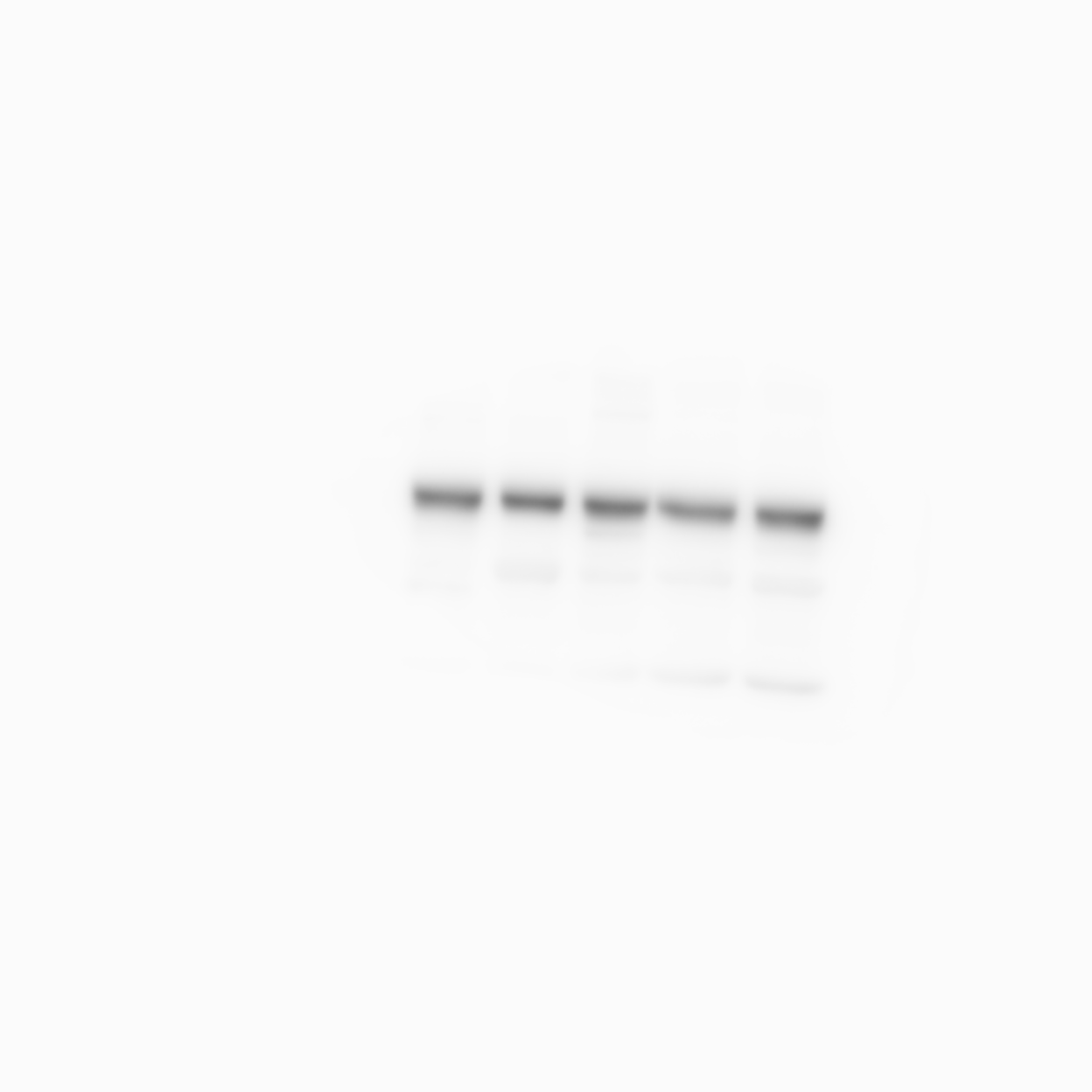

Supplement: Figure 3—source data 1. [file elife-90184-fig3-data1.zip › Figure 3-Source data 1. Raw and annotated blots/Raw blots/14-3-3 spastin GAPDH.tif]

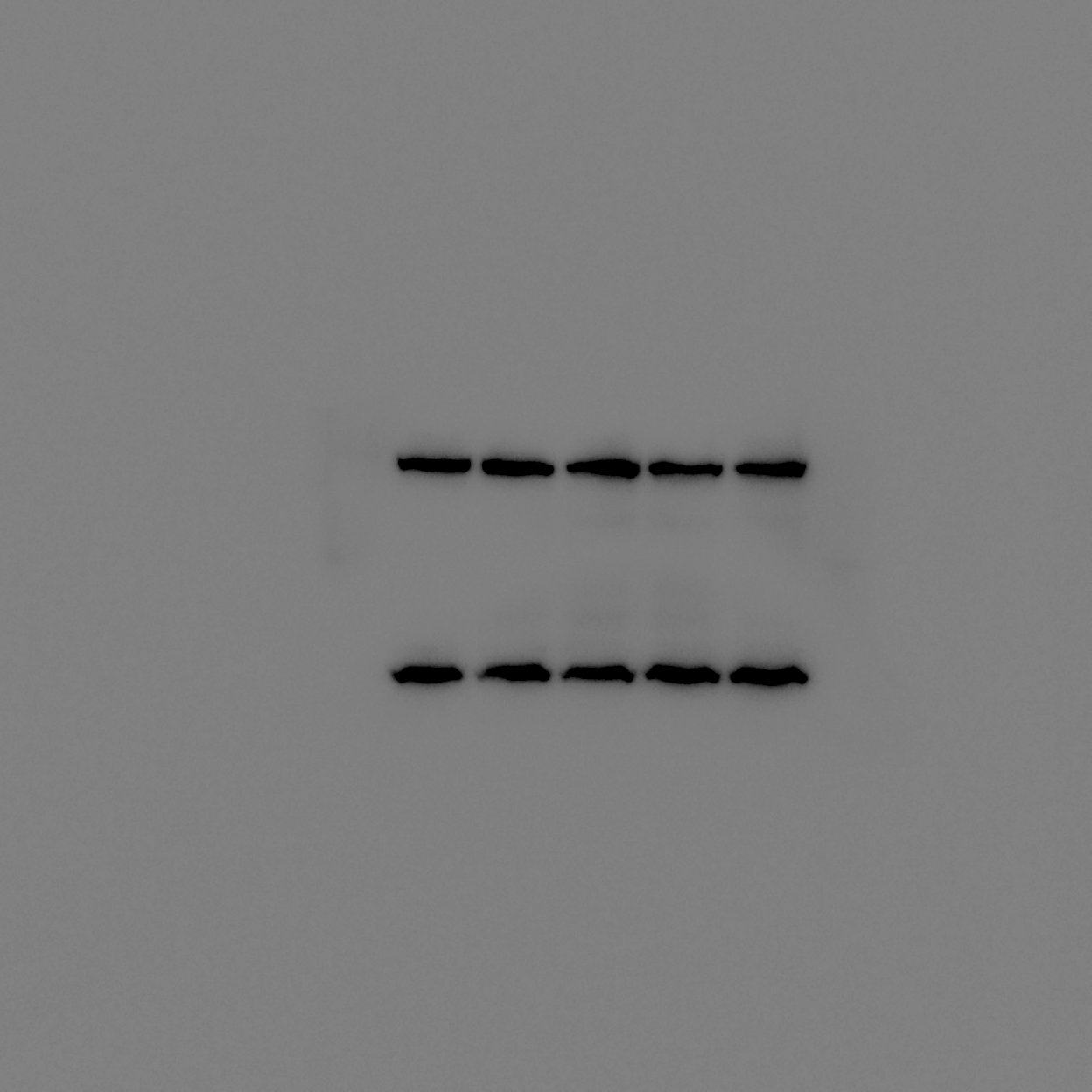

Supplement: Figure 3—source data 1. [file elife-90184-fig3-data1.zip › Figure 3-Source data 1. Raw and annotated blots/Raw blots/14-3-3 spastin GFP Flag.tif]

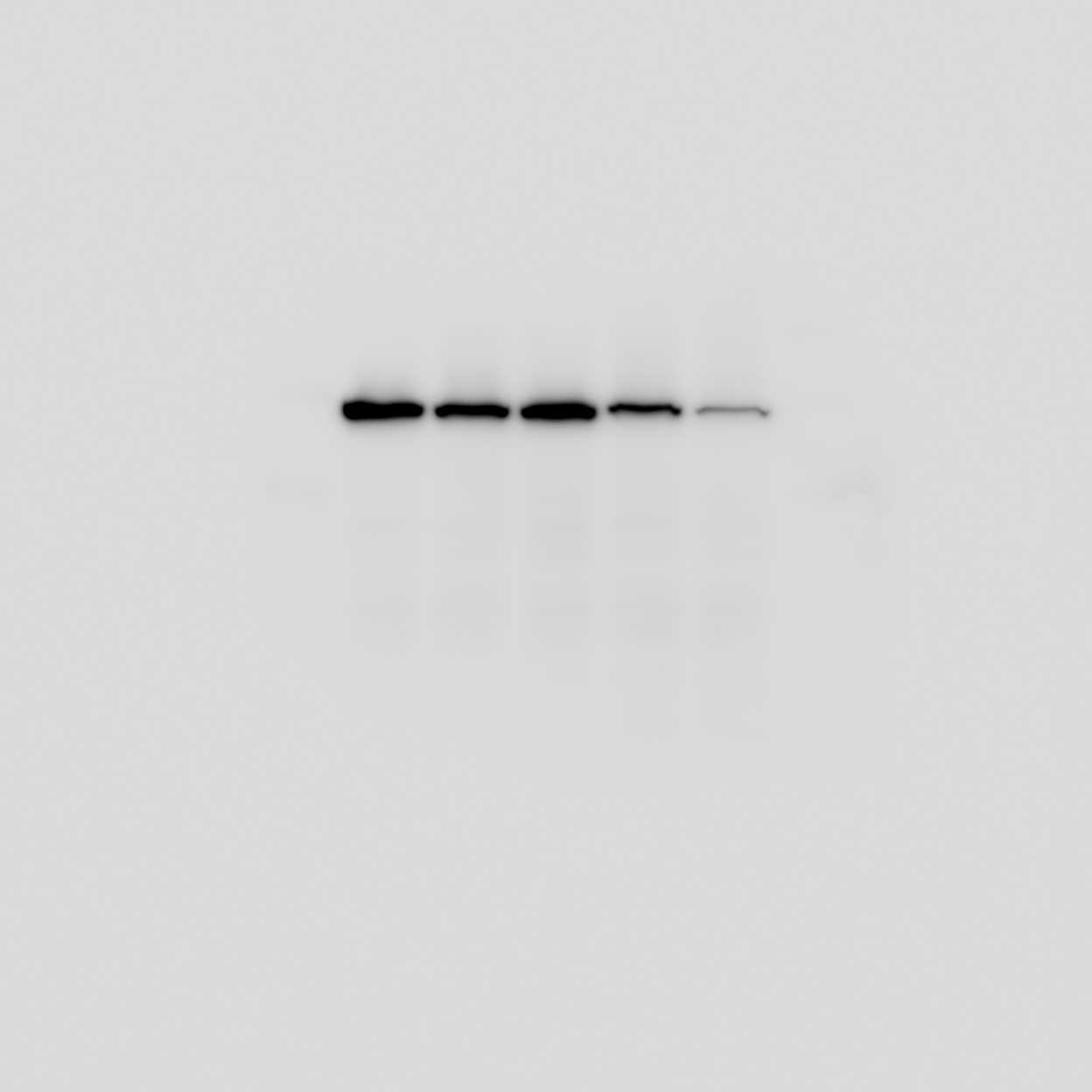

Supplement: Figure 3—source data 1. [file elife-90184-fig3-data1.zip › Figure 3-Source data 1. Raw and annotated blots/Raw blots/20200805_114924_Ch.tif]

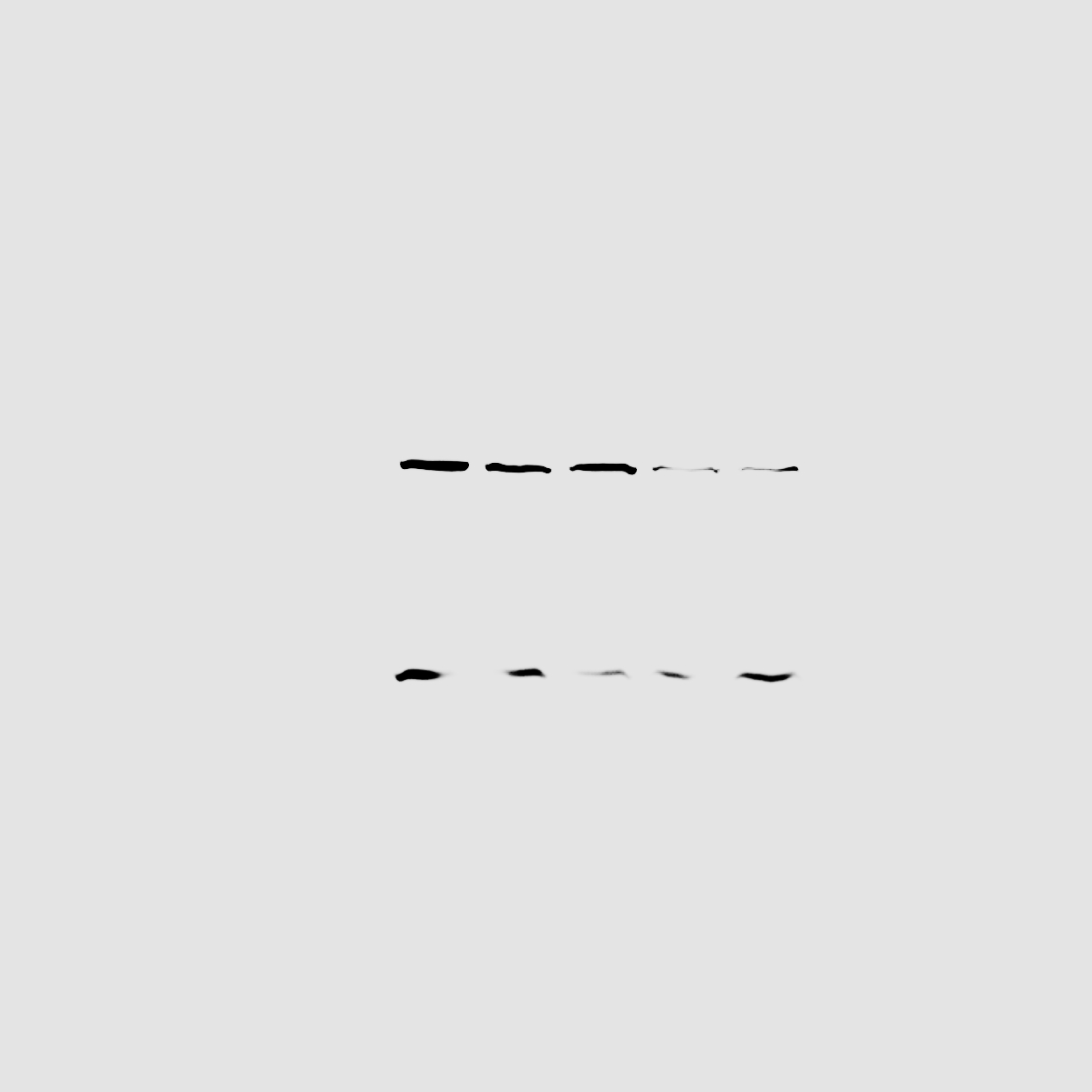

Supplement: Figure 3—source data 1. [file elife-90184-fig3-data1.zip › Figure 3-Source data 1. Raw and annotated blots/Raw blots/233a.tif]

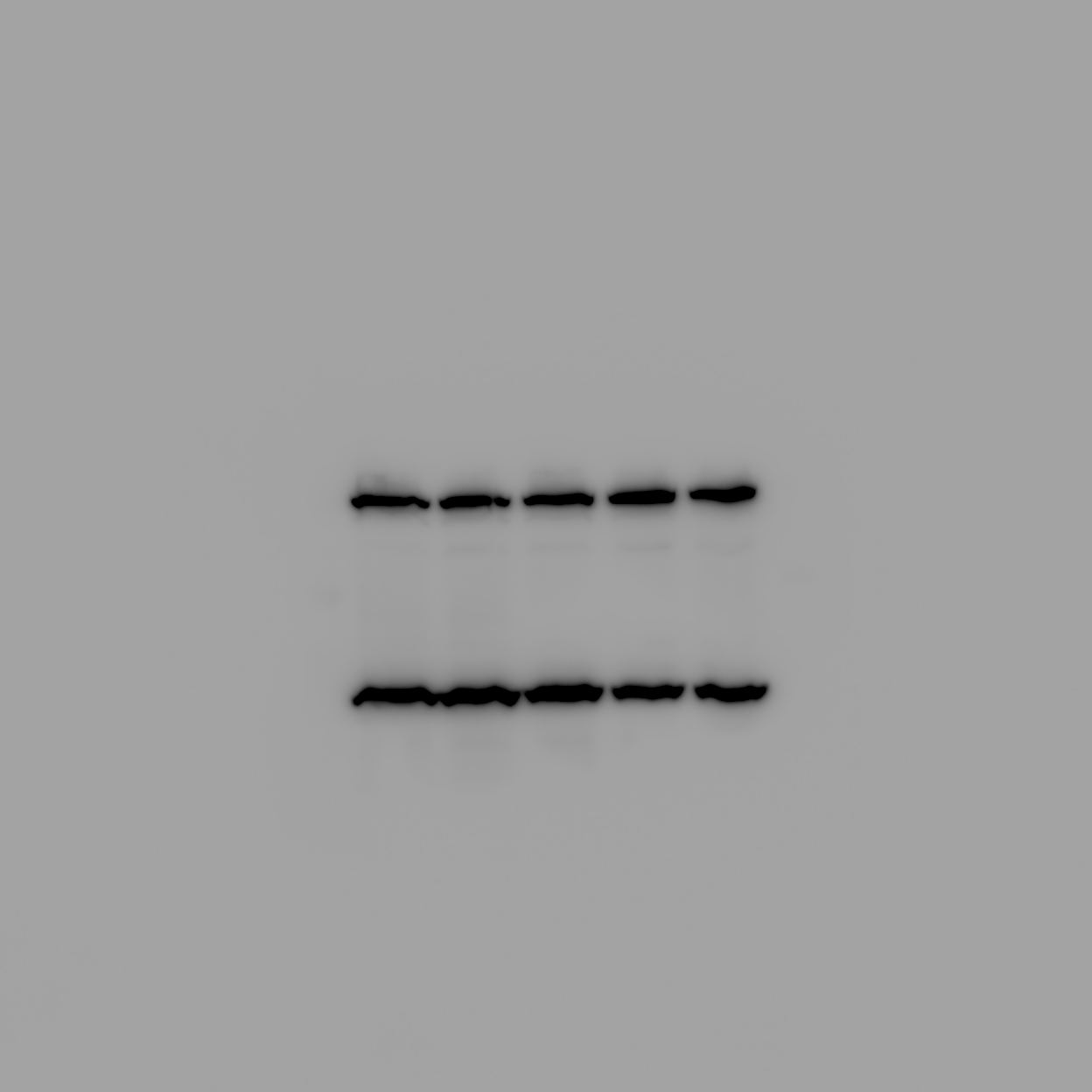

Supplement: Figure 3—source data 1. [file elife-90184-fig3-data1.zip › Figure 3-Source data 1. Raw and annotated blots/Raw blots/233d╝░GAPDH.tif]

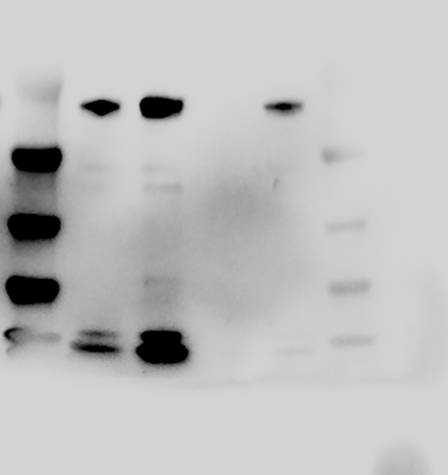

Supplement: Figure 3—source data 1. [file elife-90184-fig3-data1.zip › Figure 3-Source data 1. Raw and annotated blots/Raw blots/CHX MG132 spastin╡░░╫╦«╞╜.tif]

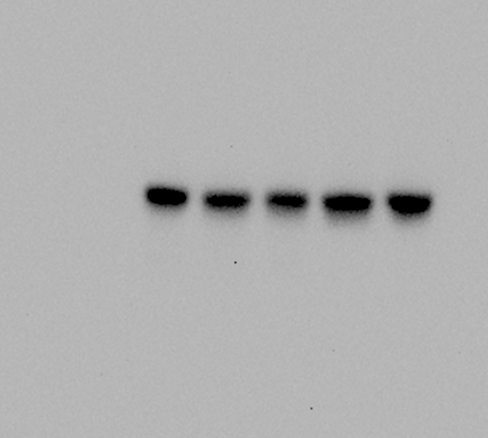

Supplement: Figure 3—source data 1. [file elife-90184-fig3-data1.zip › Figure 3-Source data 1. Raw and annotated blots/Raw blots/Flag 14-3-3╘÷╝╙spastin Flag.tif]

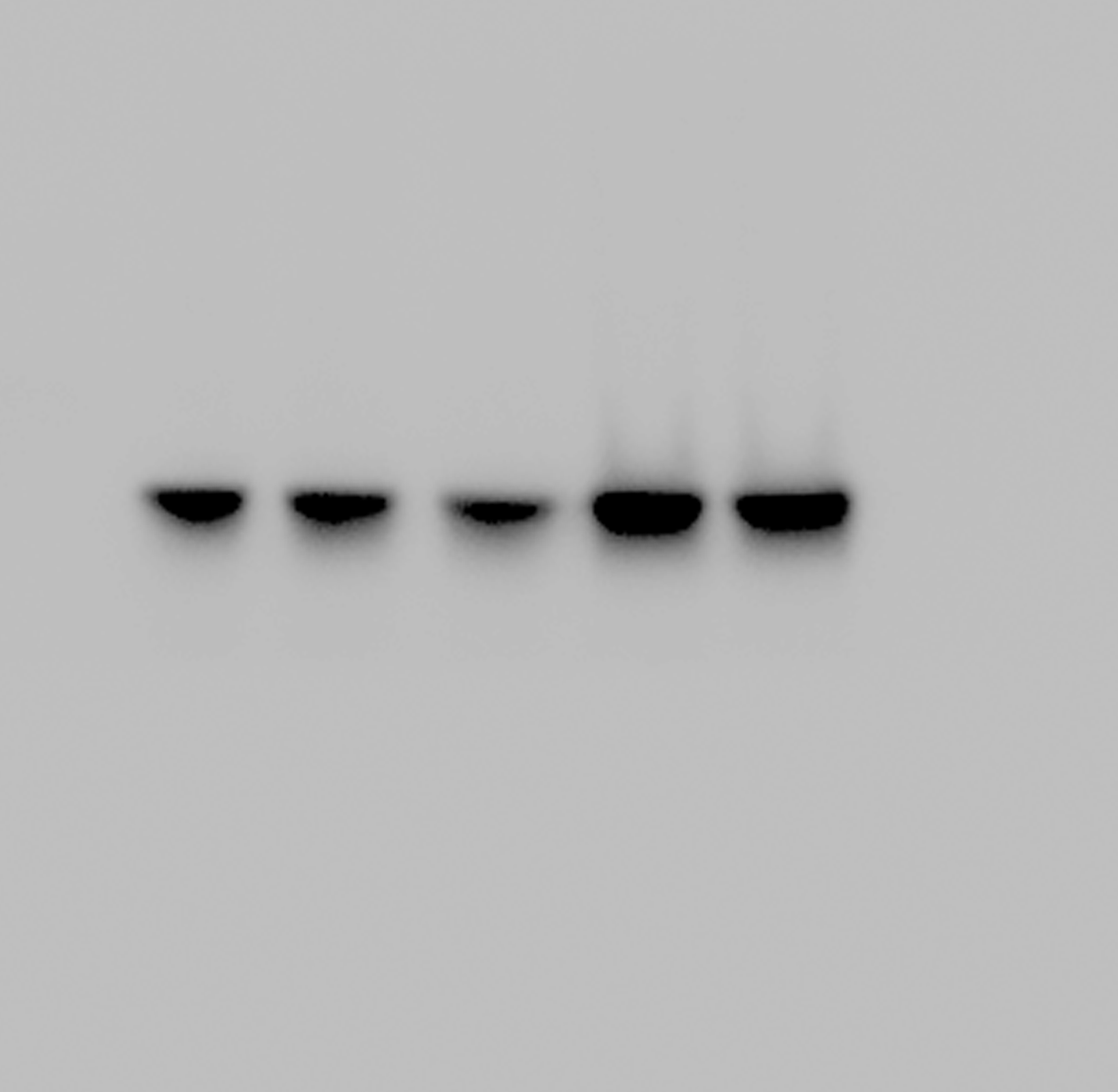

Supplement: Figure 3—source data 1. [file elife-90184-fig3-data1.zip › Figure 3-Source data 1. Raw and annotated blots/Raw blots/Flag 14-3-3╘÷╝╙spastin╦«╞╜ spastin.tif]

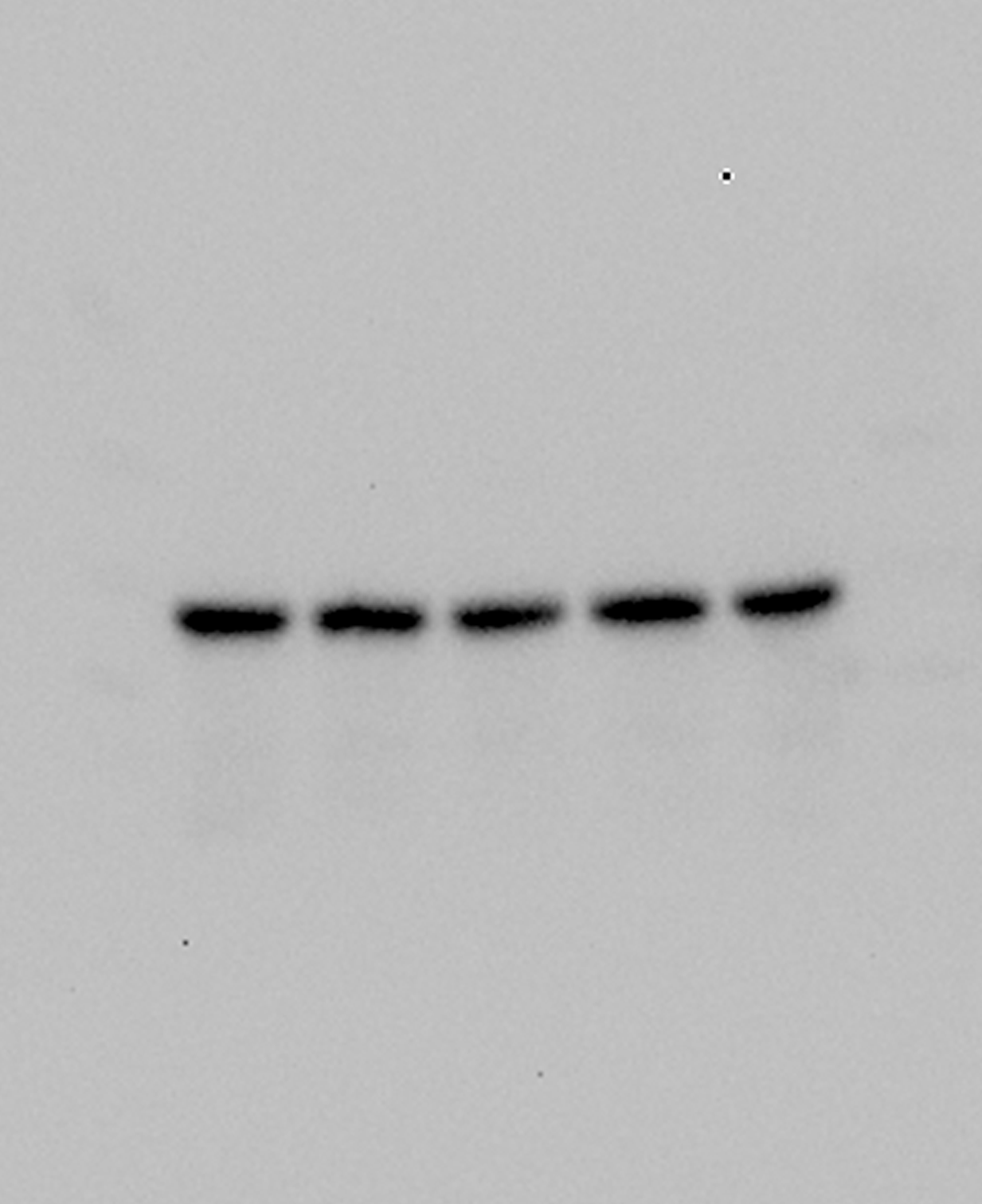

Supplement: Figure 3—source data 1. [file elife-90184-fig3-data1.zip › Figure 3-Source data 1. Raw and annotated blots/Raw blots/Flag╘÷╝╙spastin╦«╞╜ GAPDH.tif]

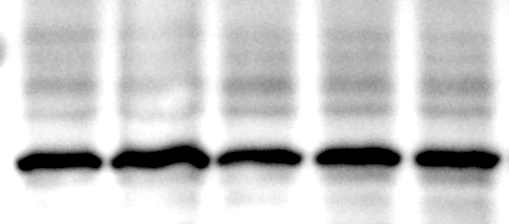

Supplement: Figure 3—source data 1. [file elife-90184-fig3-data1.zip › Figure 3-Source data 1. Raw and annotated blots/Raw blots/GAPDH re-blot.tif]

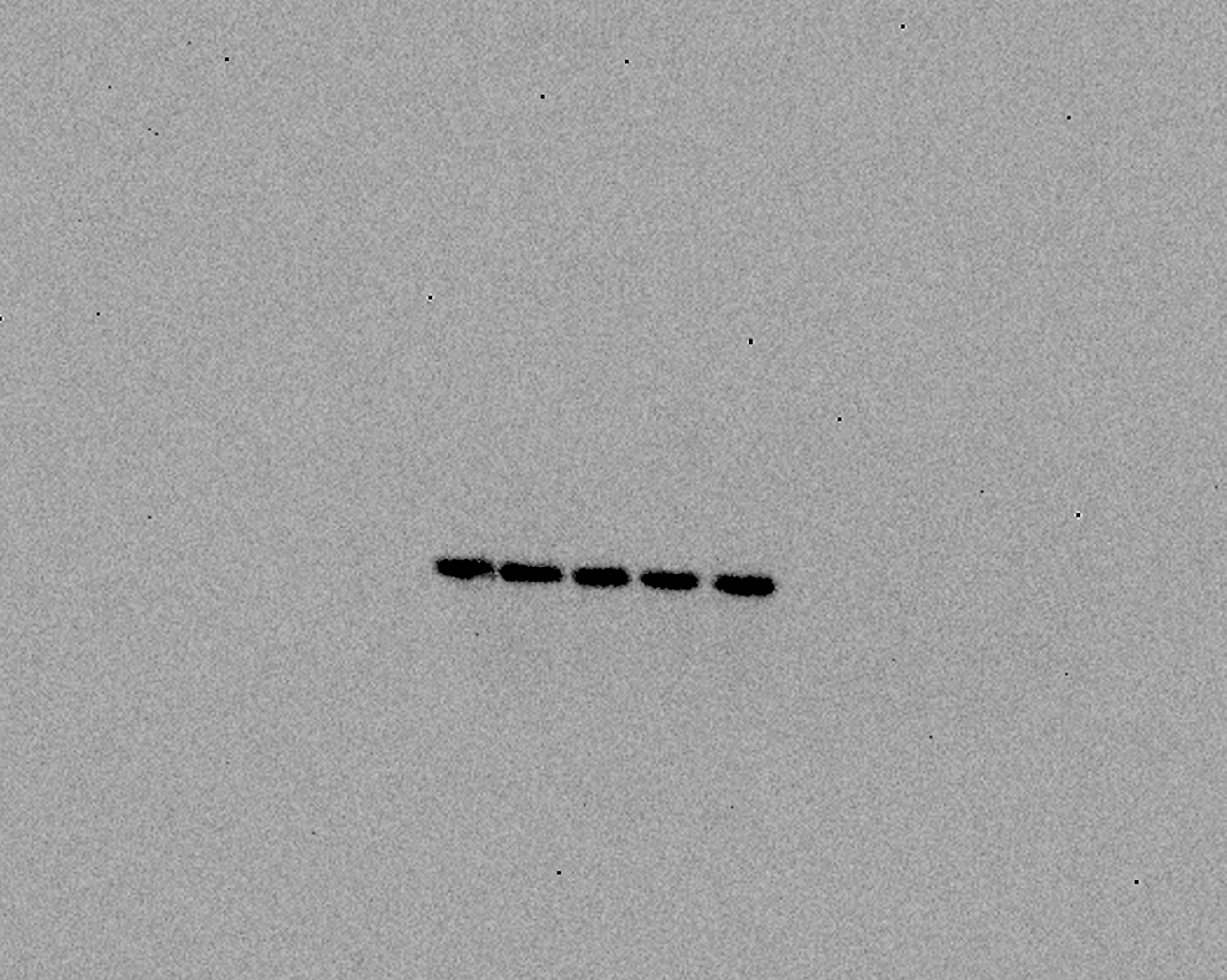

Supplement: Figure 3—source data 1. [file elife-90184-fig3-data1.zip › Figure 3-Source data 1. Raw and annotated blots/Raw blots/gapdh spastin.tif]

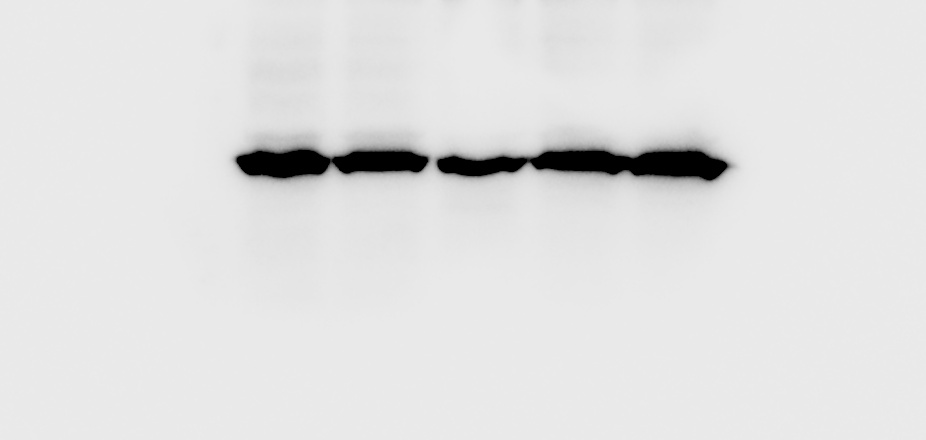

Supplement: Figure 3—source data 1. [file elife-90184-fig3-data1.zip › Figure 3-Source data 1. Raw and annotated blots/Raw blots/GAPDH.tif]

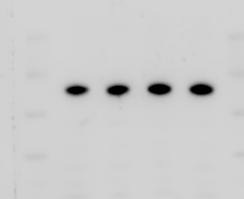

Supplement: Figure 3—source data 1. [file elife-90184-fig3-data1.zip › Figure 3-Source data 1. Raw and annotated blots/Raw blots/GAPDH-33.tif]

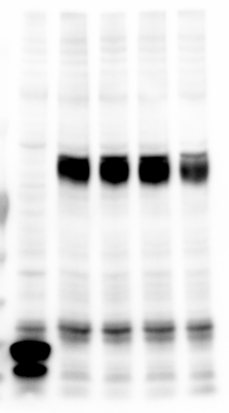

Supplement: Figure 3—source data 1. [file elife-90184-fig3-data1.zip › Figure 3-Source data 1. Raw and annotated blots/Raw blots/IP GFP.tif]

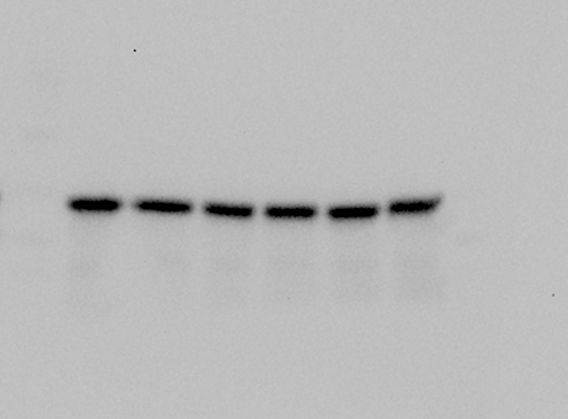

Supplement: Figure 3—source data 1. [file elife-90184-fig3-data1.zip › Figure 3-Source data 1. Raw and annotated blots/Raw blots/S233D GAPDH control.tif]

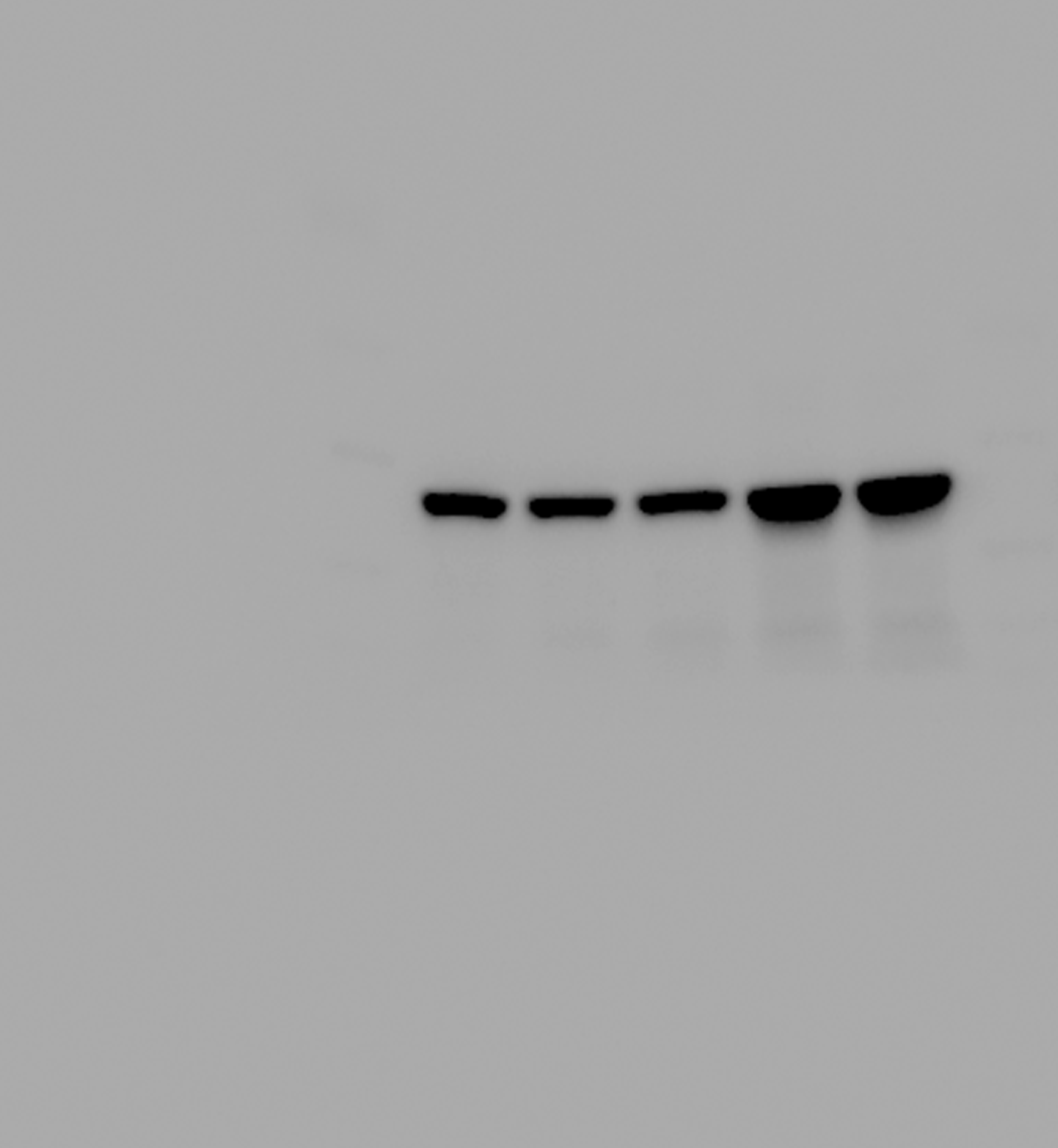

Supplement: Figure 3—source data 1. [file elife-90184-fig3-data1.zip › Figure 3-Source data 1. Raw and annotated blots/Raw blots/S233D spastin.tif]

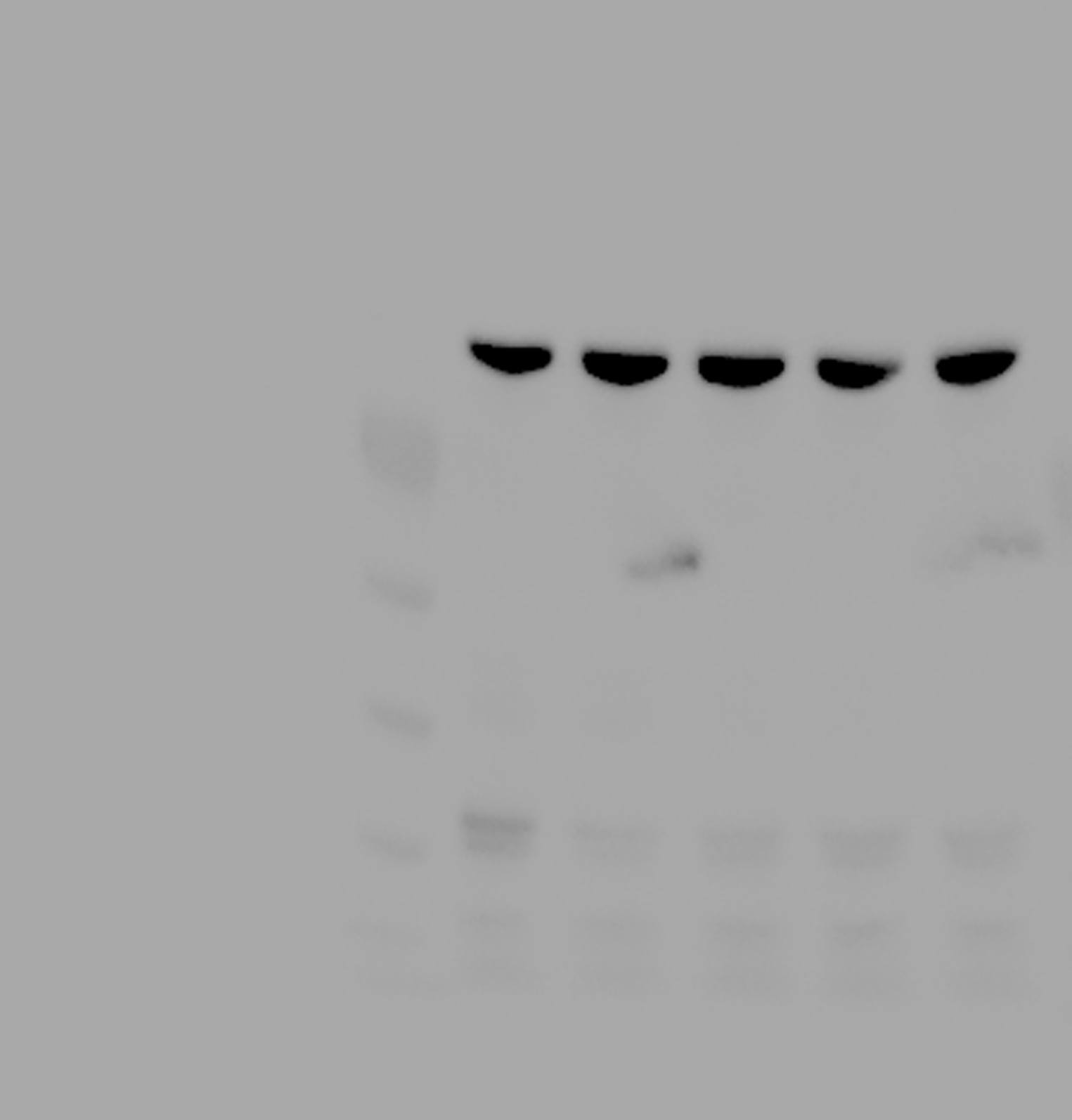

Supplement: Figure 3—source data 1. [file elife-90184-fig3-data1.zip › Figure 3-Source data 1. Raw and annotated blots/Raw blots/spastin ╬┤┤a└φ.tif]

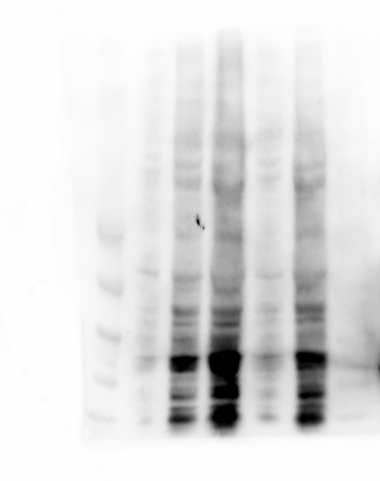

Supplement: Figure 3—source data 1. [file elife-90184-fig3-data1.zip › Figure 3-Source data 1. Raw and annotated blots/Raw blots/╖║╦╪╗»╠⌡┤°.tif]

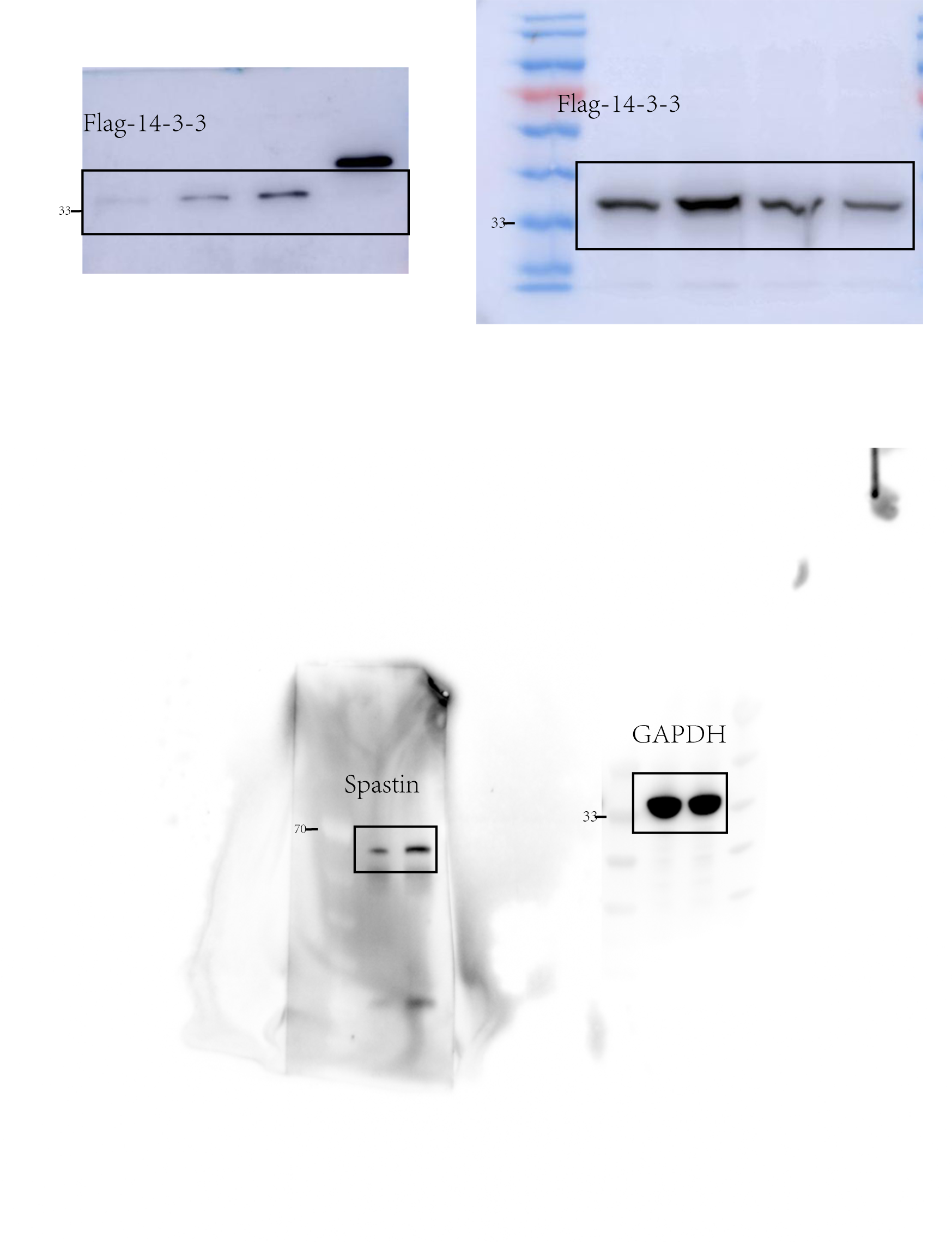

Supplement: Figure 4—source data 1. [file elife-90184-fig4-data1.zip › Figure 4-Source data 1. Raw and annotated blots/Annotated blots.tif]

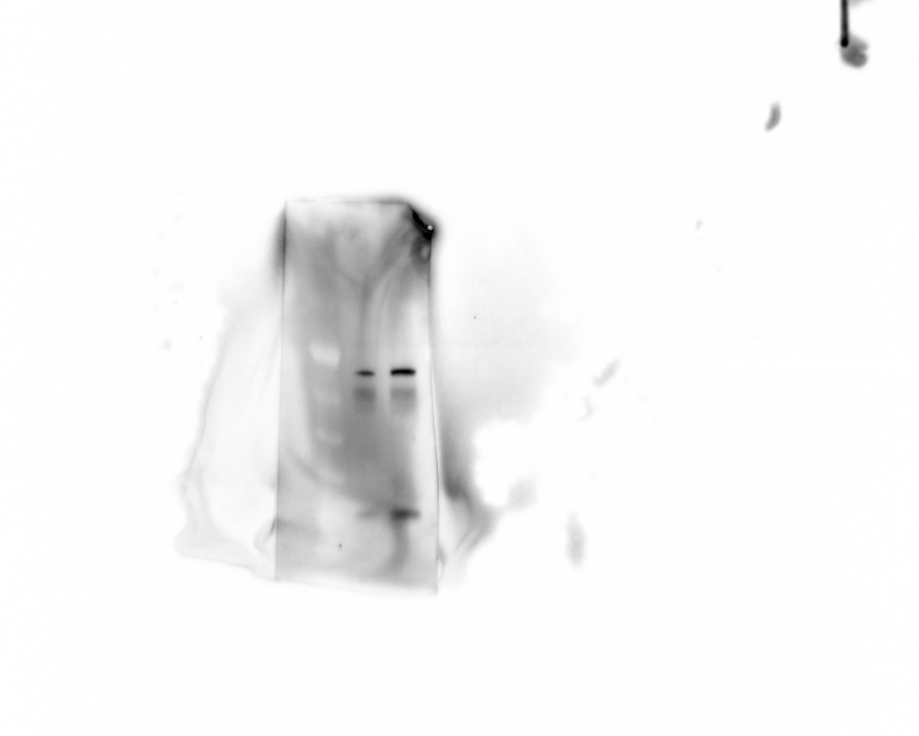

Supplement: Figure 4—source data 1. [file elife-90184-fig4-data1.zip › Figure 4-Source data 1. Raw and annotated blots/Raw blots/11.tif]

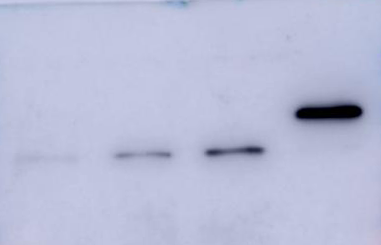

Supplement: Figure 4—source data 1. [file elife-90184-fig4-data1.zip › Figure 4-Source data 1. Raw and annotated blots/Raw blots/20201210_004015_Ch+Marker - ╕▒▒╛.tif]

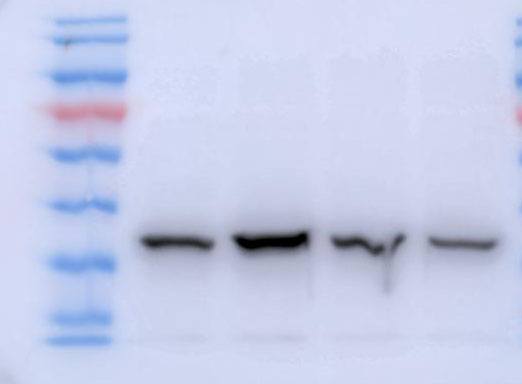

Supplement: Figure 4—source data 1. [file elife-90184-fig4-data1.zip › Figure 4-Source data 1. Raw and annotated blots/Raw blots/Flag-1433.jpg]

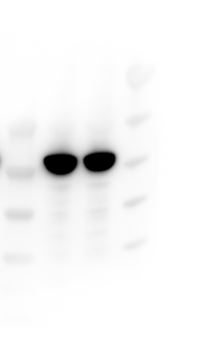

Supplement: Figure 4—source data 1. [file elife-90184-fig4-data1.zip › Figure 4-Source data 1. Raw and annotated blots/Raw blots/GAPDH.tif]

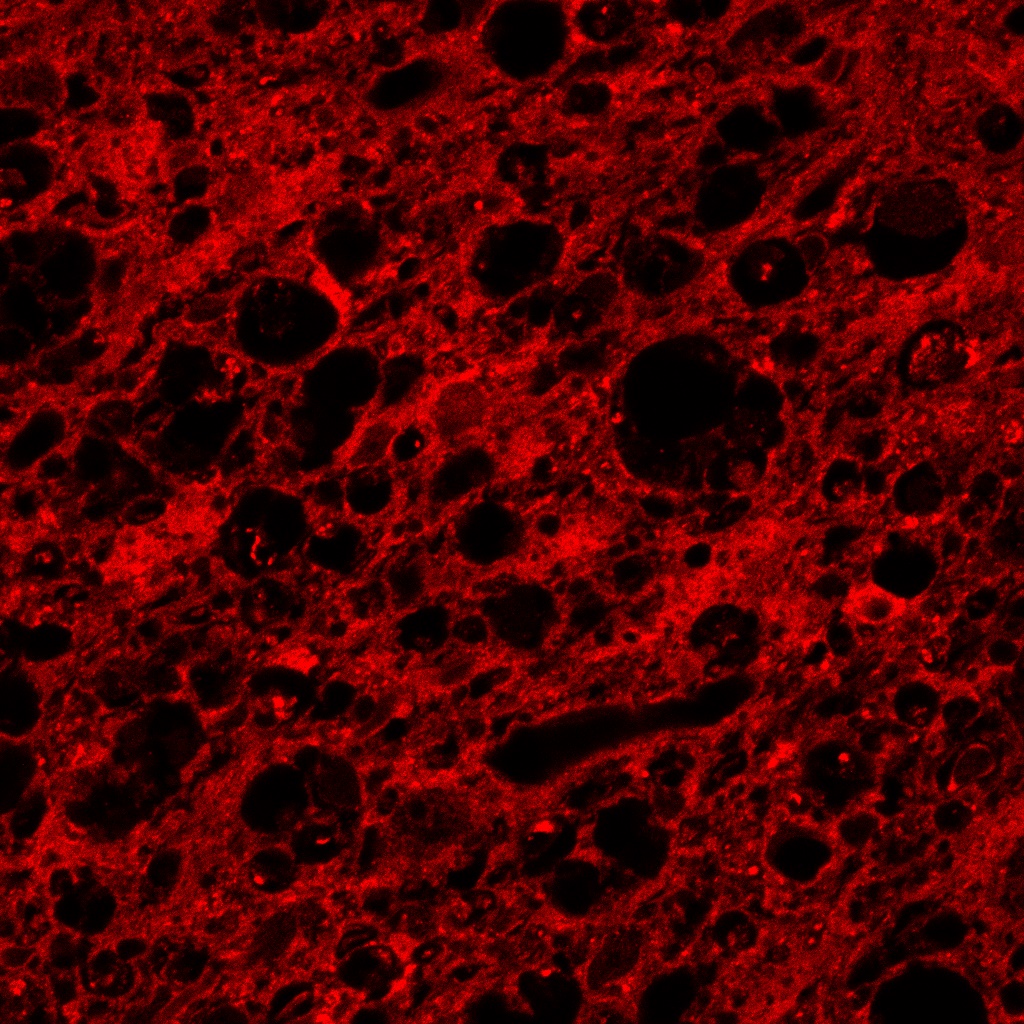

Supplement: Figure 5—source data 1. [file elife-90184-fig5-data1.zip › Figure 5-Source data 1. Raw images (Part 1)/1433 stainning control/Image 9-╡Ñ╕÷╬─╝■╡╝│÷-01_c1.jpg]

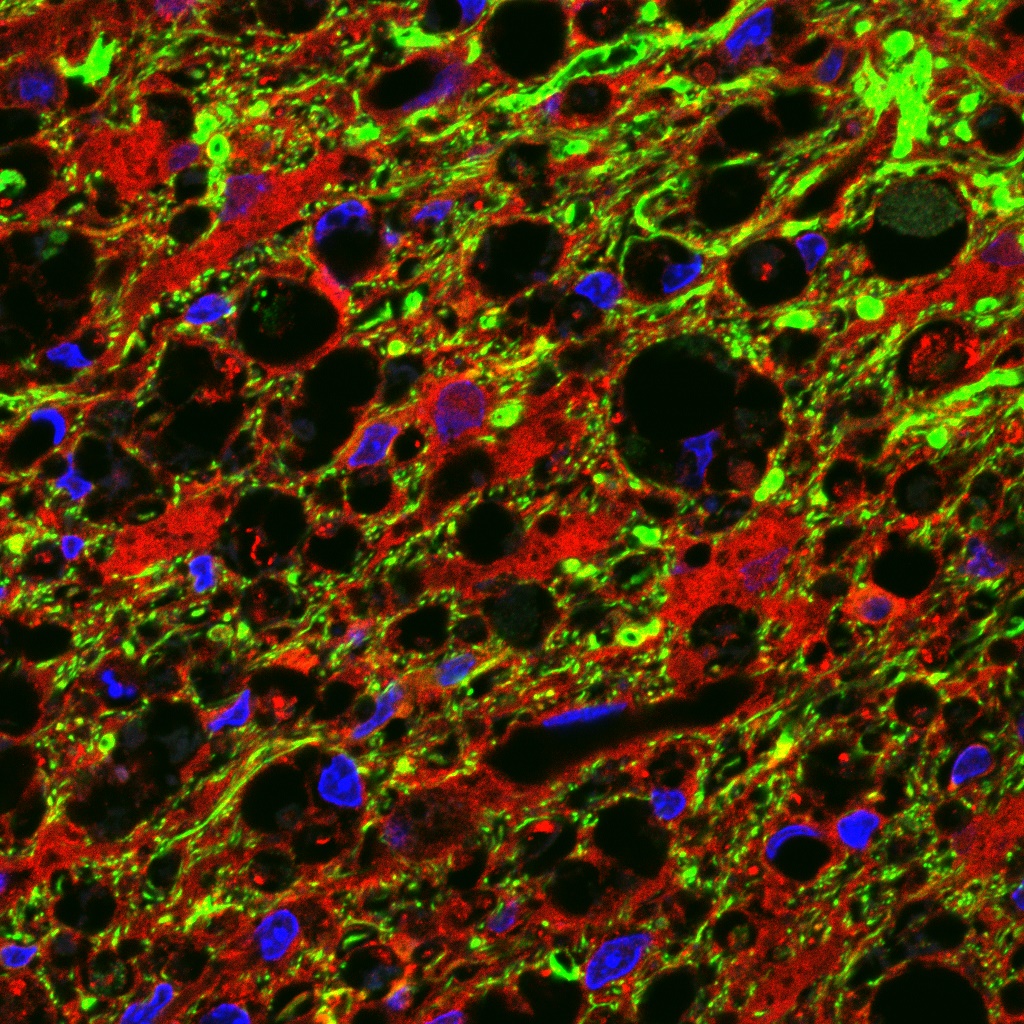

Supplement: Figure 5—source data 1. [file elife-90184-fig5-data1.zip › Figure 5-Source data 1. Raw images (Part 1)/1433 stainning control/Image 9-╡Ñ╕÷╬─╝■╡╝│÷-01_c1+2+3.jpg]

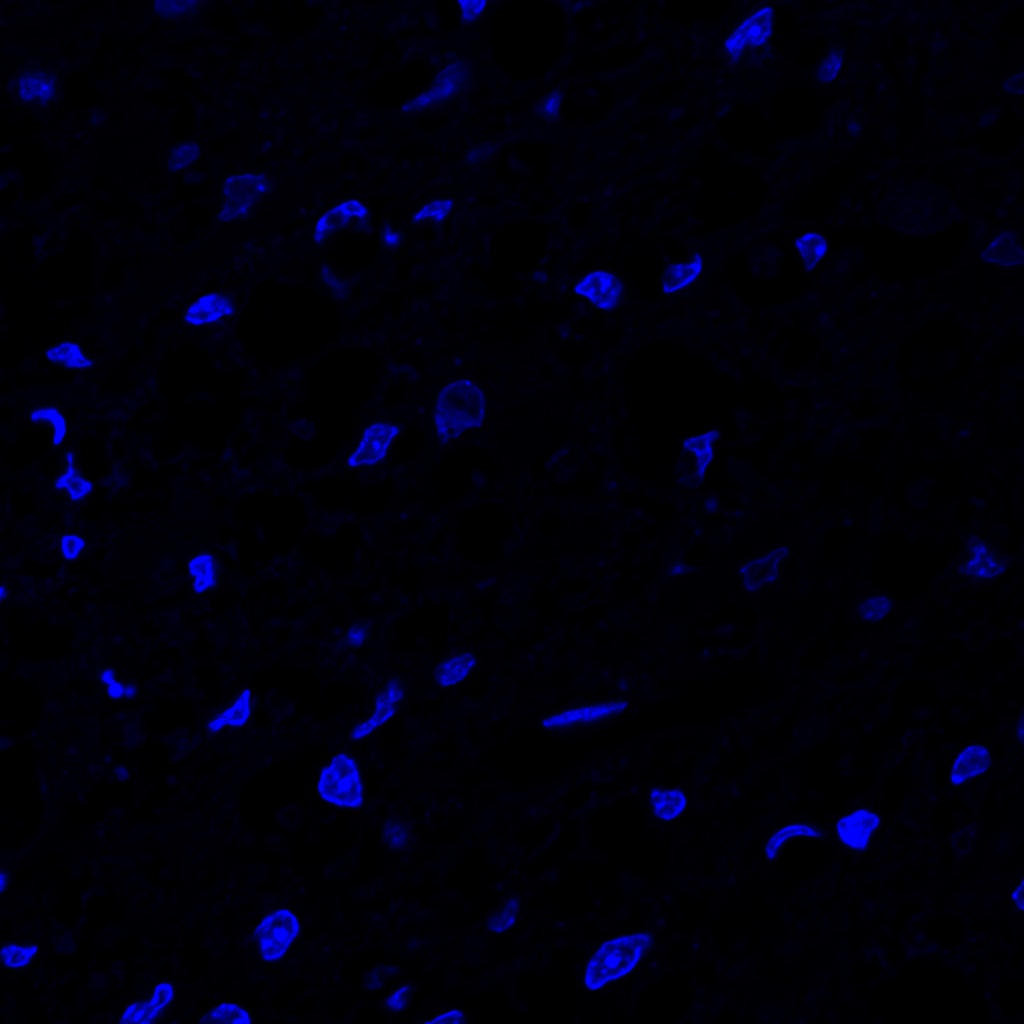

Supplement: Figure 5—source data 1. [file elife-90184-fig5-data1.zip › Figure 5-Source data 1. Raw images (Part 1)/1433 stainning control/Image 9-╡Ñ╕÷╬─╝■╡╝│÷-01_c2.jpg]

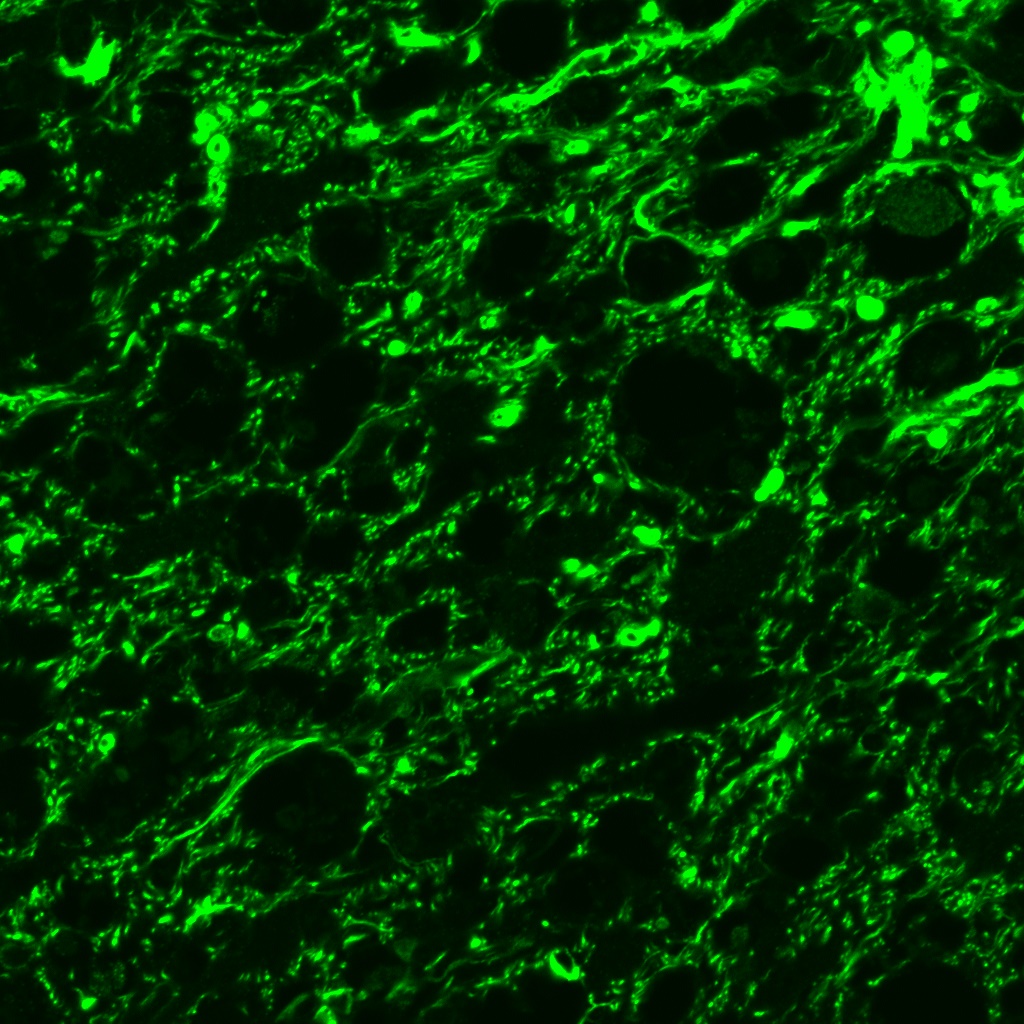

Supplement: Figure 5—source data 1. [file elife-90184-fig5-data1.zip › Figure 5-Source data 1. Raw images (Part 1)/1433 stainning control/Image 9-╡Ñ╕÷╬─╝■╡╝│÷-01_c3.jpg]

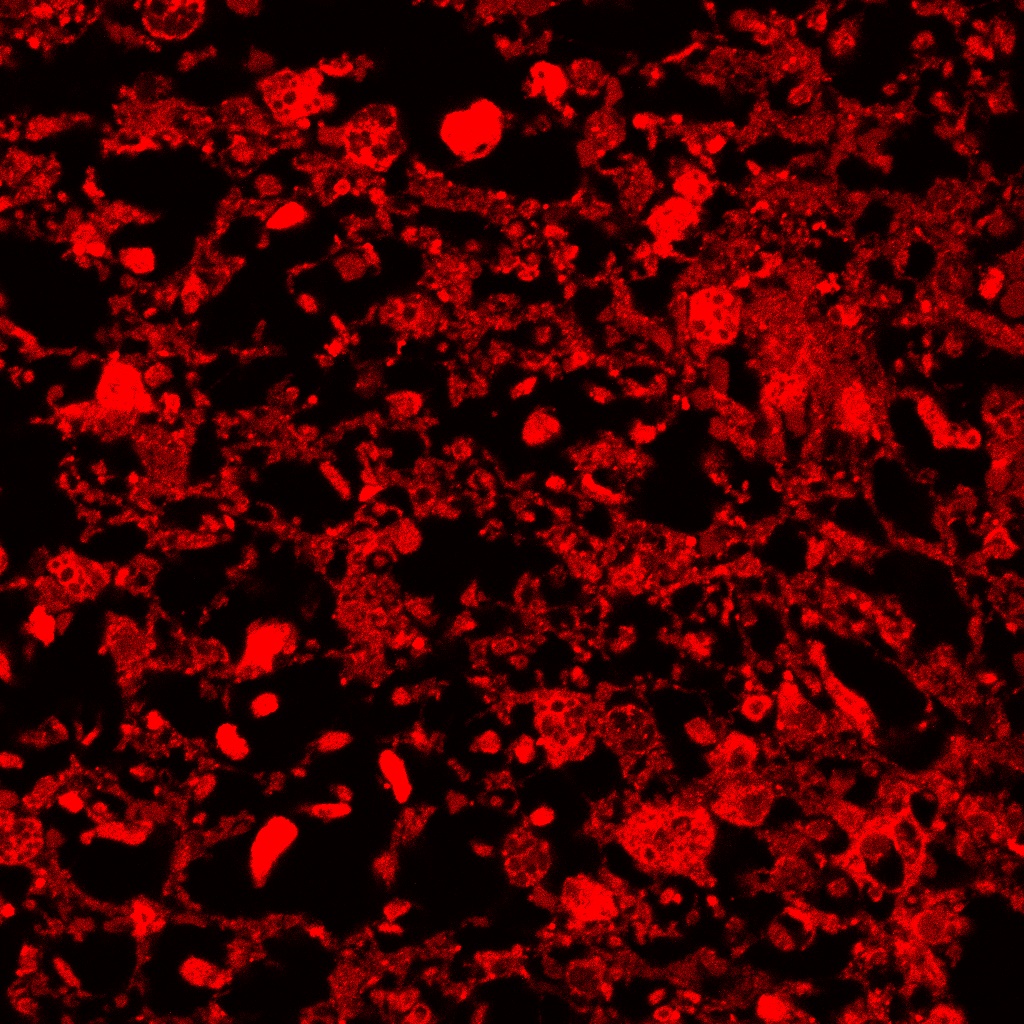

Supplement: Figure 5—source data 1. [file elife-90184-fig5-data1.zip › Figure 5-Source data 1. Raw images (Part 1)/14-3-3 stainning injury group/zhong2-╡Ñ╕÷╬─╝■╡╝│÷-01_c1.jpg]

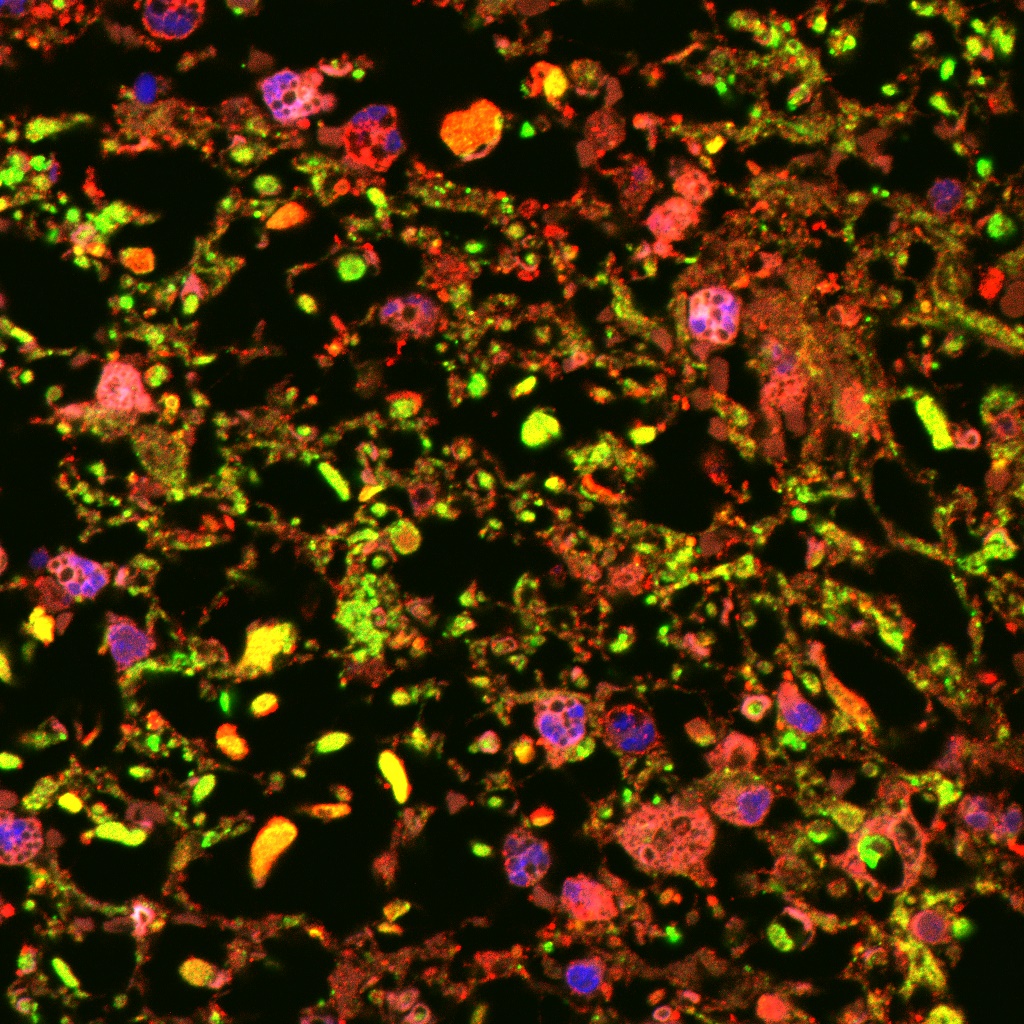

Supplement: Figure 5—source data 1. [file elife-90184-fig5-data1.zip › Figure 5-Source data 1. Raw images (Part 1)/14-3-3 stainning injury group/zhong2-╡Ñ╕÷╬─╝■╡╝│÷-01_c1+2+3.jpg]

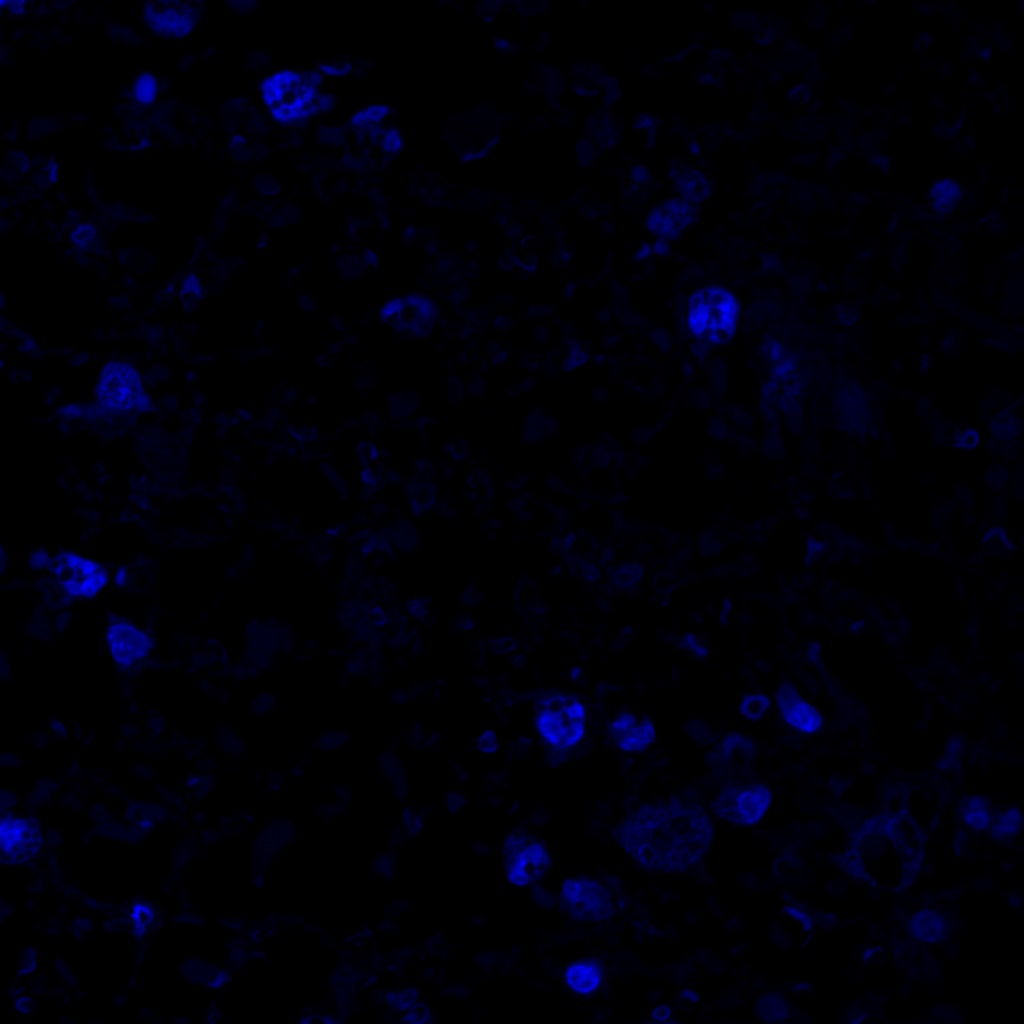

Supplement: Figure 5—source data 1. [file elife-90184-fig5-data1.zip › Figure 5-Source data 1. Raw images (Part 1)/14-3-3 stainning injury group/zhong2-╡Ñ╕÷╬─╝■╡╝│÷-01_c2.jpg]

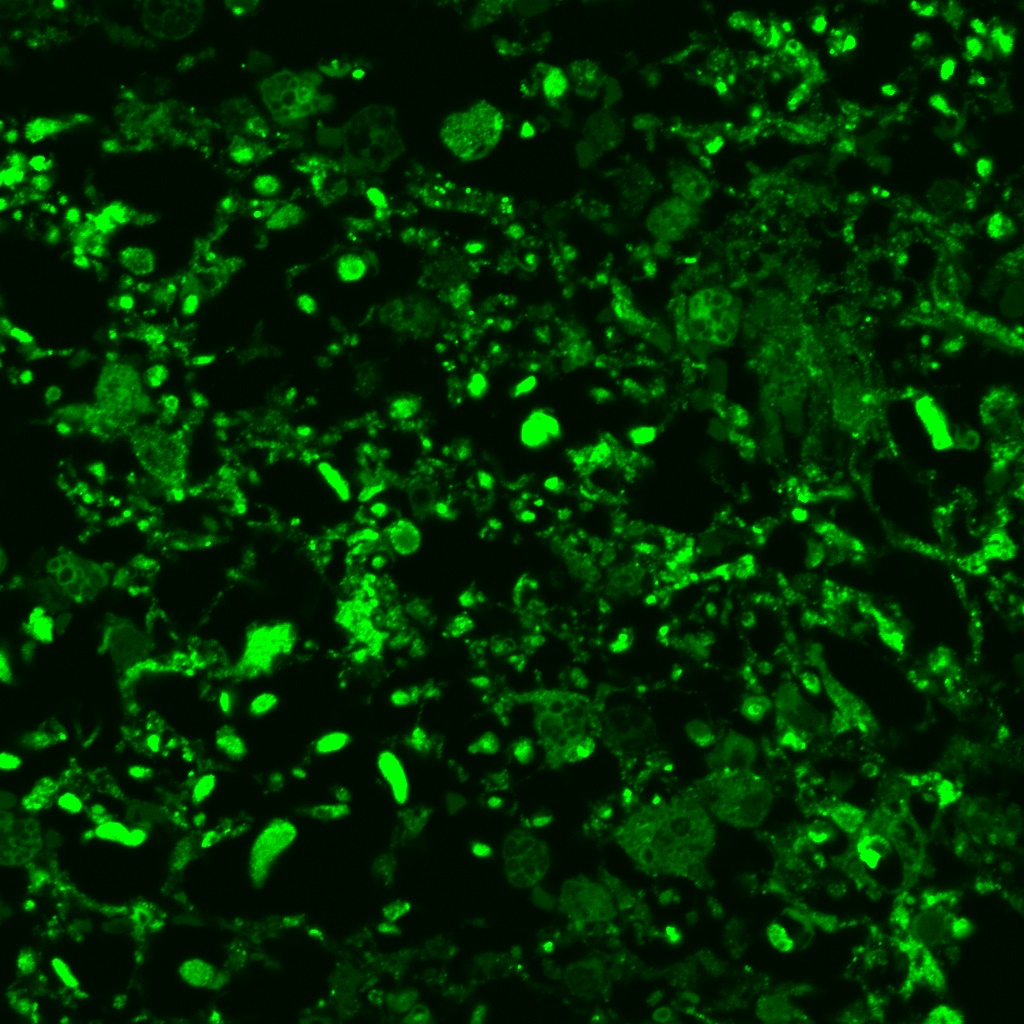

Supplement: Figure 5—source data 1. [file elife-90184-fig5-data1.zip › Figure 5-Source data 1. Raw images (Part 1)/14-3-3 stainning injury group/zhong2-╡Ñ╕÷╬─╝■╡╝│÷-01_c3.jpg]

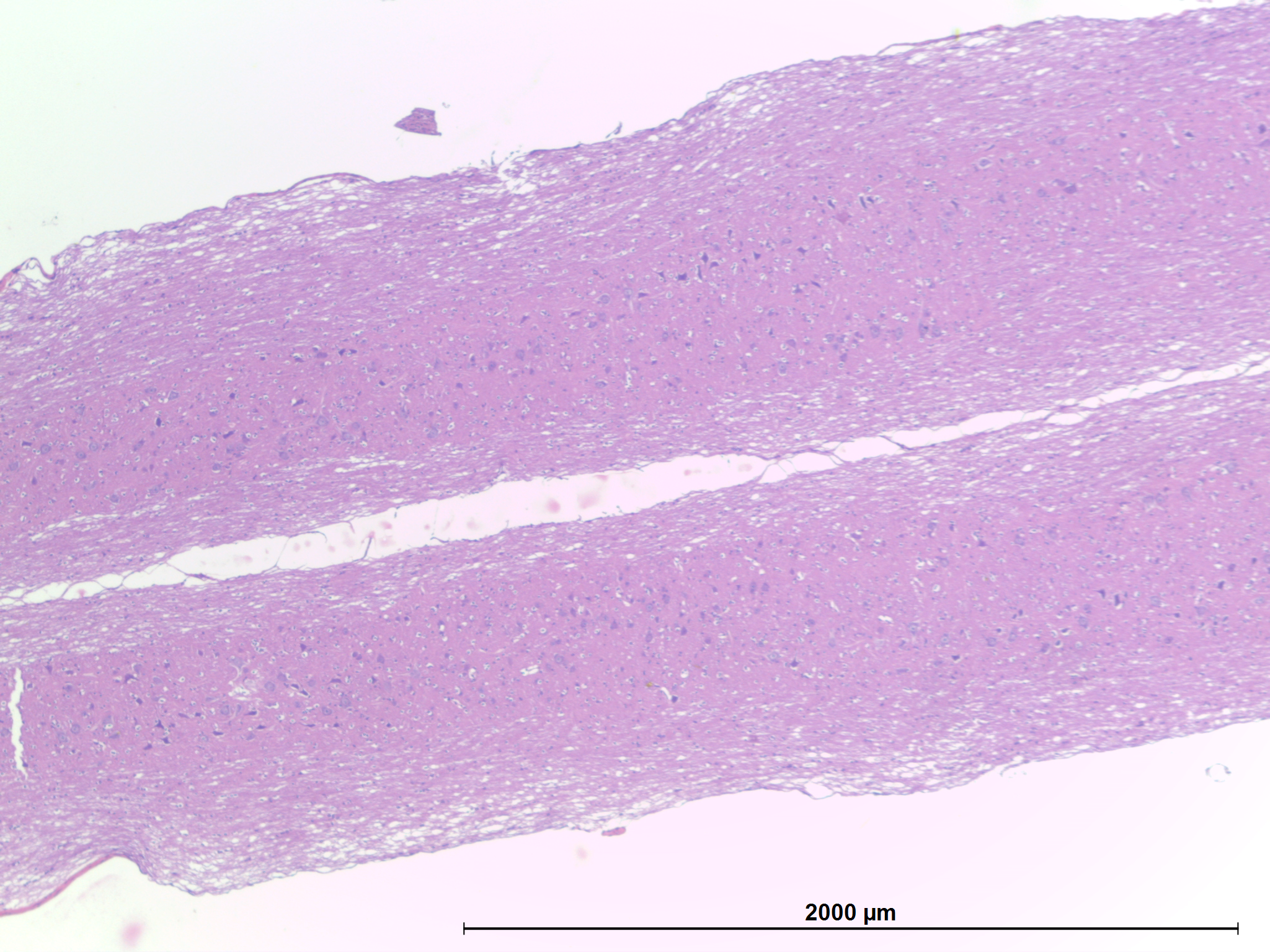

Supplement: Figure 5—source data 1. [file elife-90184-fig5-data1.zip › Figure 5-Source data 1. Raw images (Part 1)/HE stainning/Control1.2.tif]

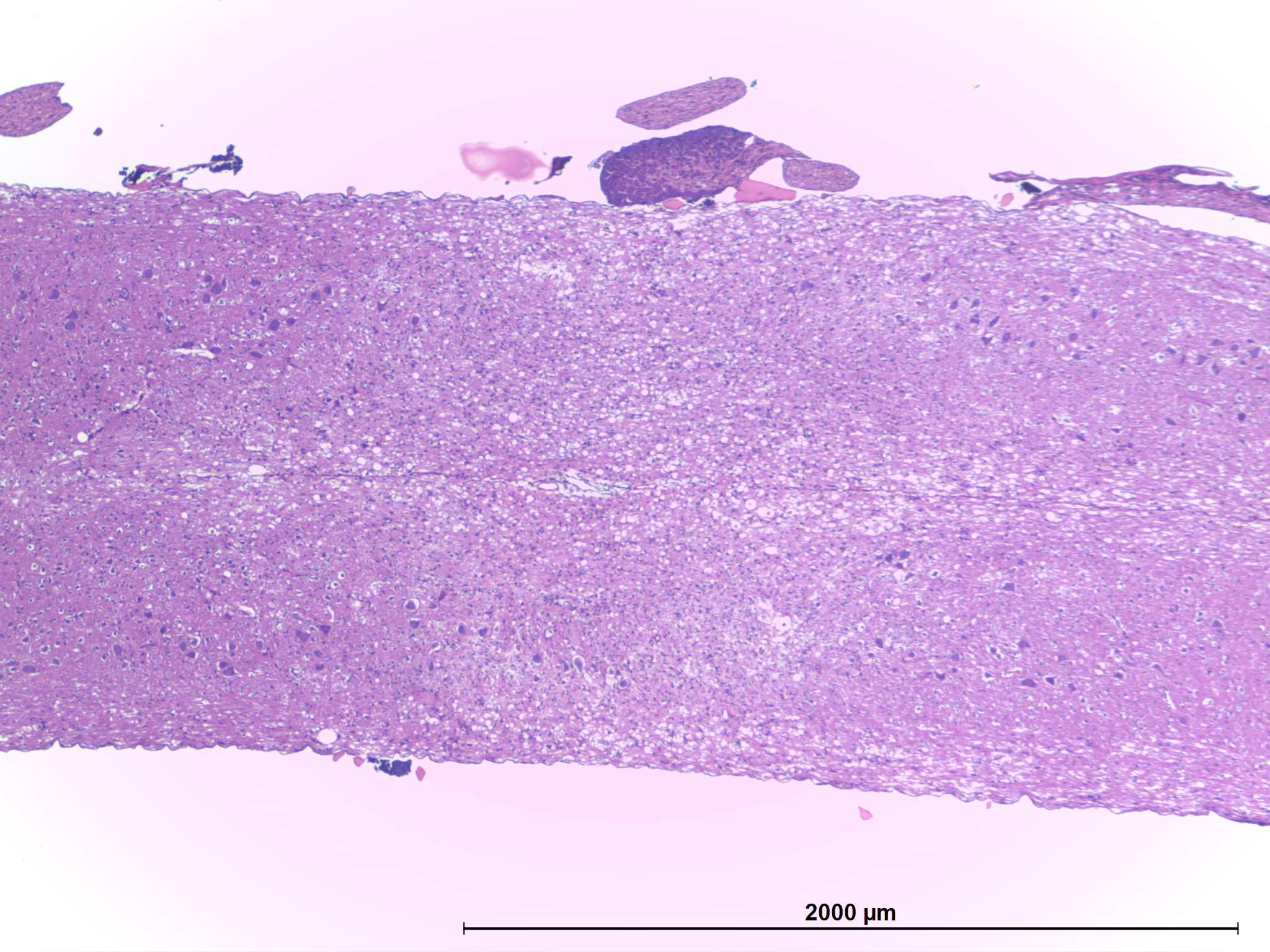

Supplement: Figure 5—source data 1. [file elife-90184-fig5-data1.zip › Figure 5-Source data 1. Raw images (Part 1)/HE stainning/FC.tif]

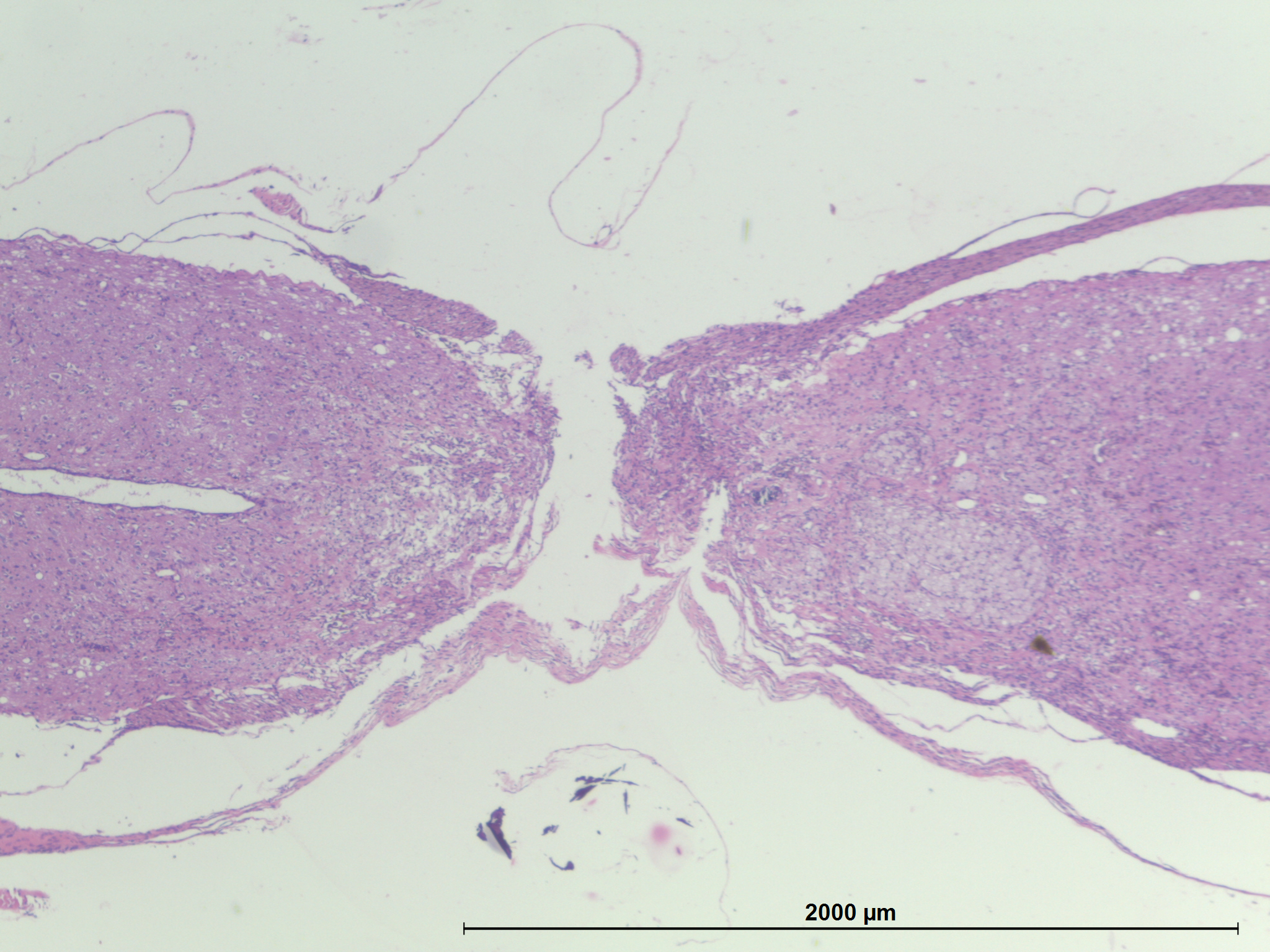

Supplement: Figure 5—source data 1. [file elife-90184-fig5-data1.zip › Figure 5-Source data 1. Raw images (Part 1)/HE stainning/FC+Zoline.tif]

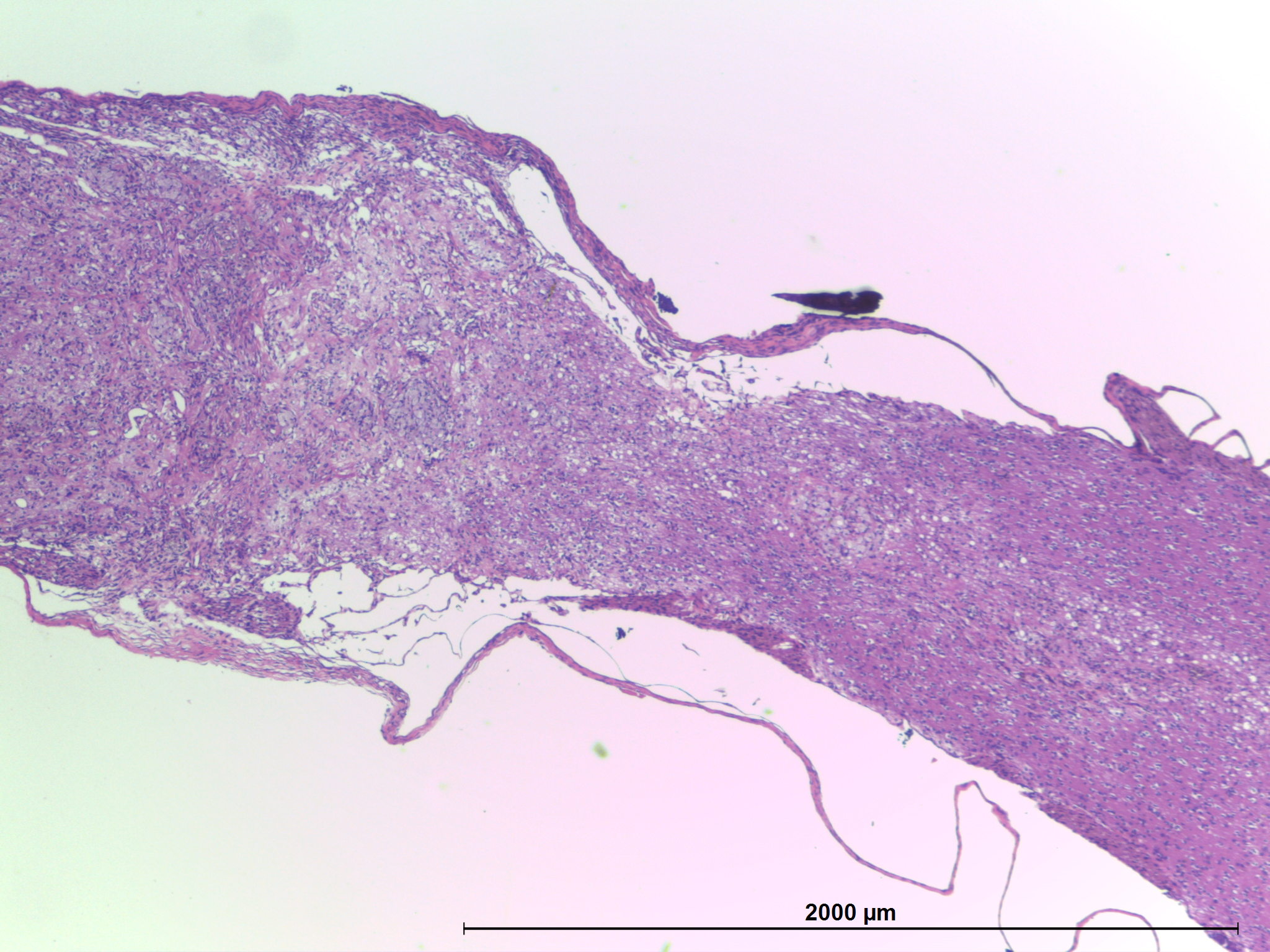

Supplement: Figure 5—source data 1. [file elife-90184-fig5-data1.zip › Figure 5-Source data 1. Raw images (Part 1)/HE stainning/injury1.tif]

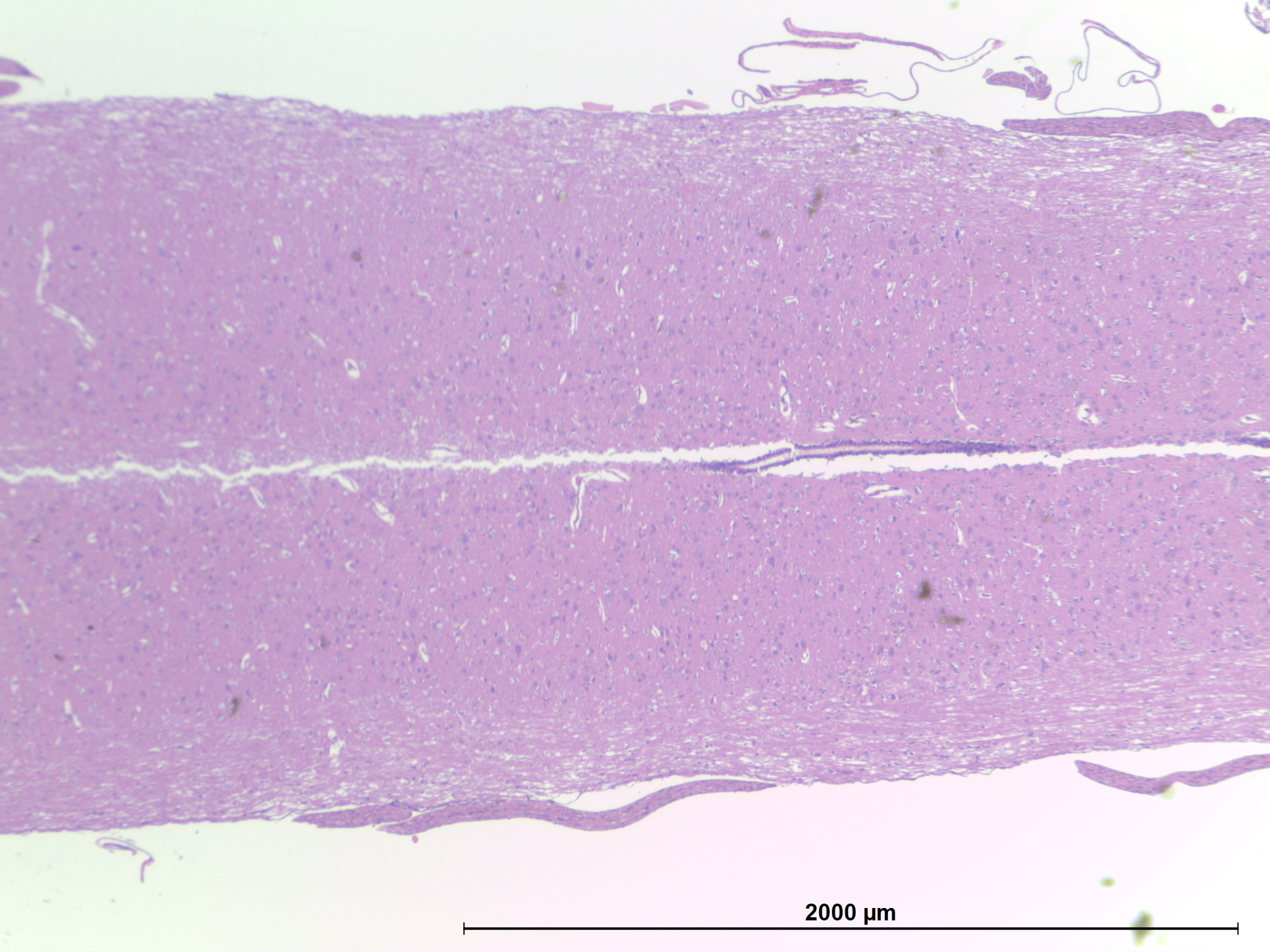

Supplement: Figure 5—source data 1. [file elife-90184-fig5-data1.zip › Figure 5-Source data 1. Raw images (Part 1)/HE stainning/Sham1-1.1.tif]

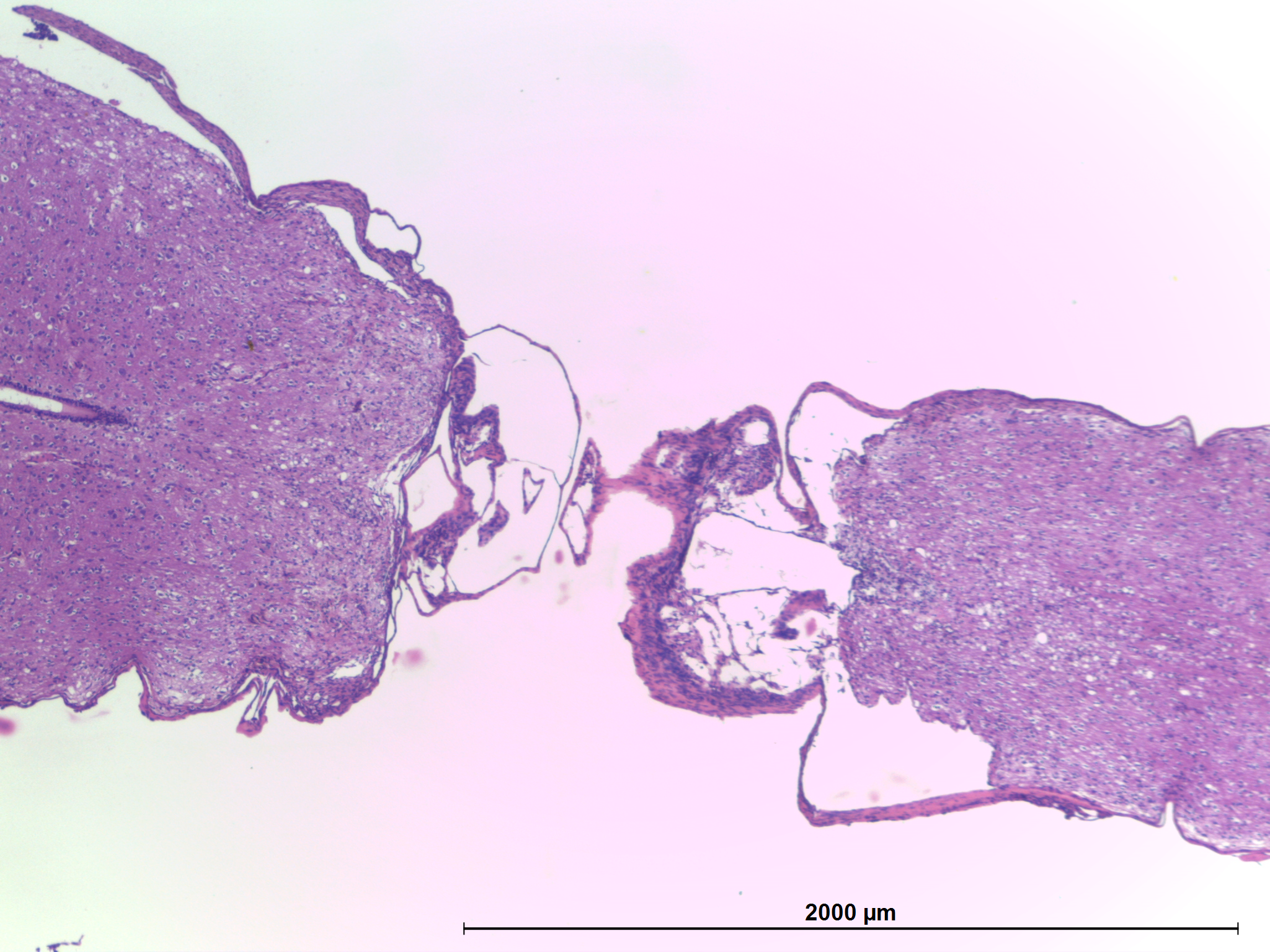

Supplement: Figure 5—source data 1. [file elife-90184-fig5-data1.zip › Figure 5-Source data 1. Raw images (Part 1)/HE stainning/zoline.tif]

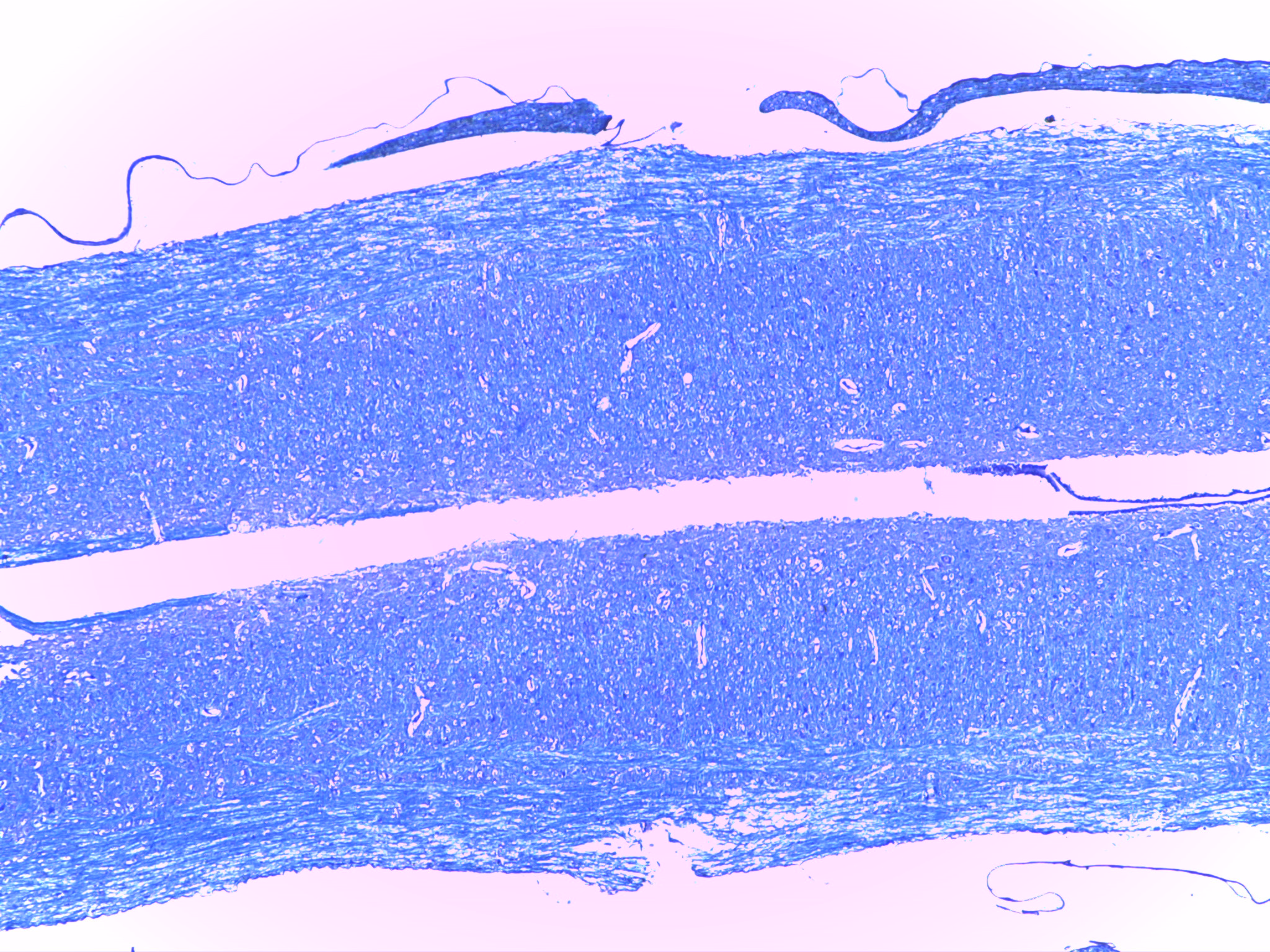

Supplement: Figure 5—source data 1. [file elife-90184-fig5-data1.zip › Figure 5-Source data 1. Raw images (Part 1)/LFB stainning/control.tif]

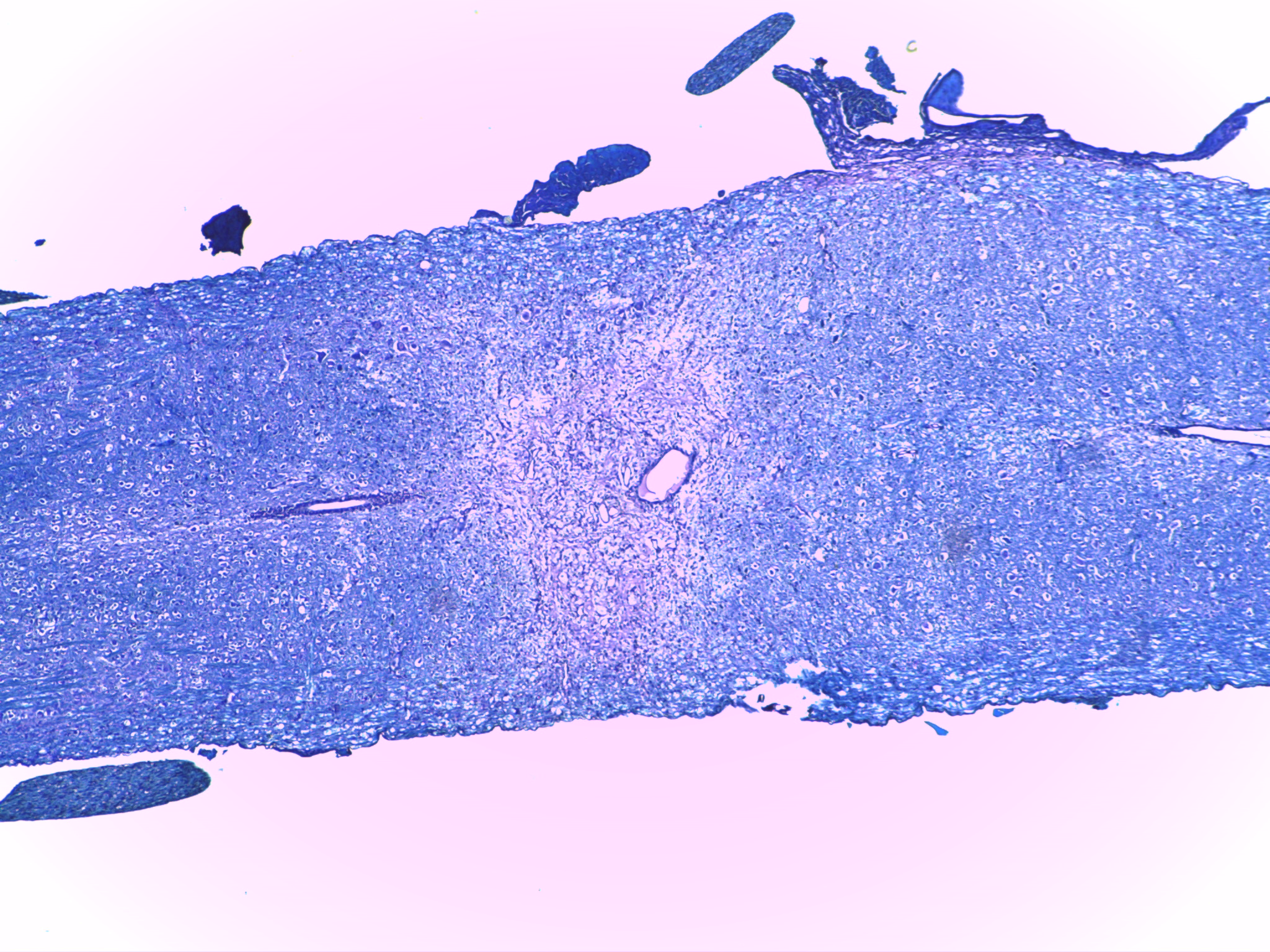

Supplement: Figure 5—source data 1. [file elife-90184-fig5-data1.zip › Figure 5-Source data 1. Raw images (Part 1)/LFB stainning/FC.tif]

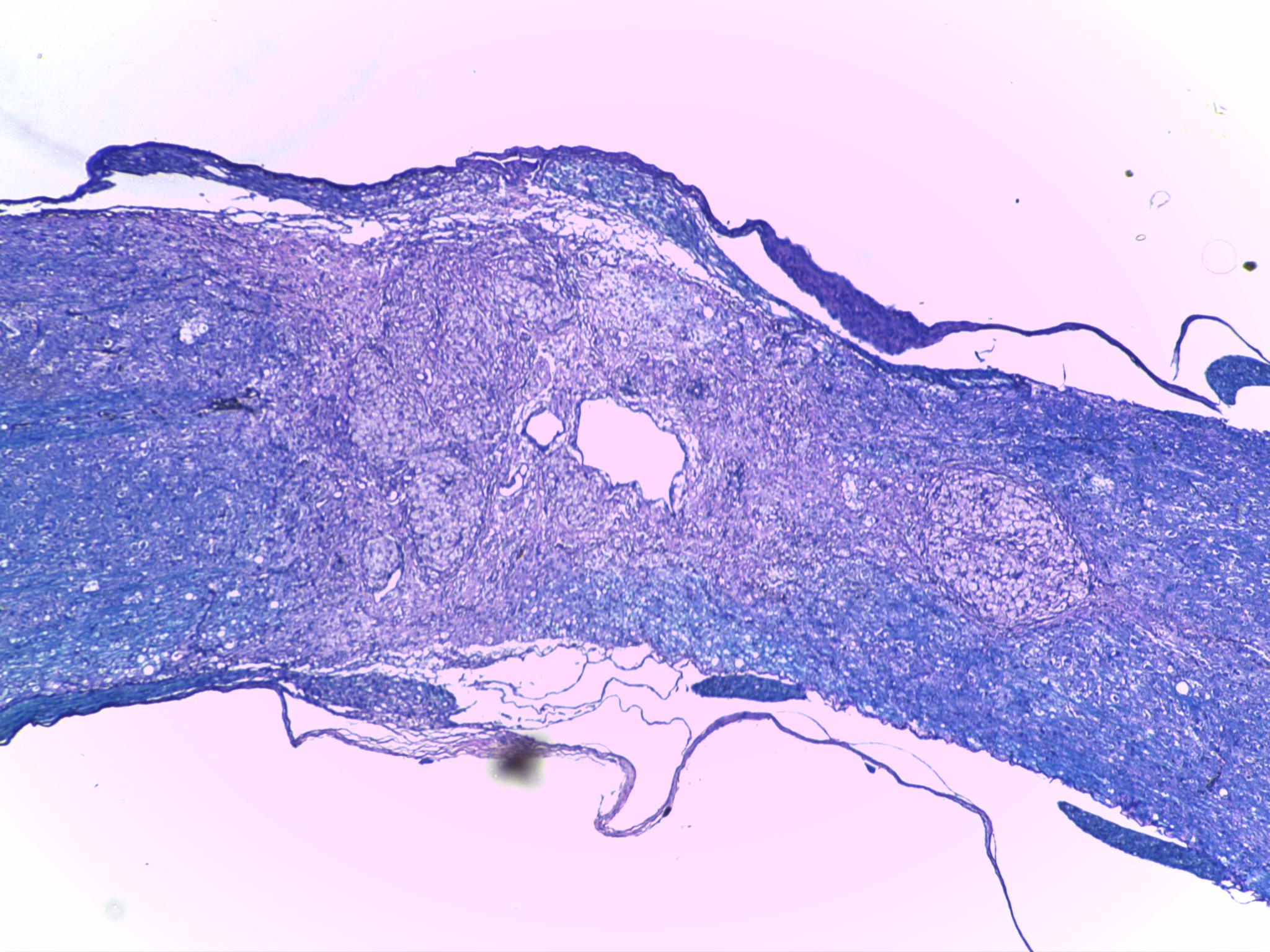

Supplement: Figure 5—source data 1. [file elife-90184-fig5-data1.zip › Figure 5-Source data 1. Raw images (Part 1)/LFB stainning/Injury .tif]

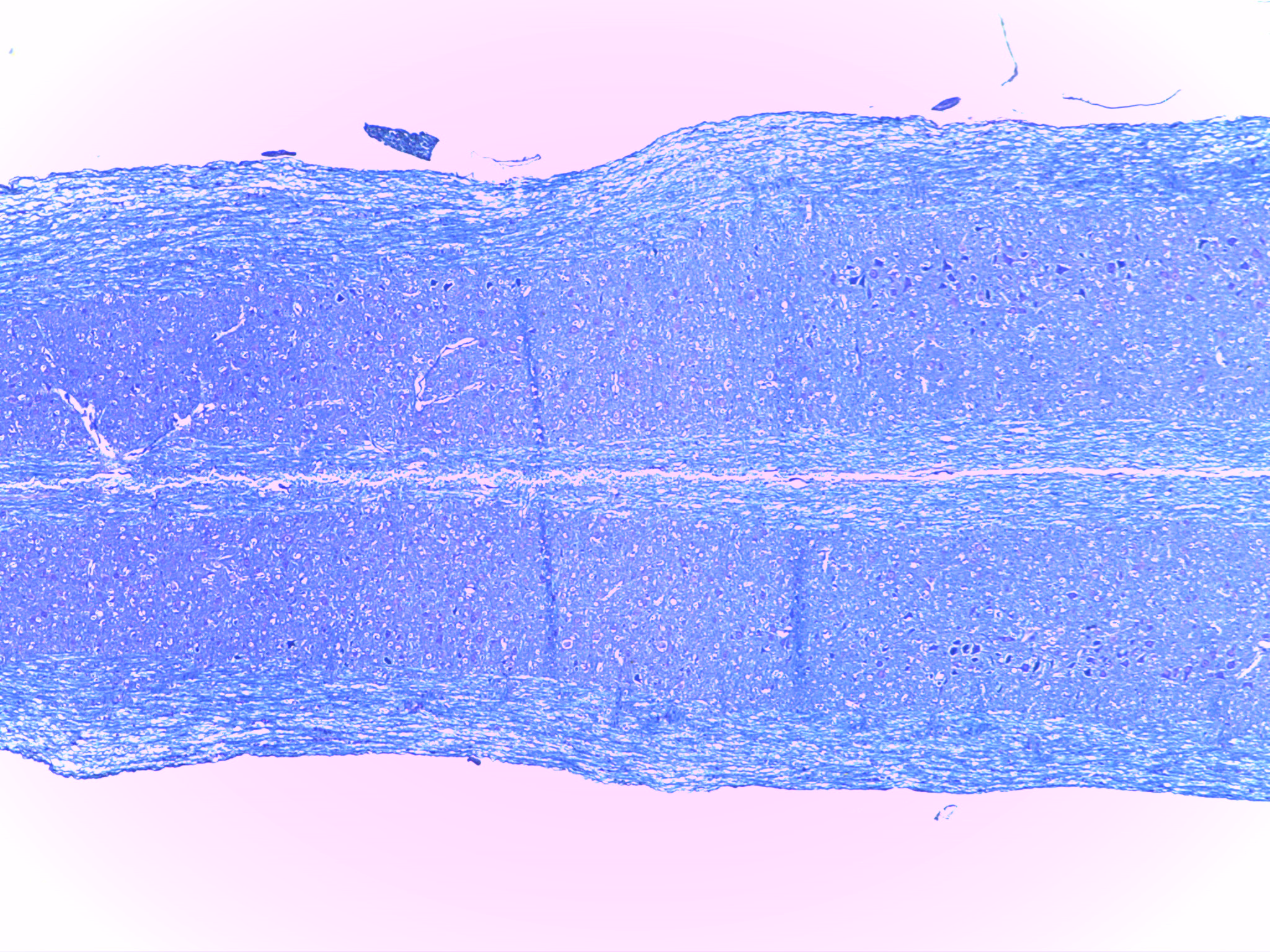

Supplement: Figure 5—source data 1. [file elife-90184-fig5-data1.zip › Figure 5-Source data 1. Raw images (Part 1)/LFB stainning/shamíó.tif]

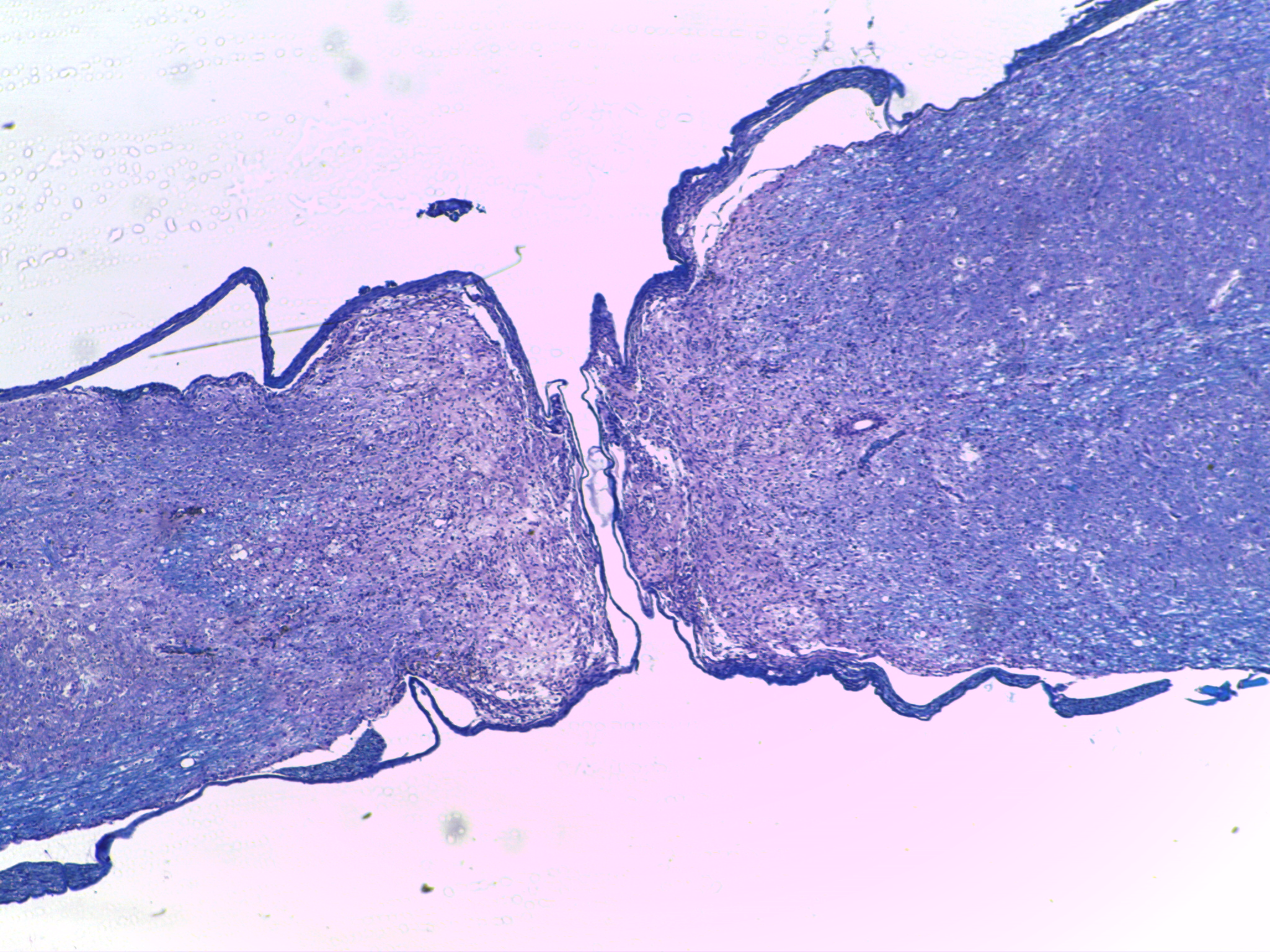

Supplement: Figure 5—source data 1. [file elife-90184-fig5-data1.zip › Figure 5-Source data 1. Raw images (Part 1)/LFB stainning/zoline.tif]

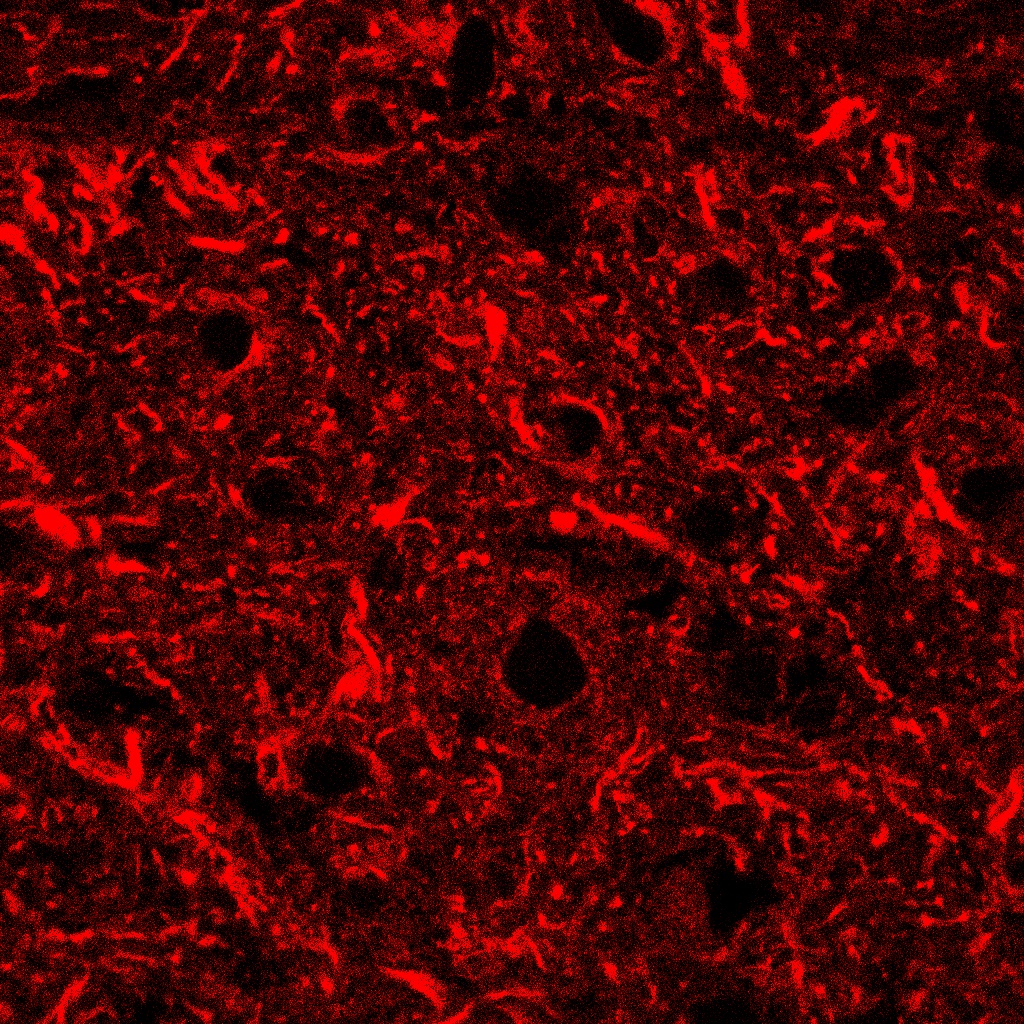

Supplement: Figure 5—source data 1. [file elife-90184-fig5-data1.zip › Figure 5-Source data 1. Raw images (Part 1)/MT dynamics/control-╡Ñ╕÷╬─╝■╡╝│÷-07/control-╡Ñ╕÷╬─╝■╡╝│÷-07_c1.jpg]

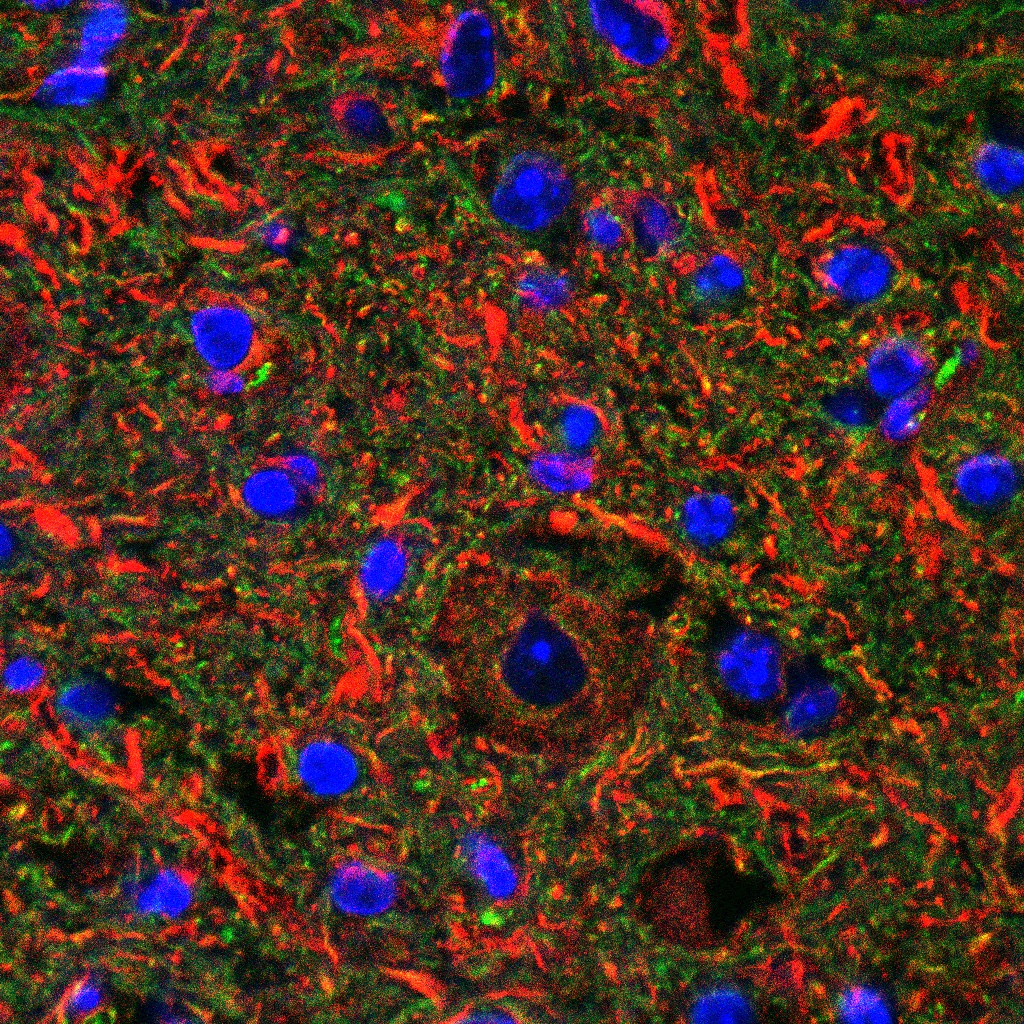

Supplement: Figure 5—source data 1. [file elife-90184-fig5-data1.zip › Figure 5-Source data 1. Raw images (Part 1)/MT dynamics/control-╡Ñ╕÷╬─╝■╡╝│÷-07/control-╡Ñ╕÷╬─╝■╡╝│÷-07_c1+2+3.jpg]

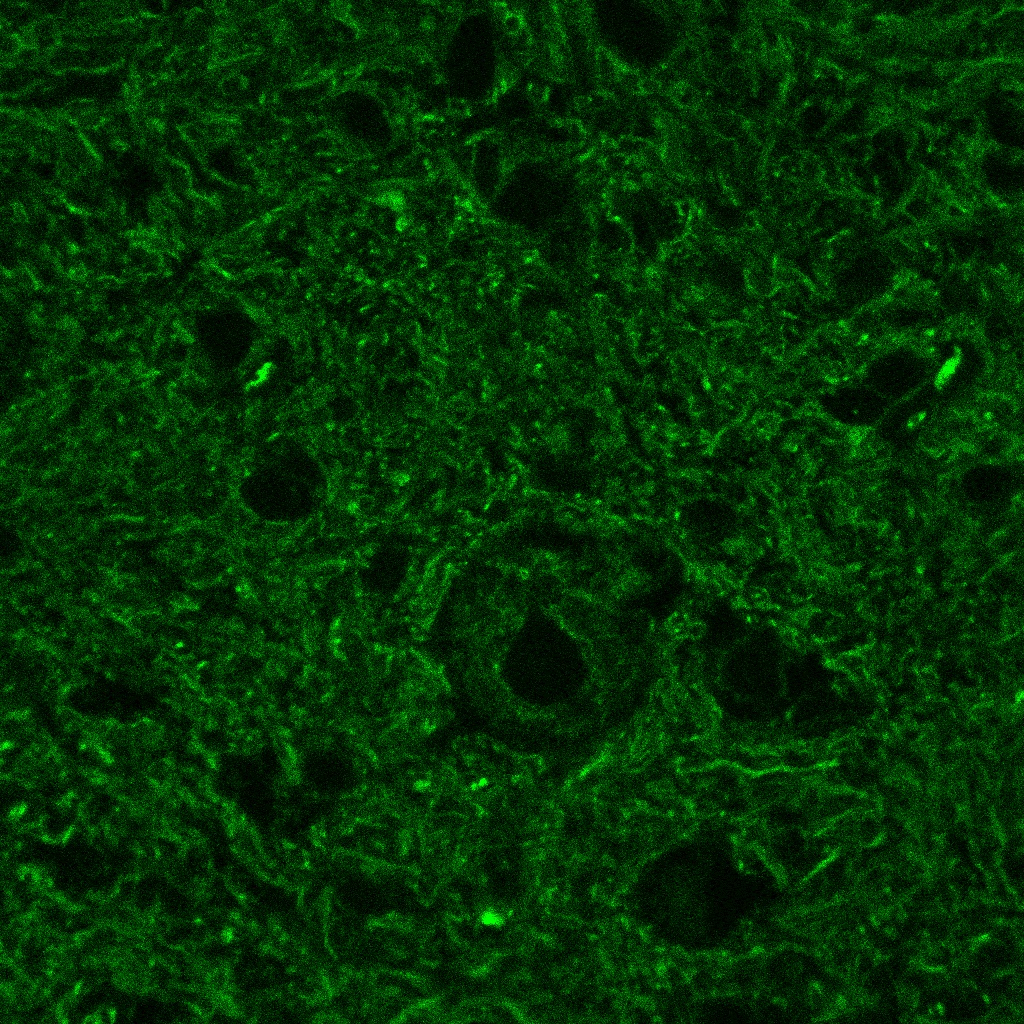

Supplement: Figure 5—source data 1. [file elife-90184-fig5-data1.zip › Figure 5-Source data 1. Raw images (Part 1)/MT dynamics/control-╡Ñ╕÷╬─╝■╡╝│÷-07/control-╡Ñ╕÷╬─╝■╡╝│÷-07_c2.jpg]

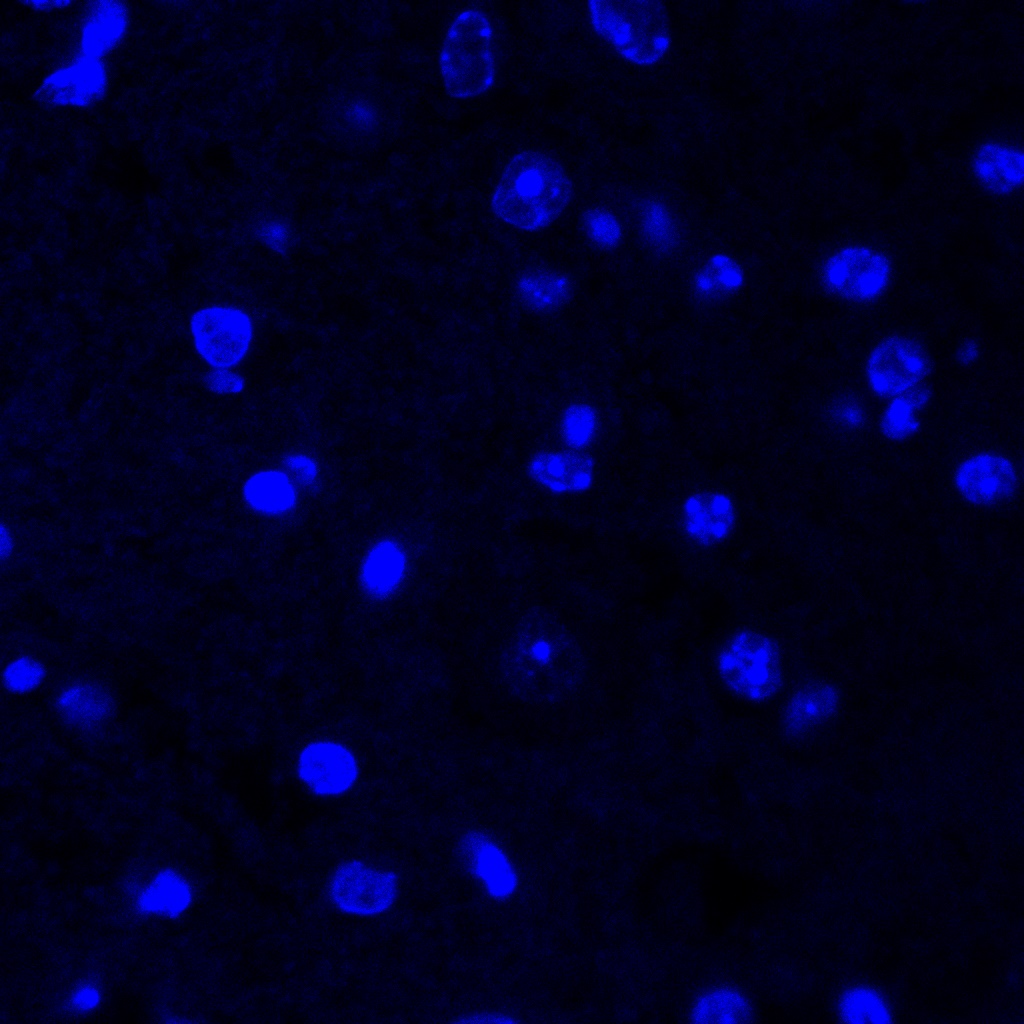

Supplement: Figure 5—source data 1. [file elife-90184-fig5-data1.zip › Figure 5-Source data 1. Raw images (Part 1)/MT dynamics/control-╡Ñ╕÷╬─╝■╡╝│÷-07/control-╡Ñ╕÷╬─╝■╡╝│÷-07_c3.jpg]

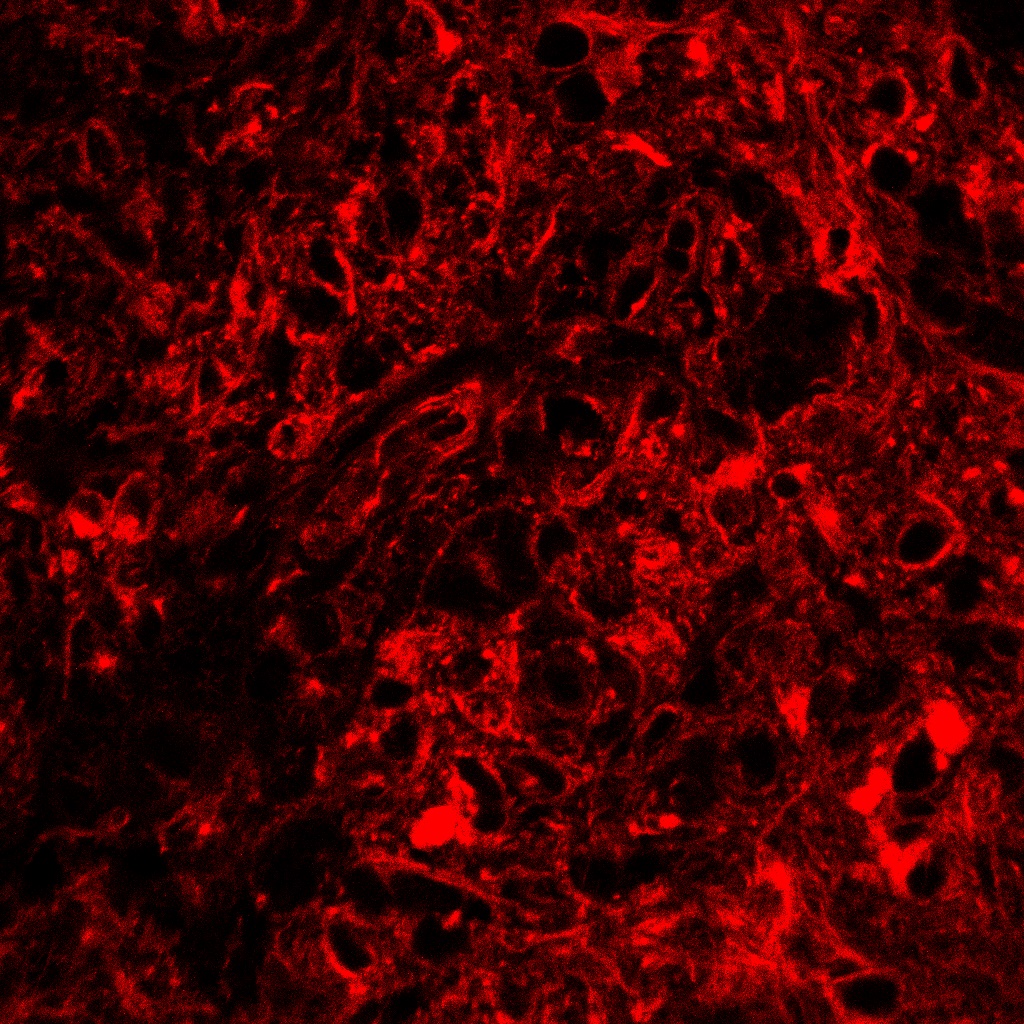

Supplement: Figure 5—source data 1. [file elife-90184-fig5-data1.zip › Figure 5-Source data 1. Raw images (Part 1)/MT dynamics/fC+Zoline-╡Ñ╕÷╬─╝■╡╝│÷-10/fC+Zoline-╡Ñ╕÷╬─╝■╡╝│÷-10_c1.jpg]

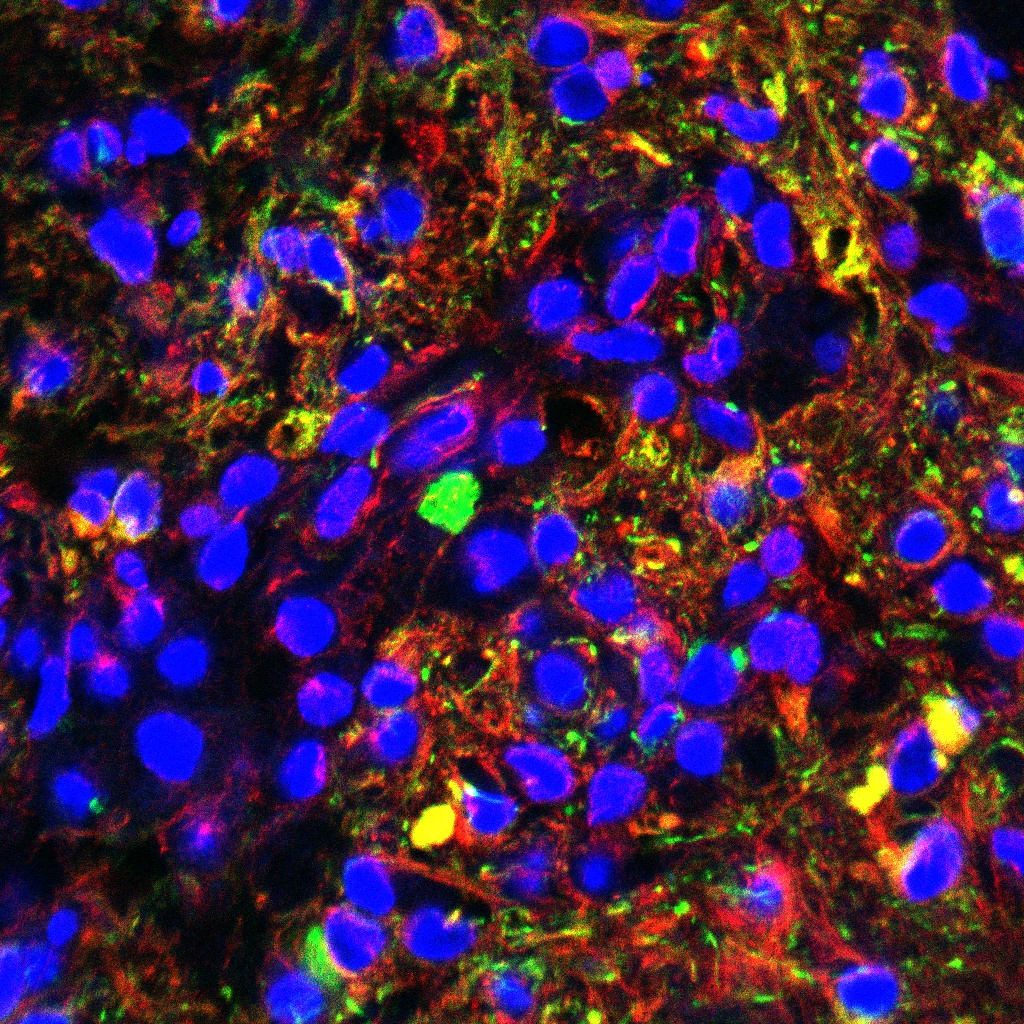

Supplement: Figure 5—source data 1. [file elife-90184-fig5-data1.zip › Figure 5-Source data 1. Raw images (Part 1)/MT dynamics/fC+Zoline-╡Ñ╕÷╬─╝■╡╝│÷-10/fC+Zoline-╡Ñ╕÷╬─╝■╡╝│÷-10_c1+2+3.jpg]

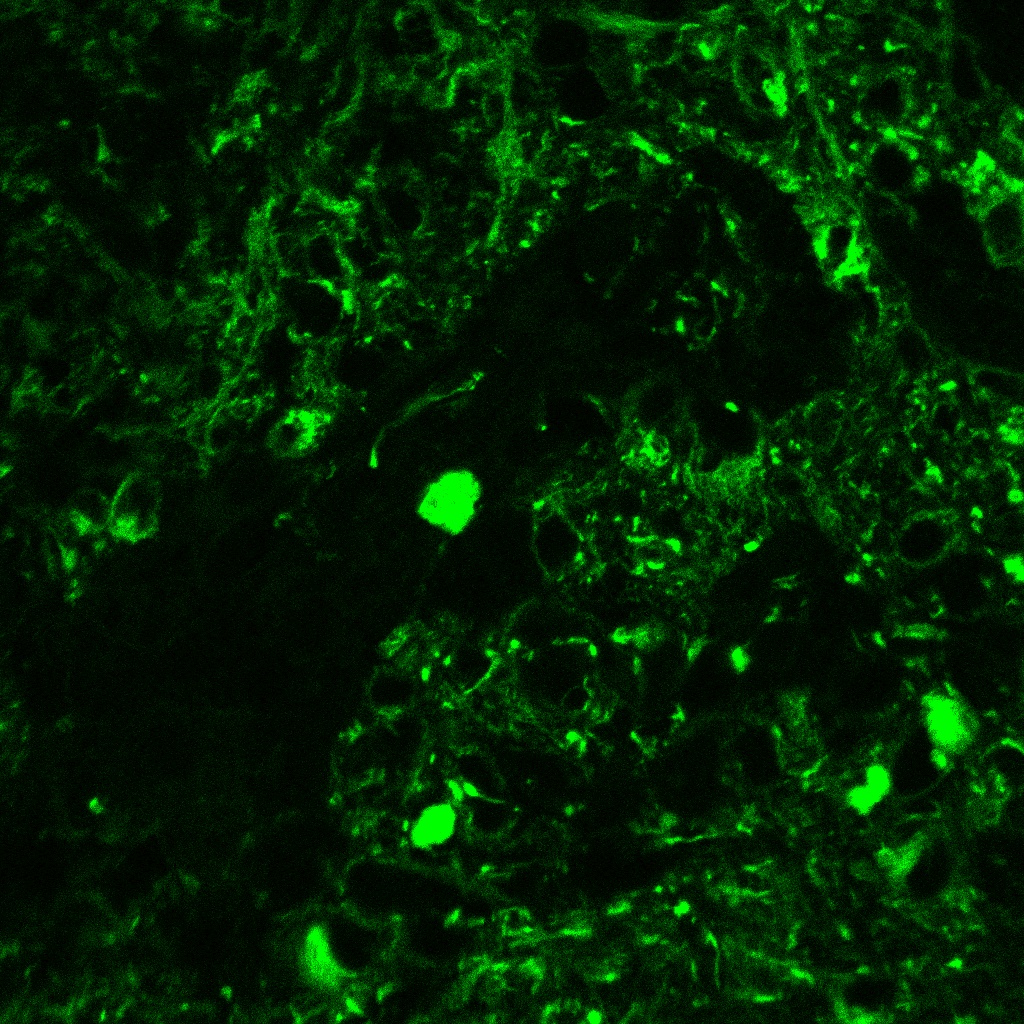

Supplement: Figure 5—source data 1. [file elife-90184-fig5-data1.zip › Figure 5-Source data 1. Raw images (Part 1)/MT dynamics/fC+Zoline-╡Ñ╕÷╬─╝■╡╝│÷-10/fC+Zoline-╡Ñ╕÷╬─╝■╡╝│÷-10_c2.jpg]

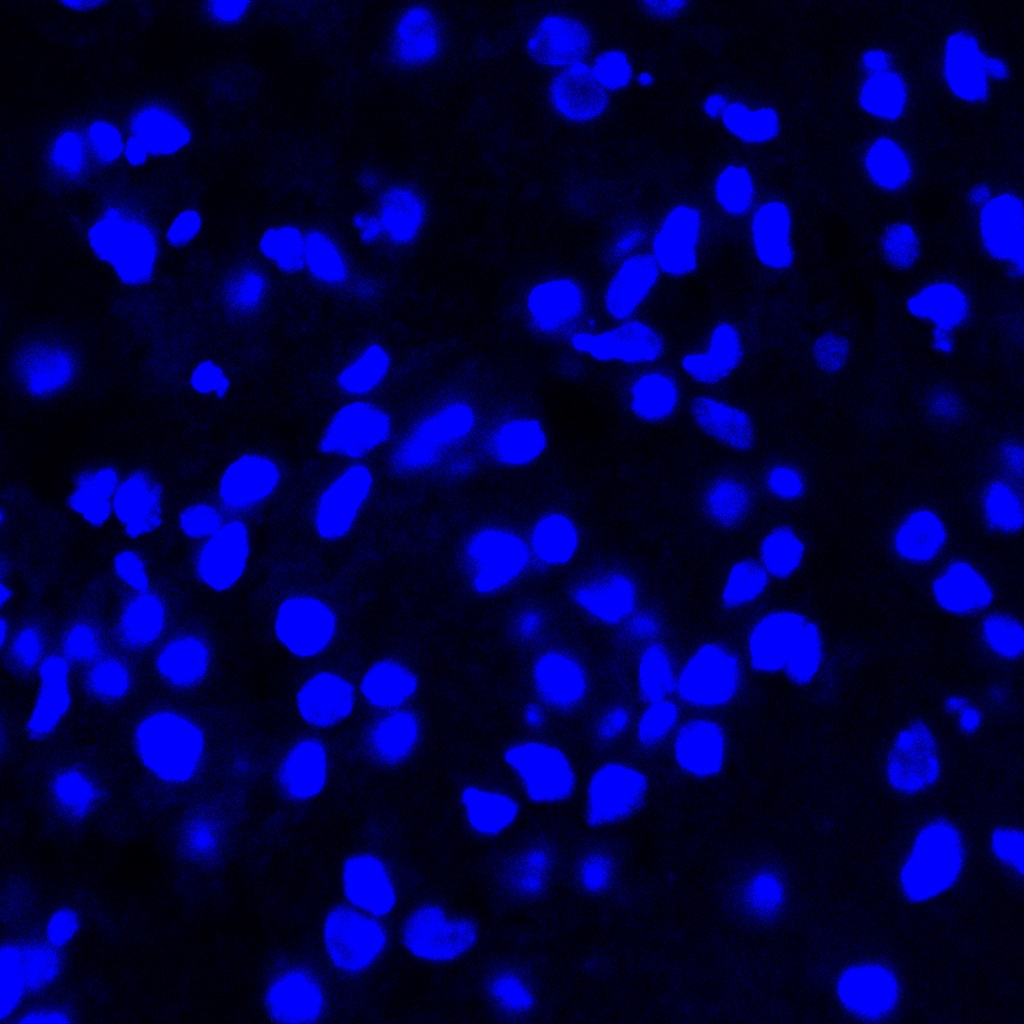

Supplement: Figure 5—source data 1. [file elife-90184-fig5-data1.zip › Figure 5-Source data 1. Raw images (Part 1)/MT dynamics/fC+Zoline-╡Ñ╕÷╬─╝■╡╝│÷-10/fC+Zoline-╡Ñ╕÷╬─╝■╡╝│÷-10_c3.jpg]

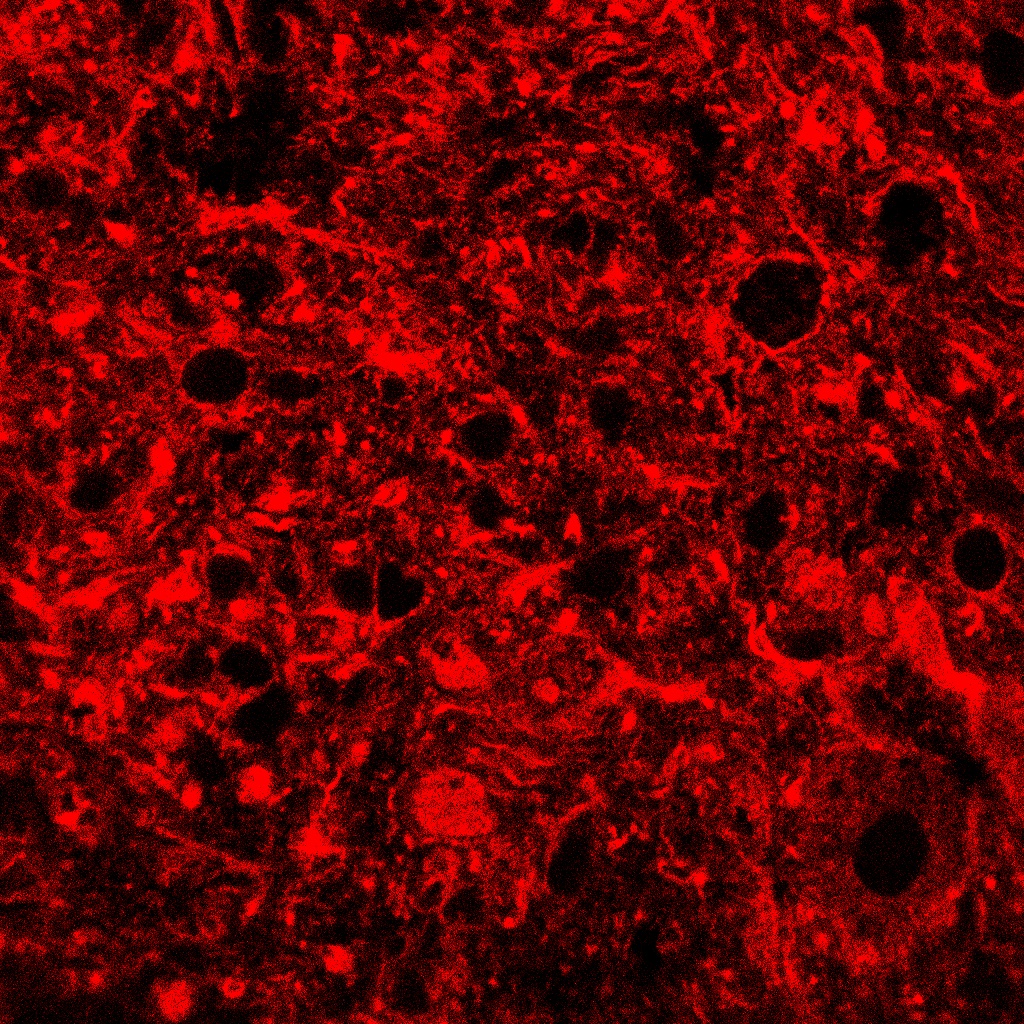

Supplement: Figure 5—source data 1. [file elife-90184-fig5-data1.zip › Figure 5-Source data 1. Raw images (Part 1)/MT dynamics/FC-╡Ñ╕÷╬─╝■╡╝│÷-09/FC-╡Ñ╕÷╬─╝■╡╝│÷-09_c1.jpg]

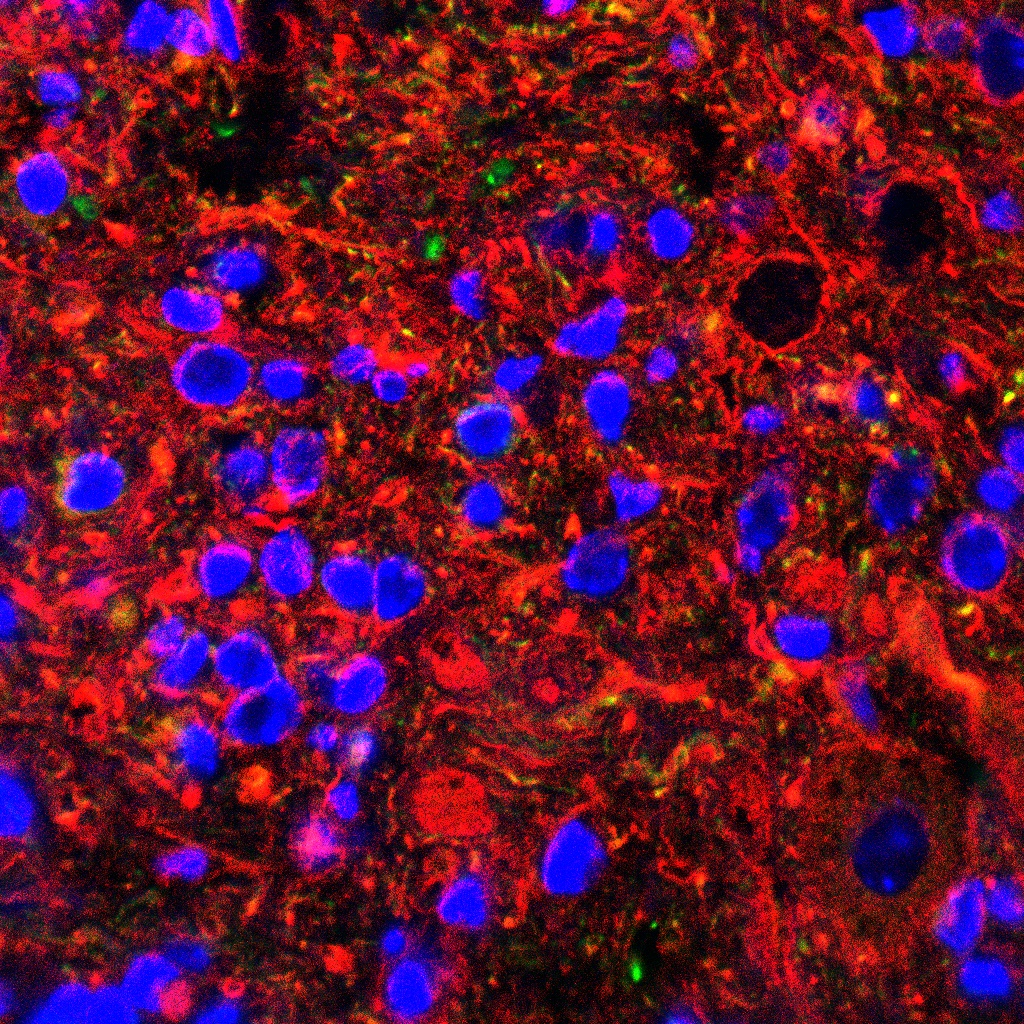

Supplement: Figure 5—source data 1. [file elife-90184-fig5-data1.zip › Figure 5-Source data 1. Raw images (Part 1)/MT dynamics/FC-╡Ñ╕÷╬─╝■╡╝│÷-09/FC-╡Ñ╕÷╬─╝■╡╝│÷-09_c1+2+3.jpg]

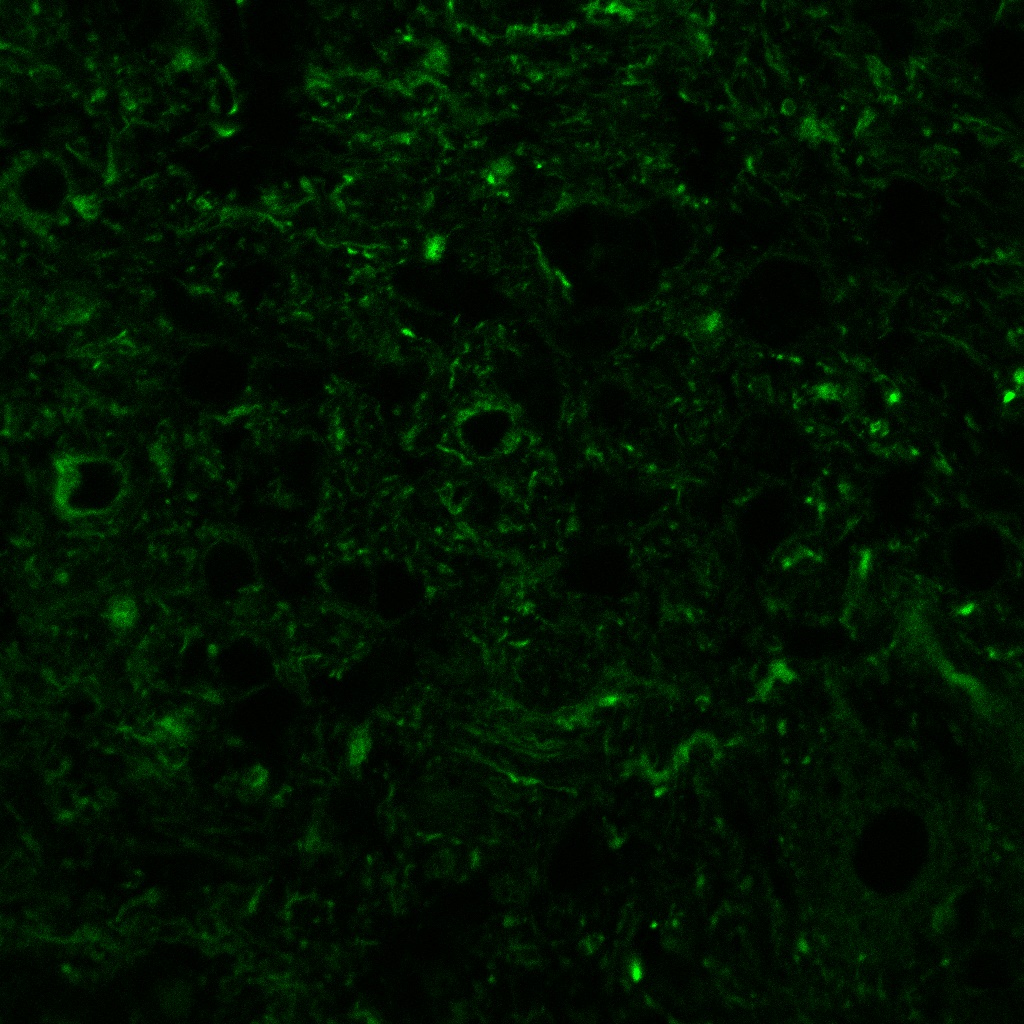

Supplement: Figure 5—source data 1. [file elife-90184-fig5-data1.zip › Figure 5-Source data 1. Raw images (Part 1)/MT dynamics/FC-╡Ñ╕÷╬─╝■╡╝│÷-09/FC-╡Ñ╕÷╬─╝■╡╝│÷-09_c2.jpg]

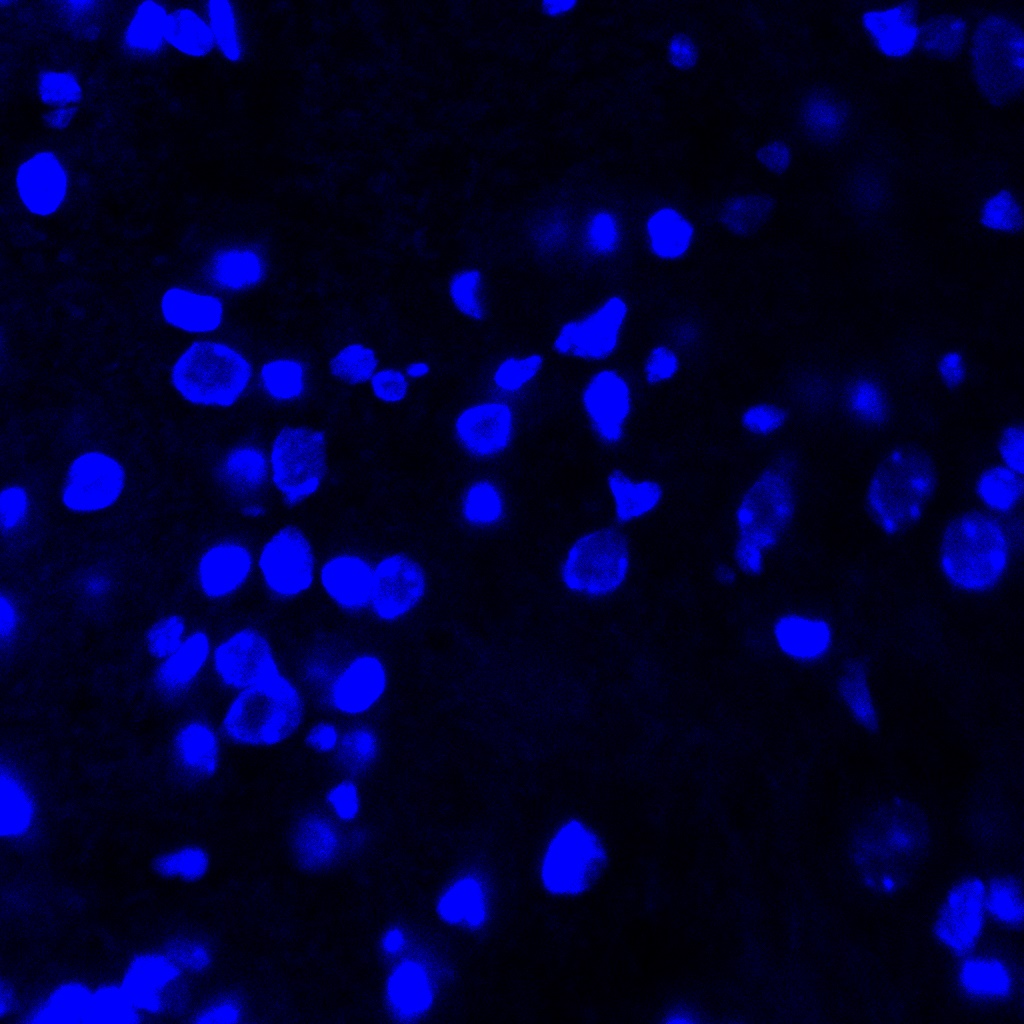

Supplement: Figure 5—source data 1. [file elife-90184-fig5-data1.zip › Figure 5-Source data 1. Raw images (Part 1)/MT dynamics/FC-╡Ñ╕÷╬─╝■╡╝│÷-09/FC-╡Ñ╕÷╬─╝■╡╝│÷-09_c3.jpg]

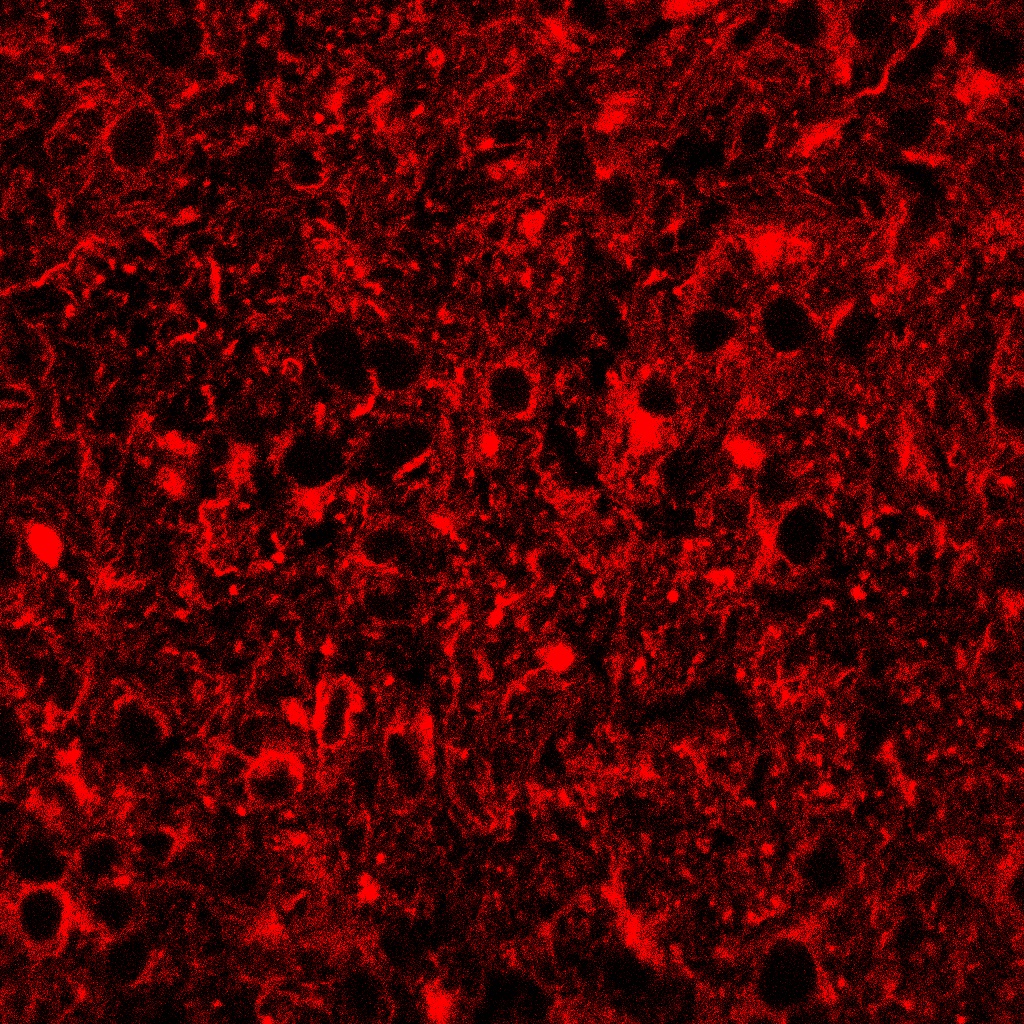

Supplement: Figure 5—source data 1. [file elife-90184-fig5-data1.zip › Figure 5-Source data 1. Raw images (Part 1)/MT dynamics/Zoline-╡Ñ╕÷╬─╝■╡╝│÷-05/Zoline-╡Ñ╕÷╬─╝■╡╝│÷-05_c1.jpg]

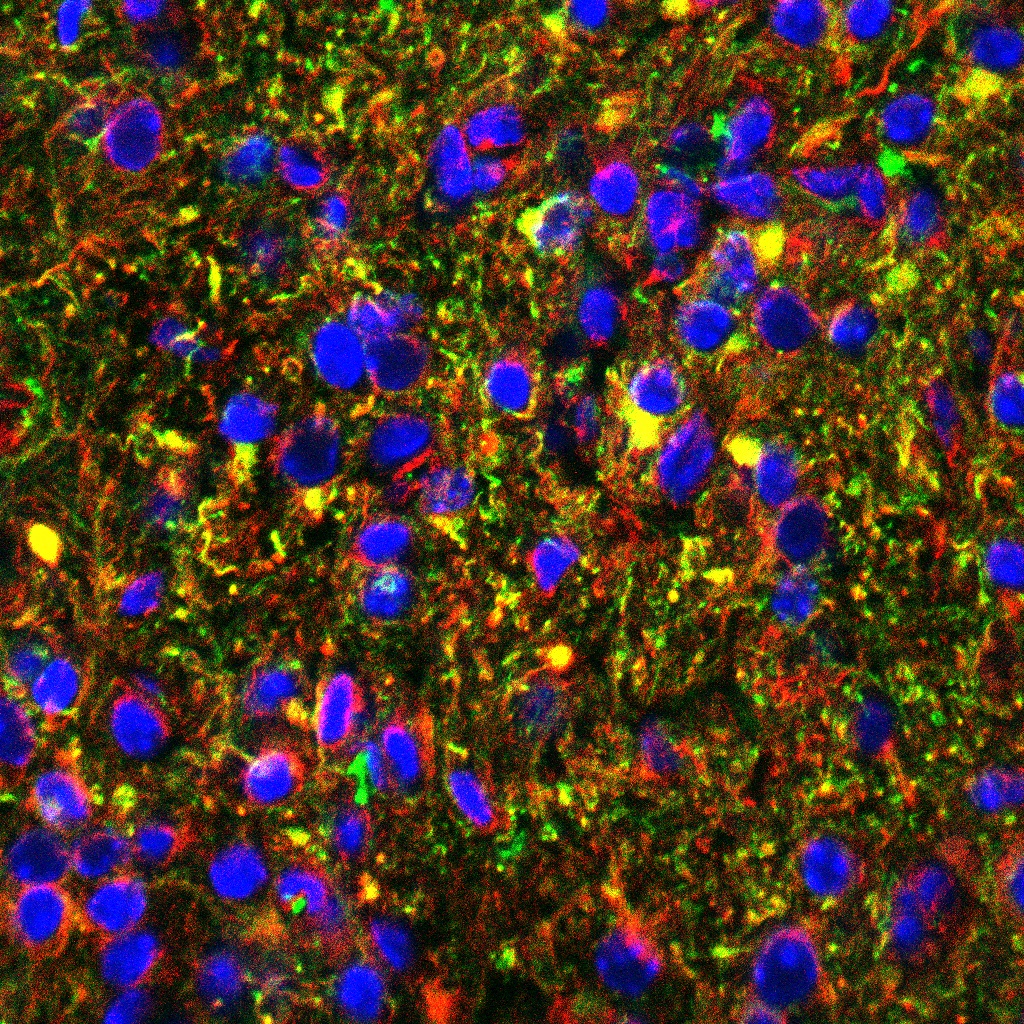

Supplement: Figure 5—source data 1. [file elife-90184-fig5-data1.zip › Figure 5-Source data 1. Raw images (Part 1)/MT dynamics/Zoline-╡Ñ╕÷╬─╝■╡╝│÷-05/Zoline-╡Ñ╕÷╬─╝■╡╝│÷-05_c1+2+3.jpg]

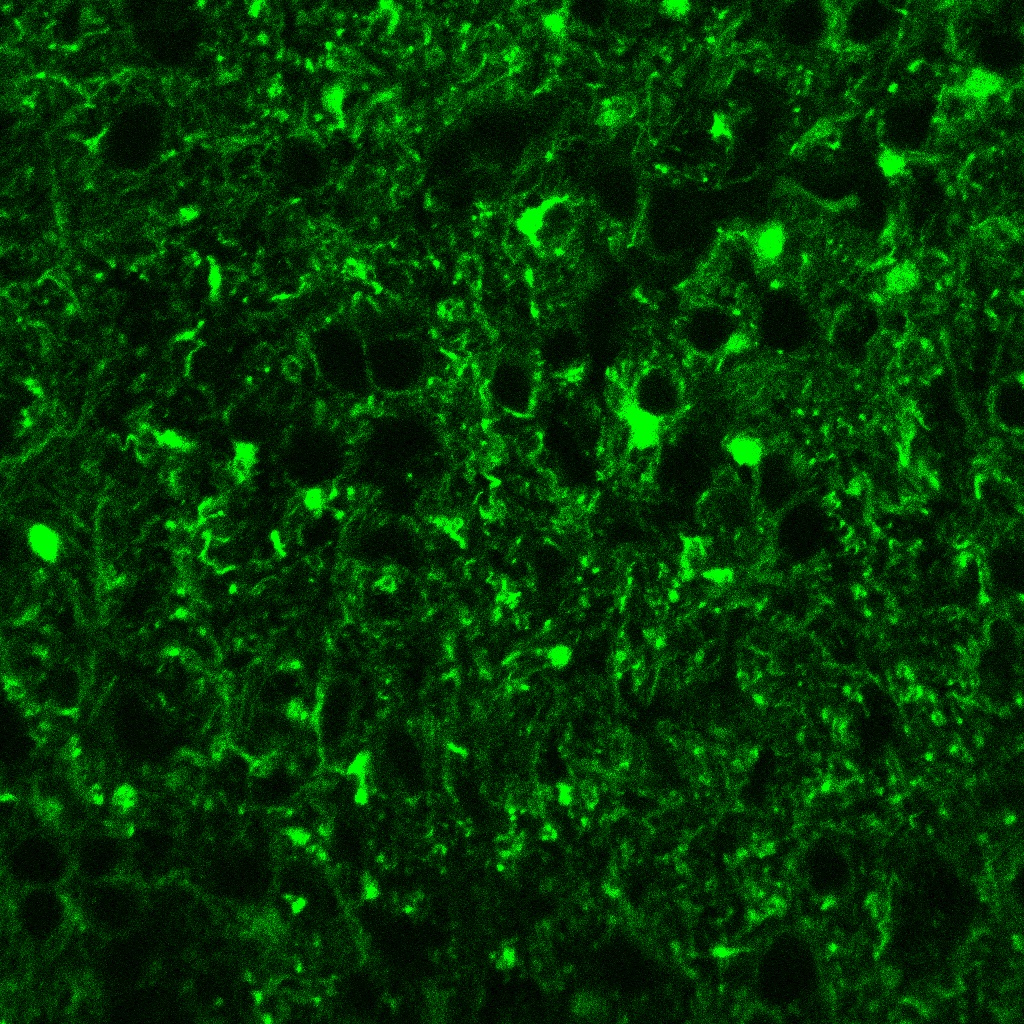

Supplement: Figure 5—source data 1. [file elife-90184-fig5-data1.zip › Figure 5-Source data 1. Raw images (Part 1)/MT dynamics/Zoline-╡Ñ╕÷╬─╝■╡╝│÷-05/Zoline-╡Ñ╕÷╬─╝■╡╝│÷-05_c2.jpg]

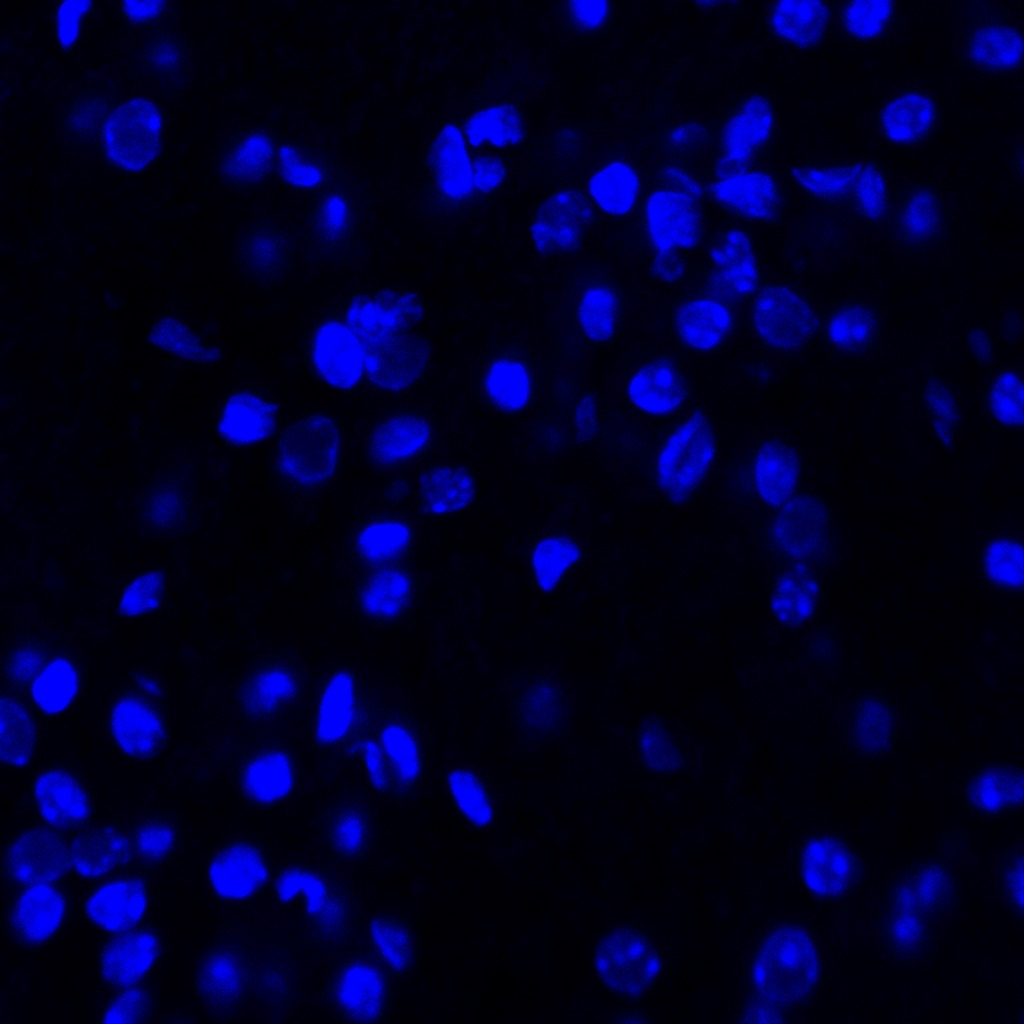

Supplement: Figure 5—source data 1. [file elife-90184-fig5-data1.zip › Figure 5-Source data 1. Raw images (Part 1)/MT dynamics/Zoline-╡Ñ╕÷╬─╝■╡╝│÷-05/Zoline-╡Ñ╕÷╬─╝■╡╝│÷-05_c3.jpg]

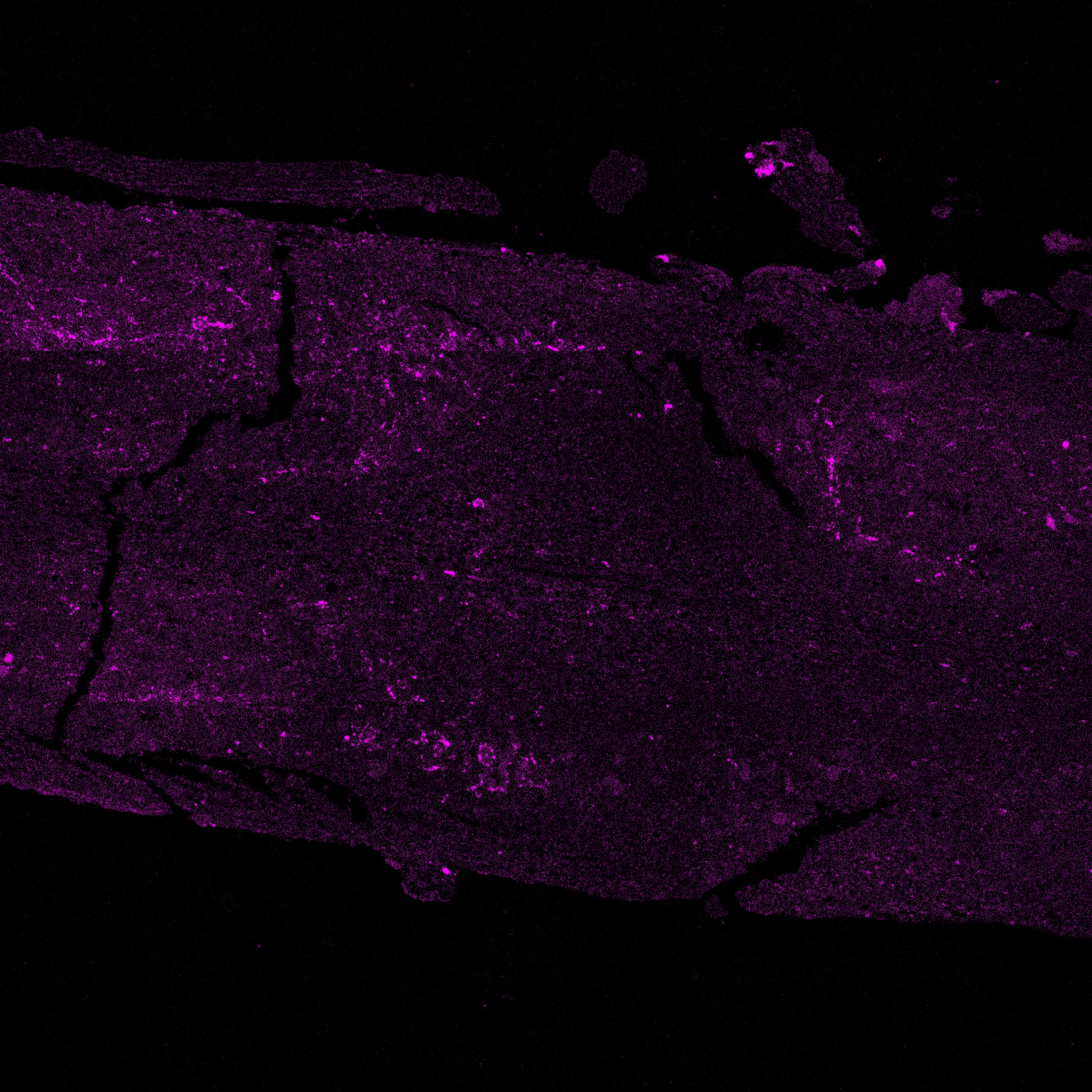

Supplement: Figure 5—source data 2. [file elife-90184-fig5-data2.zip › Figure 5-Source data 2. Raw images (Part 2)/NF GFAP 5-HT stanning/FC/5-HT.jpg]

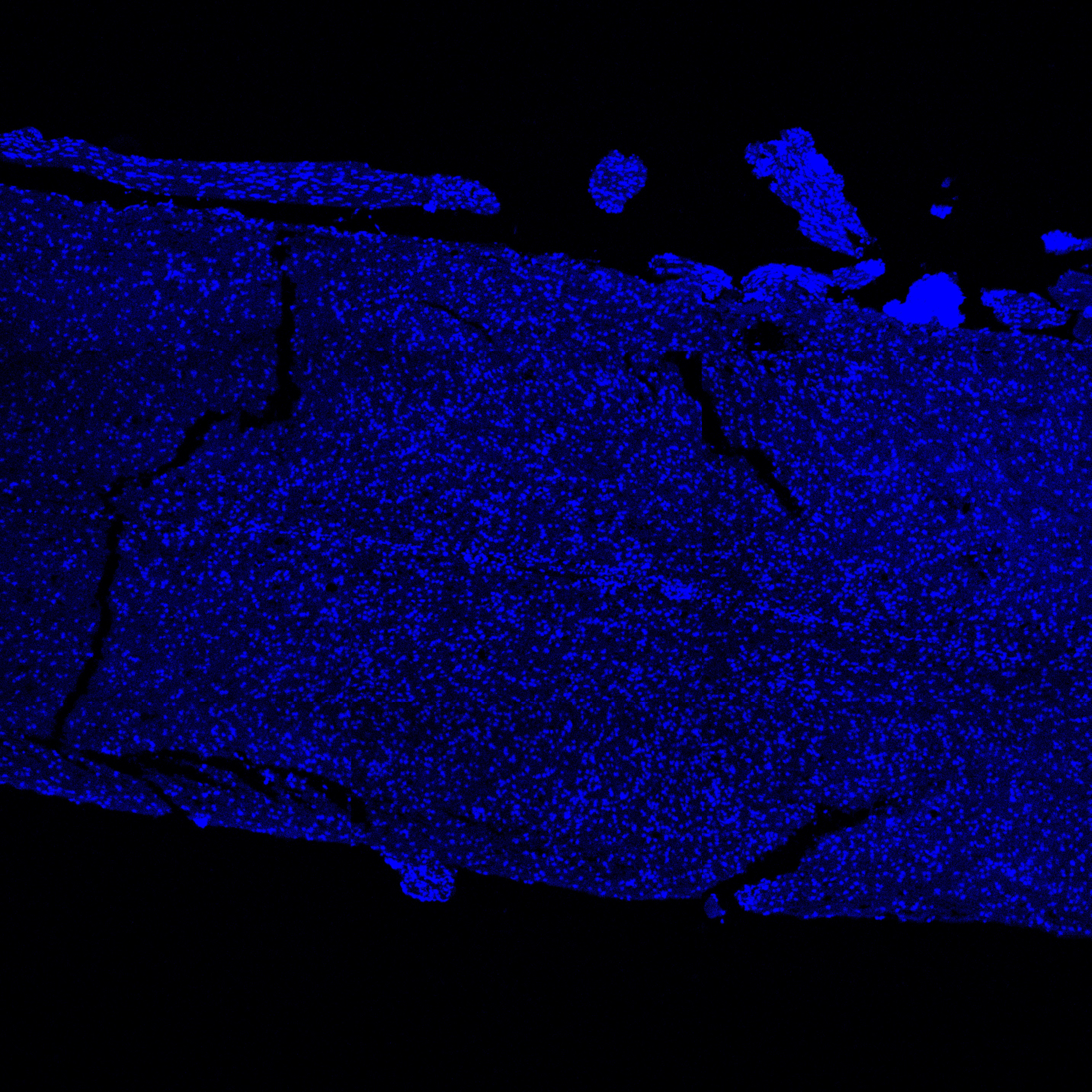

Supplement: Figure 5—source data 2. [file elife-90184-fig5-data2.zip › Figure 5-Source data 2. Raw images (Part 2)/NF GFAP 5-HT stanning/FC/DAPI.jpg]

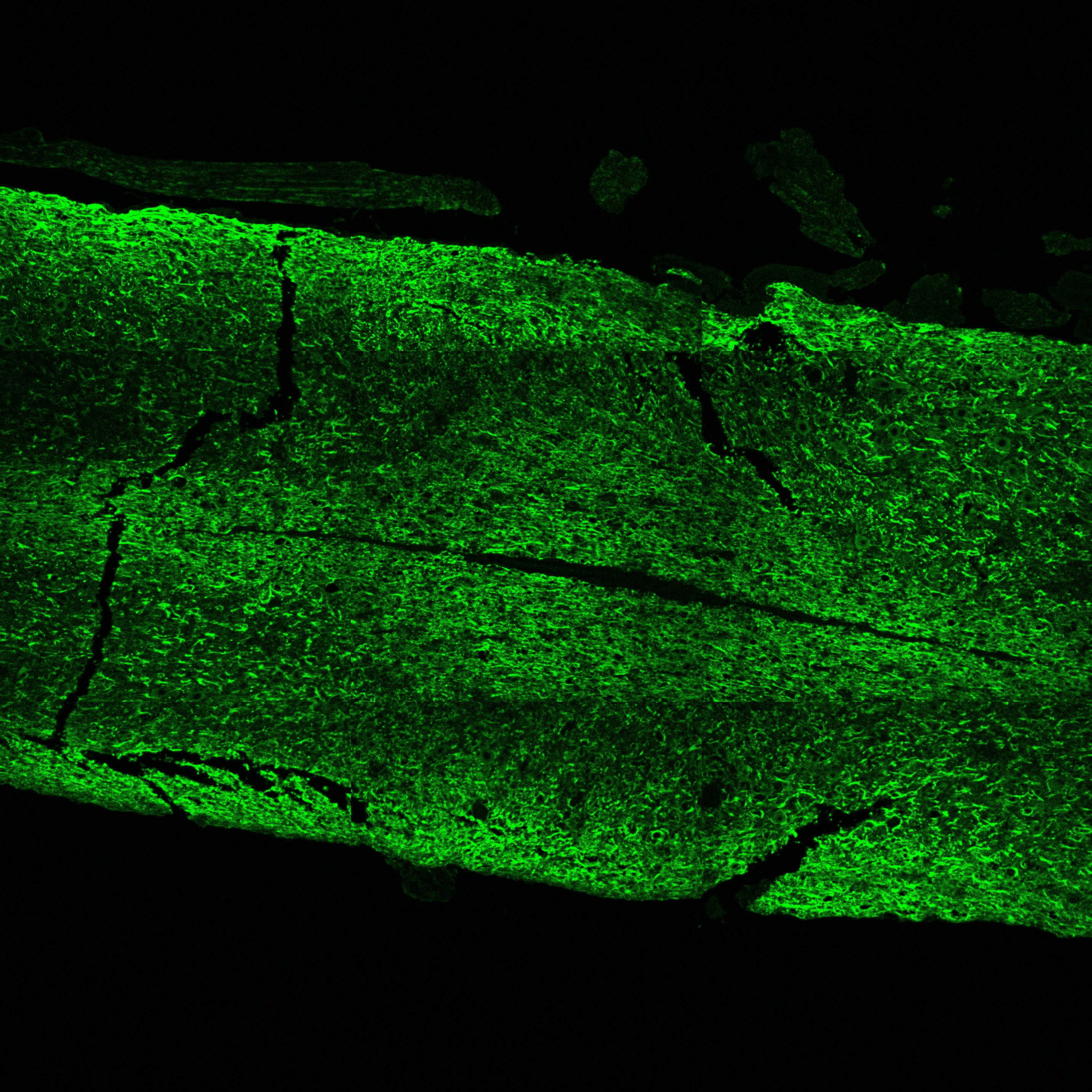

Supplement: Figure 5—source data 2. [file elife-90184-fig5-data2.zip › Figure 5-Source data 2. Raw images (Part 2)/NF GFAP 5-HT stanning/FC/GFAP.jpg]

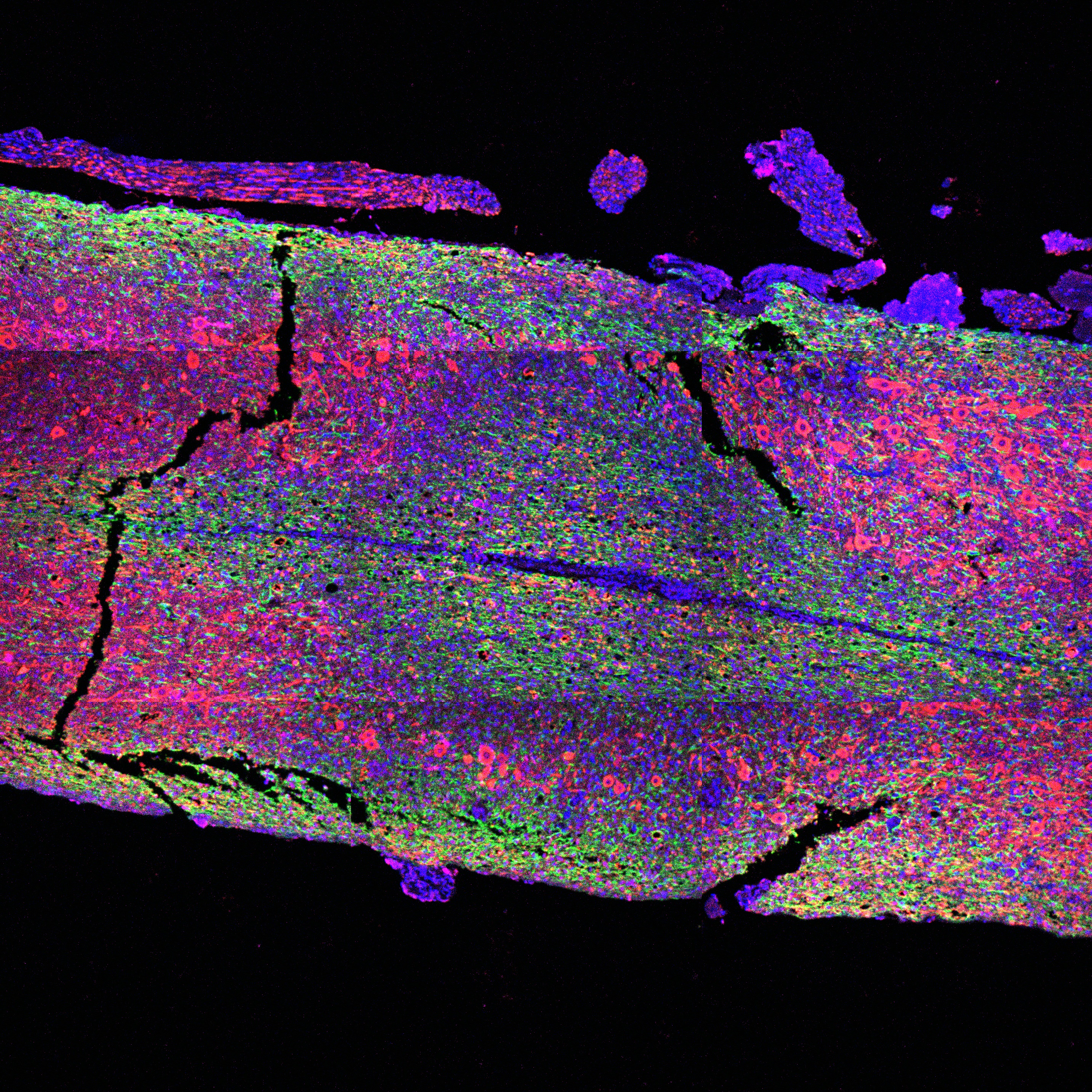

Supplement: Figure 5—source data 2. [file elife-90184-fig5-data2.zip › Figure 5-Source data 2. Raw images (Part 2)/NF GFAP 5-HT stanning/FC/Merge.jpg]

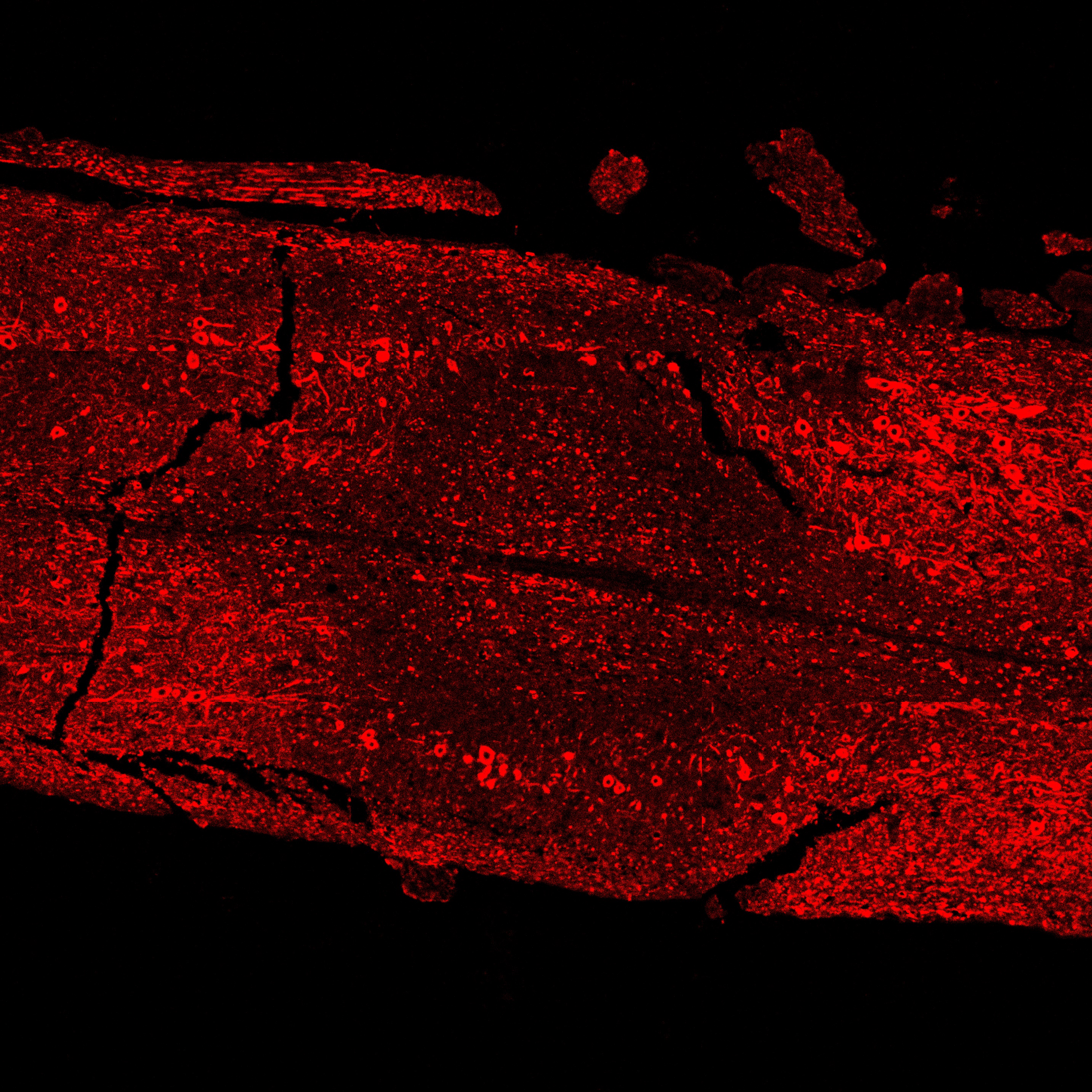

Supplement: Figure 5—source data 2. [file elife-90184-fig5-data2.zip › Figure 5-Source data 2. Raw images (Part 2)/NF GFAP 5-HT stanning/FC/NF.jpg]

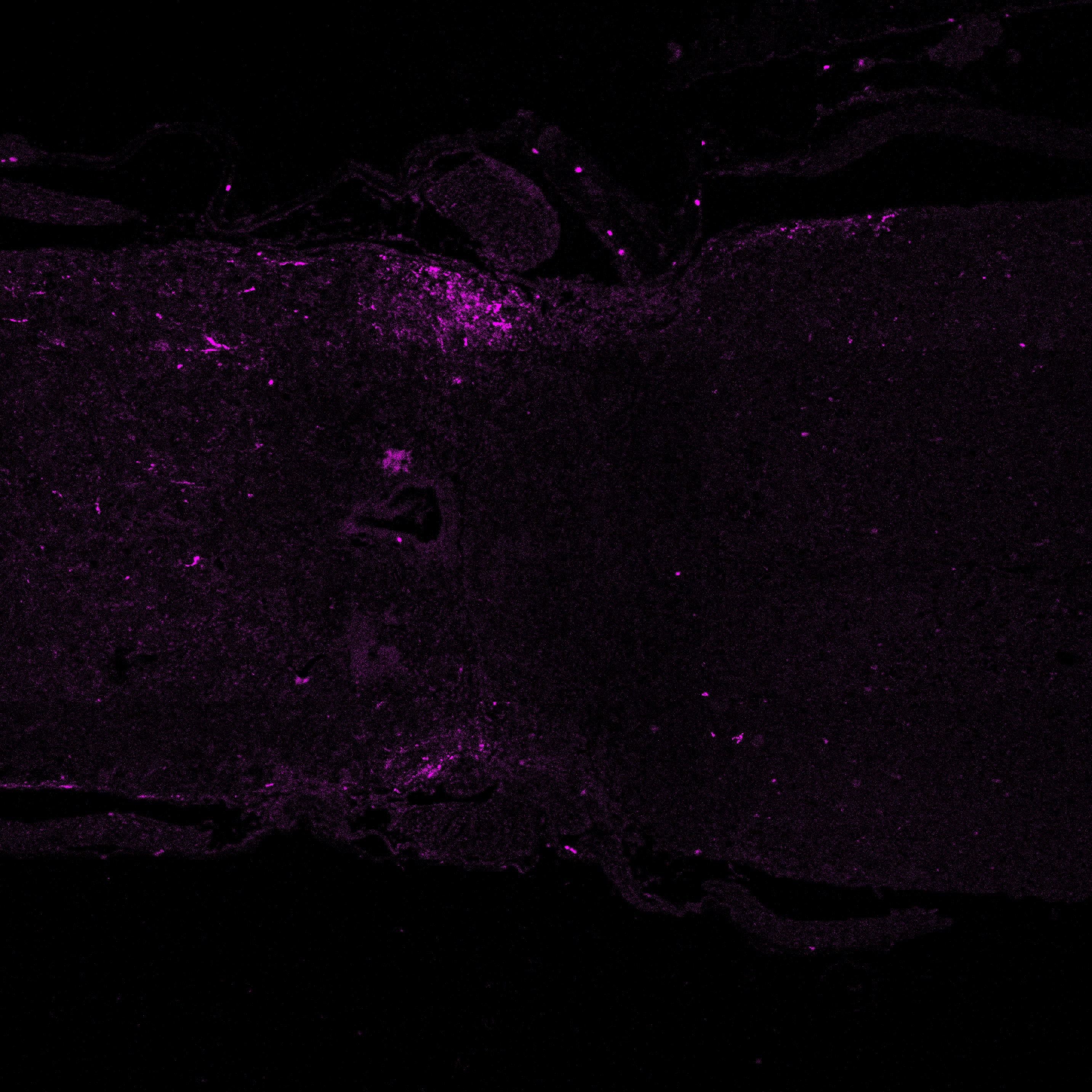

Supplement: Figure 5—source data 2. [file elife-90184-fig5-data2.zip › Figure 5-Source data 2. Raw images (Part 2)/NF GFAP 5-HT stanning/FC+zoline/5-HT.jpg]

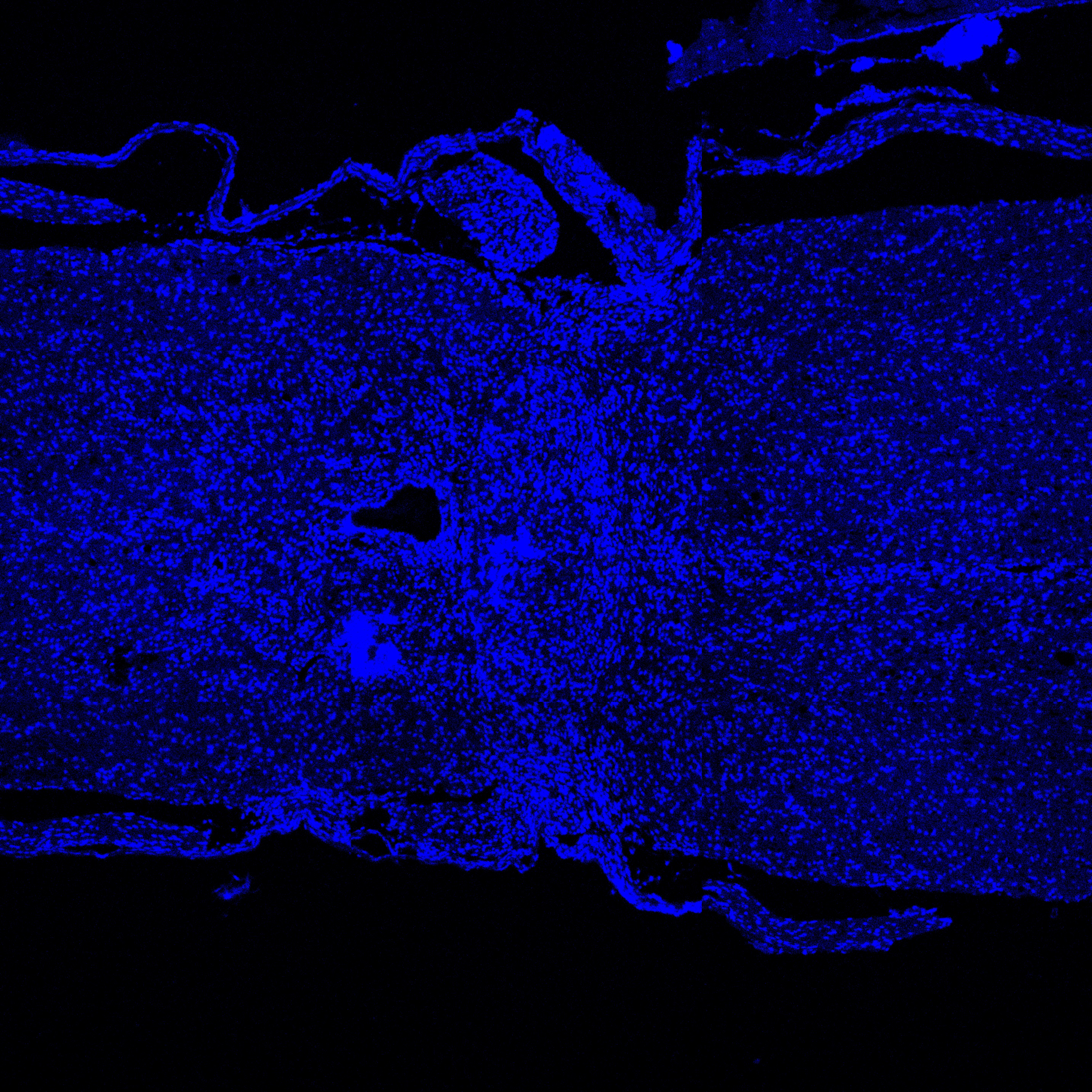

Supplement: Figure 5—source data 2. [file elife-90184-fig5-data2.zip › Figure 5-Source data 2. Raw images (Part 2)/NF GFAP 5-HT stanning/FC+zoline/DAPI.jpg]

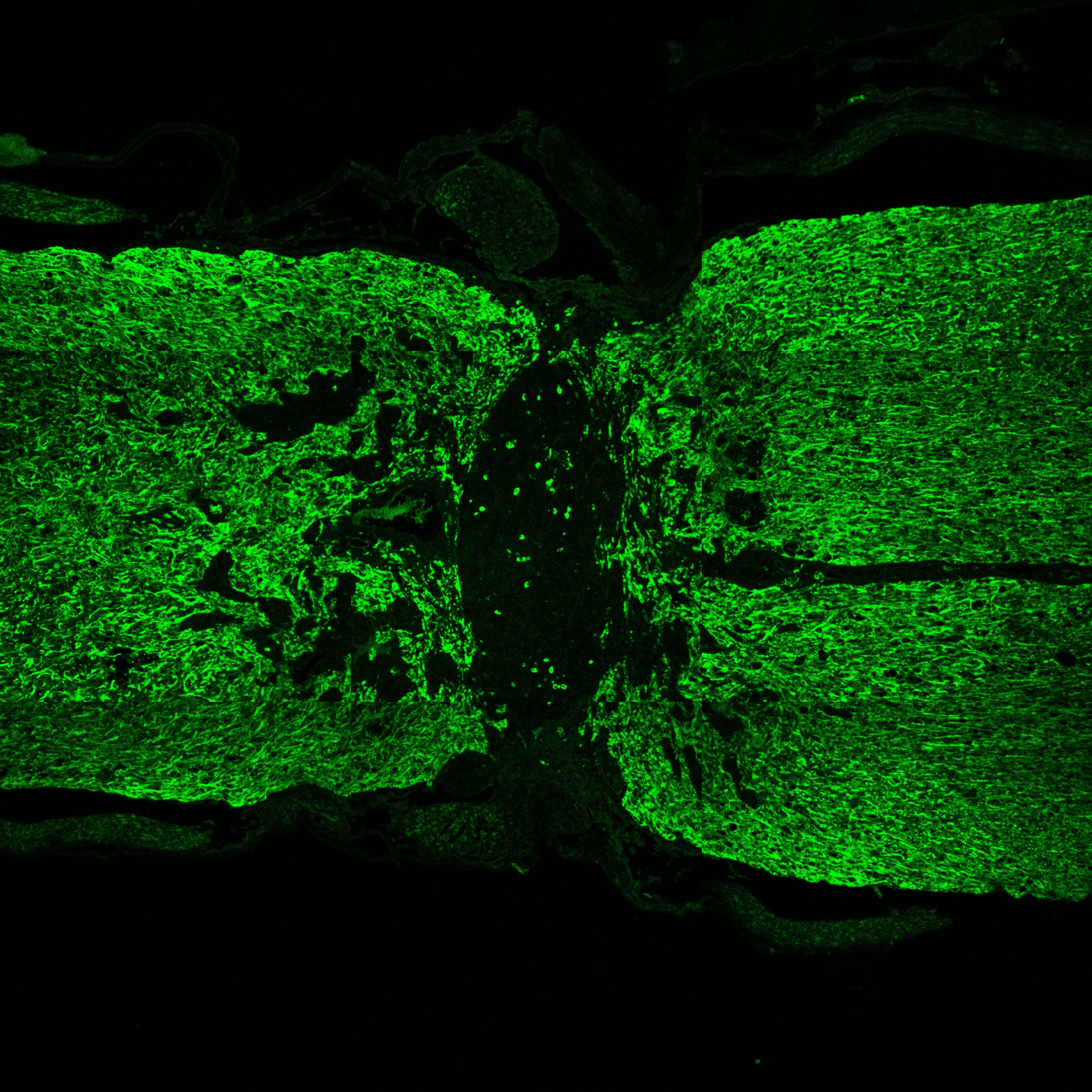

Supplement: Figure 5—source data 2. [file elife-90184-fig5-data2.zip › Figure 5-Source data 2. Raw images (Part 2)/NF GFAP 5-HT stanning/FC+zoline/GFAP.jpg]

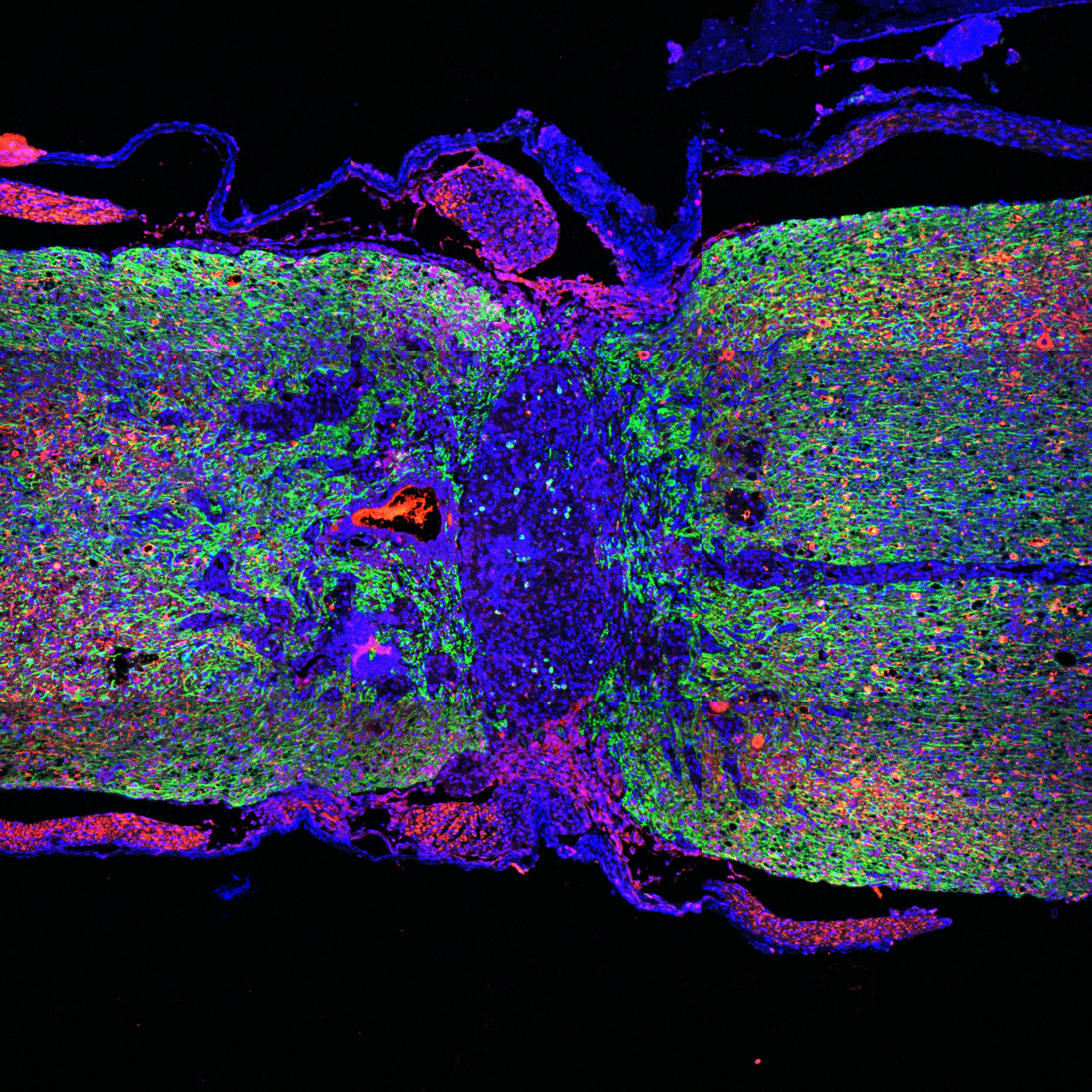

Supplement: Figure 5—source data 2. [file elife-90184-fig5-data2.zip › Figure 5-Source data 2. Raw images (Part 2)/NF GFAP 5-HT stanning/FC+zoline/Merge.jpg]

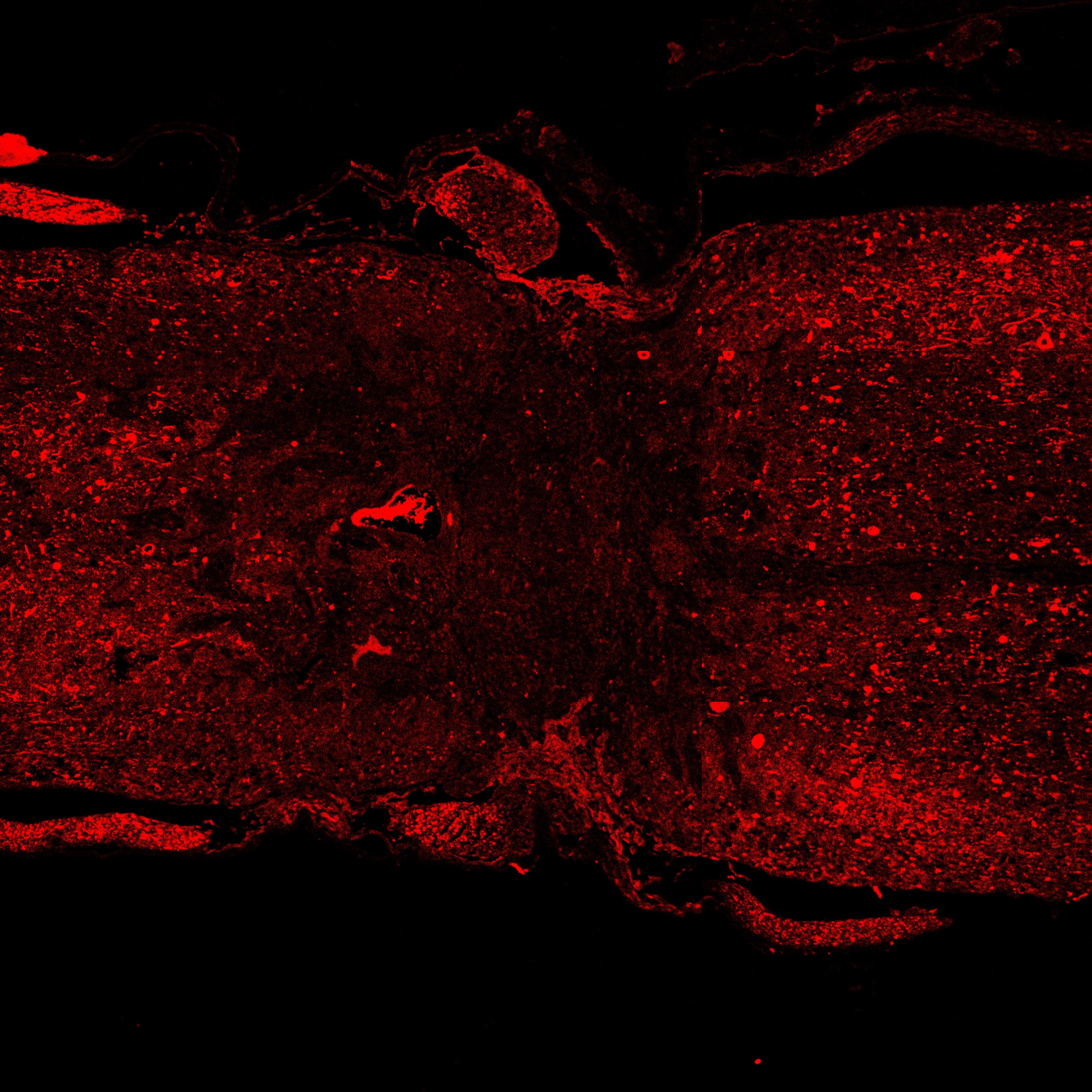

Supplement: Figure 5—source data 2. [file elife-90184-fig5-data2.zip › Figure 5-Source data 2. Raw images (Part 2)/NF GFAP 5-HT stanning/FC+zoline/NF.jpg]

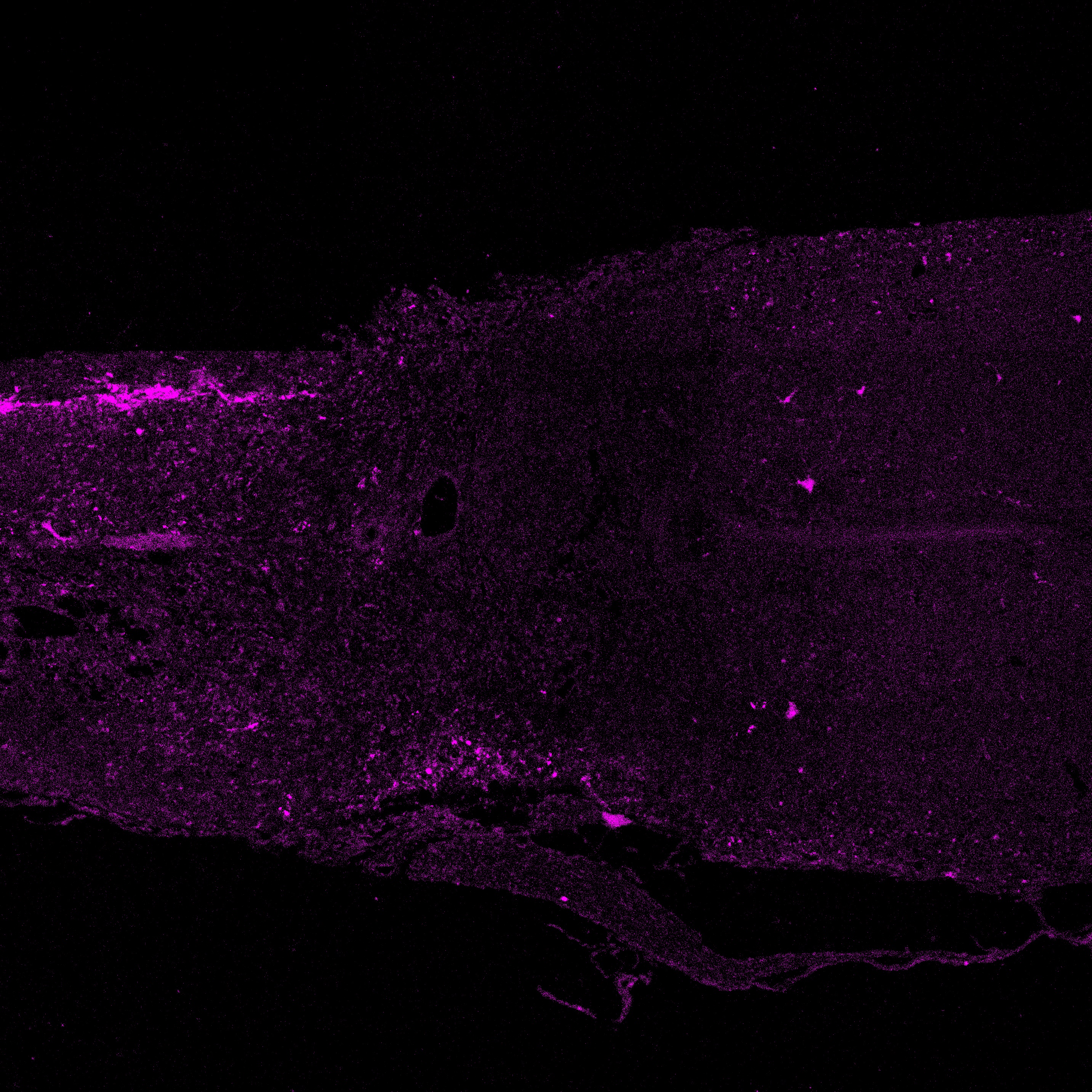

Supplement: Figure 5—source data 2. [file elife-90184-fig5-data2.zip › Figure 5-Source data 2. Raw images (Part 2)/NF GFAP 5-HT stanning/injury/5-HT.jpg]

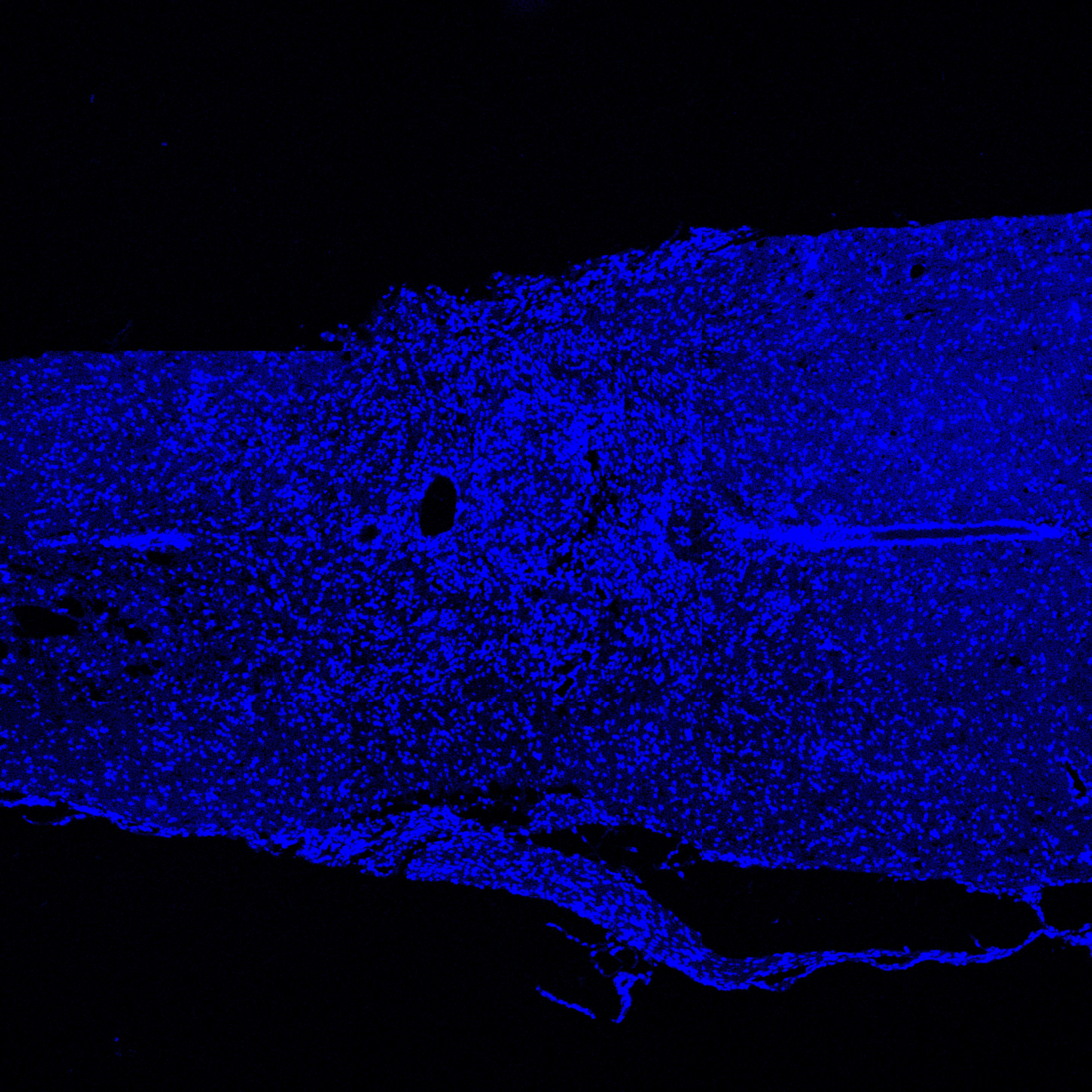

Supplement: Figure 5—source data 2. [file elife-90184-fig5-data2.zip › Figure 5-Source data 2. Raw images (Part 2)/NF GFAP 5-HT stanning/injury/DAPI.jpg]

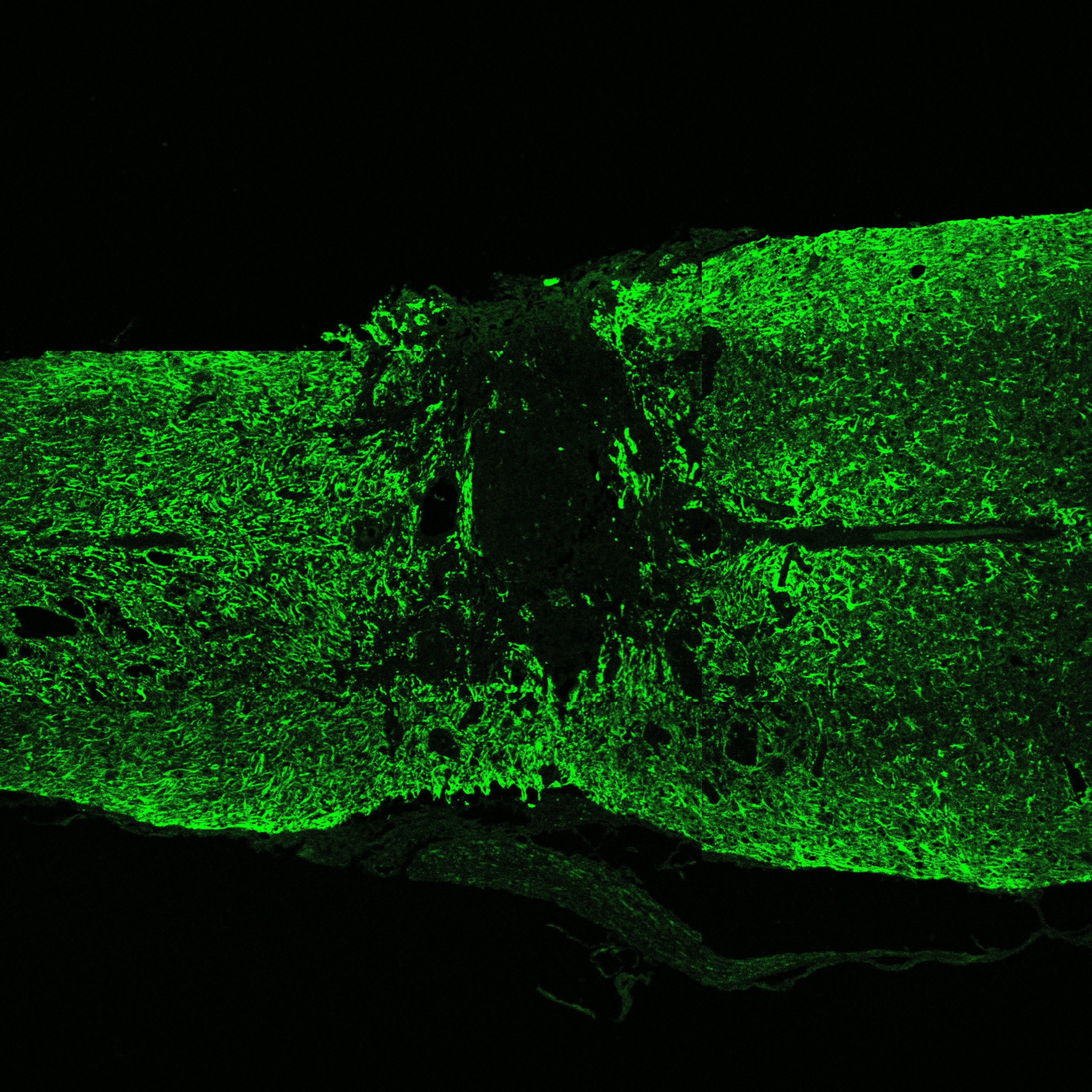

Supplement: Figure 5—source data 2. [file elife-90184-fig5-data2.zip › Figure 5-Source data 2. Raw images (Part 2)/NF GFAP 5-HT stanning/injury/GFAP.jpg]

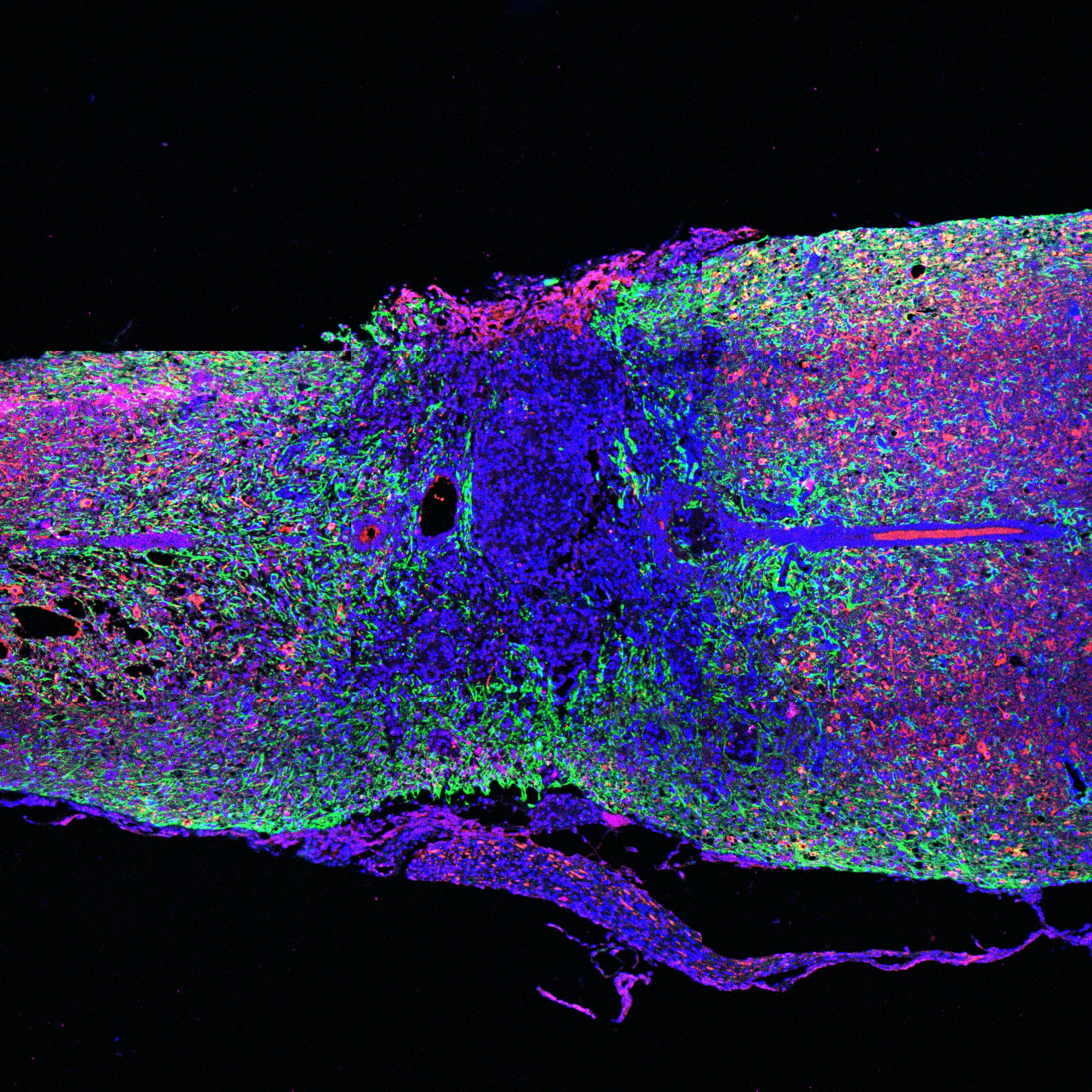

Supplement: Figure 5—source data 2. [file elife-90184-fig5-data2.zip › Figure 5-Source data 2. Raw images (Part 2)/NF GFAP 5-HT stanning/injury/Merge.jpg]

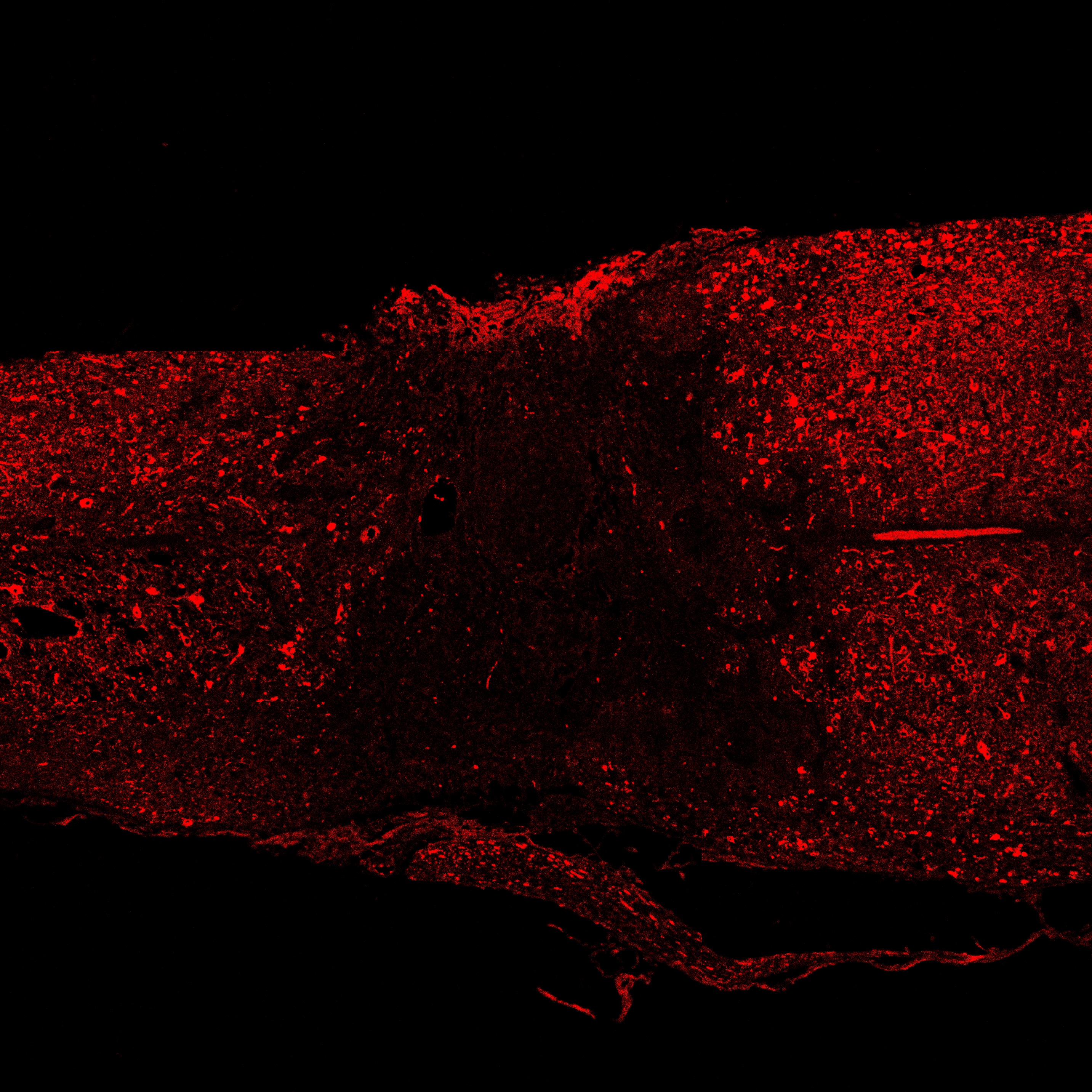

Supplement: Figure 5—source data 2. [file elife-90184-fig5-data2.zip › Figure 5-Source data 2. Raw images (Part 2)/NF GFAP 5-HT stanning/injury/NF.jpg]

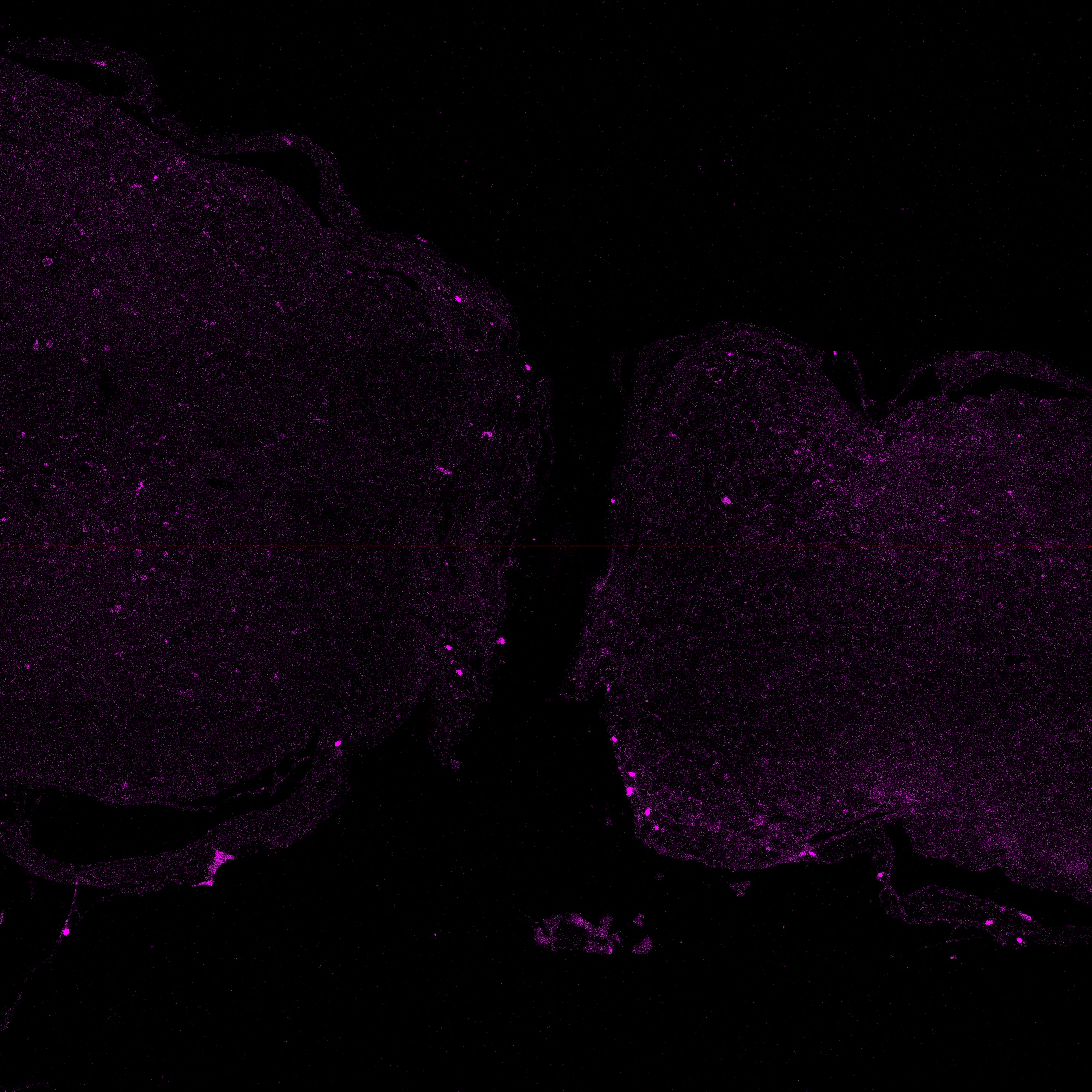

Supplement: Figure 5—source data 2. [file elife-90184-fig5-data2.zip › Figure 5-Source data 2. Raw images (Part 2)/NF GFAP 5-HT stanning/zoline/5-HT.jpg]

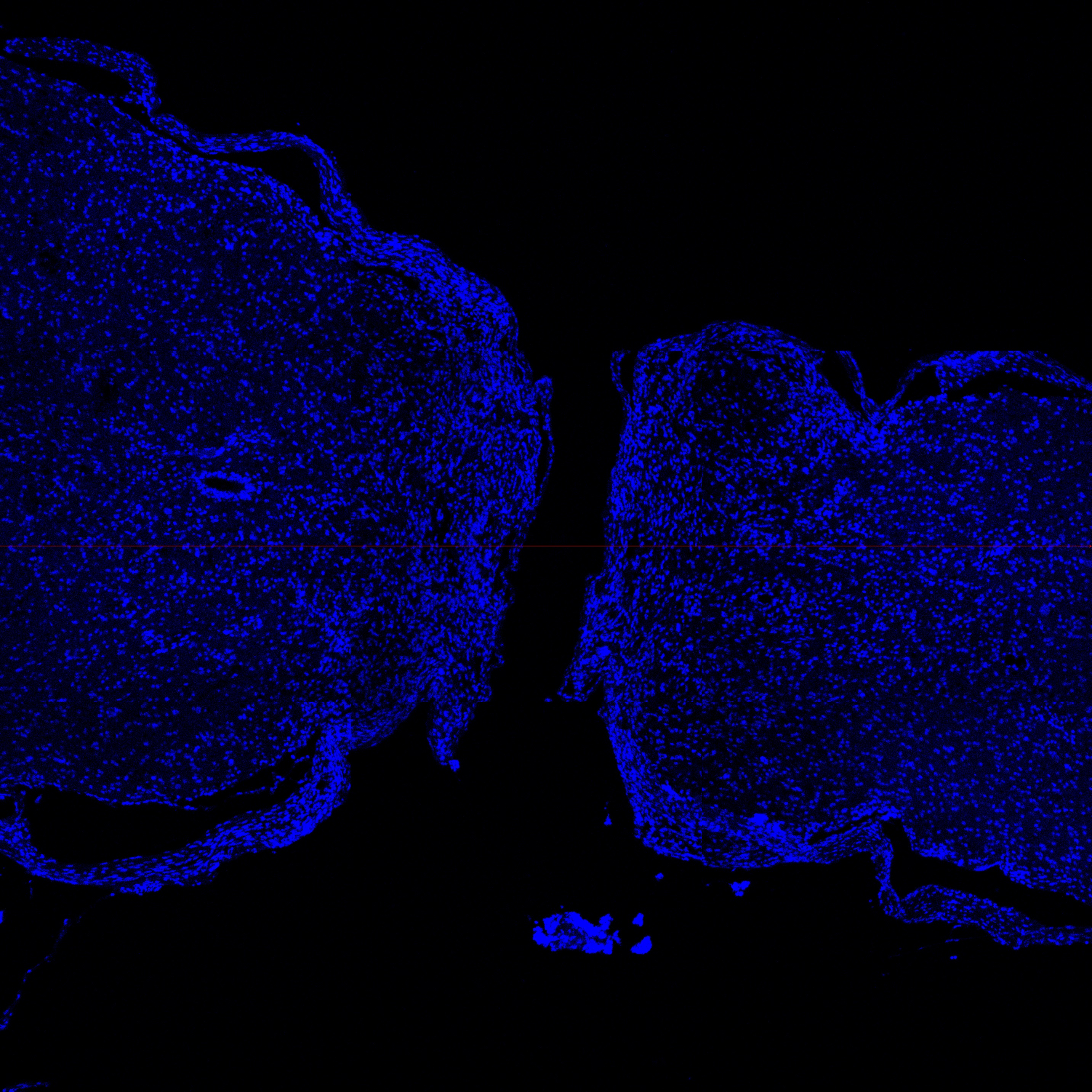

Supplement: Figure 5—source data 2. [file elife-90184-fig5-data2.zip › Figure 5-Source data 2. Raw images (Part 2)/NF GFAP 5-HT stanning/zoline/DAPI.jpg]

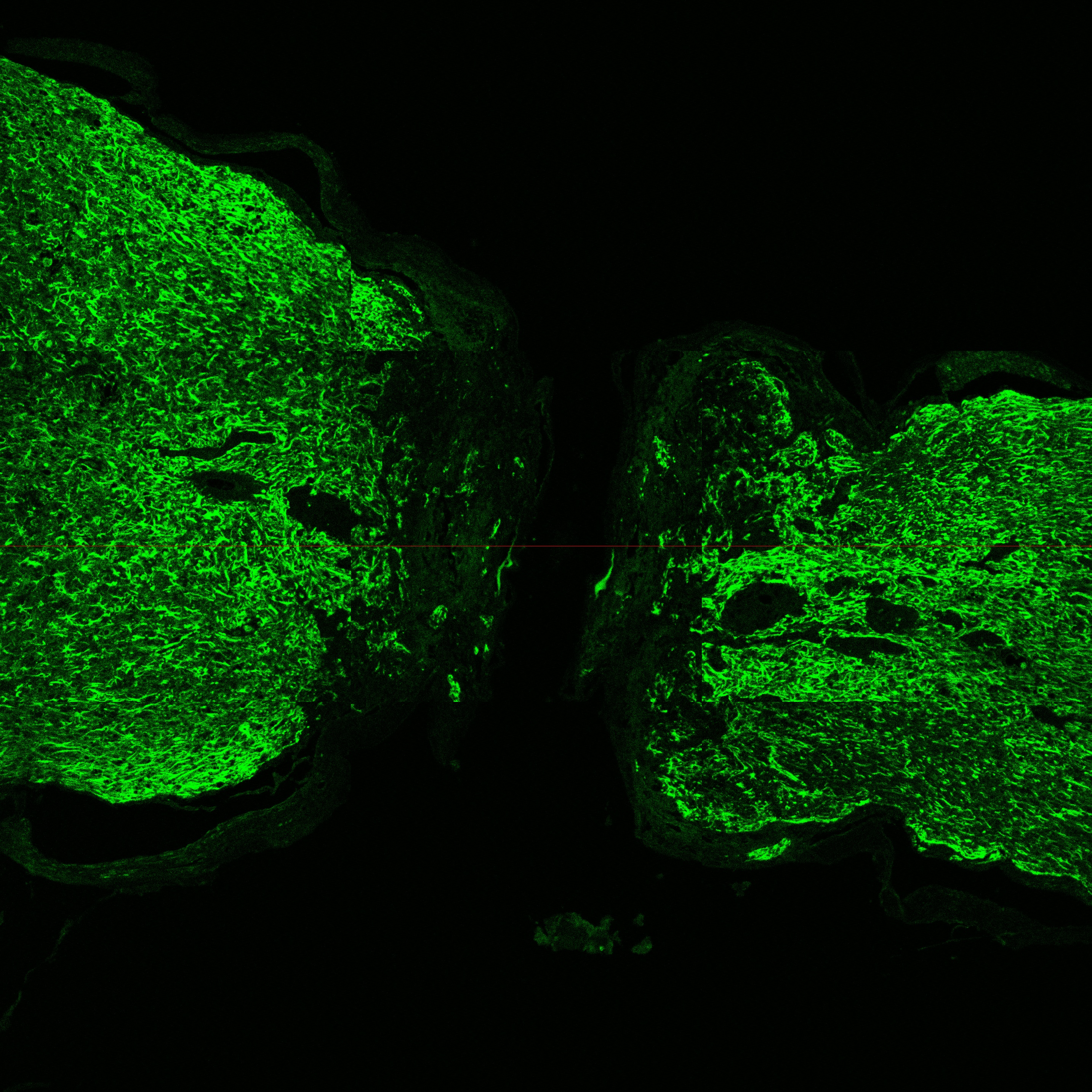

Supplement: Figure 5—source data 2. [file elife-90184-fig5-data2.zip › Figure 5-Source data 2. Raw images (Part 2)/NF GFAP 5-HT stanning/zoline/GFAP.jpg]

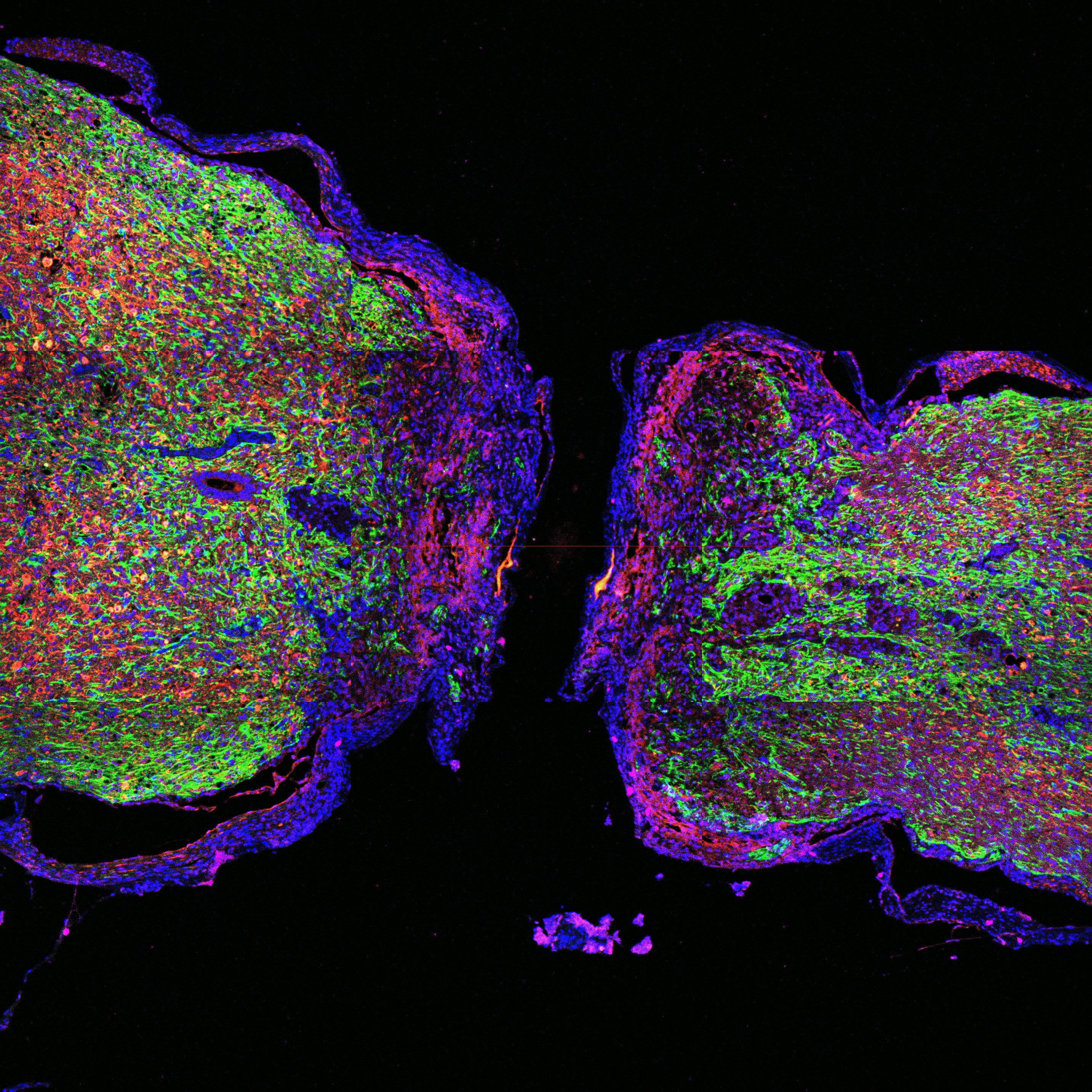

Supplement: Figure 5—source data 2. [file elife-90184-fig5-data2.zip › Figure 5-Source data 2. Raw images (Part 2)/NF GFAP 5-HT stanning/zoline/Merge.jpg]

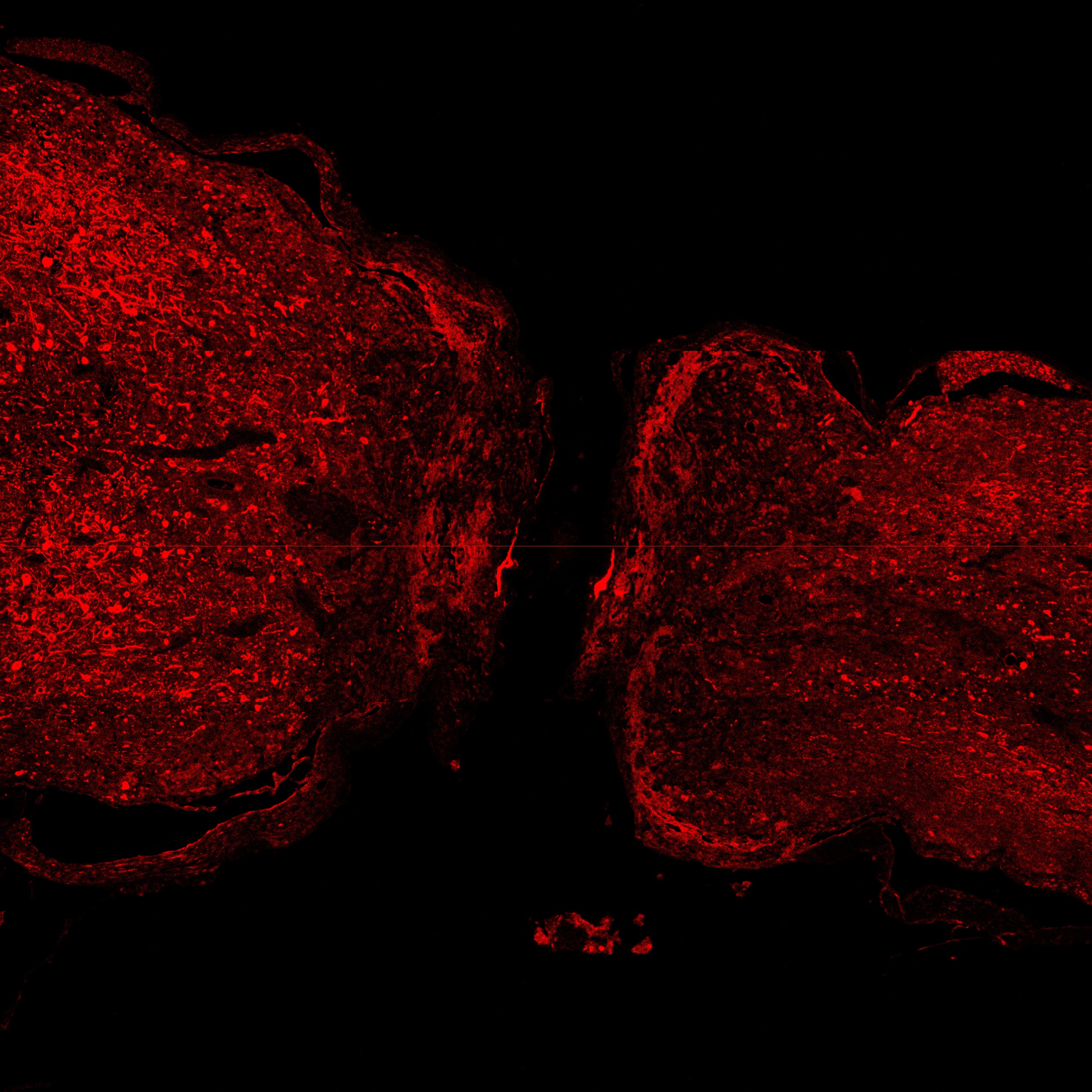

Supplement: Figure 5—source data 2. [file elife-90184-fig5-data2.zip › Figure 5-Source data 2. Raw images (Part 2)/NF GFAP 5-HT stanning/zoline/NF.jpg]
