# Supplementary figures and images for: 14-3-3 protein augments the protein stability of phosphorylated spastin and promotes the recovery of spinal cord injury through its agonist intervention (part 2 of 2)
Source: eLife. 2024 Jan 17;12:RP90184. doi: 10.7554/eLife.90184 (PMC10945579; doi:10.7554/eLife.90184)

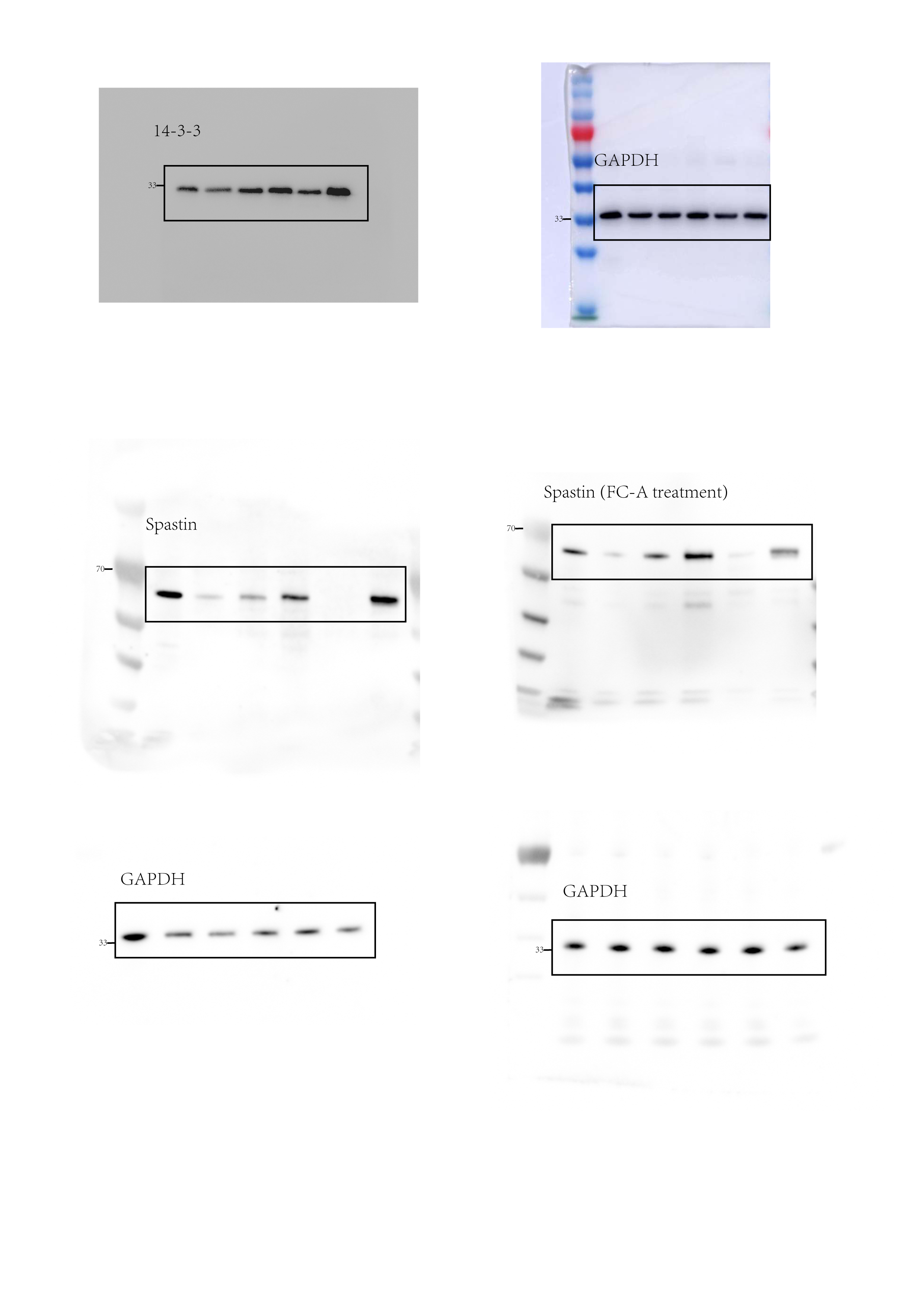

Supplement: Figure 5—source data 3. [file elife-90184-fig5-data3.zip › Figure 5-Source data 3. Raw and annotated blots for Figure 5/Annotated blots.tif]

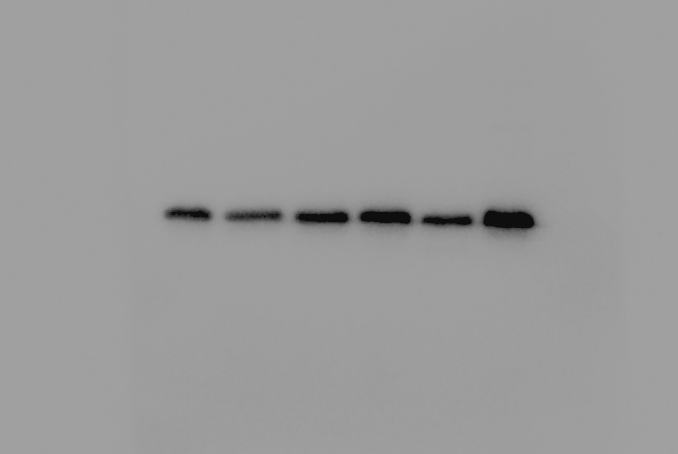

Supplement: Figure 5—source data 3. [file elife-90184-fig5-data3.zip › Figure 5-Source data 3. Raw and annotated blots for Figure 5/Raw blots/14-3-3▒φ┤∩╟Θ┐÷.tif]

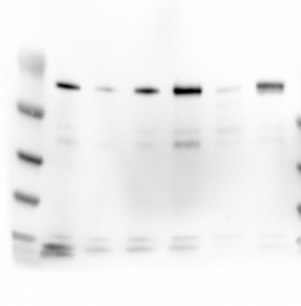

Supplement: Figure 5—source data 3. [file elife-90184-fig5-data3.zip › Figure 5-Source data 3. Raw and annotated blots for Figure 5/Raw blots/FC A ║≤spastin▒φ┤∩╟Θ┐÷.tif]

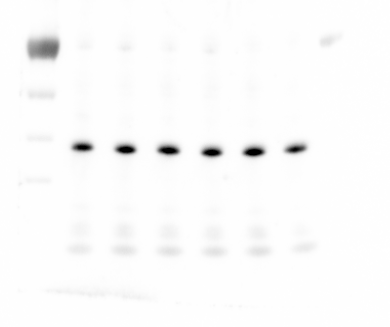

Supplement: Figure 5—source data 3. [file elife-90184-fig5-data3.zip › Figure 5-Source data 3. Raw and annotated blots for Figure 5/Raw blots/FCA╫Θ╡─GAPDH▒φ┤∩╟Θ┐÷.tif]

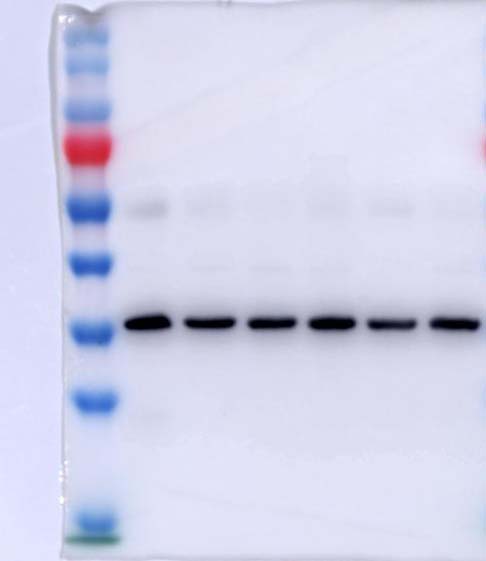

Supplement: Figure 5—source data 3. [file elife-90184-fig5-data3.zip › Figure 5-Source data 3. Raw and annotated blots for Figure 5/Raw blots/GAPDH.jpg]

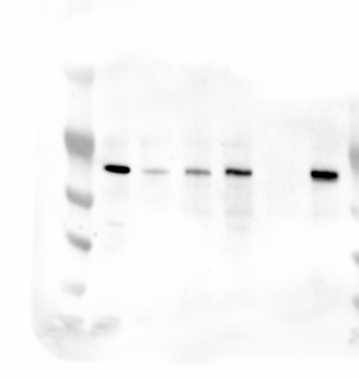

Supplement: Figure 5—source data 3. [file elife-90184-fig5-data3.zip › Figure 5-Source data 3. Raw and annotated blots for Figure 5/Raw blots/spastin▒φ┤∩╟Θ┐÷.tif]

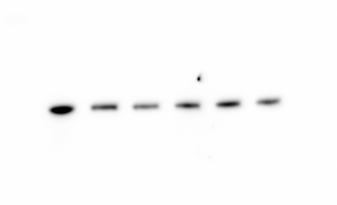

Supplement: Figure 5—source data 3. [file elife-90184-fig5-data3.zip › Figure 5-Source data 3. Raw and annotated blots for Figure 5/Raw blots/╦≡╔╦╫Θspastin▒φ┤∩╡─GAPDH.tif]

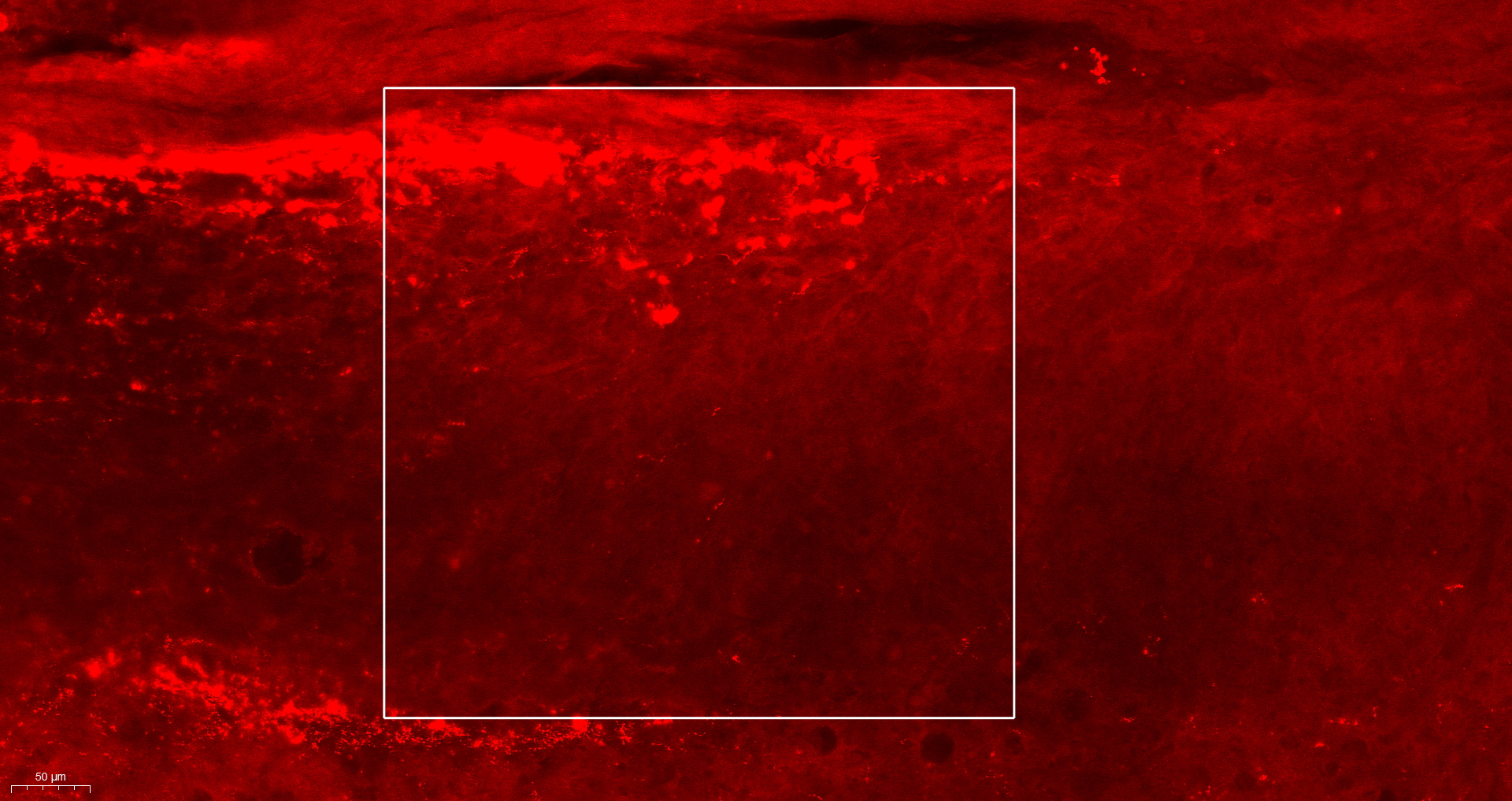

Supplement: Figure 7—source data 1. [file elife-90184-fig7-data1.zip › Figure 7-Source data 1. Raw Images for Figure 7/5-HT/Boxed selected for llustration/1.1.jpg]

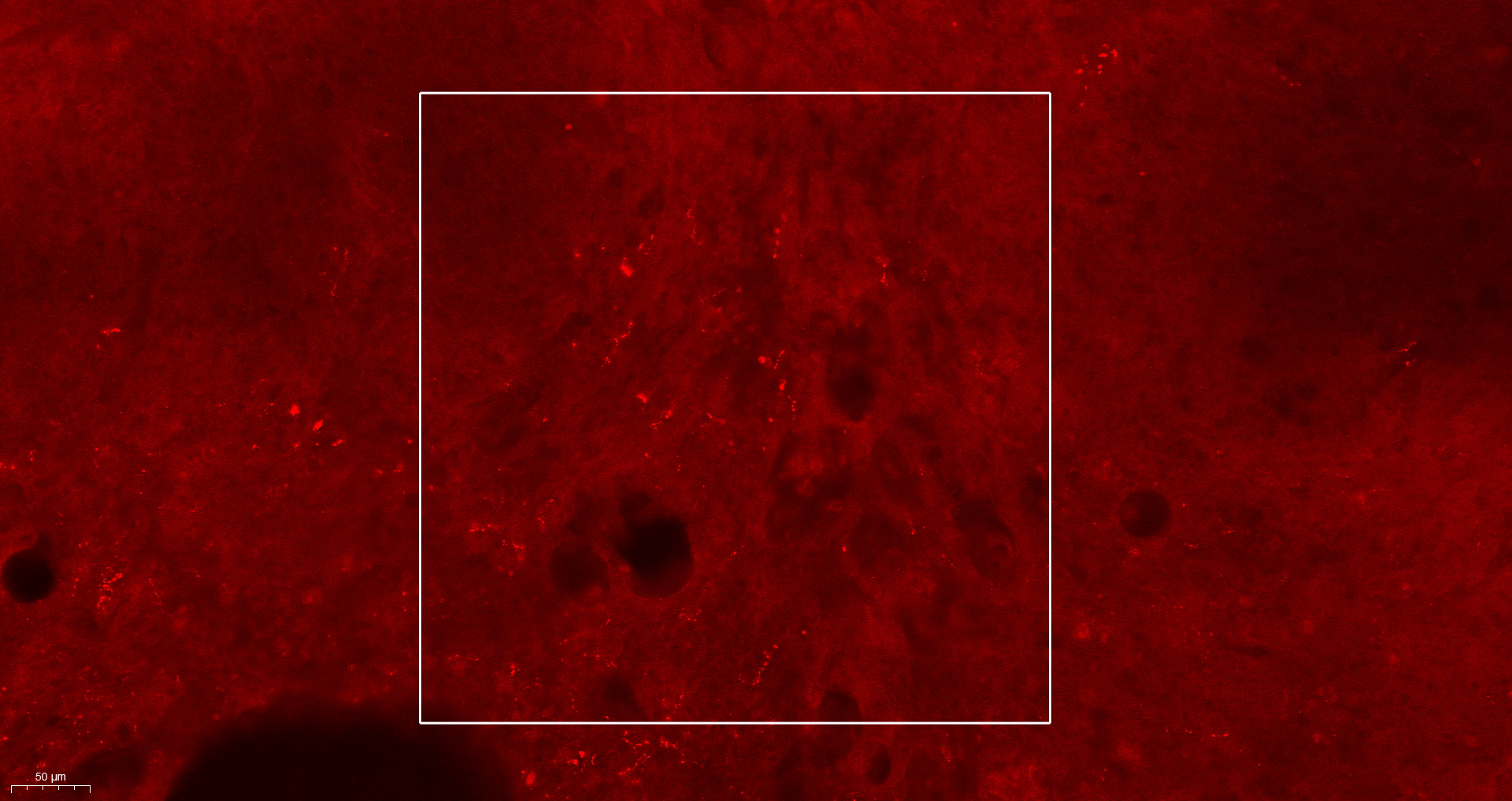

Supplement: Figure 7—source data 1. [file elife-90184-fig7-data1.zip › Figure 7-Source data 1. Raw Images for Figure 7/5-HT/Boxed selected for llustration/1.2.jpg]

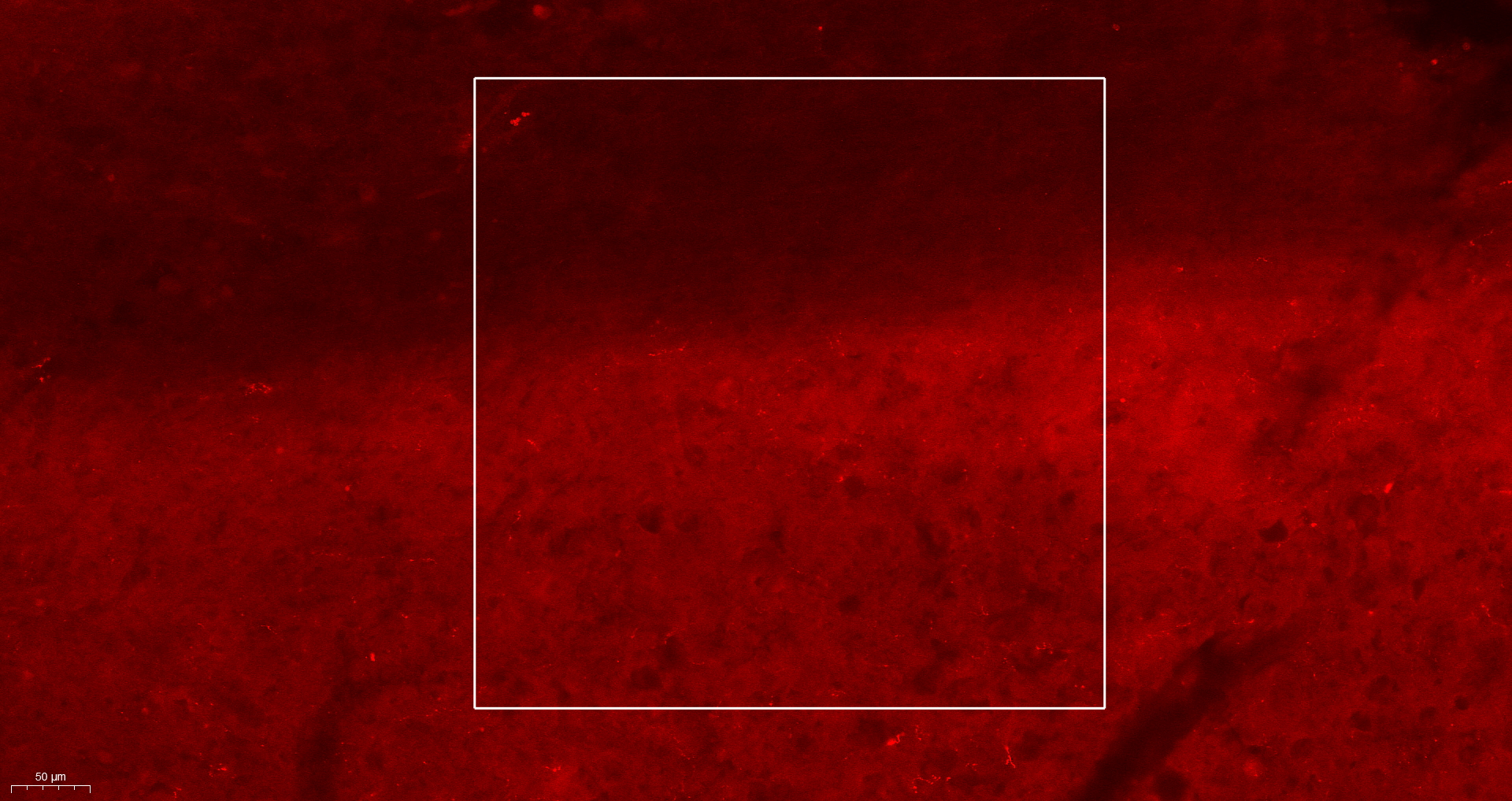

Supplement: Figure 7—source data 1. [file elife-90184-fig7-data1.zip › Figure 7-Source data 1. Raw Images for Figure 7/5-HT/Boxed selected for llustration/1.3.jpg]

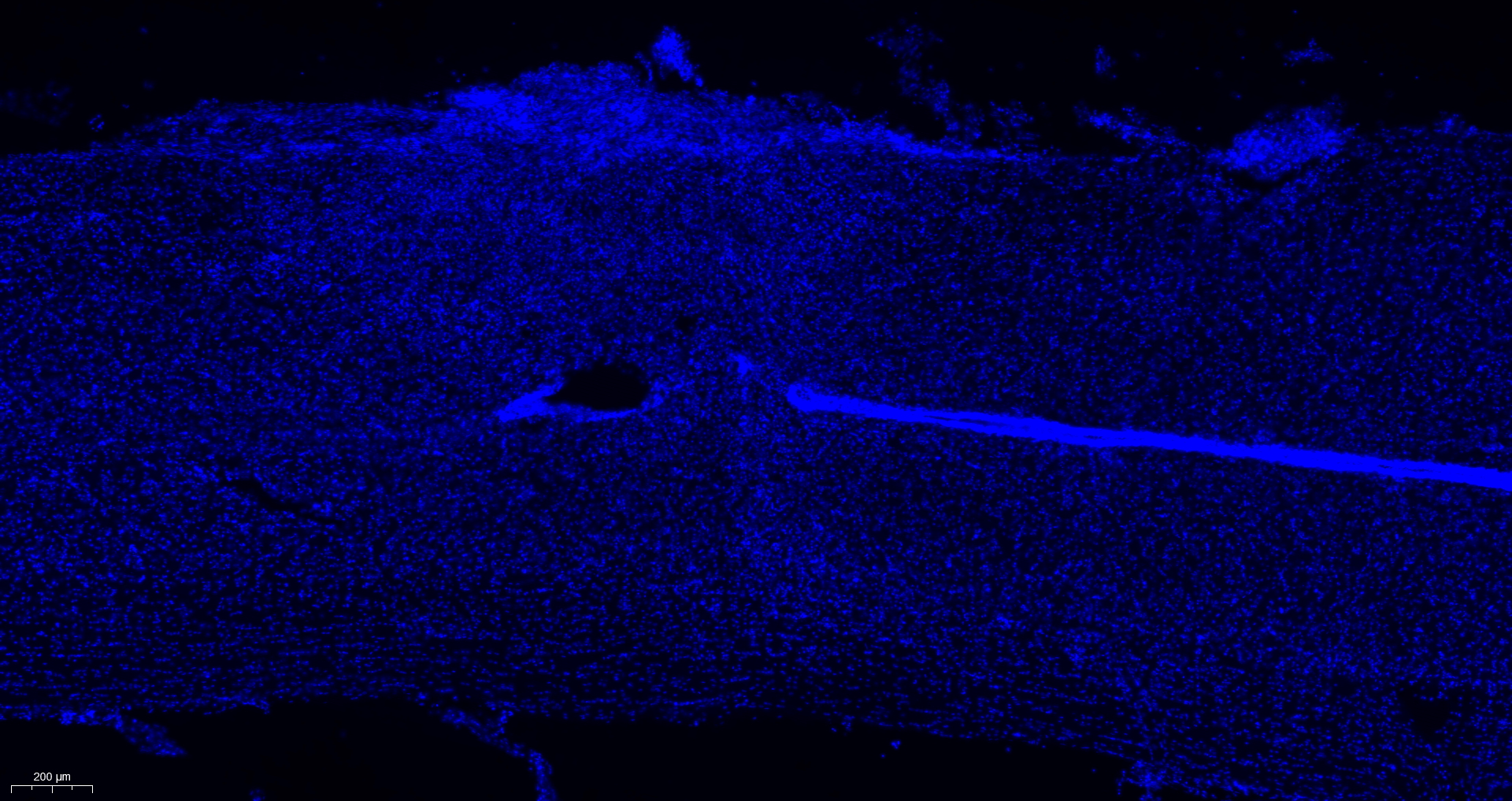

Supplement: Figure 7—source data 1. [file elife-90184-fig7-data1.zip › Figure 7-Source data 1. Raw Images for Figure 7/5-HT/Boxed selected for llustration/1.jpg]

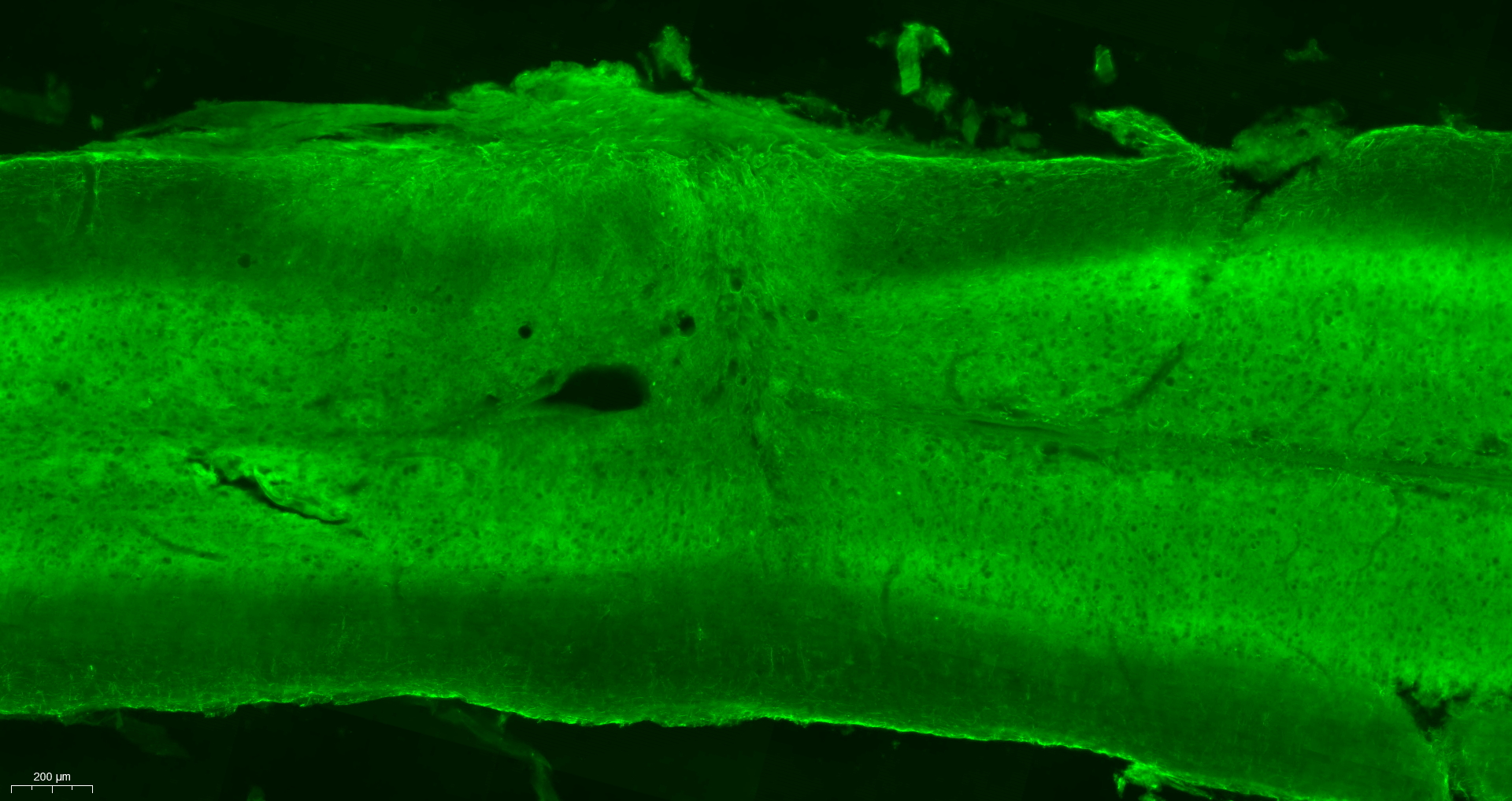

Supplement: Figure 7—source data 1. [file elife-90184-fig7-data1.zip › Figure 7-Source data 1. Raw Images for Figure 7/5-HT/Boxed selected for llustration/2.jpg]

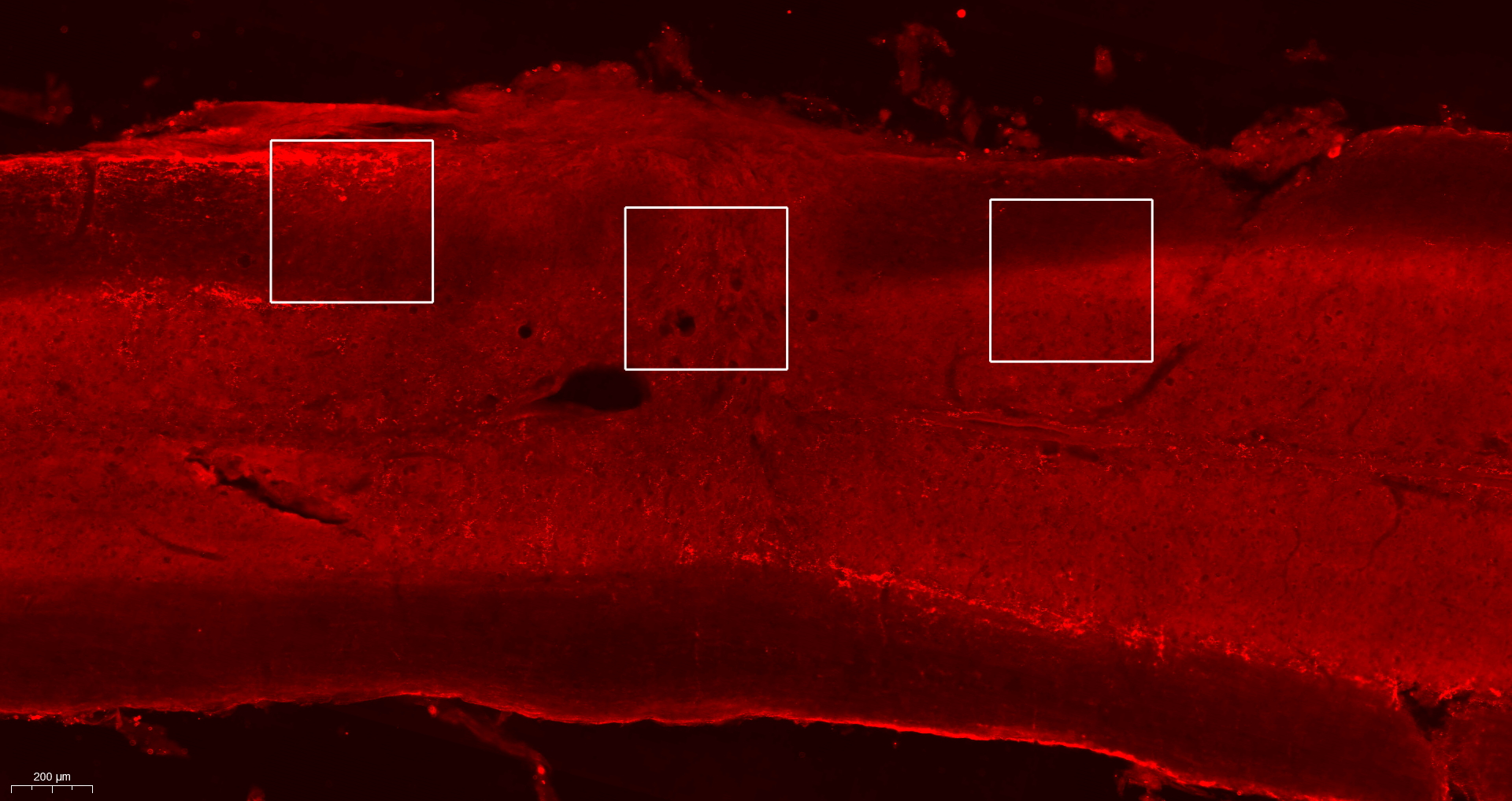

Supplement: Figure 7—source data 1. [file elife-90184-fig7-data1.zip › Figure 7-Source data 1. Raw Images for Figure 7/5-HT/Boxed selected for llustration/3.jpg]

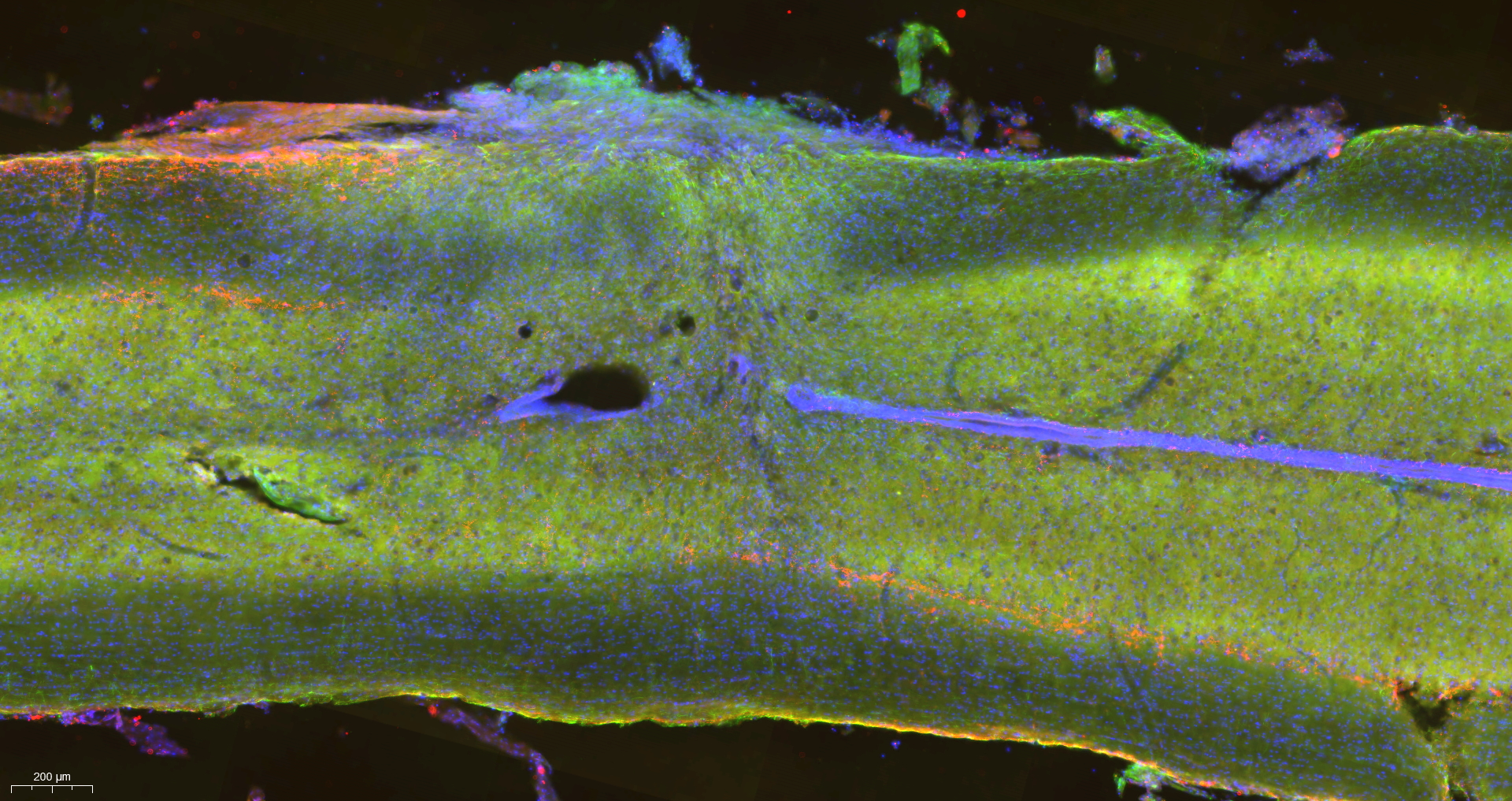

Supplement: Figure 7—source data 1. [file elife-90184-fig7-data1.zip › Figure 7-Source data 1. Raw Images for Figure 7/5-HT/Boxed selected for llustration/4.jpg]

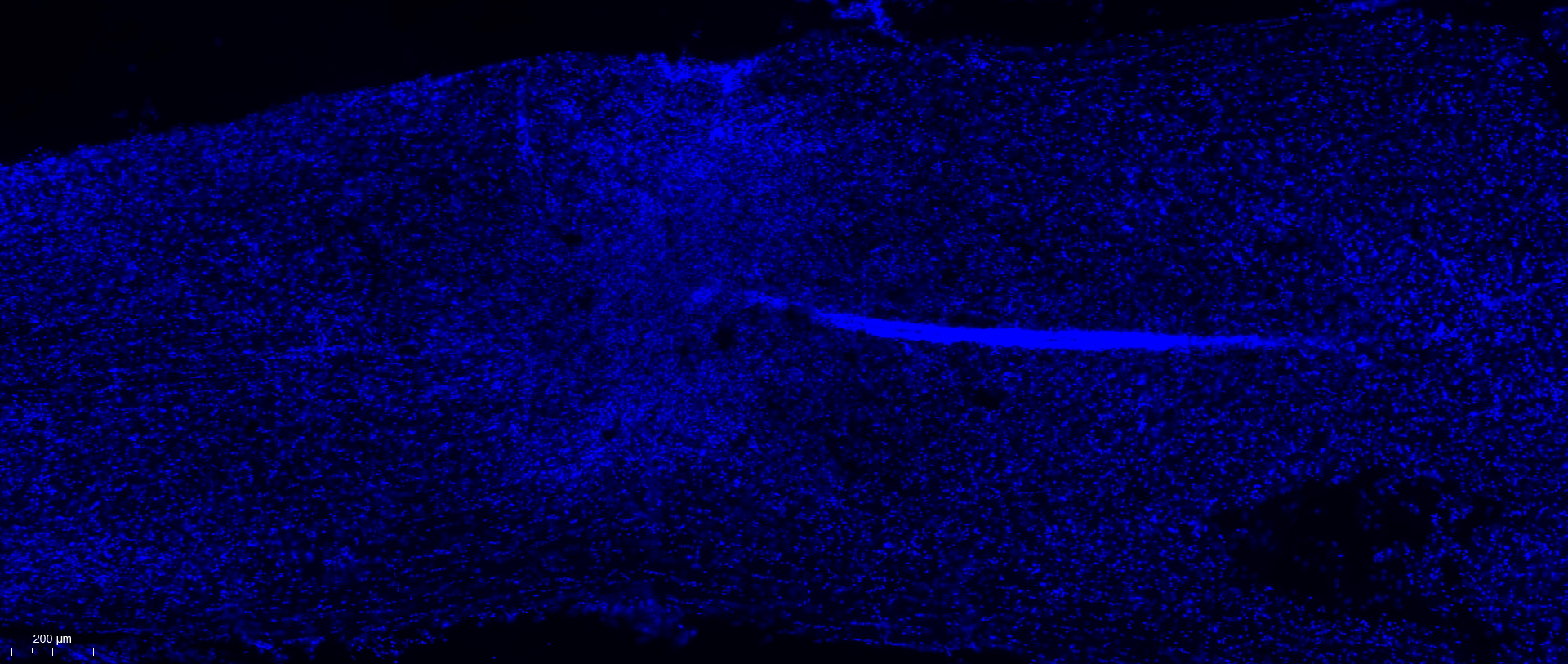

Supplement: Figure 7—source data 1. [file elife-90184-fig7-data1.zip › Figure 7-Source data 1. Raw Images for Figure 7/5-HT/FC-A/1.jpg]

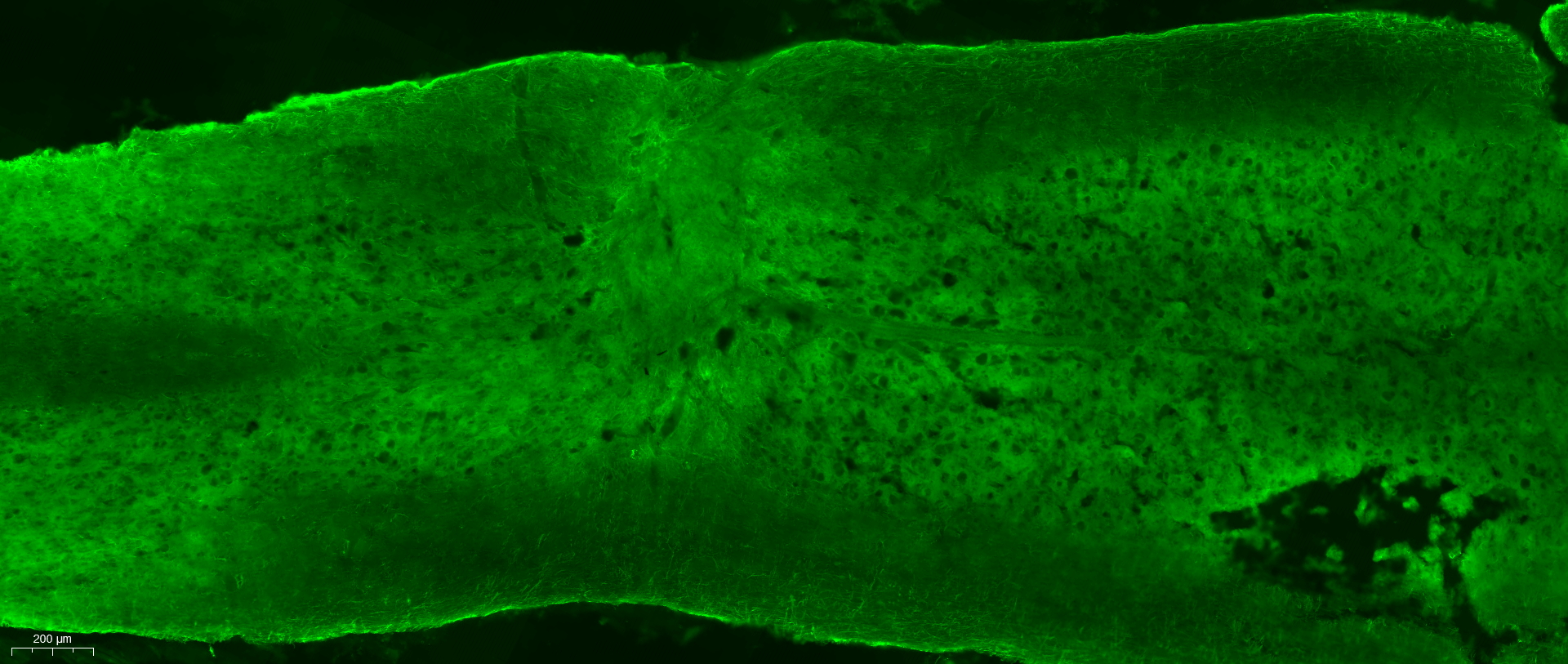

Supplement: Figure 7—source data 1. [file elife-90184-fig7-data1.zip › Figure 7-Source data 1. Raw Images for Figure 7/5-HT/FC-A/2.jpg]

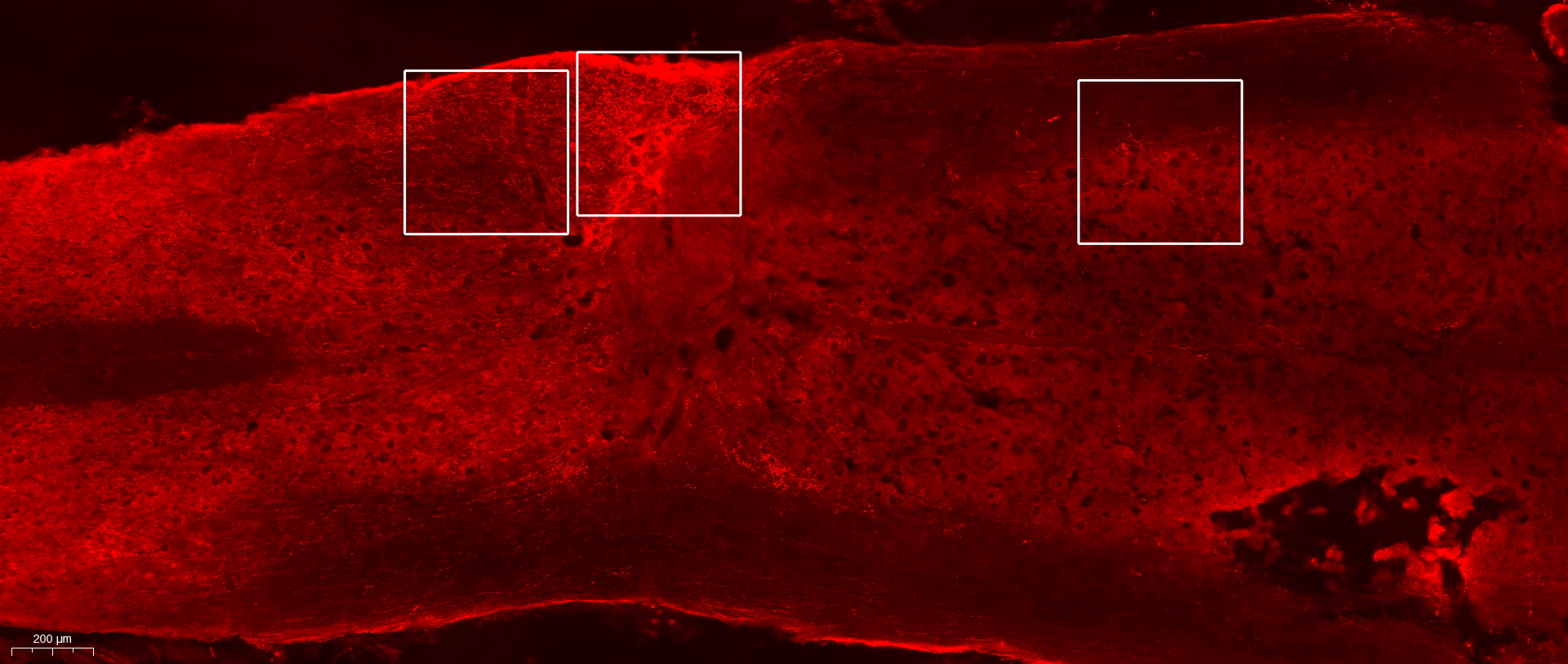

Supplement: Figure 7—source data 1. [file elife-90184-fig7-data1.zip › Figure 7-Source data 1. Raw Images for Figure 7/5-HT/FC-A/3.jpg]

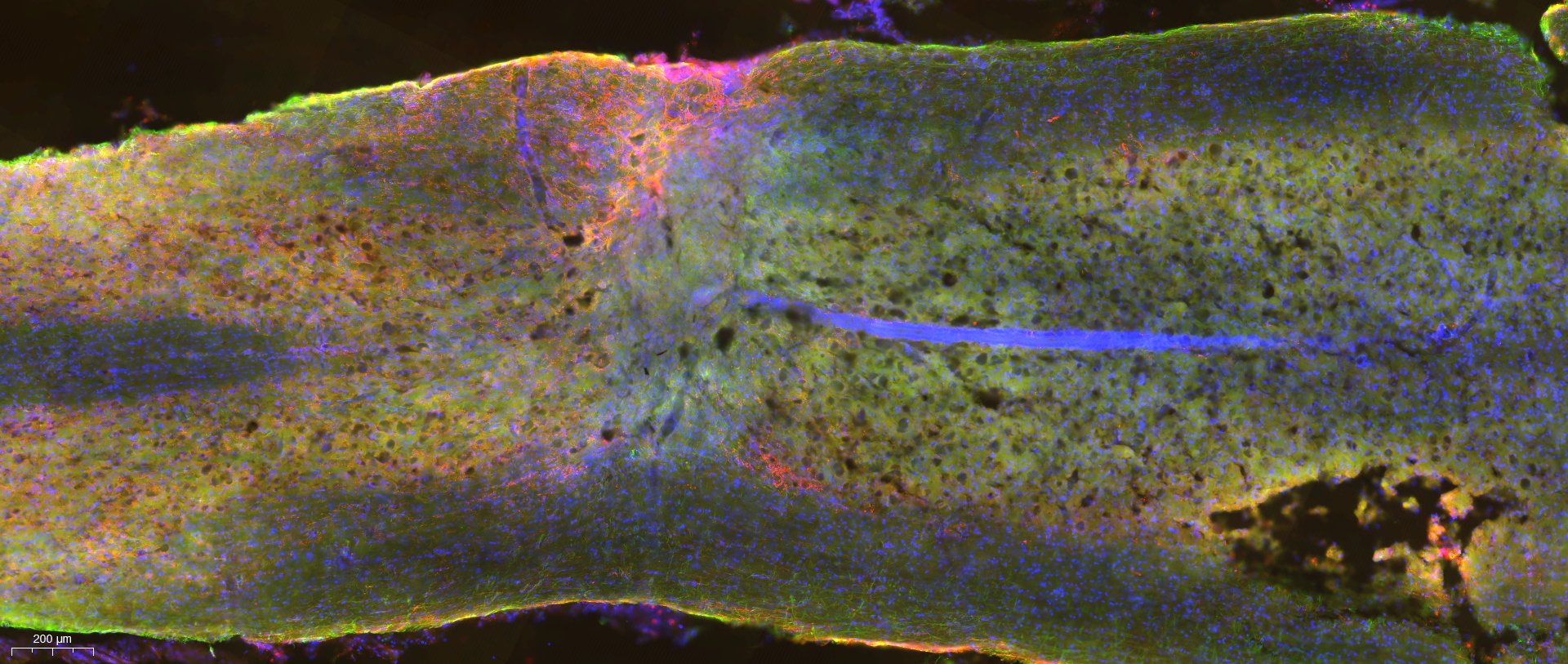

Supplement: Figure 7—source data 1. [file elife-90184-fig7-data1.zip › Figure 7-Source data 1. Raw Images for Figure 7/5-HT/FC-A/4.jpg]

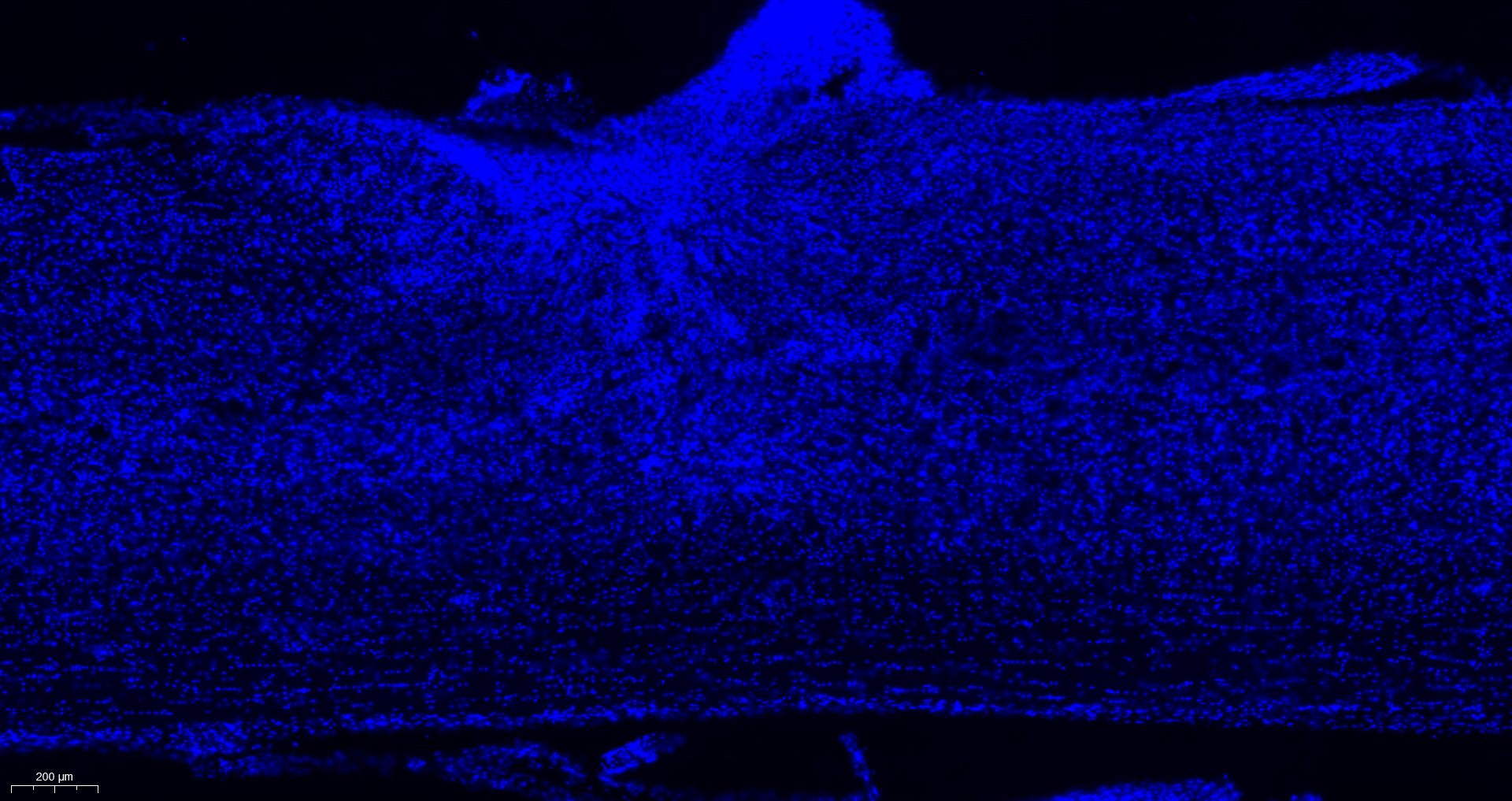

Supplement: Figure 7—source data 1. [file elife-90184-fig7-data1.zip › Figure 7-Source data 1. Raw Images for Figure 7/5-HT/FC-A+Zoline/1.jpg]

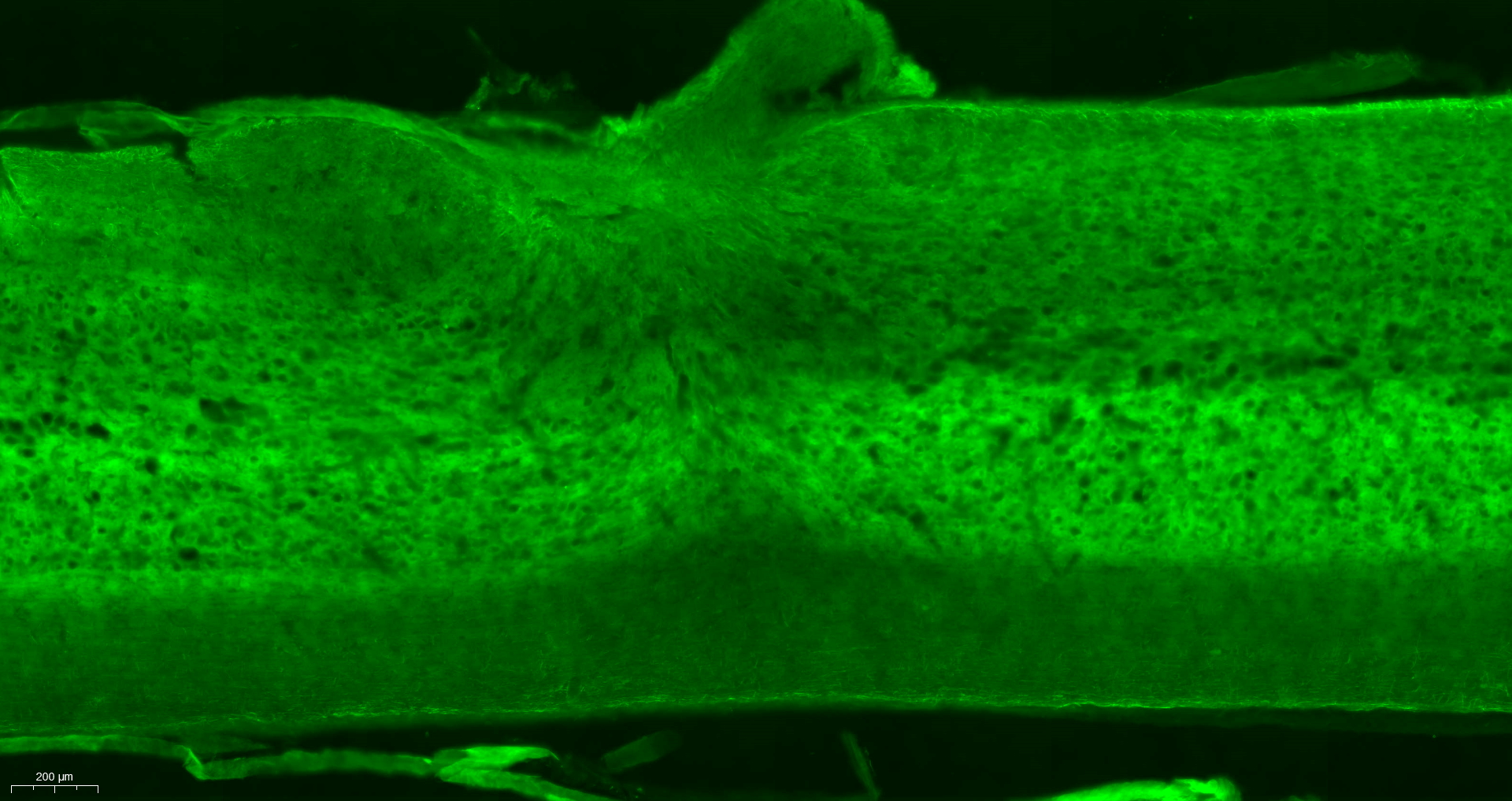

Supplement: Figure 7—source data 1. [file elife-90184-fig7-data1.zip › Figure 7-Source data 1. Raw Images for Figure 7/5-HT/FC-A+Zoline/2.jpg]

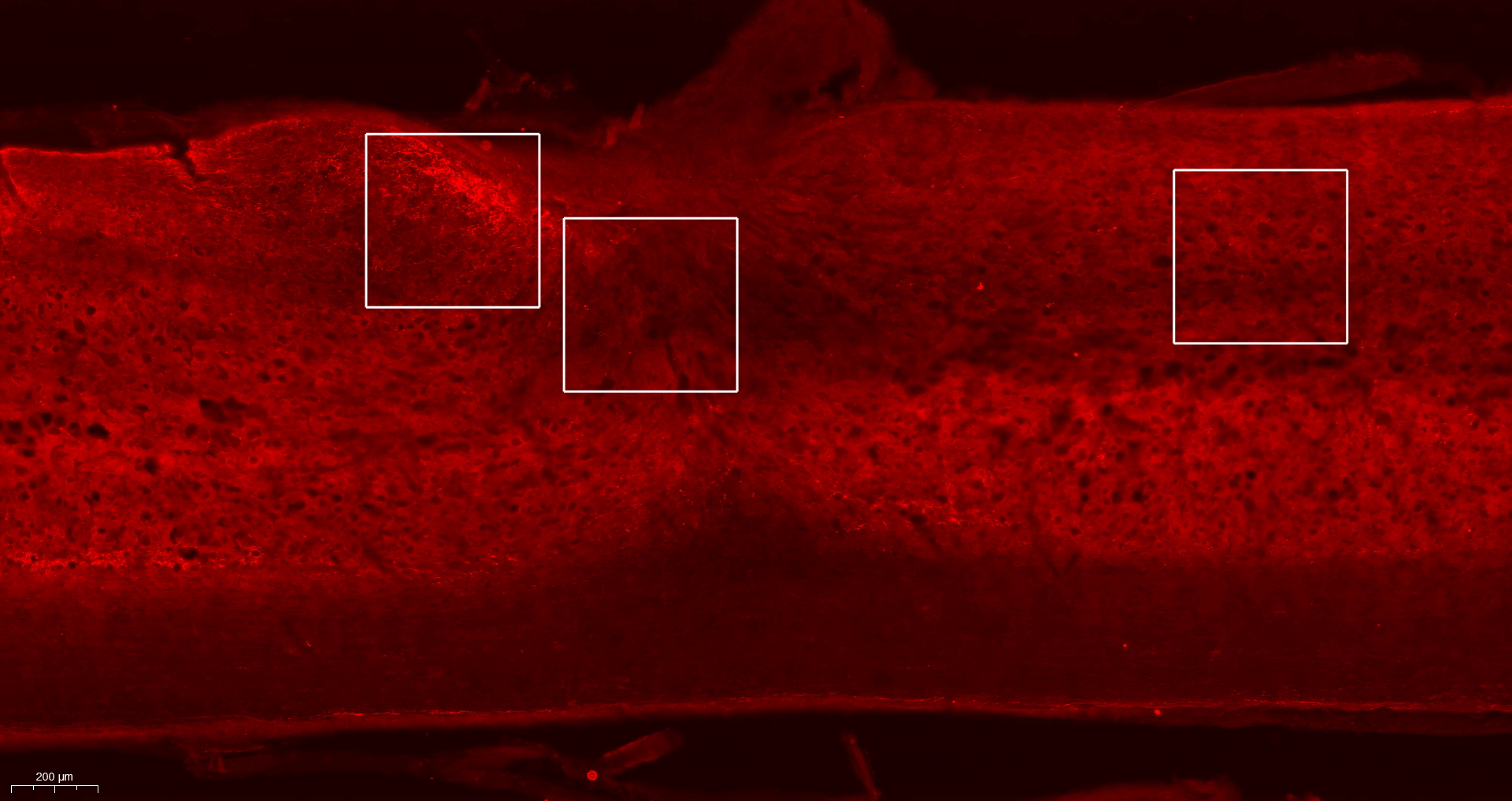

Supplement: Figure 7—source data 1. [file elife-90184-fig7-data1.zip › Figure 7-Source data 1. Raw Images for Figure 7/5-HT/FC-A+Zoline/3.jpg]

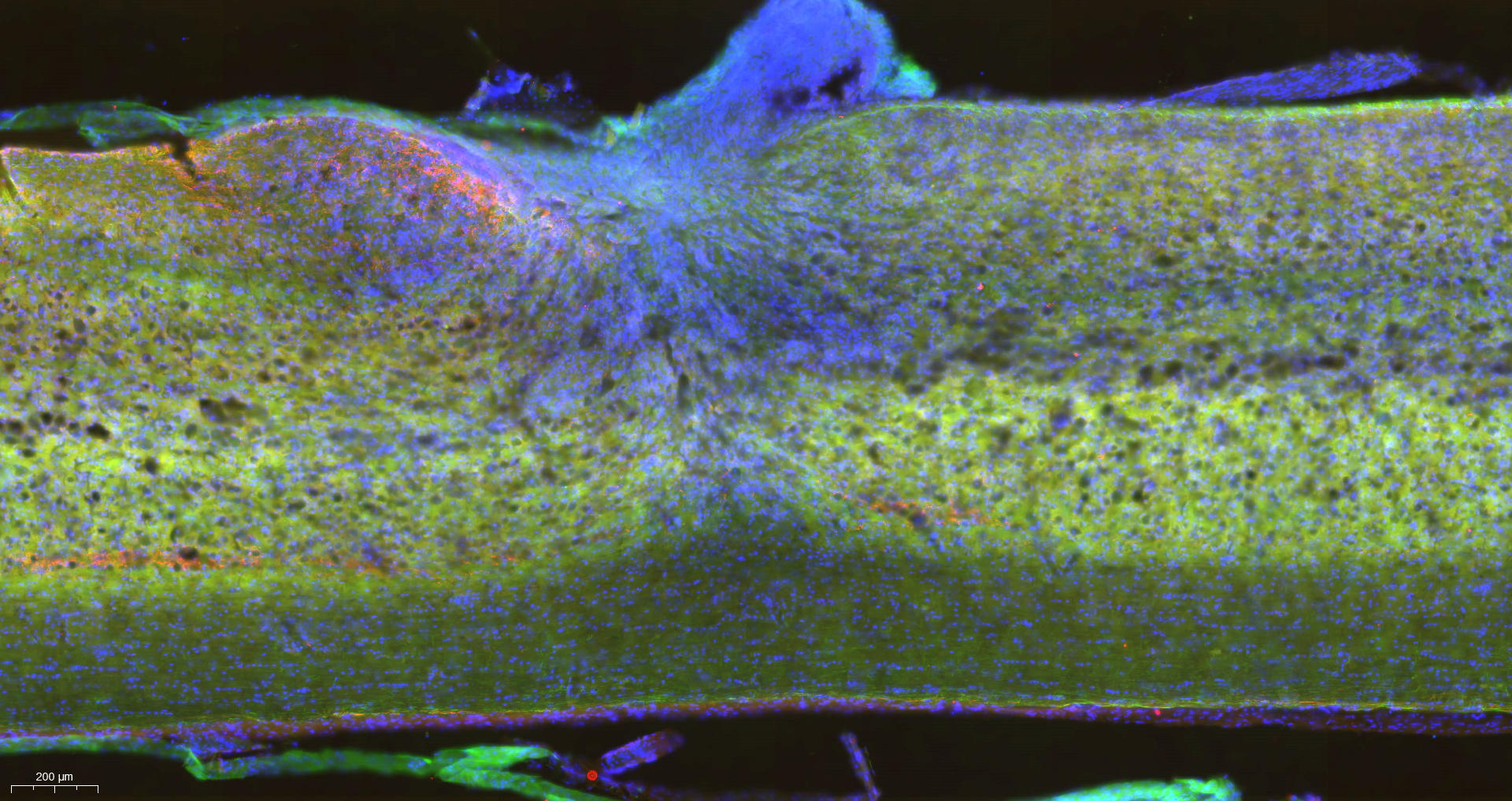

Supplement: Figure 7—source data 1. [file elife-90184-fig7-data1.zip › Figure 7-Source data 1. Raw Images for Figure 7/5-HT/FC-A+Zoline/4.jpg]

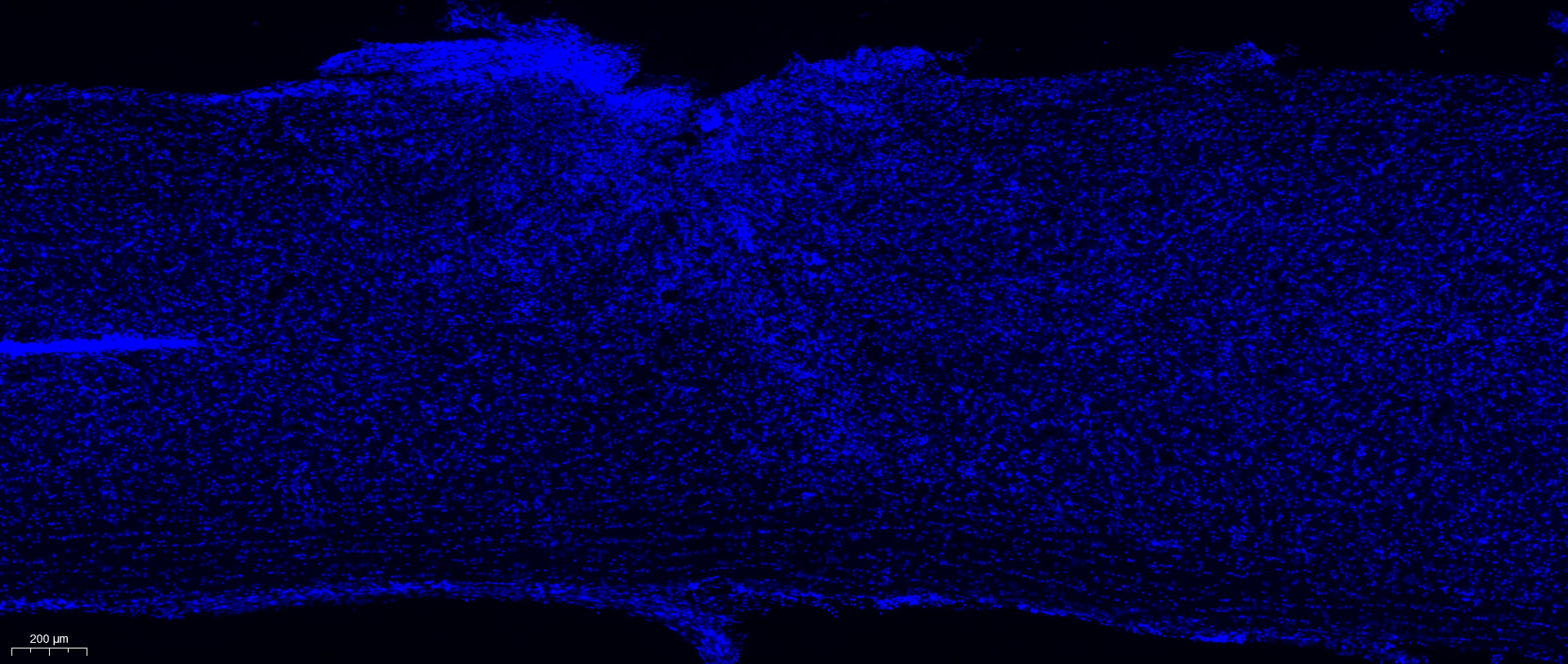

Supplement: Figure 7—source data 1. [file elife-90184-fig7-data1.zip › Figure 7-Source data 1. Raw Images for Figure 7/5-HT/Injury/1.jpg]

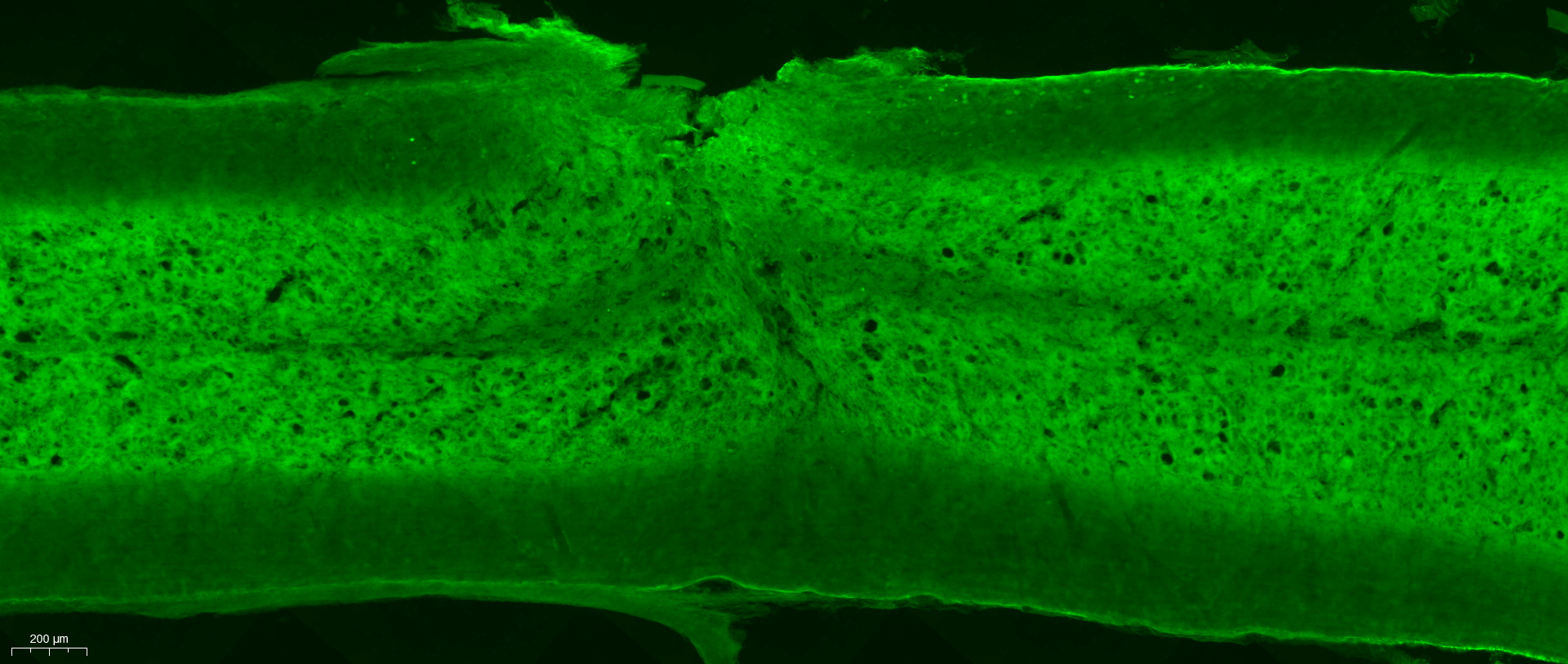

Supplement: Figure 7—source data 1. [file elife-90184-fig7-data1.zip › Figure 7-Source data 1. Raw Images for Figure 7/5-HT/Injury/2.jpg]

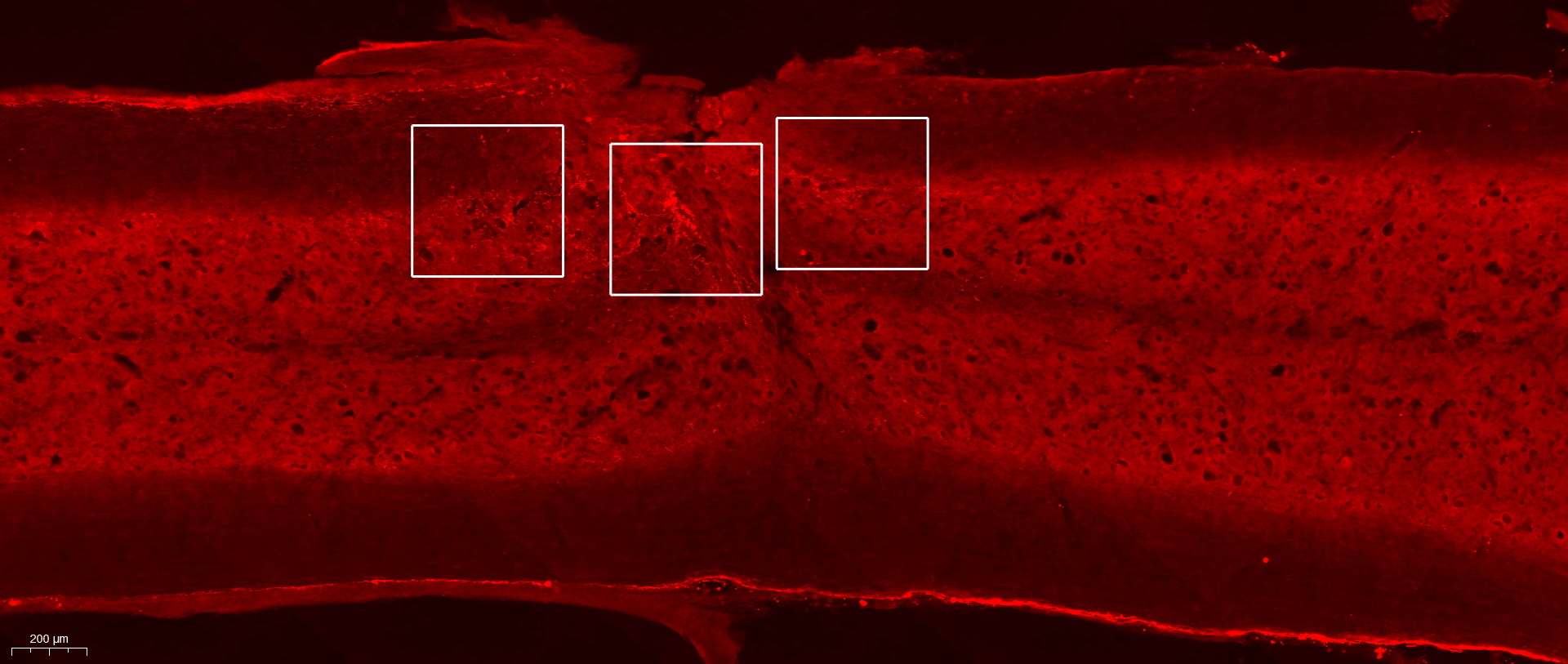

Supplement: Figure 7—source data 1. [file elife-90184-fig7-data1.zip › Figure 7-Source data 1. Raw Images for Figure 7/5-HT/Injury/3.jpg]

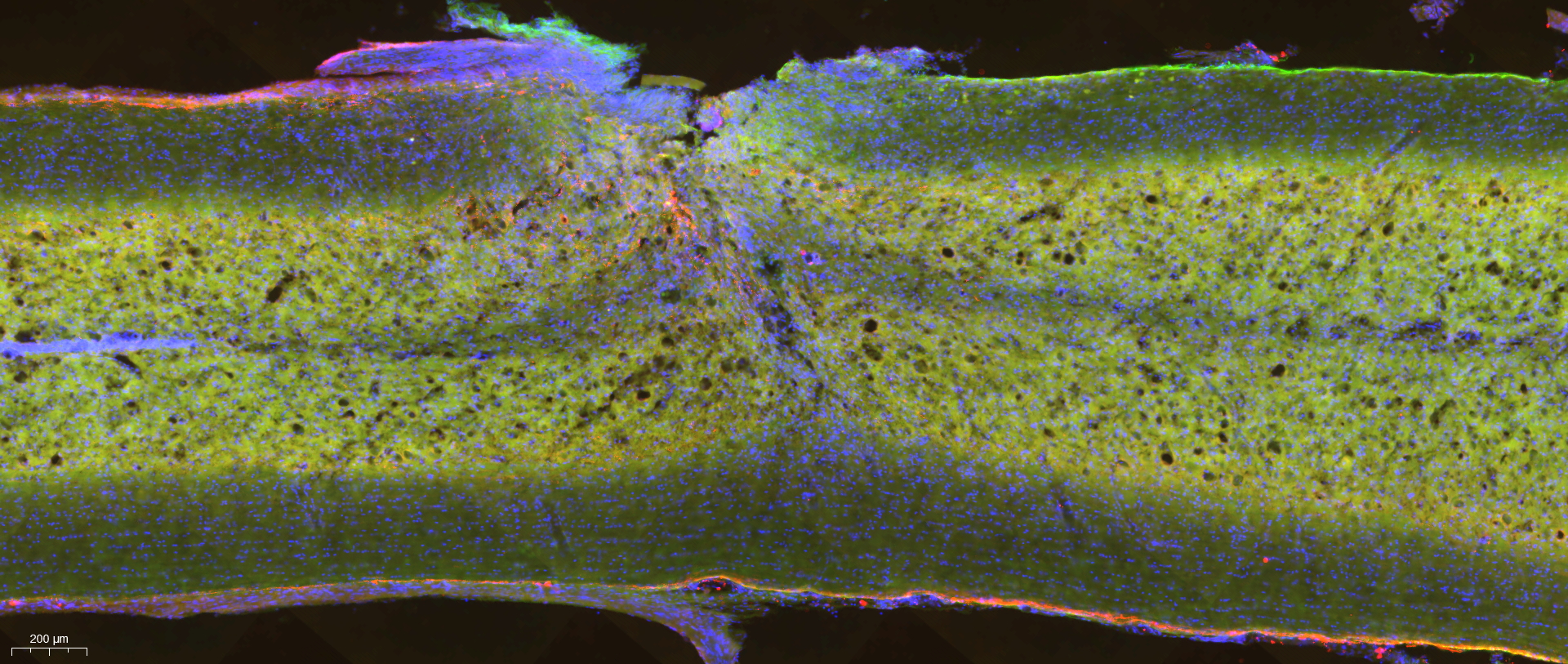

Supplement: Figure 7—source data 1. [file elife-90184-fig7-data1.zip › Figure 7-Source data 1. Raw Images for Figure 7/5-HT/Injury/4.jpg]

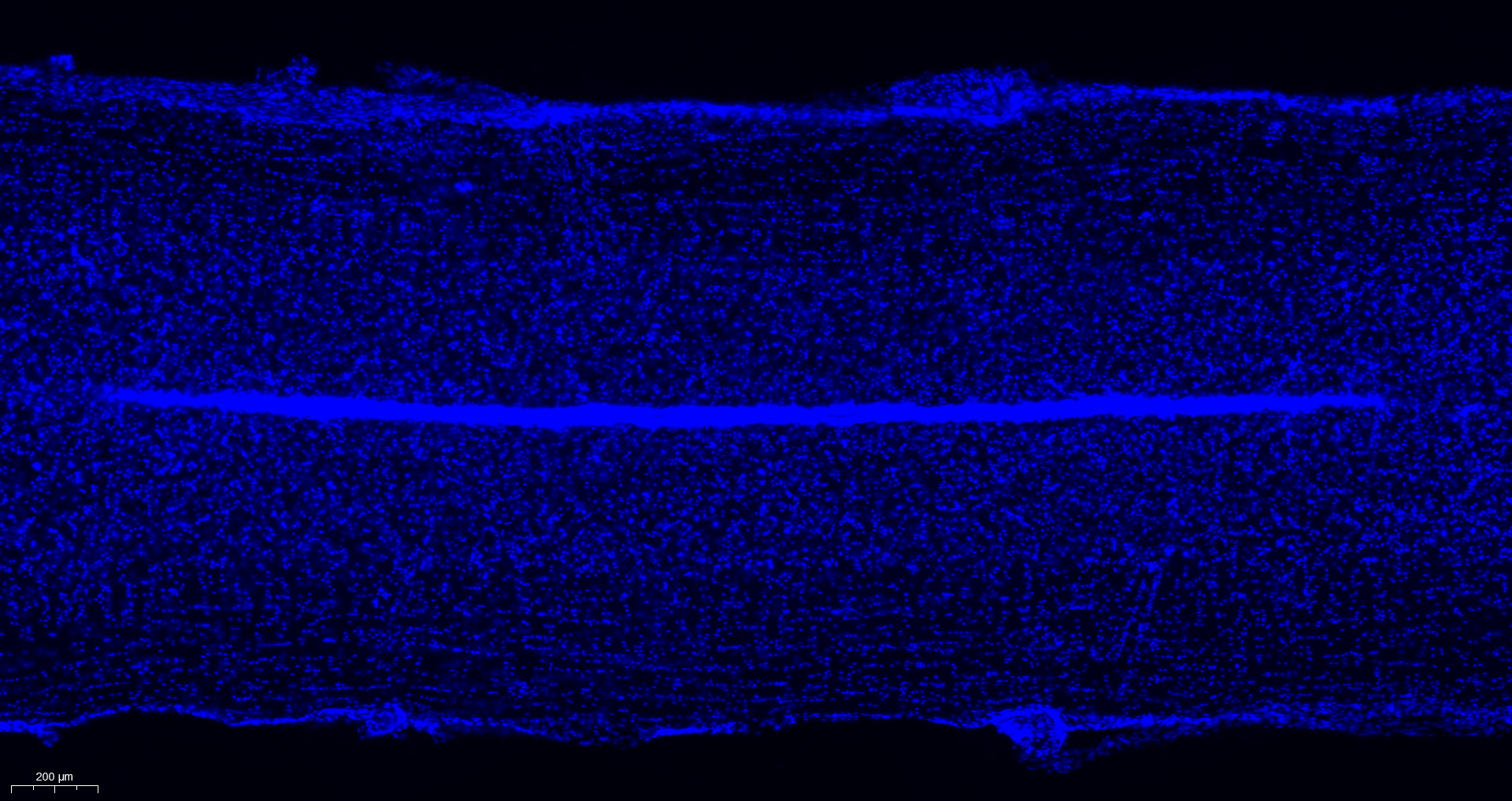

Supplement: Figure 7—source data 1. [file elife-90184-fig7-data1.zip › Figure 7-Source data 1. Raw Images for Figure 7/5-HT/Sham/1.jpg]

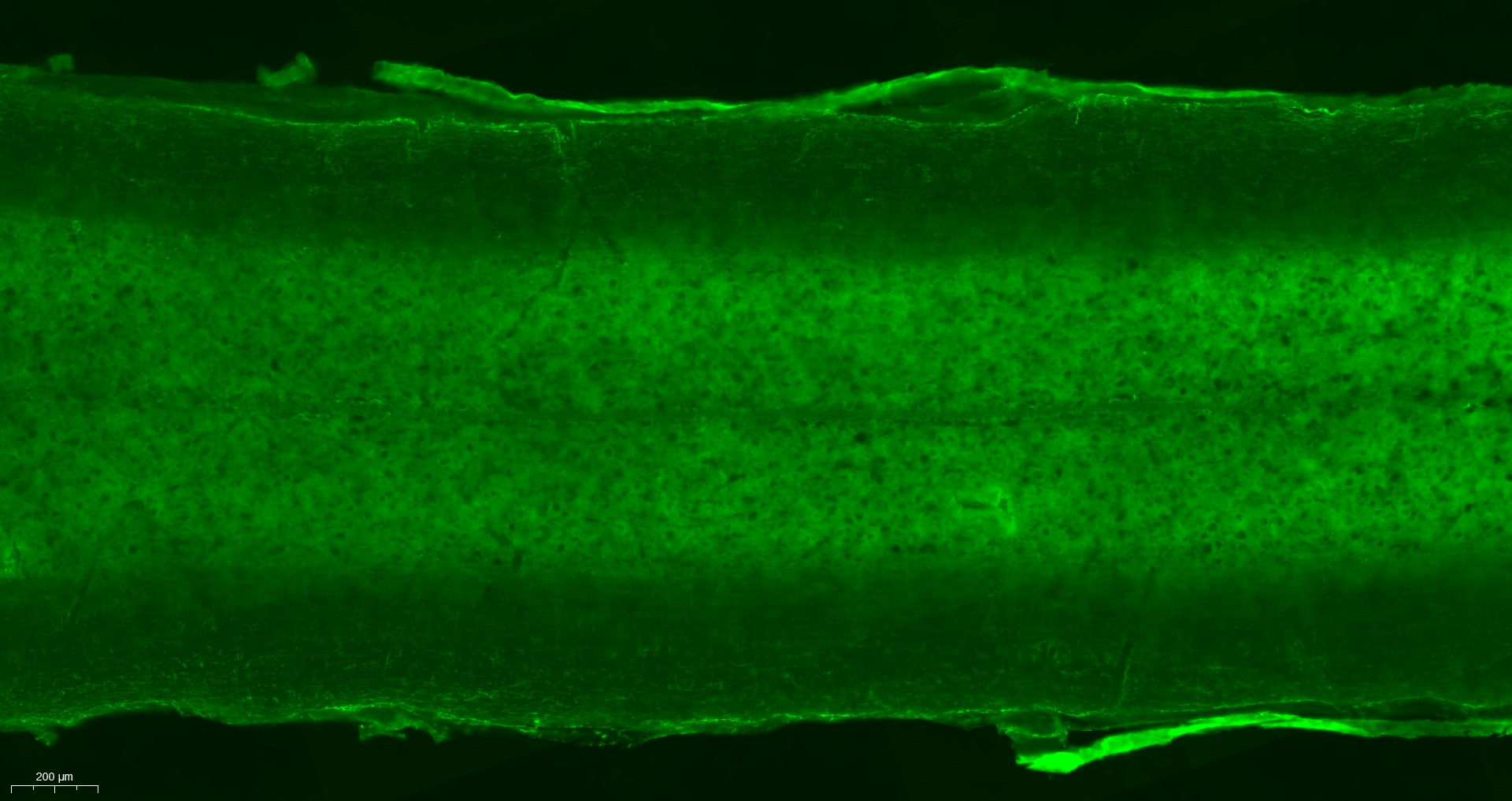

Supplement: Figure 7—source data 1. [file elife-90184-fig7-data1.zip › Figure 7-Source data 1. Raw Images for Figure 7/5-HT/Sham/2.jpg]

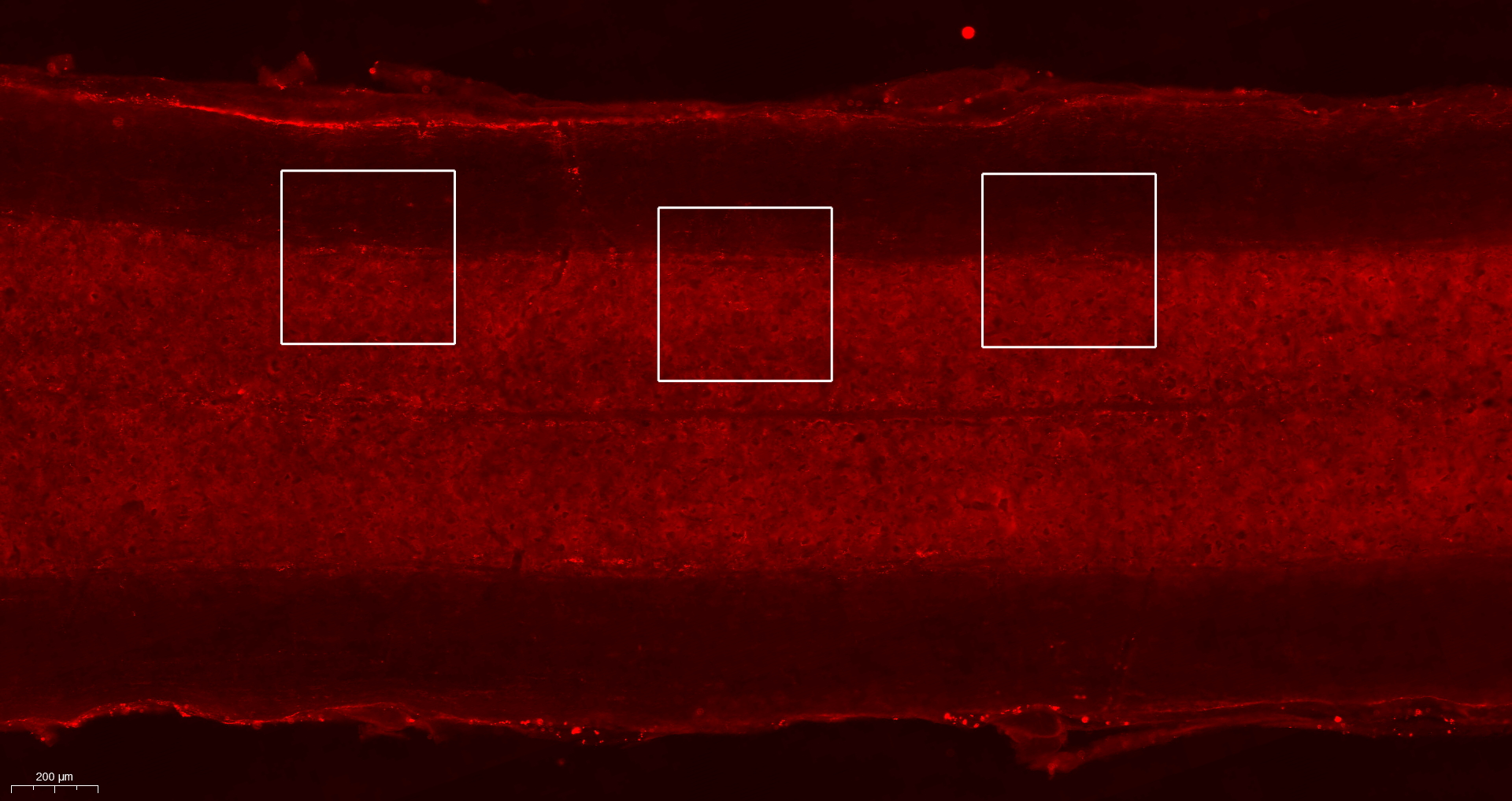

Supplement: Figure 7—source data 1. [file elife-90184-fig7-data1.zip › Figure 7-Source data 1. Raw Images for Figure 7/5-HT/Sham/3.jpg]

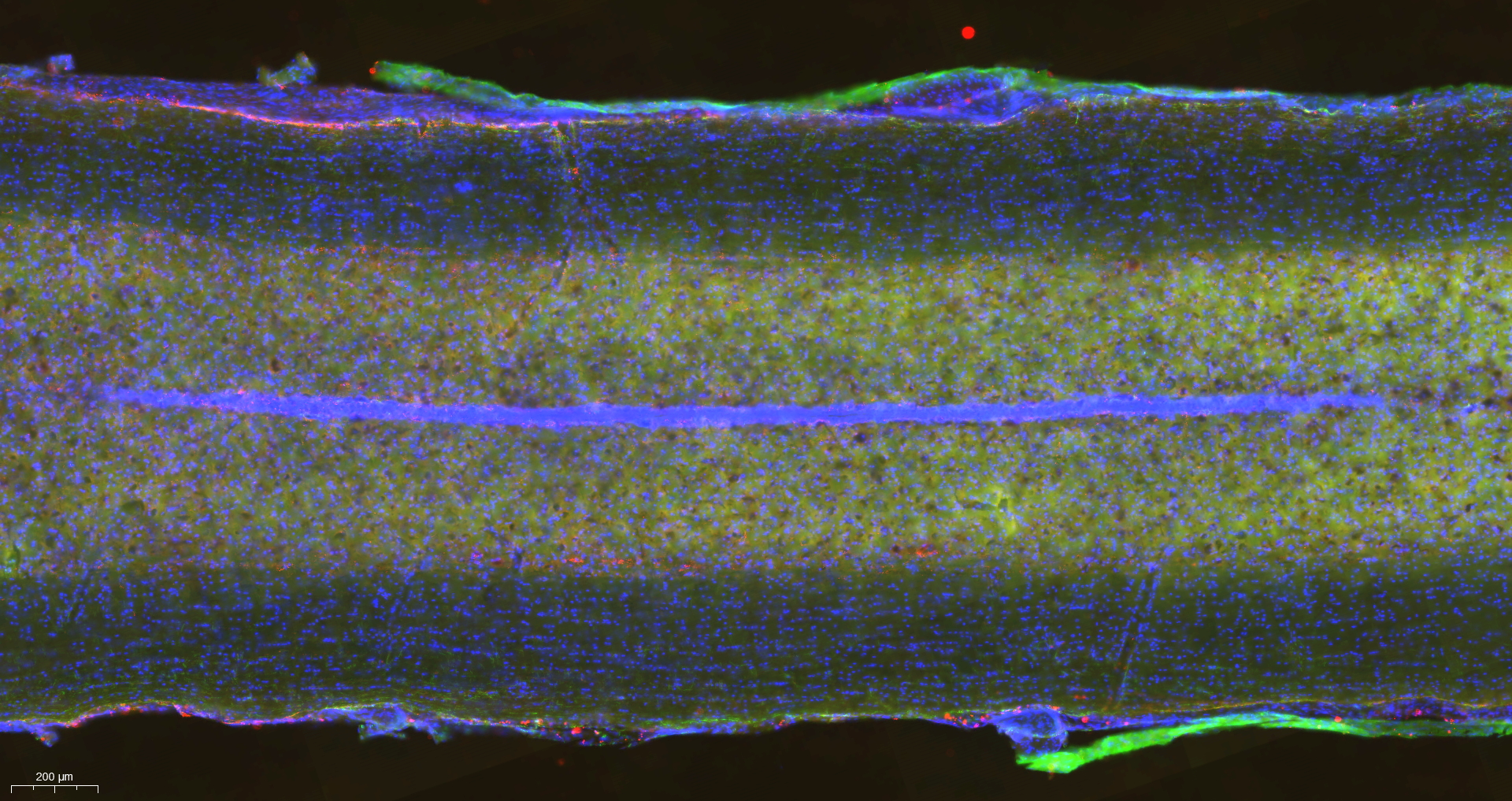

Supplement: Figure 7—source data 1. [file elife-90184-fig7-data1.zip › Figure 7-Source data 1. Raw Images for Figure 7/5-HT/Sham/4.jpg]

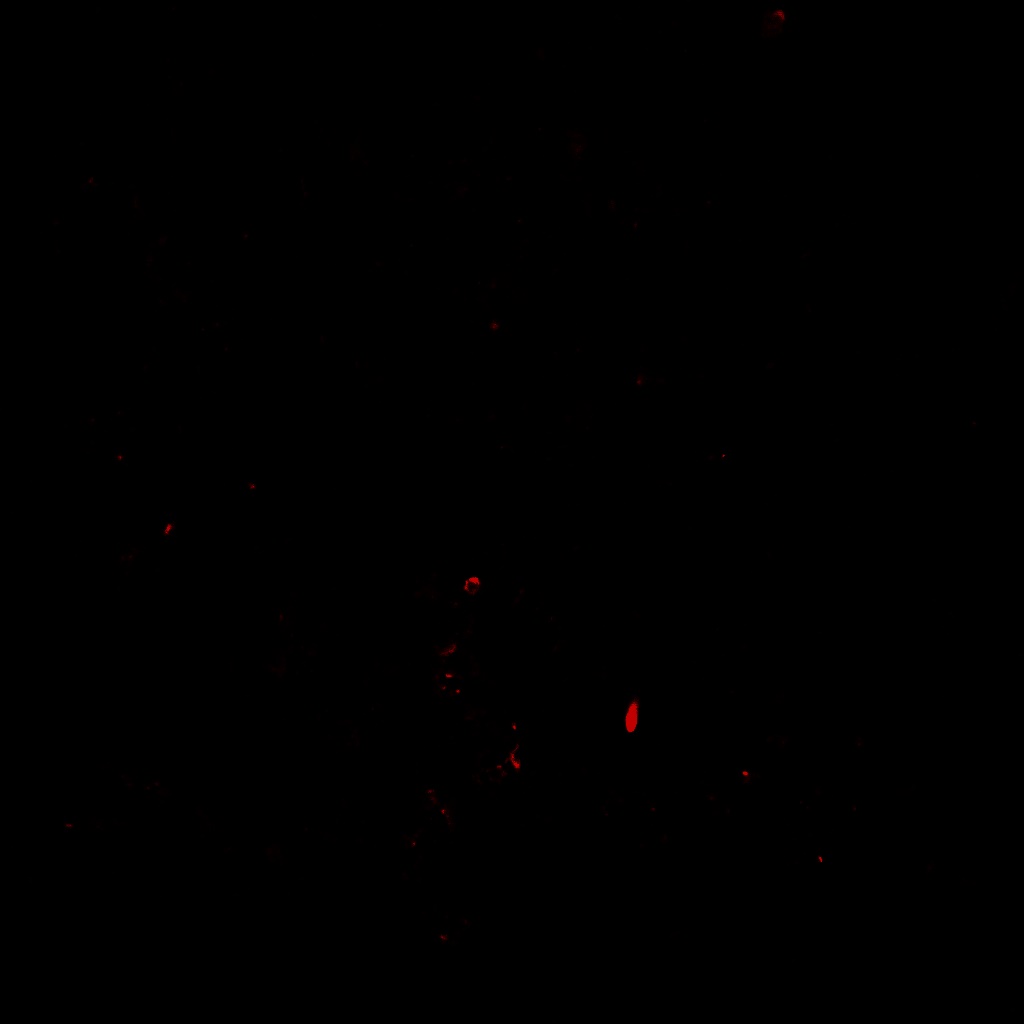

Supplement: Figure 7—source data 1. [file elife-90184-fig7-data1.zip › Figure 7-Source data 1. Raw Images for Figure 7/NFH and MBP images/FC+Zoline/Image 8-╡Ñ╕÷╬─╝■╡╝│÷-05_c1.jpg]

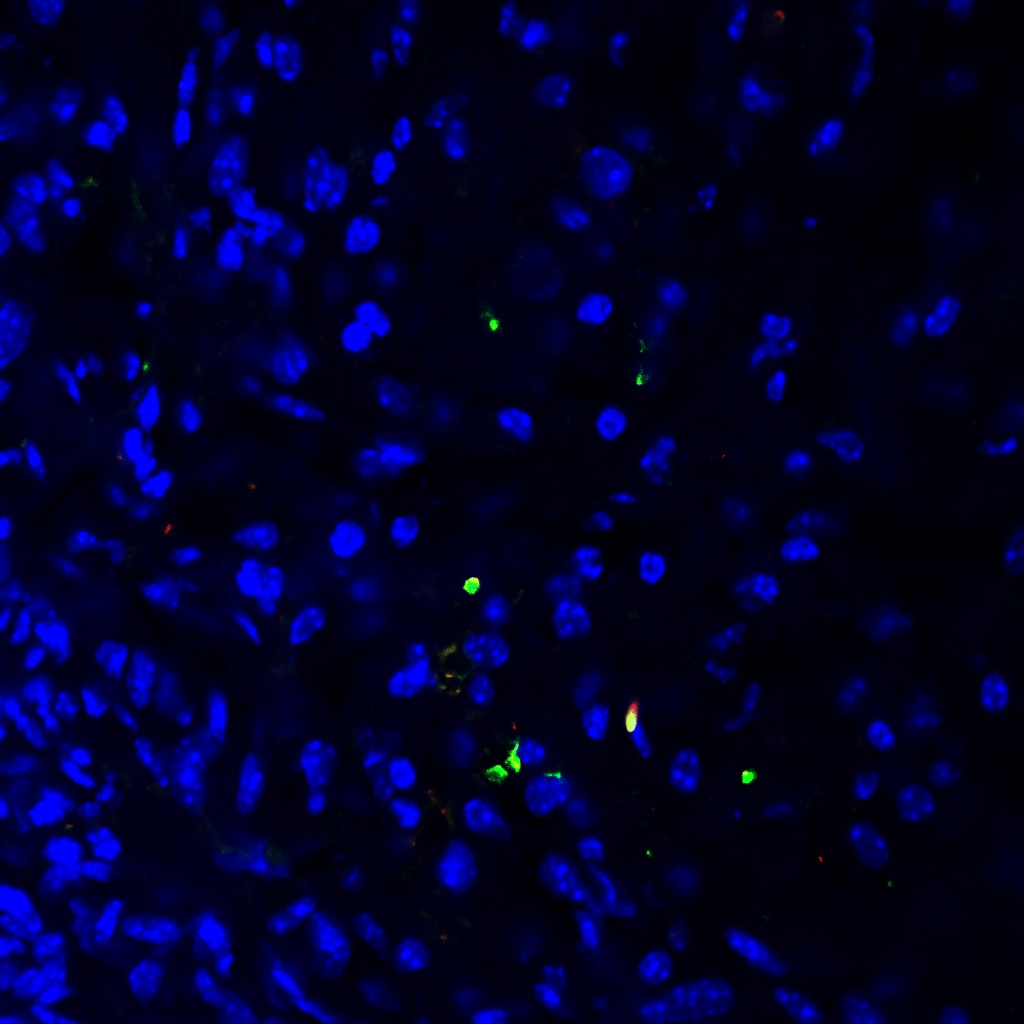

Supplement: Figure 7—source data 1. [file elife-90184-fig7-data1.zip › Figure 7-Source data 1. Raw Images for Figure 7/NFH and MBP images/FC+Zoline/Image 8-╡Ñ╕÷╬─╝■╡╝│÷-05_c1+2+3.jpg]

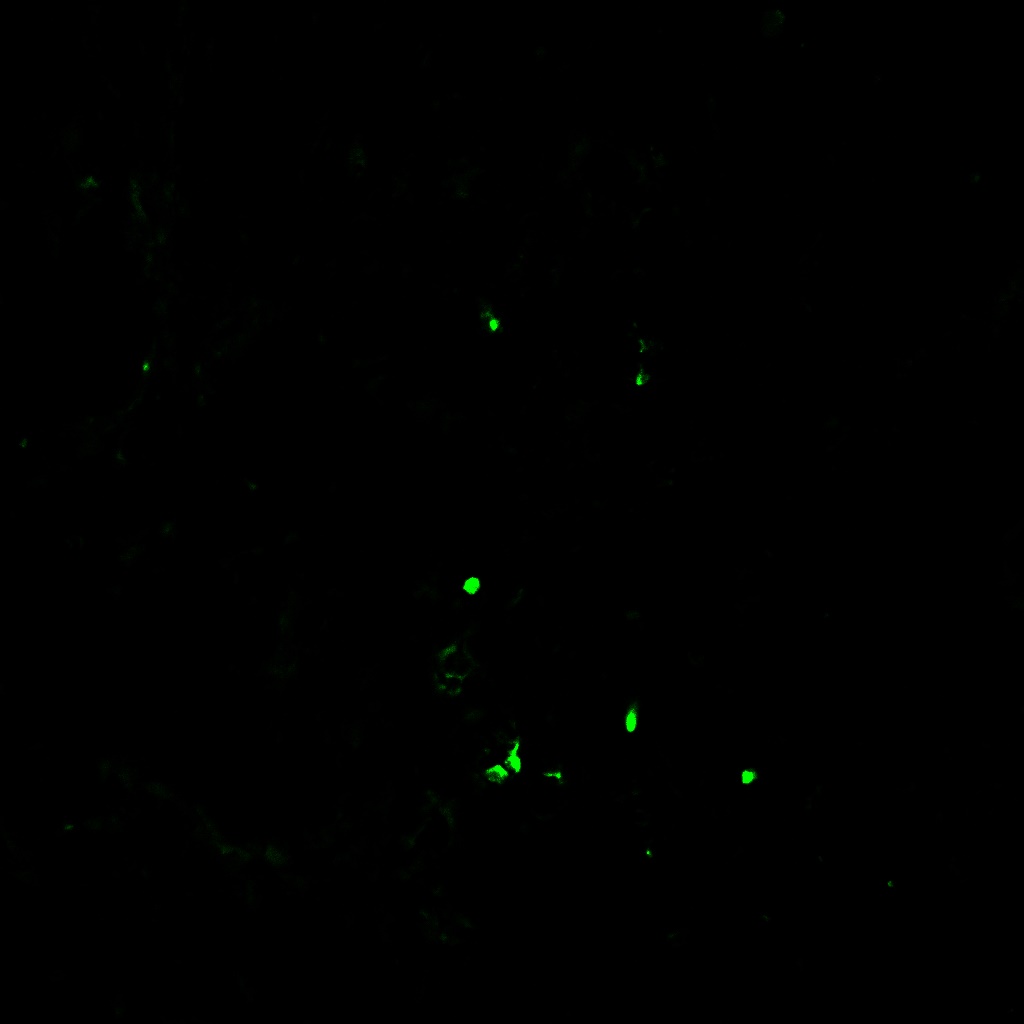

Supplement: Figure 7—source data 1. [file elife-90184-fig7-data1.zip › Figure 7-Source data 1. Raw Images for Figure 7/NFH and MBP images/FC+Zoline/Image 8-╡Ñ╕÷╬─╝■╡╝│÷-05_c2.jpg]

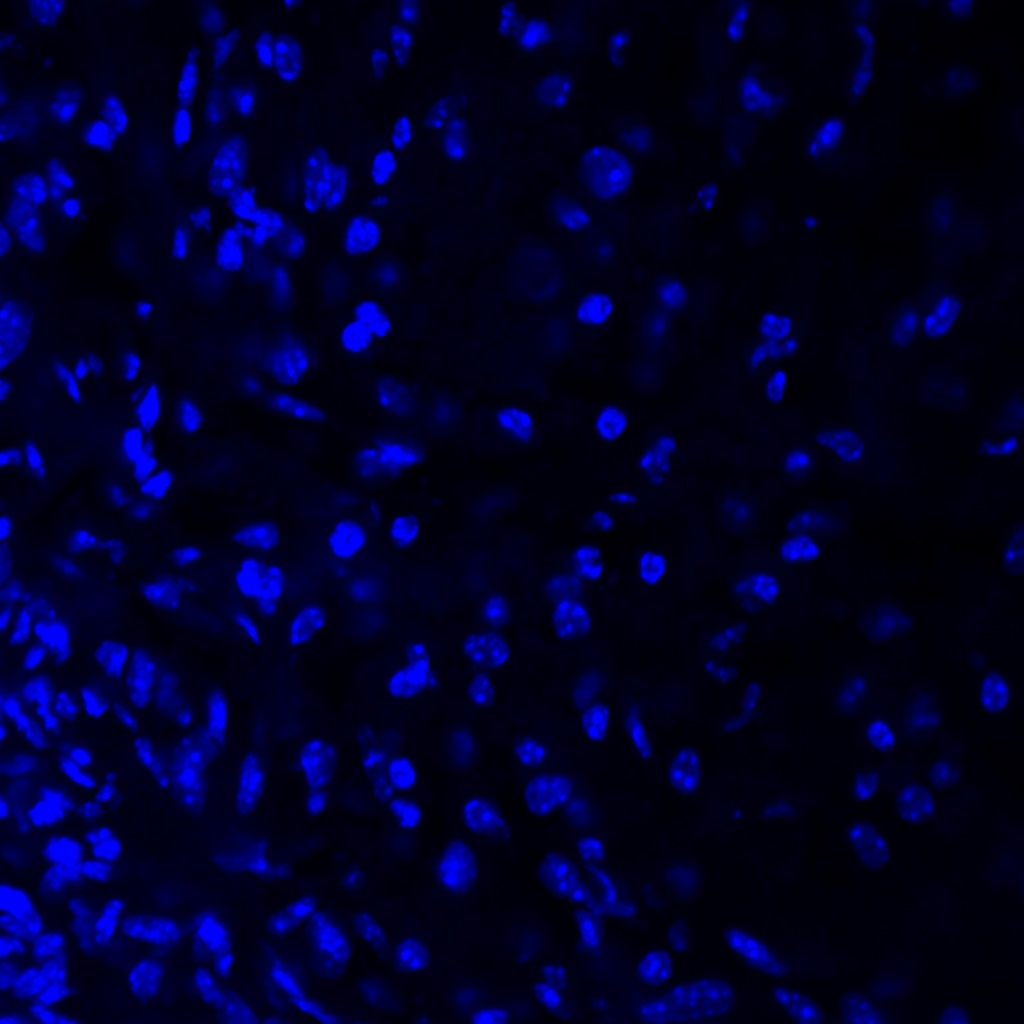

Supplement: Figure 7—source data 1. [file elife-90184-fig7-data1.zip › Figure 7-Source data 1. Raw Images for Figure 7/NFH and MBP images/FC+Zoline/Image 8-╡Ñ╕÷╬─╝■╡╝│÷-05_c3.jpg]

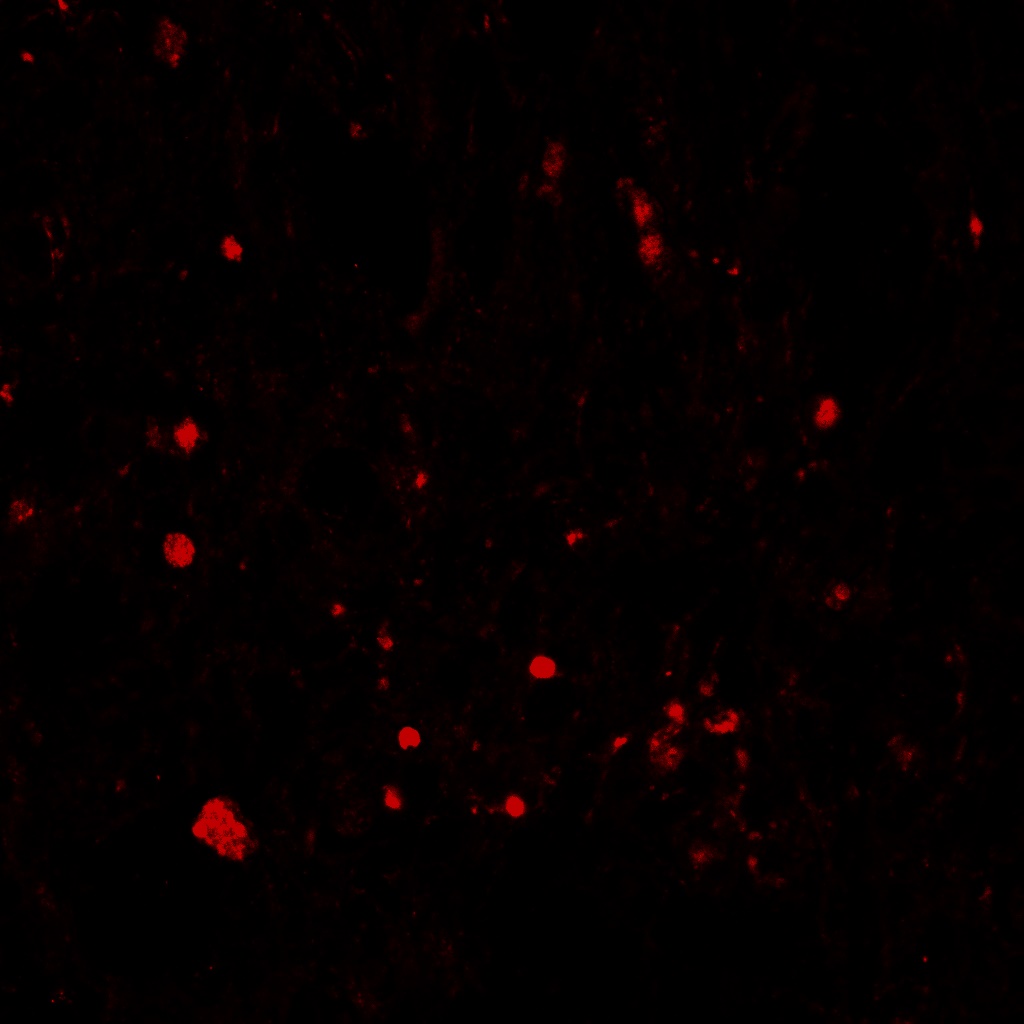

Supplement: Figure 7—source data 1. [file elife-90184-fig7-data1.zip › Figure 7-Source data 1. Raw Images for Figure 7/NFH and MBP images/FC-A/Image 7-╡Ñ╕÷╬─╝■╡╝│÷-04_c1.jpg]

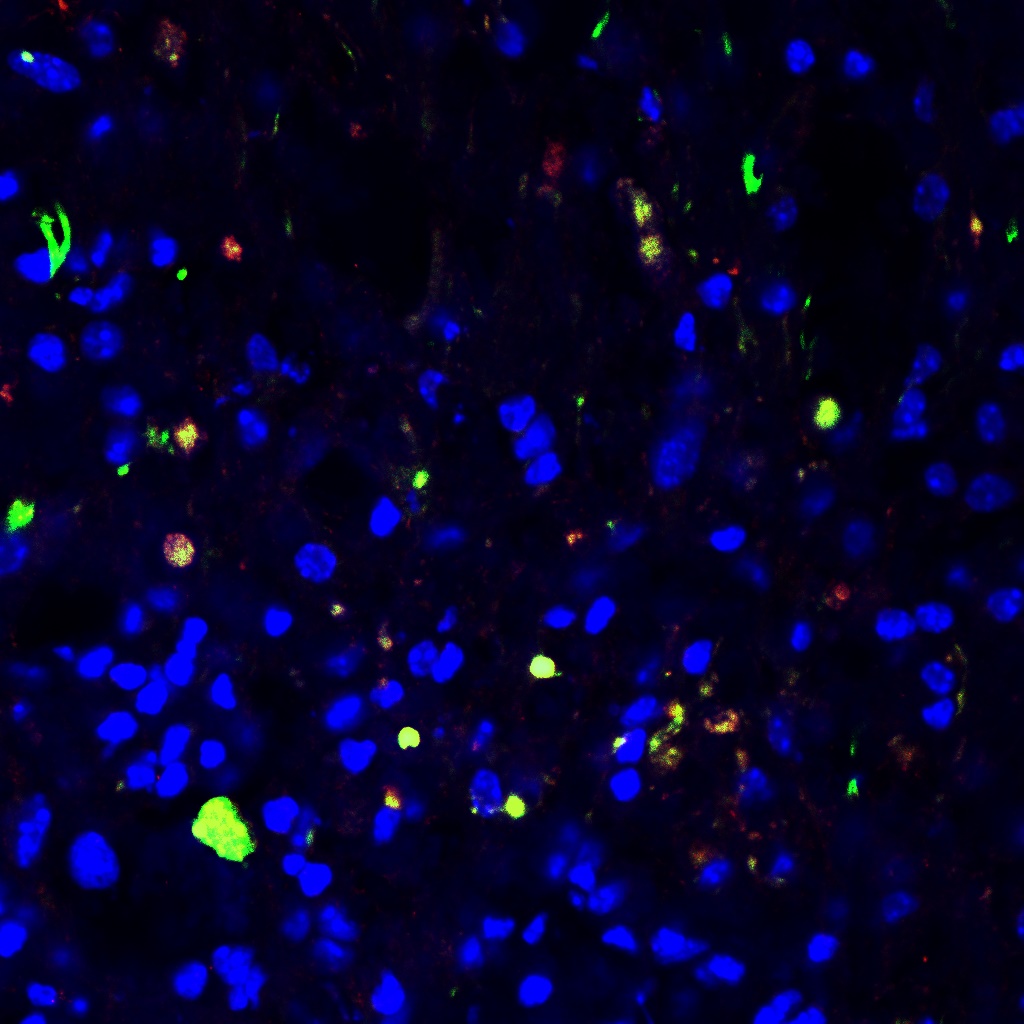

Supplement: Figure 7—source data 1. [file elife-90184-fig7-data1.zip › Figure 7-Source data 1. Raw Images for Figure 7/NFH and MBP images/FC-A/Image 7-╡Ñ╕÷╬─╝■╡╝│÷-04_c1+2+3.jpg]

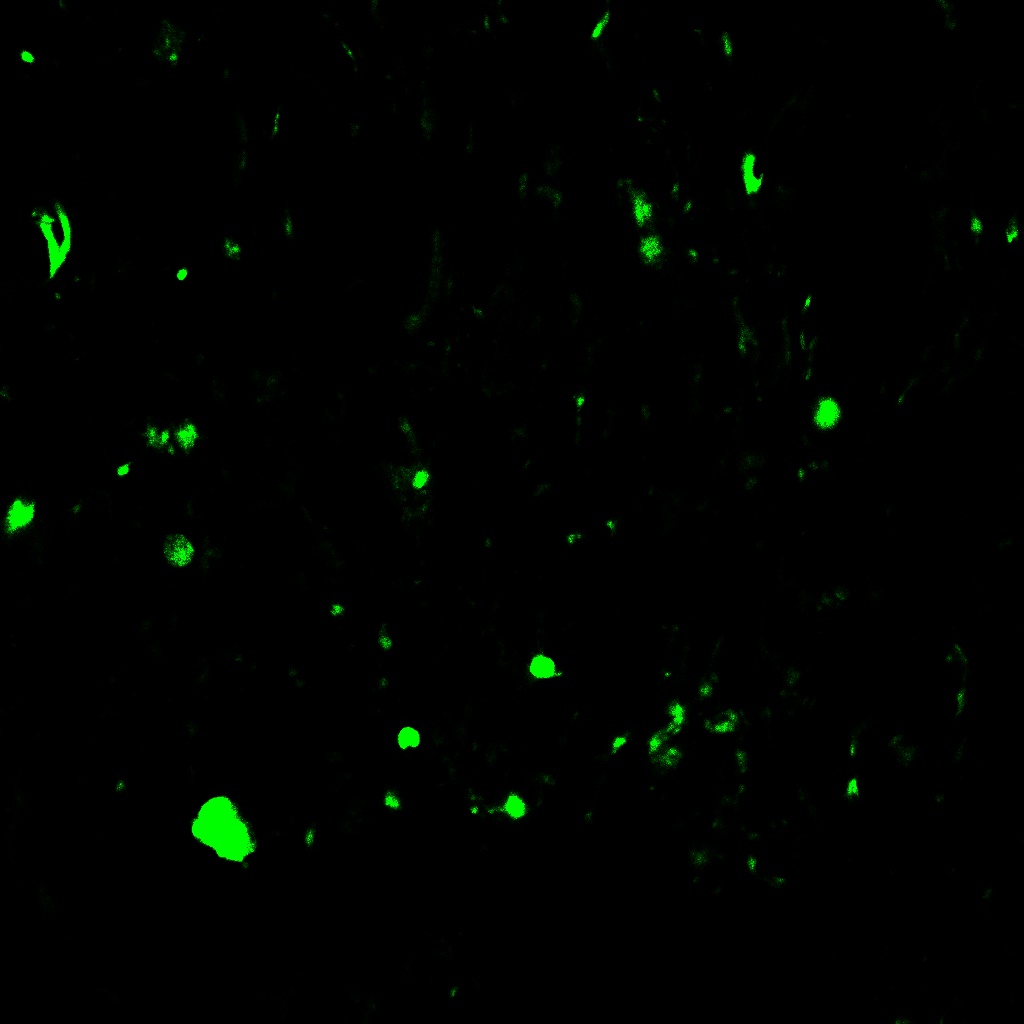

Supplement: Figure 7—source data 1. [file elife-90184-fig7-data1.zip › Figure 7-Source data 1. Raw Images for Figure 7/NFH and MBP images/FC-A/Image 7-╡Ñ╕÷╬─╝■╡╝│÷-04_c2.jpg]

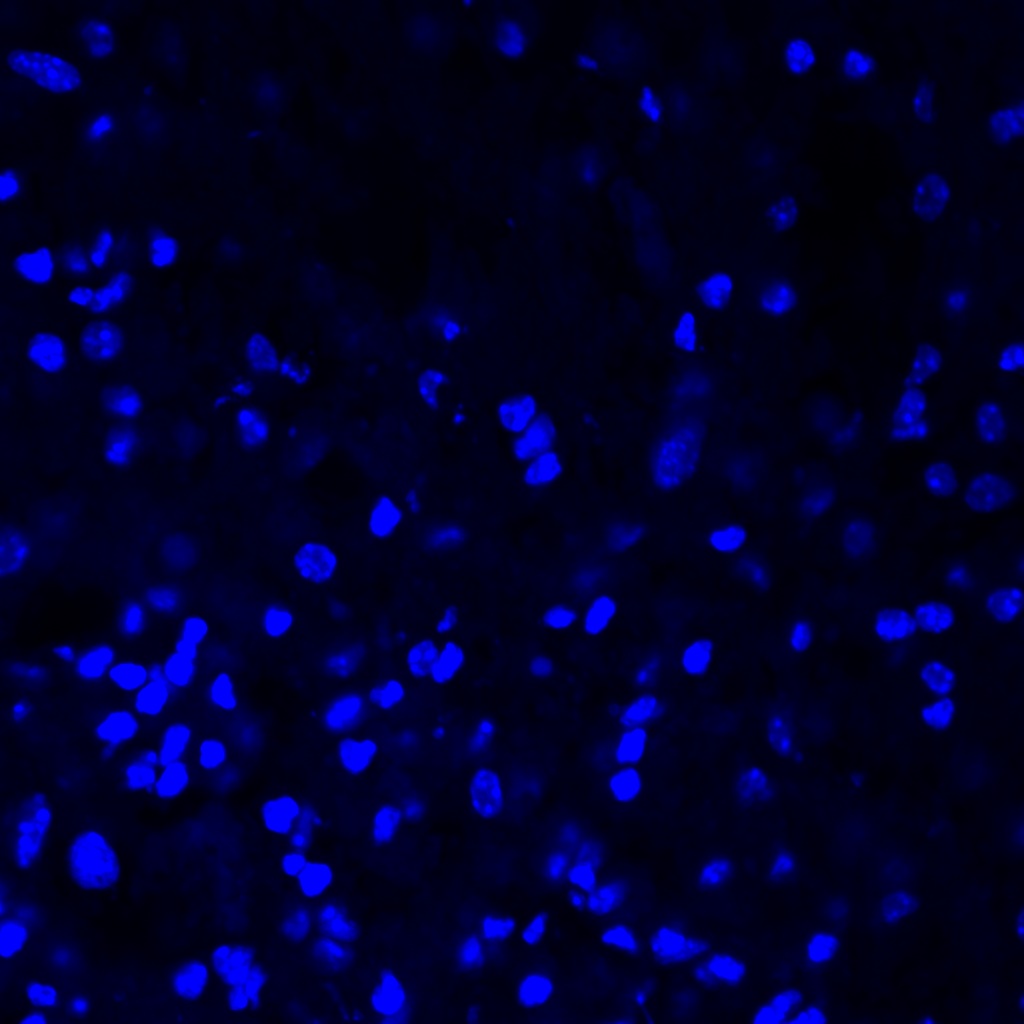

Supplement: Figure 7—source data 1. [file elife-90184-fig7-data1.zip › Figure 7-Source data 1. Raw Images for Figure 7/NFH and MBP images/FC-A/Image 7-╡Ñ╕÷╬─╝■╡╝│÷-04_c3.jpg]

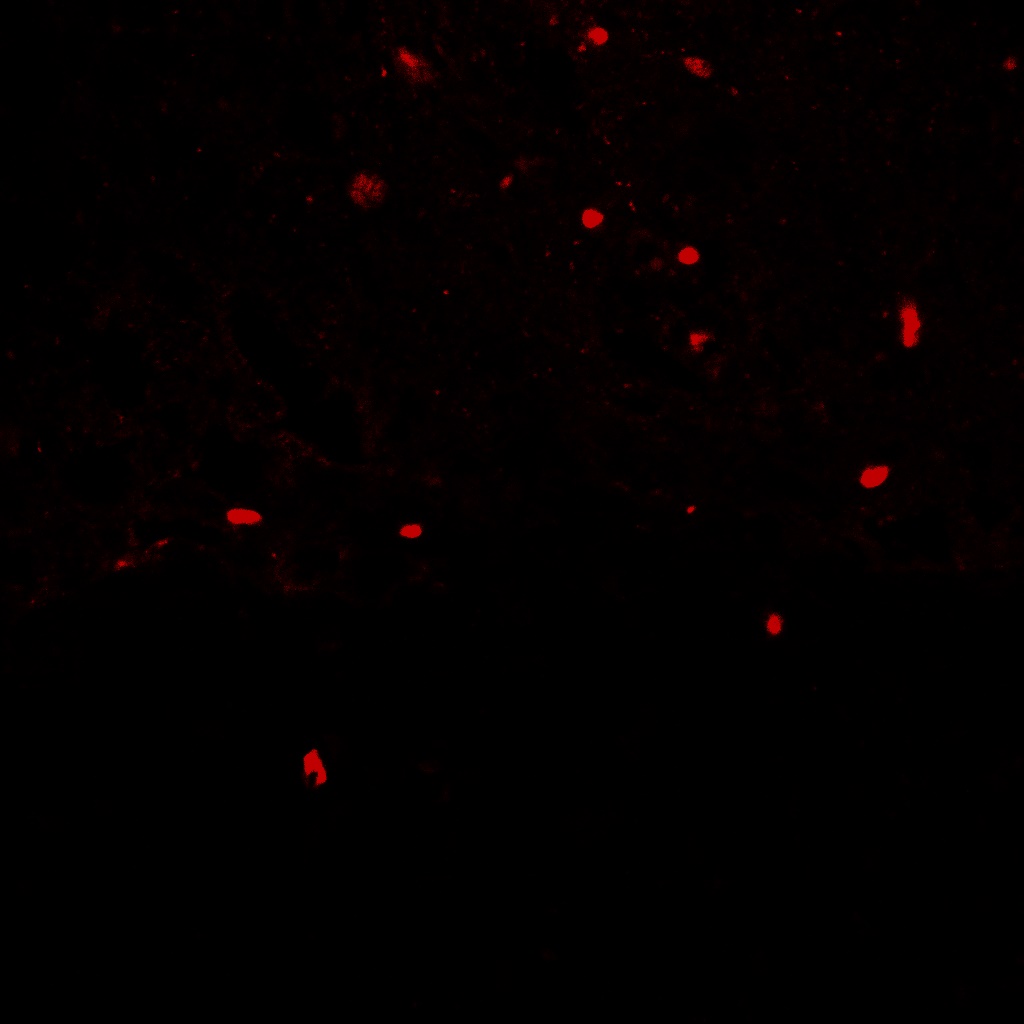

Supplement: Figure 7—source data 1. [file elife-90184-fig7-data1.zip › Figure 7-Source data 1. Raw Images for Figure 7/NFH and MBP images/Injury/Image 9-╡Ñ╕÷╬─╝■╡╝│÷-06_c1.jpg]

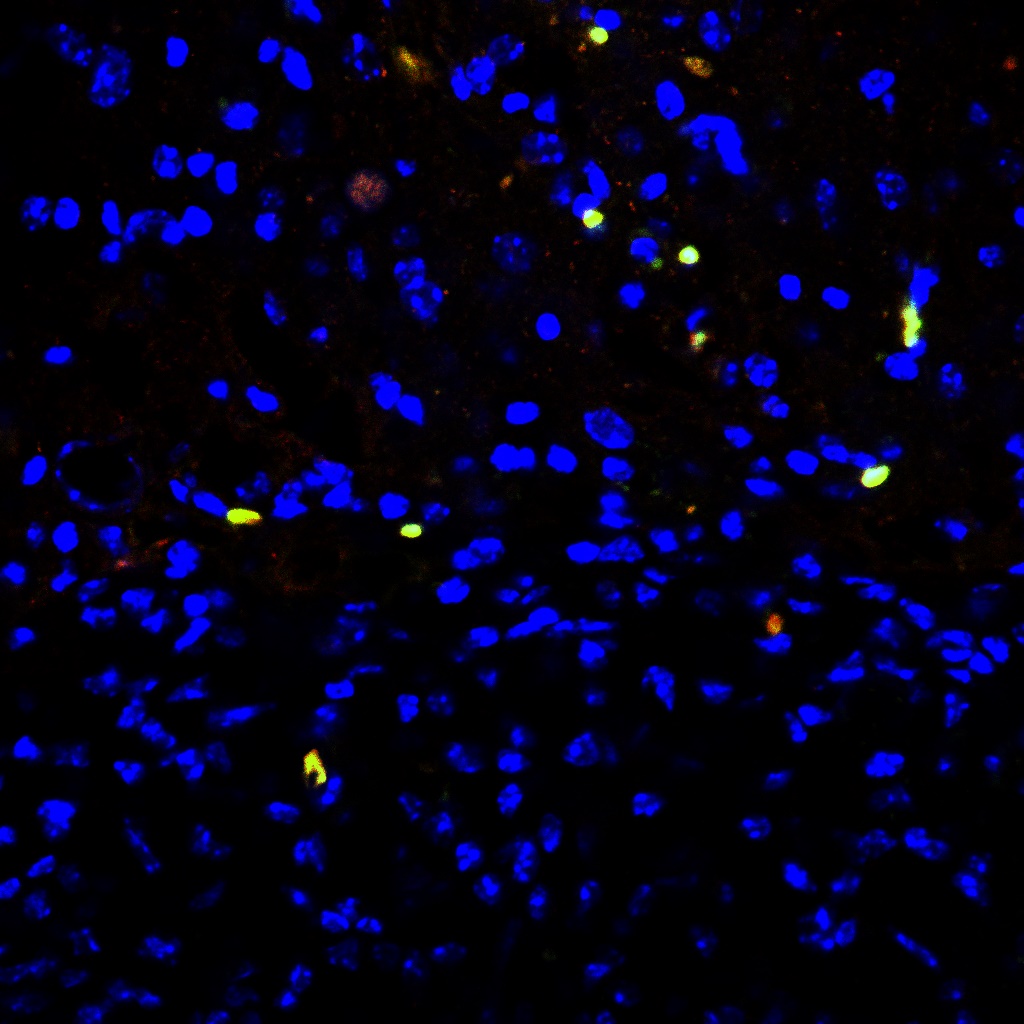

Supplement: Figure 7—source data 1. [file elife-90184-fig7-data1.zip › Figure 7-Source data 1. Raw Images for Figure 7/NFH and MBP images/Injury/Image 9-╡Ñ╕÷╬─╝■╡╝│÷-06_c1+2+3.jpg]

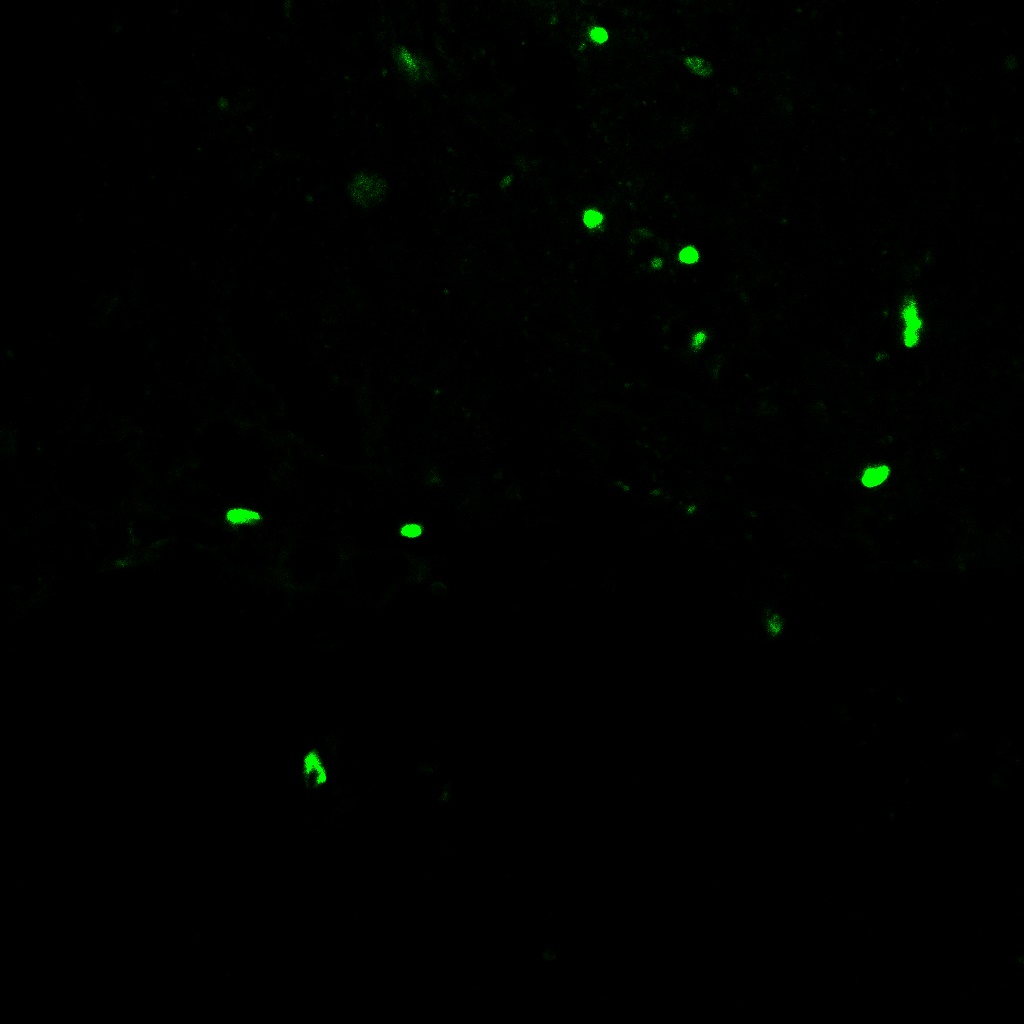

Supplement: Figure 7—source data 1. [file elife-90184-fig7-data1.zip › Figure 7-Source data 1. Raw Images for Figure 7/NFH and MBP images/Injury/Image 9-╡Ñ╕÷╬─╝■╡╝│÷-06_c2.jpg]

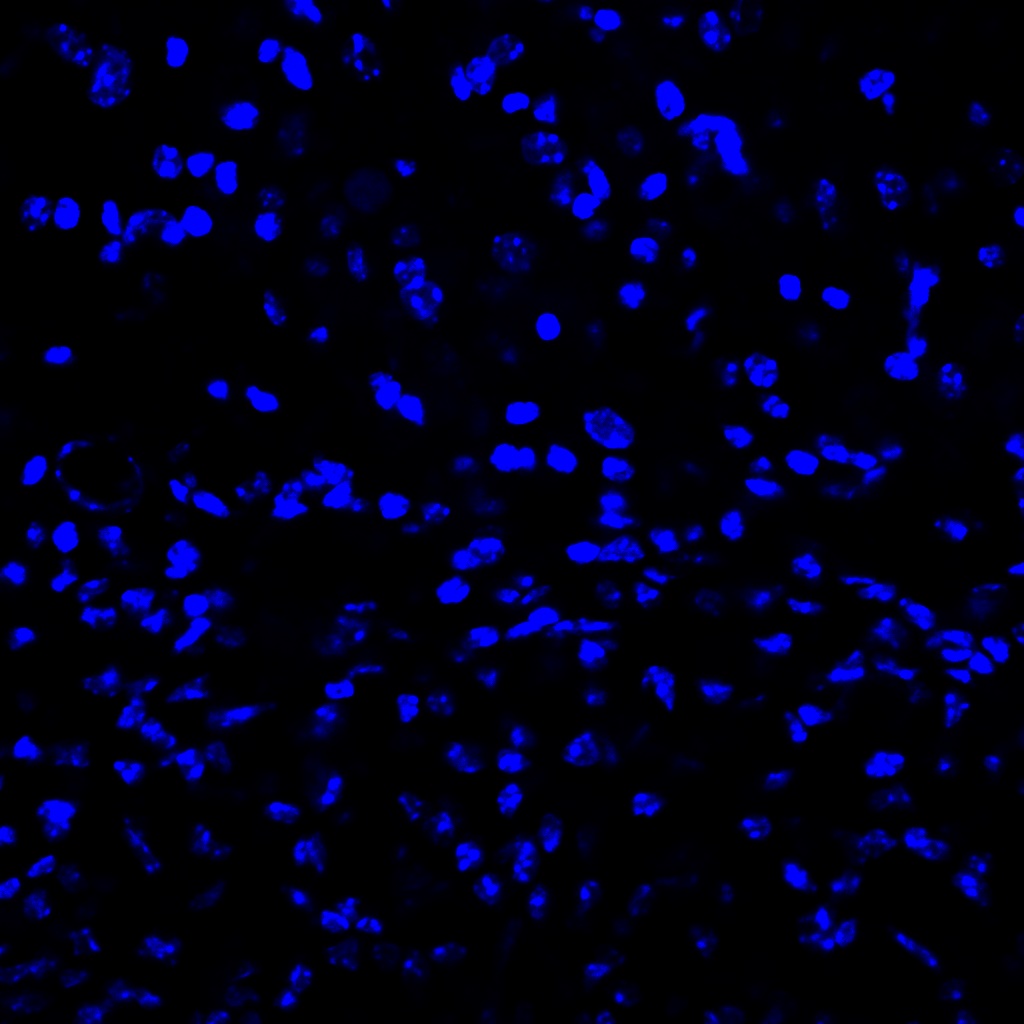

Supplement: Figure 7—source data 1. [file elife-90184-fig7-data1.zip › Figure 7-Source data 1. Raw Images for Figure 7/NFH and MBP images/Injury/Image 9-╡Ñ╕÷╬─╝■╡╝│÷-06_c3.jpg]

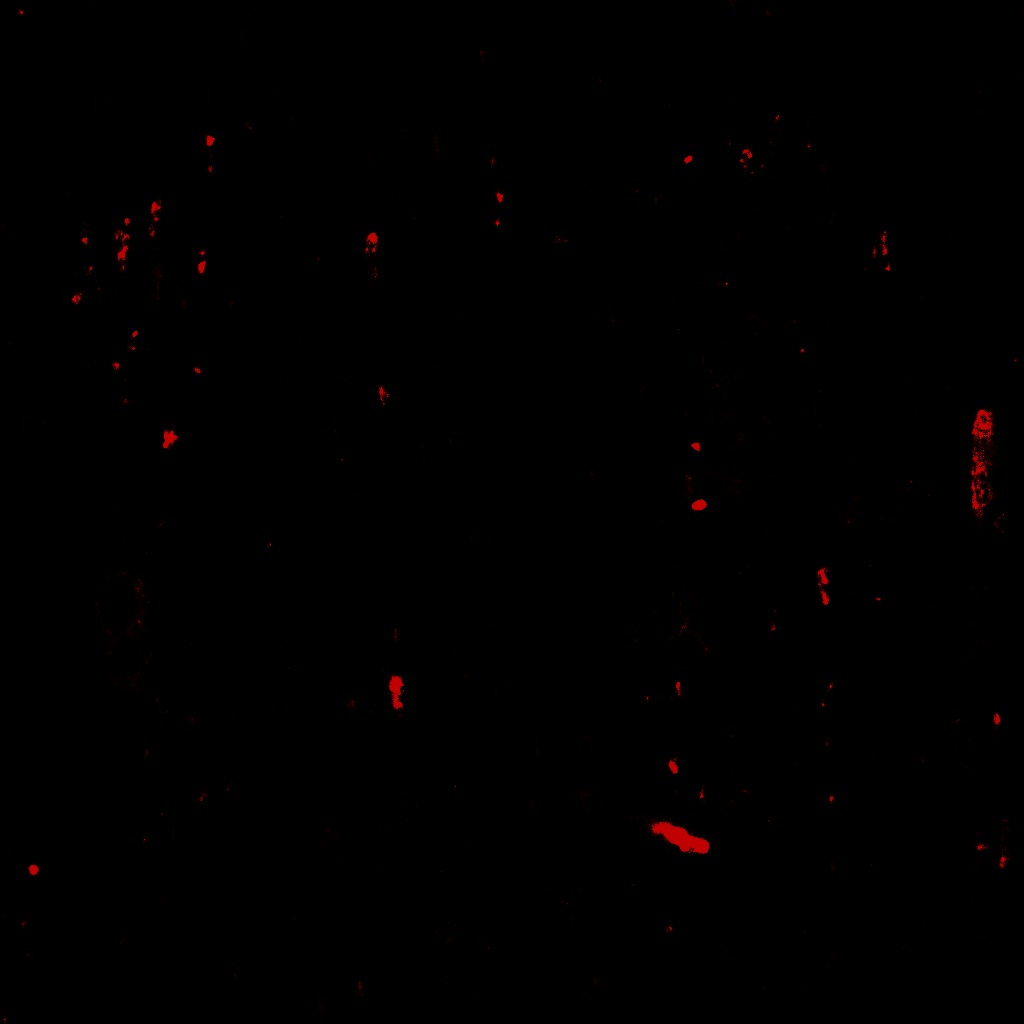

Supplement: Figure 7—source data 1. [file elife-90184-fig7-data1.zip › Figure 7-Source data 1. Raw Images for Figure 7/NFH and MBP images/Sham/Image 4-╡Ñ╕÷╬─╝■╡╝│÷-01_c1.jpg]

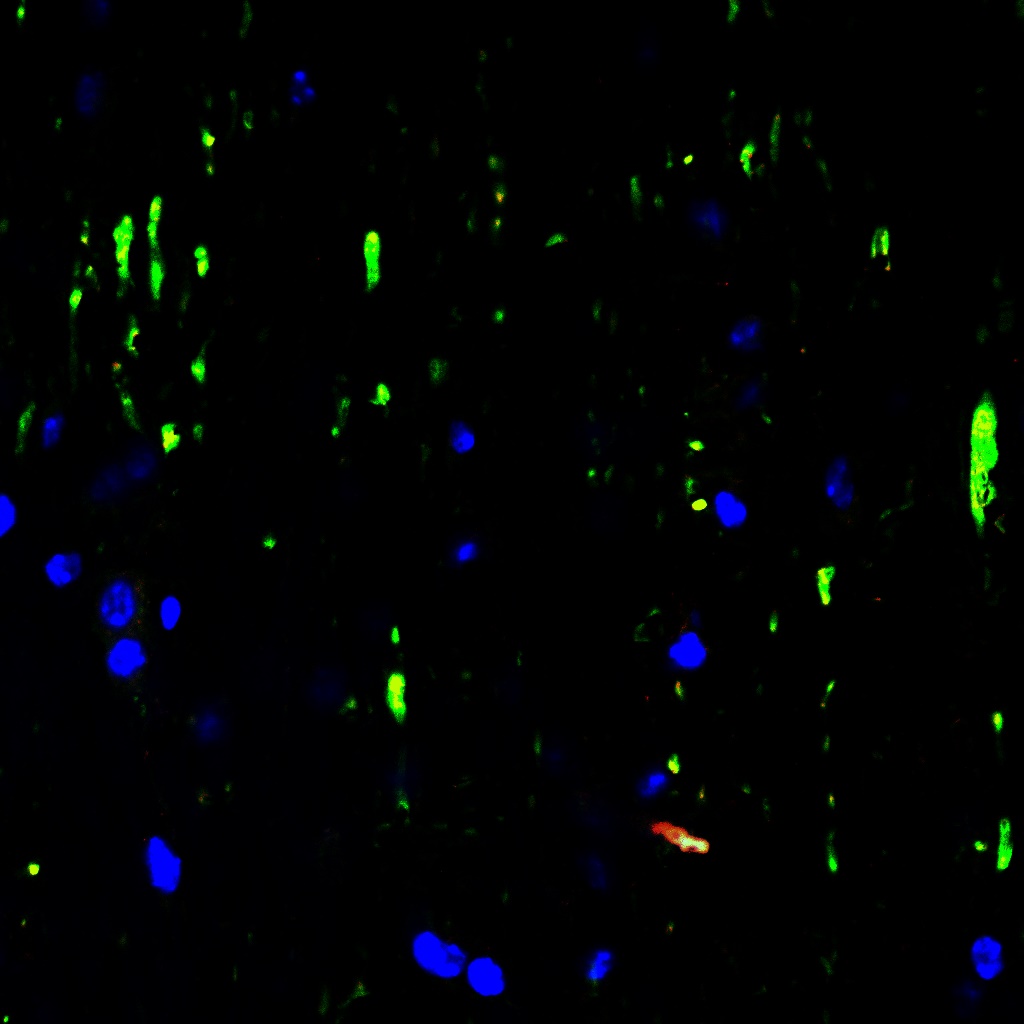

Supplement: Figure 7—source data 1. [file elife-90184-fig7-data1.zip › Figure 7-Source data 1. Raw Images for Figure 7/NFH and MBP images/Sham/Image 4-╡Ñ╕÷╬─╝■╡╝│÷-01_c1+2+3.jpg]

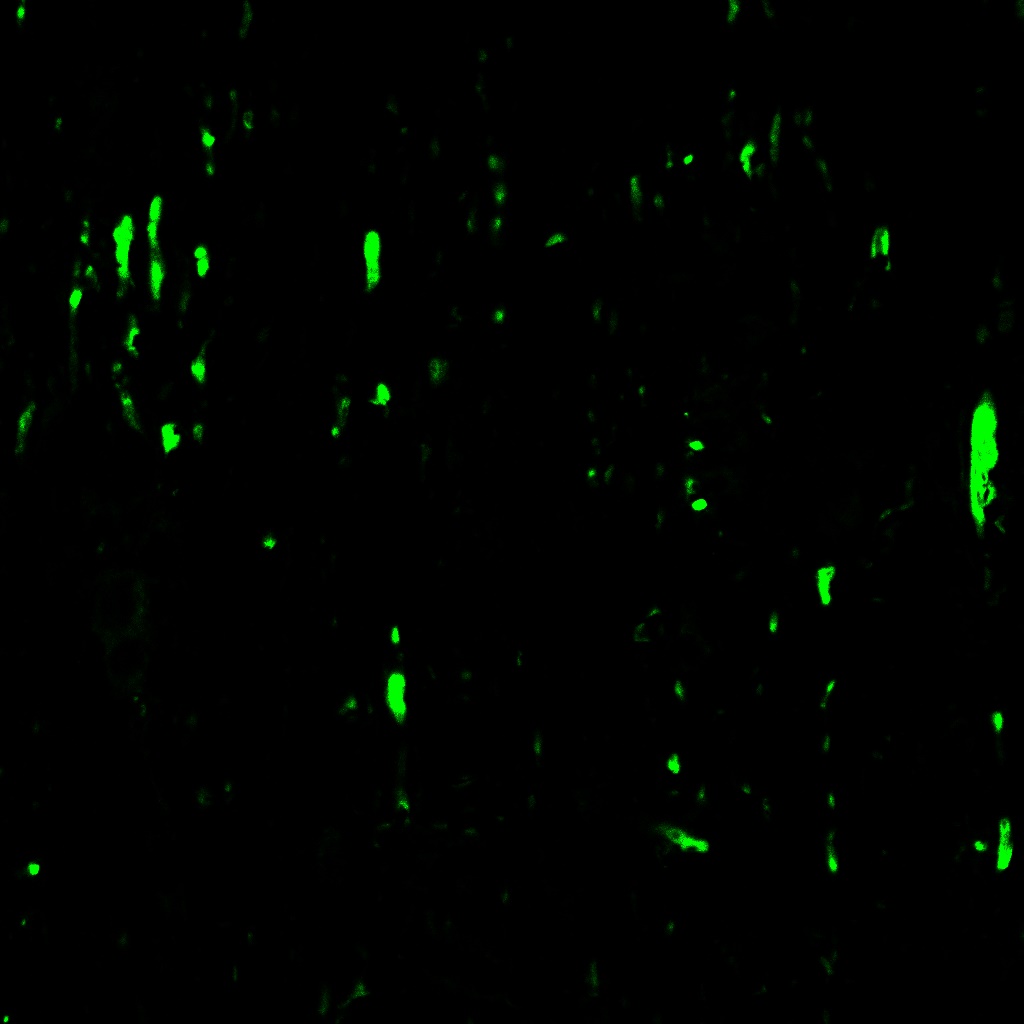

Supplement: Figure 7—source data 1. [file elife-90184-fig7-data1.zip › Figure 7-Source data 1. Raw Images for Figure 7/NFH and MBP images/Sham/Image 4-╡Ñ╕÷╬─╝■╡╝│÷-01_c2.jpg]

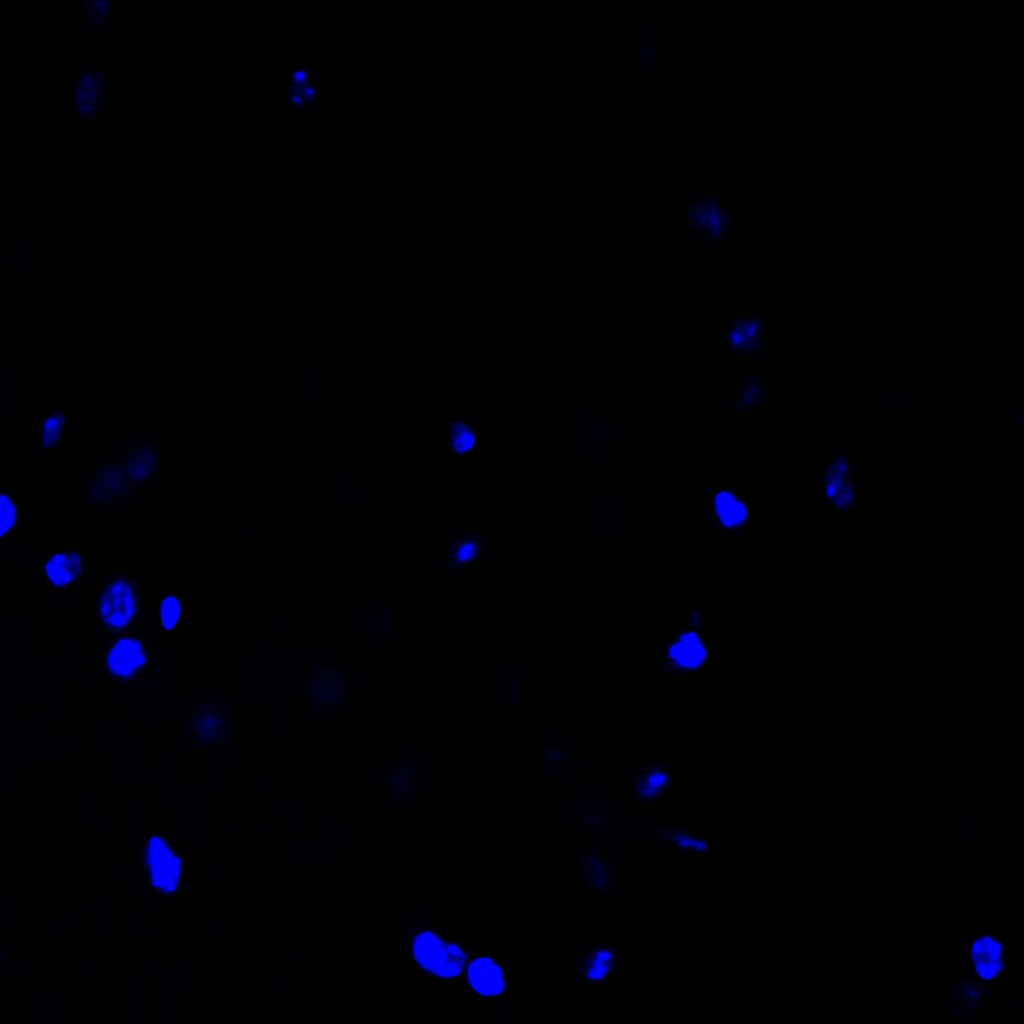

Supplement: Figure 7—source data 1. [file elife-90184-fig7-data1.zip › Figure 7-Source data 1. Raw Images for Figure 7/NFH and MBP images/Sham/Image 4-╡Ñ╕÷╬─╝■╡╝│÷-01_c3.jpg]

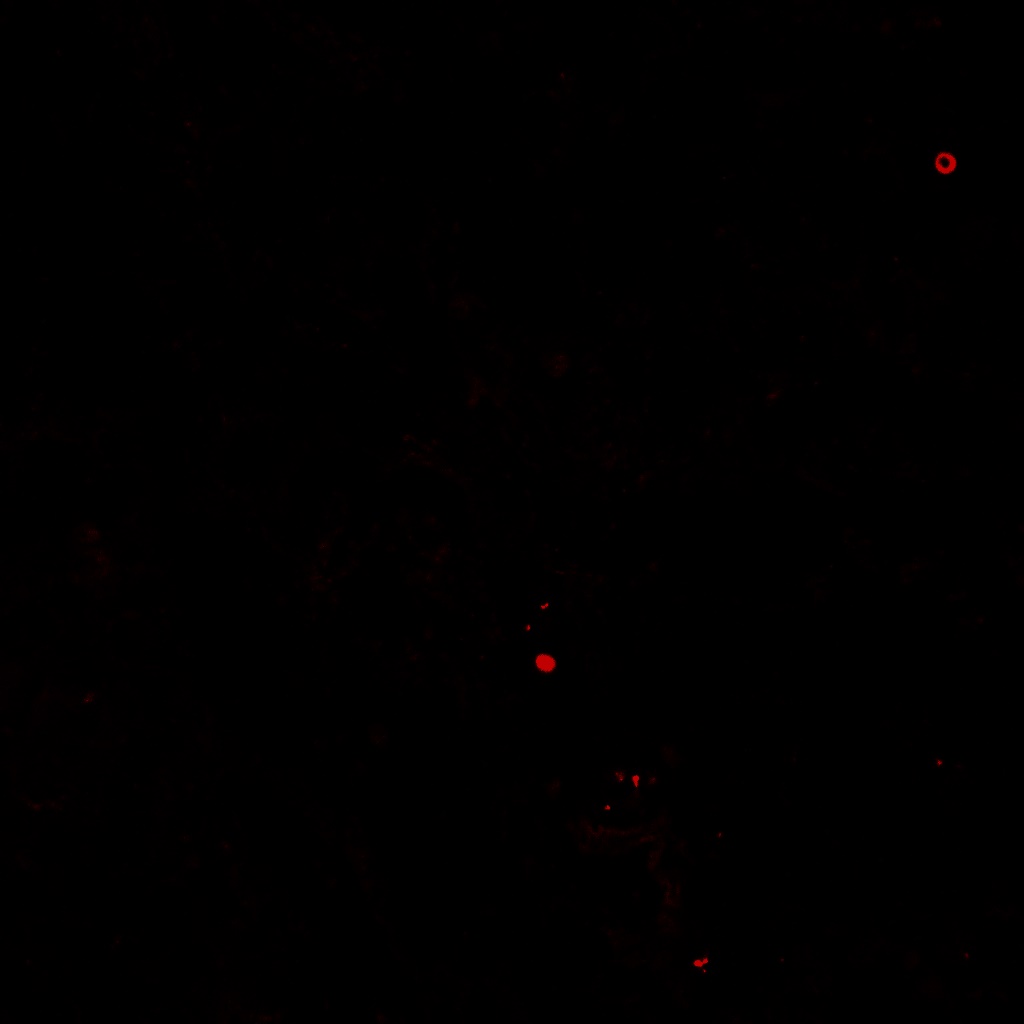

Supplement: Figure 7—source data 1. [file elife-90184-fig7-data1.zip › Figure 7-Source data 1. Raw Images for Figure 7/NFH and MBP images/Zoline/Image 2-╡Ñ╕÷╬─╝■╡╝│÷-08_c1.jpg]

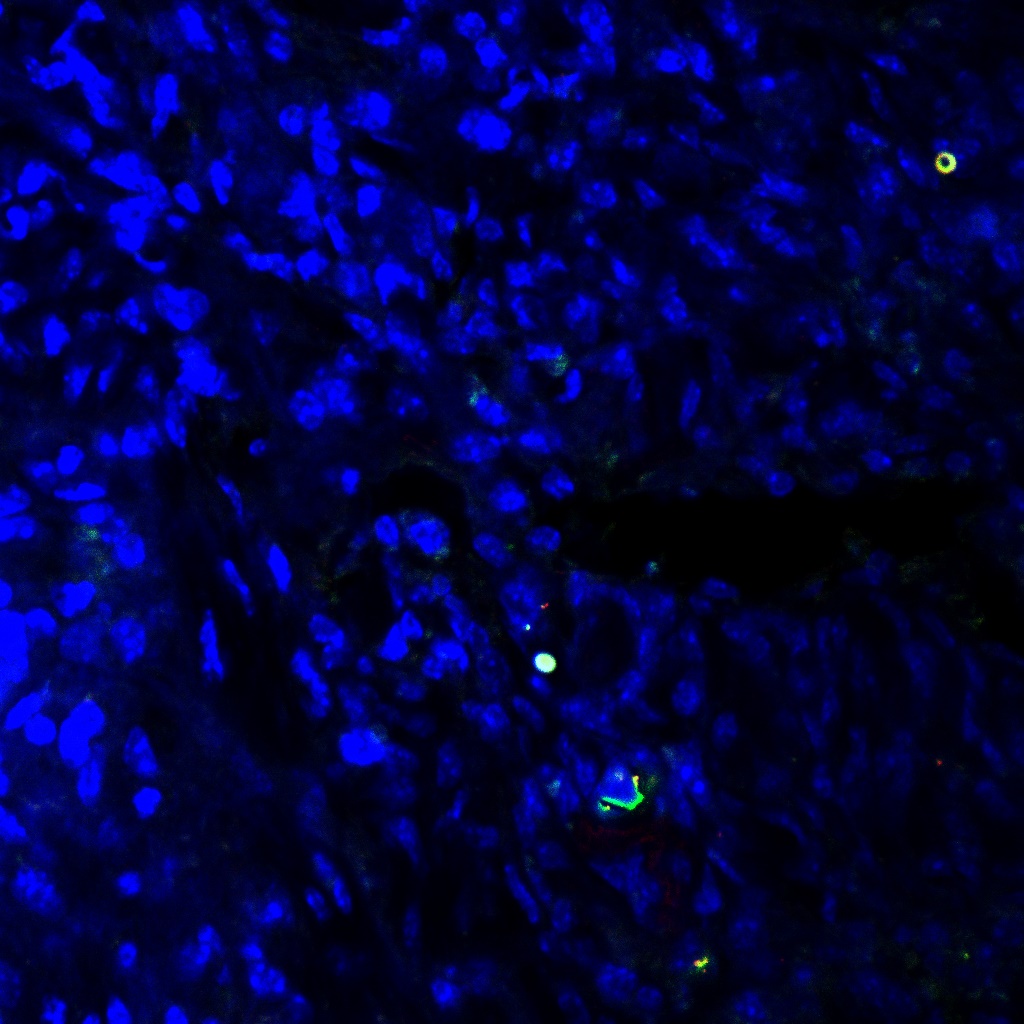

Supplement: Figure 7—source data 1. [file elife-90184-fig7-data1.zip › Figure 7-Source data 1. Raw Images for Figure 7/NFH and MBP images/Zoline/Image 2-╡Ñ╕÷╬─╝■╡╝│÷-08_c1+2+3.jpg]

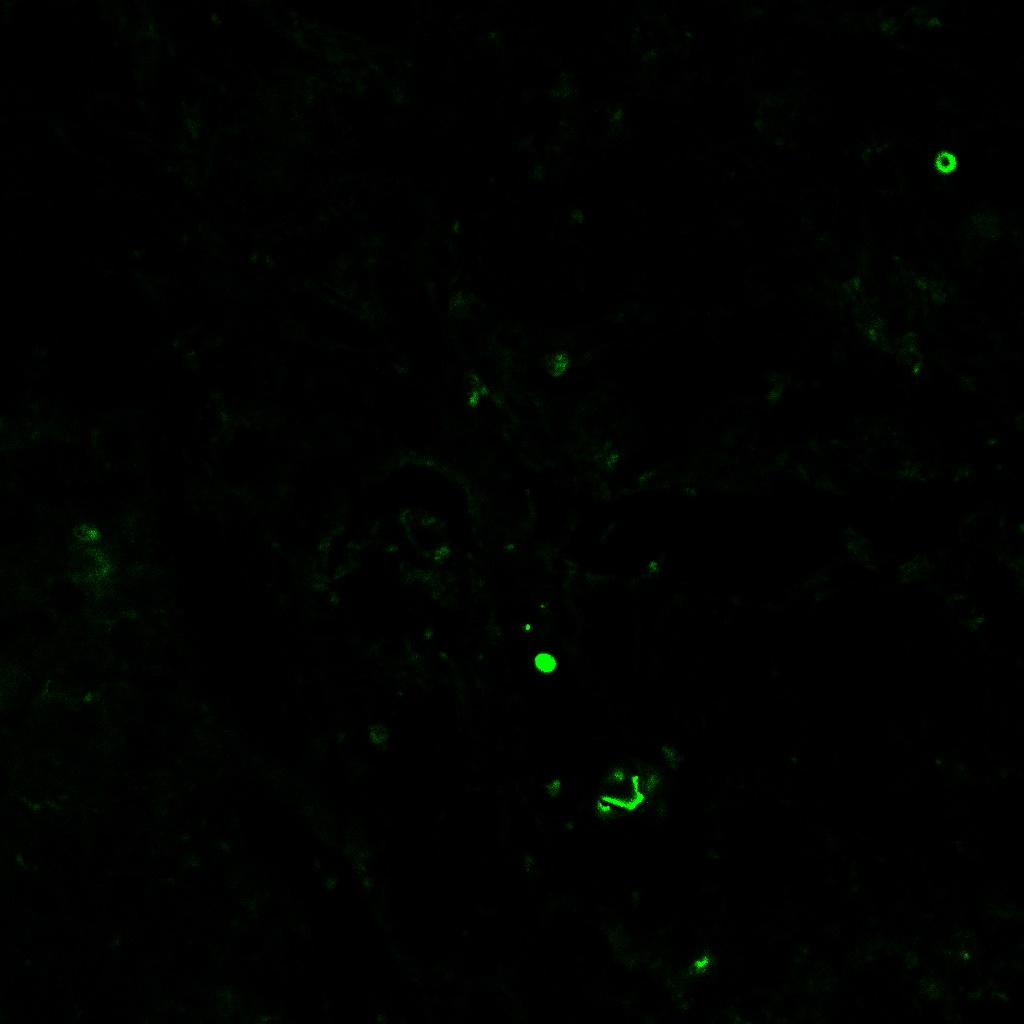

Supplement: Figure 7—source data 1. [file elife-90184-fig7-data1.zip › Figure 7-Source data 1. Raw Images for Figure 7/NFH and MBP images/Zoline/Image 2-╡Ñ╕÷╬─╝■╡╝│÷-08_c2.jpg]

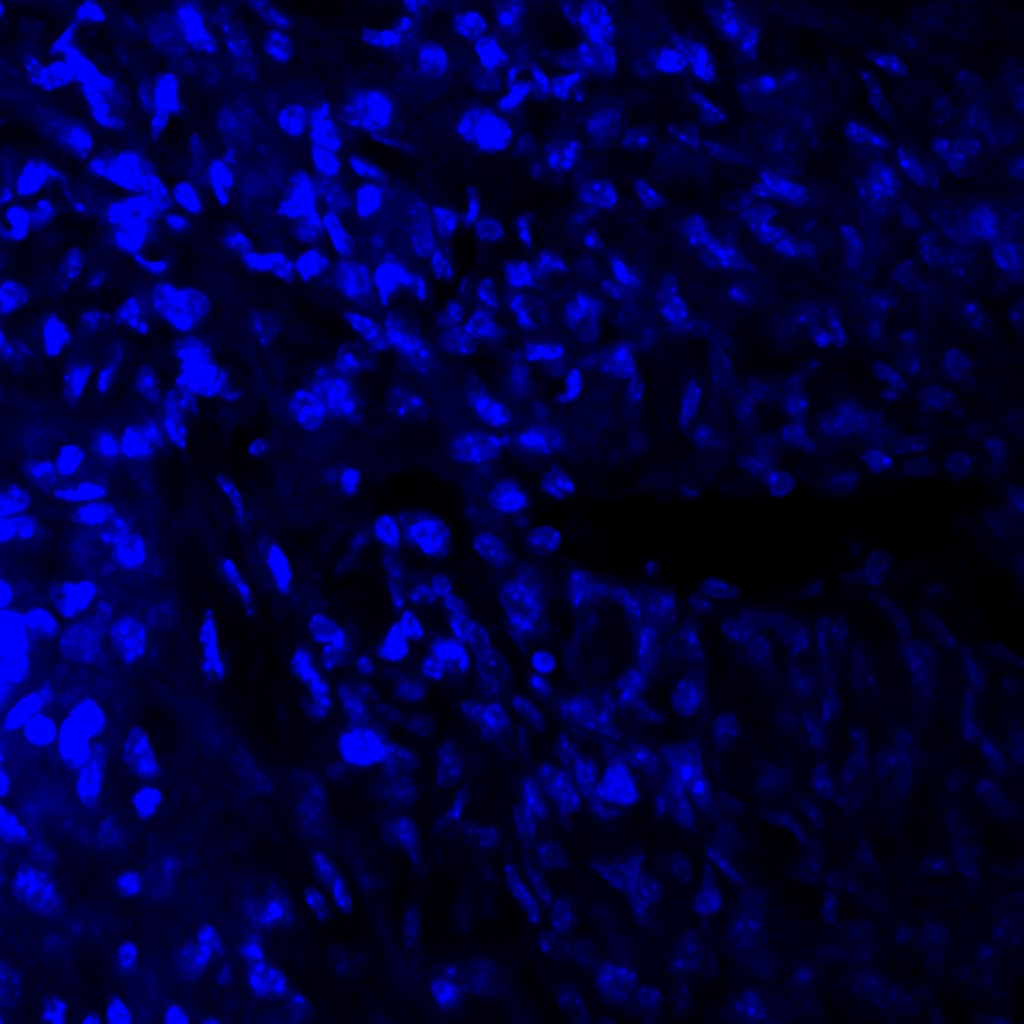

Supplement: Figure 7—source data 1. [file elife-90184-fig7-data1.zip › Figure 7-Source data 1. Raw Images for Figure 7/NFH and MBP images/Zoline/Image 2-╡Ñ╕÷╬─╝■╡╝│÷-08_c3.jpg]
